# Single-cell resolution characterization of myeloid-derived cell states with implication in cancer outcome

**DOI:** 10.1038/s41467-024-49916-4

**Published:** 2024-07-07

**Authors:** Gabriela Rapozo Guimarães, Giovanna Resk Maklouf, Cristiane Esteves Teixeira, Leandro de Oliveira Santos, Nayara Gusmão Tessarollo, Nayara Evelin de Toledo, Alessandra Freitas Serain, Cristóvão Antunes de Lanna, Marco Antônio Pretti, Jéssica Gonçalves Vieira da Cruz, Marcelo Falchetti, Mylla M. Dimas, Igor Salerno Filgueiras, Otavio Cabral-Marques, Rodrigo Nalio Ramos, Fabiane Carvalho de Macedo, Fabiana Resende Rodrigues, Nina Carrossini Bastos, Jesse Lopes da Silva, Edroaldo Lummertz da Rocha, Cláudia Bessa Pereira Chaves, Andreia Cristina de Melo, Pedro M. M. Moraes-Vieira, Marcelo A. Mori, Mariana Boroni

**Affiliations:** 1 Laboratory of Bioinformatics and Computational Biology, Division of Experimental and Translational Research, Brazilian National Cancer Institute (INCA), Rio de Janeiro, RJ Brazil; 2 Department of Microbiology, Immunology, and Parasitology, Federal University of Santa Catarina, Florianópolis, SC Brazil; 3 Department of Immunology, Institute of Biomedical Sciences, University of São Paulo,(USP), São Paulo, Brazil; 4 Instituto D’Or de Ensino e Pesquisa, São Paulo, Brazil; 5 Department of Medicine, Division of Molecular Medicine, Laboratory of Medical Investigation 29, School of Medicine, University of São Paulo (USP), São Paulo, Brazil; 6 Laboratory of Medical Investigation in Pathogenesis and Directed Therapy in Onco-Immuno-Hematology (LIM-31), Departament of Hematology and Cell Therapy, Hospital das Clínicas HCFMUSP, School of Medicine, University of São Paulo (USP), São Paulo, Brazil; 7 Division of Pathology, Brazilian National Cancer Institute (INCA), Rio de Janeiro, RJ Brazil; 8 Division of Clinical Research and Technological Development, Brazilian National Cancer Institute (INCA), Rio de Janeiro, RJ Brazil; 9 Gynecologic Oncology Section, Brazilian National Cancer Institute (INCA), Rio de Janeiro, RJ Brazil; 10 Laboratory of Immunometabolism, Department of Genetics, Evolution, Microbiology, and Immunology, Institute of Biology, Universidade Estadual de Campinas, Campinas, SP Brazil; 11 Obesity and Comorbidities Research Center (OCRC), Universidade Estadual de Campinas, Campinas, SP Brazil; 12 Experimental Medicine Research Cluster (EMRC), Universidade Estadual de Campinas, Campinas, SP Brazil; 13 Laboratory of Aging Biology, Department of Biochemistry and Tissue Biology, Universidade Estadual de Campinas, Campinas, SP Brazil

**Keywords:** Molecular medicine, Prognostic markers, Cancer microenvironment, Tumour immunology

## Abstract

Tumor-associated myeloid-derived cells (MDCs) significantly impact cancer prognosis and treatment responses due to their remarkable plasticity and tumorigenic behaviors. Here, we integrate single-cell RNA-sequencing data from different cancer types, identifying 29 MDC subpopulations within the tumor microenvironment. Our analysis reveals abnormally expanded MDC subpopulations across various tumors and distinguishes cell states that have often been grouped together, such as TREM2+ and FOLR2+ subpopulations. Using deconvolution approaches, we identify five subpopulations as independent prognostic markers, including states co-expressing TREM2 and PD-1, and FOLR2 and PDL-2. Additionally, TREM2 alone does not reliably predict cancer prognosis, as other TREM2+ macrophages show varied associations with prognosis depending on local cues. Validation in independent cohorts confirms that FOLR2-expressing macrophages correlate with poor clinical outcomes in ovarian and triple-negative breast cancers. This comprehensive MDC atlas offers valuable insights and a foundation for futher analyses, advancing strategies for treating solid cancers.

## Abstract (web summary)

Tumour-associated myeloid cells have been linked to patient outcome and treatment response in multiple cancer types. Here, the authors use deconvolution of single cell RNA-sequencing data to identify myeloid populations which are prognostic across cancer types.

## Introduction

The tumor microenvironment (TME) represents a dynamic network consisting of diverse cell types and acellular components that intricately interact with malignant cells. The understanding of immune cells within the TME, particularly lymphocytes, has paved the way for the development of immunotherapies capable of inducing long-lasting responses across a wide spectrum of cancers^1^. However, the current immune-checkpoint inhibitor therapies only confer benefits to a subset of individuals^2^, justifying a better comprehension of other key immune cells with protumoral activities. Among the crucial players in the TME are myeloid-derived cells (MDCs), comprising a heterogeneous array of populations including monocytes, macrophages, conventional dendritic cells (cDC), and polymorphonuclear granulocytes. These MDCs exhibit remarkable plasticity, allowing them to adopt various cellular states and perform a wide range of functions when exposed to different niches^3^. Intriguingly, while MDCs possess the capacity to enhance cancer cell phagocytosis and induce cytotoxic tumor death when properly activated^4,5^, they can also foster tumor growth by facilitating and sustaining cancer hallmarks^6^.

The majority of MDCs originate from bone marrow progenitor cells and subsequently migrate into the TME. Macrophages, however, can derive from two primary sources: 1) erythro-myeloid progenitors in the yolk sac and fetal liver before birth, which exhibit enriched expression of *FOLR2*, *PLTP*, and *LYVE1* markers^7–9^; and 2) circulating monocytes originating from the bone marrow and recruited into the tumor^10^. Monocyte-derived macrophages have a transient lifespan and necessitate constant replenishment from circulating monocytes^11^. Their ontogeny can be indicated by the expression of monocyte-related markers such as *FN1*, *SELL*, and *VCAN*^12^.

Macrophages, regardless of their origin, can exhibit distinct phenotypes. Macrophages have been classified into two main polarization states: classically activated M1, characterized by a proinflammatory phenotype, and alternatively activated M2, associated with tissue remodeling and/or anti-inflammatory properties^10^. In the context of the TME, M2 macrophages are commonly referred to as tumor-associated macrophages (TAMs), and their presence has been correlated with poor prognosis in several tumor types^13–15^. However, it is important to note that not all TAMs display a clear M1 or M2 phenotype, as they often express markers associated with both polarization states^16,17^. This underscores the need to move beyond the simplistic M1/M2 dichotomy and delineate distinct TAM states.

TAMs play a multifaceted role within the TME, influencing tumor growth, epithelial-mesenchymal plasticity, extracellular matrix remodeling, cell invasion, and angiogenesis, subsequently impacting tumor progression, metastasis, and therapy resistance. Furthermore, the interaction between TAMs and malignant cells can facilitate immune evasion through TME shaping^18^. Given the pivotal role of TAMs and other MDCs in promoting tumor growth and metastasis, they have emerged as promising targets for cancer therapy. Numerous strategies aimed at depleting or modulating the functional/phenotypic reprogramming, infiltration, or activation of TAMs are being explored^19^. However, the lack of precise markers to distinguish between MDC subpopulations and states poses a challenge to the effectiveness of these treatments. Therefore, a thorough characterization of MDC populations within the TME is urgently required to overcome this limitation.

While recent studies have utilized high-resolution technologies such as single-cell RNA-sequencing (scRNA-Seq) and spatial transcriptomics to investigate the immune landscape of different cancer types, a comprehensive pan-cancer integrated analysis encompassing MDCs in the TME with associations with cancer prognostic has been lacking. To address this critical gap, we employed data from three distinct scRNA-Seq technologies, integrating them to construct a comprehensive pan-cancer catalog of MDC subpopulations. Our analysis incorporated data from seven solid tumor types, enabling a thorough characterization of tumor-associated cells. Consequently, we identified and characterized MDC populations/states within these tumors uncovering abnormally expanded MDC subpopulations associated with a poor prognosis across various tumor origins. These findings pave the way for the development of effective targeted immunotherapy strategies aimed at specific MDC subpopulations.

## Results

### A thorough integration strategy recovers an in-depth pan-cancer repertoire of MDCs in the tumor microenvironment

**Fig. 1:**
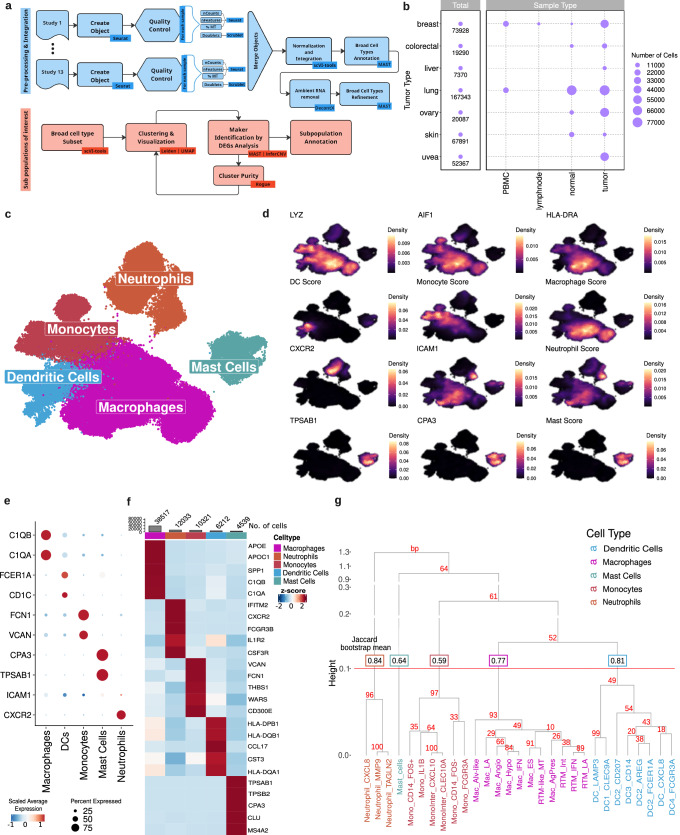
Multi-tissue single-cell atlas of tumor and healthy samples. **a** Workflow for integrative analysis of single-cell RNA-sequencing data outlining a comprehensive pipeline from preprocessing multiple scRNA-Seq studies through quality control, data integration, and normalization, to the identification and annotation of cell subpopulations. Key analytic tools are noted at each step, including Seurat for object creation and quality control, scVI-tools for data normalization and integration, and other methods used for cell type annotation, ambient RNA removal, and cluster purity assessment. **b** Dot plot showing the distribution of sample types across the tissues and peripheral blood mononuclear cells (PBMC) analyzed in this study. Dot size indicates the number of cells by sample type. **c** Uniform Manifold Approximation and Projection (UMAP) of 51,687 myeloid-derived cells, color-coded by cell types. **d** Density plots highlighting the expression of gene/score for each cell. **e** Dot plot showing the expression of specific markers by each myeloid-derived subpopulation. Dot size indicates the percent of expressing cells, and the color is the scaled average expression. **f** Heatmap showing the proportion of cells and their respective specific markers from each MDC. The color scale represents the scaled expression of each gene. **g** Dendrogram represents the hierarchical clustering of MDCs based on their gene expression similarity. The branch’s height indicates the distance or dissimilarity between clusters, with a lower height reflecting greater similarity. The Jaccard bootstrap mean values are overlaid on the dendrogram, measuring cluster stability based on resampling. Values closer to 1 indicate higher confidence. The dashed line across the dendrogram serves as a cut-off threshold for defining distinct clusters based on Jaccard similarity. The colored boxes correspond to broad cell types categorized by their predominant function or phenotype. Each terminal node of the dendrogram is labeled with the subcluster name derived from the expression of key marker genes. The values in red on each node represent the bootstrap percentage related to the confidence of the node’s position. A total of 10.000 replicates were used for bootstrapping. Source data are provided as a Source Data file. %MT - percentage of transcripts that map to mitochondrial genes.

To define the landscape of cell subtypes in the TME, we integrated scRNA-Seq data comprising 392,204 high-quality cells from 13 public datasets (Supplementary Data 1 and Supplementary Fig. 1a). The integrated datasets comprised three different technologies (10x Genomics, InDrop, and Smart-Seq2) (Supplementary Fig. 1b) and samples from different anatomical sites of healthy donors and patients diagnosed with different cancer types, totaling 138 individuals (Supplementary Data 2). Our pipeline allowed for the integration of a heterogeneous group of datasets, and even though we applied stringent quality control, it retained crucial biological variation while efficiently removing typical contaminants such as doublets and ambient RNA, as well as noise due to batch effect (Fig. 1a). The final integrated dataset encompassed seven tumors, including breast, colorectal, liver, lung, ovary, skin, and uveal melanomas, as well as their adjacent tissue counterparts, metastatic samples, blood samples, and adjacent lymph nodes (Fig. 1b).

Using our approach, we identified homogeneous cell groups distributed in clusters. The clusters were annotated based on canonical gene markers and functional signatures (Supplementary Data 3 and 4) of major cell types and differentially expressed genes (DEGs) (Supplementary Data 5 and Supplementary Fig. 1c–f), yielding 10 broadly recognized cell types. Malignant cells were identified by exhibiting a copy number variation (CNV) estimate above the median value of non-malignant cells in the TME (Supplementary Fig. 1c). Low-quality cells were annotated as non-identifiable, and were removed for further analysis (Supplementary Fig. 1d). MDCs were the second-largest group of cells in the TME (Supplementary Fig. 1e), and their proportion in tumor samples was 1.74 times greater than that in normal samples (Supplementary Fig. 1g). The percentage of these cells varied across sample types from 1.7% to 25%, except for metastatic lung samples, where over 60% of TME cells matched mononuclear phagocytes (Supplementary Fig. 1h).

Among the MDC subpopulations (Fig. 1c–g), mononuclear phagocytes (n = 51,687), positive for *LYZ*, *AIF1*, and *HLA-DRA*, were the majority. Other populations, such as neutrophils, marked by *CXCR2* and *ICAM1*, and mast cells, identified by *TPSAB1* and *CPA3* expression, were also largely detected. Megakaryocytes were the smallest group among MDCs (n = 185) and were not further investigated in this study (Supplementary Fig. 1d).

To characterize the MDC subpopulations, we conducted unsupervised clustering and identified five major lineages (Fig. 1e, f): mast cells (n = 4539), neutrophils (n = 12,033), DCs (n = 6212), monocytes (n = 10,321), and macrophages (n = 38,517). Additional unsupervised hierarchical clustering analysis of the MDCs subpopulations, anchored in 750 highly variable genes, substantiates the delineation of the principal five broad cell types (Fig. 1g). The dendrogram reinforces the proximity of mononuclear phagocytes and elucidates a closer gene expression similarity between macrophages and dendritic cells compared to their expression relationship with monocytes. This observation suggests shared functional or ontogenic pathways between macrophages and dendritic cells. To avoid subjective and arbitrary definitions of cell clusters, we employed entropy-based statistics to accurately quantify the purity of each cluster. Our approach improved the ability to detect and define robust signatures, ranging from common cell states to less frequent ones, performing better than previous strategies that evaluated small and isolated datasets^20,21^. Broad cell types displayed on average purity scores of 0.95 (values above 0.9 are recommended as a pure cluster) (Supplementary Fig. 1i,j). Our findings provide new insights into the MDC subpopulations and contribute to a better characterization of their distribution in the TME.

### MDCs are heterogeneous and phenotypically diverse across tumor samples

Mast cells and neutrophils (Fig. 1c–g, and Supplementary Fig. 2) corresponded to the less frequent MDC subpopulations identified in our study, and given their low pan-cancer representativeness, they have not been further studied here in detail.

**Fig. 2:**
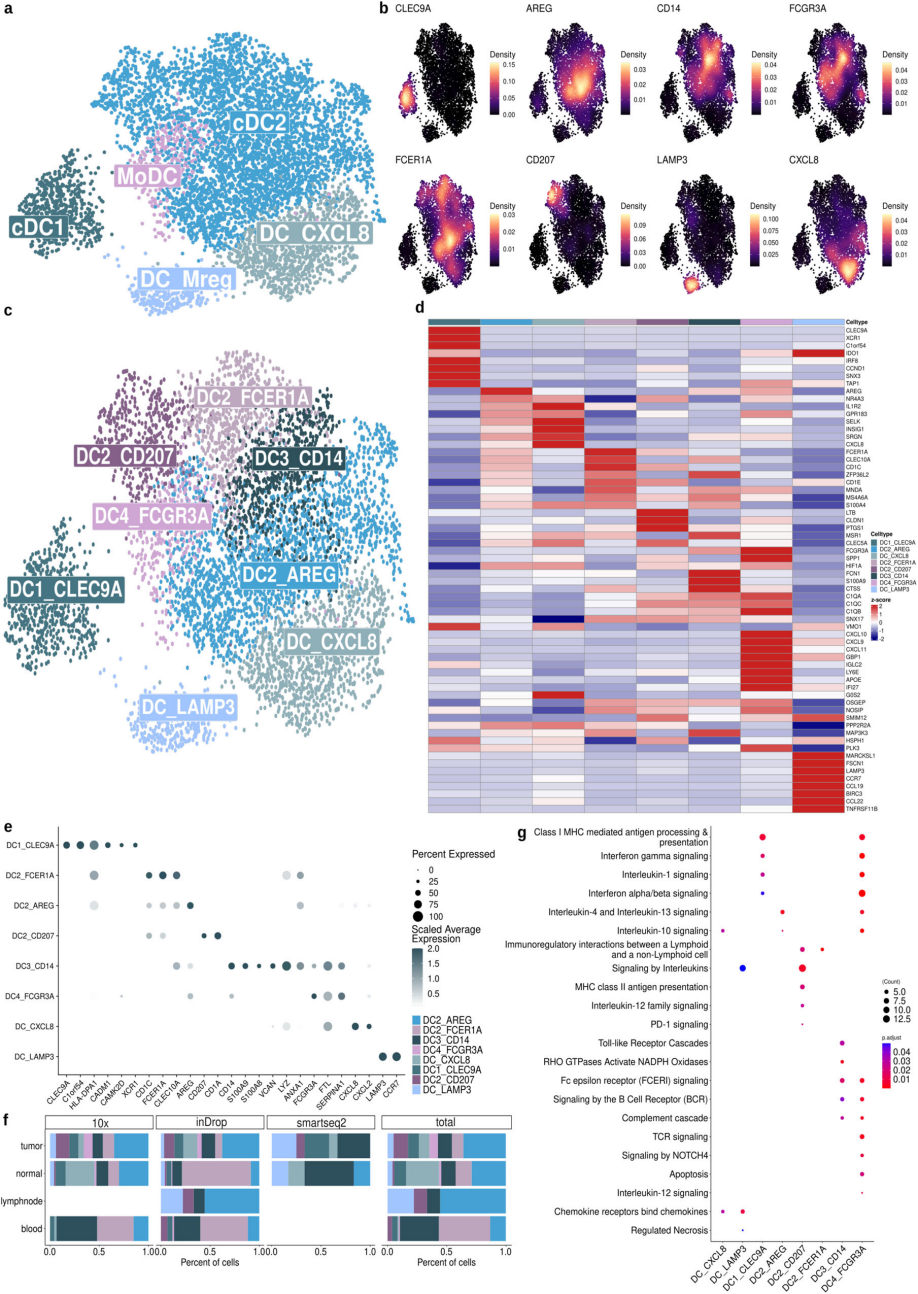
Characterization of dendritic cell subpopulations. **a** UMAP color-coded by the broad classification of dendritic cells. **b** Density plots highlighting the gene expression of each cell for specific markers. **c** UMAP of DC subpopulations colored by eight states: DC1_CLE9A, DC2_207, DC2_AREG, DC2_FCER1A, DC3_CD14, DC4_FCGR3A, DC_CXCL8 and DC_LAMP3. **d** Heatmap showing the DEGs per cluster. The color scale represents the scaled expression of each gene. **e** Dot plot showing the mean expression of genes related to DC cell subpopulations. Dot size indicates the fraction of expressing cells, colored based on normalized expression levels. **f** Bar plot showing the distribution of DC cells across sample types by three different sequencing platforms (10x, inDrop, Smart-seq2), with an aggregated total. **g** Enrichment pathways analysis of DC subpopulations using Reactome database. The size of each circle represents the number of genes and such circles are colored by p-adjust corrected by Benjamini-Hochberg (BH) after one-sided Fisher’s exact test. Source data are provided as a Source Data file.

DCs were the fourth most prevalent subpopulation of MDCs, accounting for 9% of these cells (Fig. 1f). Guided by foundational studies^22^, we identified six primary DC groups (Fig. 2a). The DC1 subpopulation, marked by *CLEC9A* and *CADM1* expression, included 580 cells, while the larger group, DC2, was distinguished by *CD1C* expression and was further divided into three phenotypes as shown in Fig. 2a–d. The DC3 subpopulation, notable for *CD14* and *S100A9* markers, included 765 cells enriched within tumor samples. In contrast, the DC4 subpopulation had 314 cells expressing high levels of *FCGR3A* and *SERPINA1*, predominantly found in blood and indicative of Monocyte-derived Dendritic Cells (MoDC) (Supplementary Fig. 3a, b). Additionally, we identified the DC_CXCL8 subpopulation (n = 993) (Fig. 2c, d), expressing chemokines like *CXCL8* and *CXCL2*, and the DC_LAMP3 subpopulation (n = 285), characterized as migratory mature DCs expressing *LAMP3* and *CCR7* (Fig. 2a-d). The DC2_AREG was enriched in the tumor samples, especially in ovary, lung and breast tumors, while DC_CXCL8 was abundant in skin samples (Fig. 2e, Supplementary Fig. 3a, b). These cells showed increased expression of genes coding for PD-1 and PD-2 ligands, suggesting a potential immunosuppressive role (Supplementary Fig. 3d). Additionally, functional enrichment analysis further supported the cell’s profile (Fig. 2f).

The DC2 subpopulation was subdivided into three subpopulations/states DC2_AREG (n = 2044), enriched in tumors and lymph nodes, DC2_FCER1A (n = 726), found mostly in the blood (Fig. 2c–f and Supplementary Fig. 3a,b), and a subpopulation expressing *CD207*, a langerin protein encoder (DC2_CD207, n = 505), which was abundant in tumor samples, particularly in lung and ovarian tumors and lymph nodes (Fig. 2e and Supplementary Fig. 3b,c). Although langerin is primarily expressed by epidermal macrophages known as Langerhans cells^23,24^, DC2_CD207 is a type 2 cDC subpopulation (expressing *CD1A*) distinct from the aforementioned cell (Fig. 2c, d). DC-activating signals, such as pro-inflammatory cytokines (interferon-alpha/beta and gamma) and antigen presentation, were observed in DC1_CLEC9A and DC2_CD207. DC2_AREG appeared to have regulatory properties, including the modulation of IL-10, -4, and -13 signaling pathways (Fig. 2g and Supplementary Data 6).

**Fig. 3:**
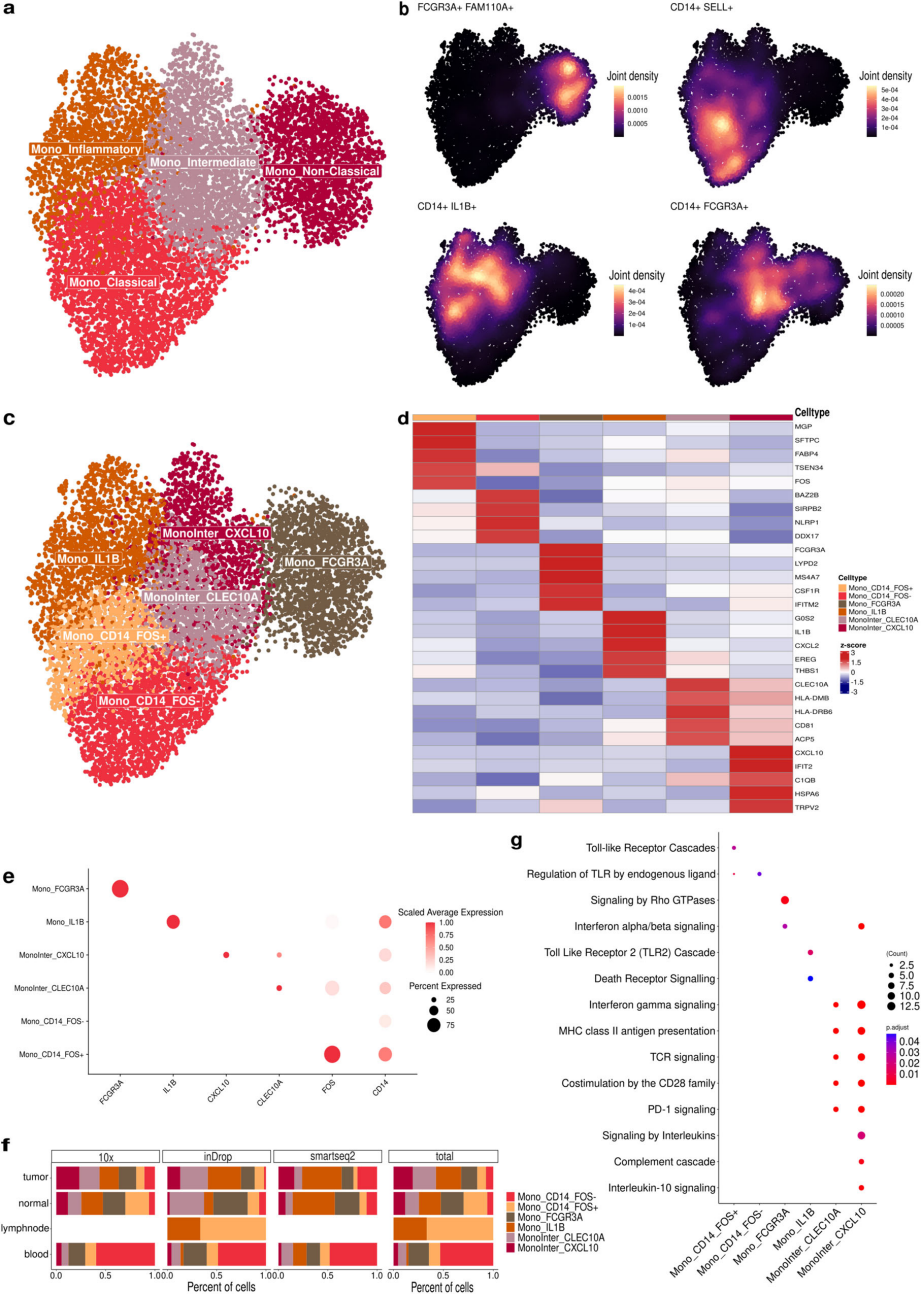
Characterization of monocytes subpopulations. **a** UMAP of monocytes subpopulations colored by four states: Mono_Inflammatory, Mono_Intermediate, Mono_Non-Classical, and Classical. **b** Density plots highlighting the gene expression and co-expression of specific genes for each cell. **c** UMAP of monocytes subpopulations colored by the six states identified. **d** Heatmap showing the DEGs per cluster. The color scale represents the scaled expression of each gene. **e** Dot plot showing the mean expression of genes related to monocytes subpopulations. Dot size indicates the percent of expressing cells, and the dot color the scaled average expression. **f** Bar plot showing the distribution of monocytes across sample types by three different sequencing platforms (10x, inDrop, Smart-seq2), with an aggregated total. **g** Enrichment pathways analysis of monocytes subpopulations using Reactome database. The size of each circle represents the number of genes and such circles are colored by p-adjust using one-sided Fisher’s exact test with BH multiple-testing correction. Source data are provided as a Source Data file.

Monocytes are precursors of tumor-infiltrating myeloid cells, and we identified four major groups (Non-Classical, Classical, Inflammatory, and Intermediate) (Fig. 3a, b), reflecting six states with distinct gene programs (Fig. 3c–e). The Mono_FCG3RA (n = 2018), exhibited a non-classical phenotype characterized by the co-expression of *FCGR3A* and *FAM110A* within a single cluster (Fig. 3b–e). The classical phenotype was marked by the expression of common monocyte markers such as *CD14* and *SELL* (Fig. 3b, e), and was further differentiated into two states based on *FOS* expression: Mono_CD14_FOS^−^ (n = 2150) and Mono_CD14_FOS^+^ (n = 1497) (Fig. 3c-e). Mono_CD14_FOS^+^ was enriched in lymph nodes and normal tissues, such as the ovary (Supplementary Fig. 4a–c), while Mono_CD14_FOS^-^ was more prevalent in blood samples (Fig. 3f and Supplementary Fig. 4a–c). These two states are poorly described, and their functions remain unclear, although both are enriched in Toll-like receptor activation pathways (Fig. 3g and Supplementary Data 6).

Additionally, among the Mono_Inflammatory group, the Mono_IL1B (n = 1739) was identified as displaying a pro-inflammatory profile with high expression levels of *IL1B*, *CXCL2*, and *EREG* (Fig. 3c–e), as well as other inflammatory genes (Supplementary Fig. 4d and Data 4) with increased expression of genes involved in Toll-like receptor pathways and death receptor ligands that activate caspase cascades (Fig. 3g).

We were also able to identify two monocyte clusters displaying an intermediate phenotype between monocytes and antigen-presenting cells, expressing both genes coding for CD16 and CD14. We called this group Mono_Inter, from which *CXCL10* and *CLEC10A* expressions divided this monocytes into MonoInter_CXCL10 (n = 1238) and MonoInter_CLEC10A (n = 1559), respectively (Fig. 3c–e). These cells expressed markers related to antigen presentation, such as *HLA-DMB* and *HLA-DRB6* (Fig. 3d), and were enriched in MHC class II antigen presentation, interferon/gamma signaling, and costimulation by CD28 pathways, as well as PD-1 signaling (Fig. 3g and Supplementary Data 5).

**Fig. 4:**
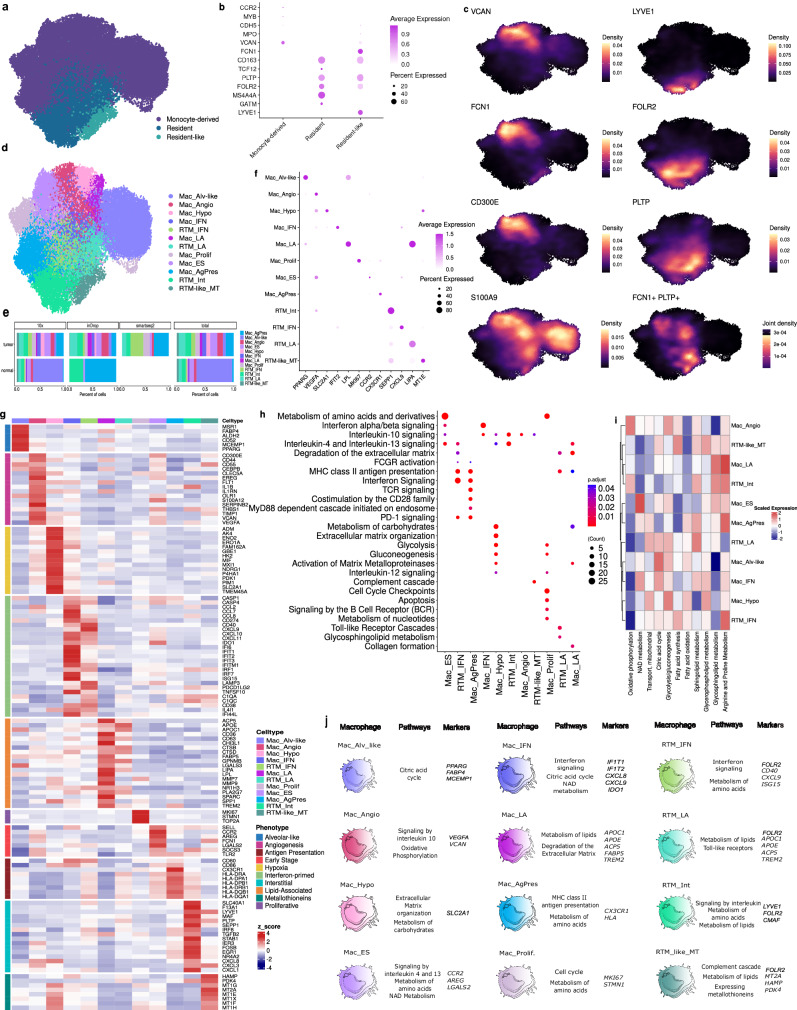
Characterization of macrophage subpopulations. **a** UMAP of macrophages color-coded by the ontogeny subdivision. **b** Dot plot showing the mean expression of the resident and monocyte-derived macrophage-related markers. The dot size indicates the percent of expressing cells, and the dot color is the scaled average expression. **c** Density plots highlighting the gene expression and co-expression of each cell. **d** UMAP of macrophages colored by the twelve states identified. **e** Bar plot showing the distribution of Mac subpopulations across sample types by three different sequencing platforms (10x, inDrop, Smart-seq2), with an aggregated total. **f** Dot plot showing the mean expression of genes related to the Mac subpopulation. Dot size indicates the percent of expressing cells, and the dot color the scaled average expression. **g** Heatmap showing the gene signature per subpopulation. The color scale represents the scaled expression of each gene. **h** Dot plot representing the functional enrichment analysis of Mac subpopulations using Reactome database. The size of each circle represents the number of genes and such circles are colored by p-adjust using one-sided Fisher’s exact test with BH multiple-testing correction. **i** Heatmap showing main metabolism signature for each Mac states. The color scale represents the scaled expression of each pathway. **j** Schematic overview of the diverse phenotypes and functional signatures of Mac subpopulations characterized in this study. Source data are provided as a Source Data file.

Macrophages were the largest group of mononuclear phagocytes in this study, corresponding to 51.6% of all myeloid cells (Fig. 1f). We identified three main clusters of macrophages based on their ontogeny markers^25^: 1) monocyte-derived macrophages distinguished by the expression of monocyte markers such as *CCR2* (an embryonic cell marker indicative of monocyte lineage), *VCAN*, *S100A6*, and *CD52*; 2) likely resident-tissue macrophages (RTM), expressing *CD163* (highly expressed in the initial phase (first wave) of embryonic hematopoiesis, along with markers associated with the second wave of macrophage generation, such as MPO, and other tissue residency markers such as *TCF12*, *MS4A4A*, *GATM*, *LYVE1*, *FOLR2*, and *PLTP*; and 3) resident-like macrophages (RTM-like), resembling resident macrophages, they comprise of monocyte-derived macrophages that exhibit a combination of markers from both the aforementioned groups (Fig. 4a–c). Although monocyte-derived macrophages were the most common type in tumors, RTMs were surprisingly shown to be expanded in tumors (Supplementary Fig. 5a, b), particularly in lung samples (Supplementary Fig. 5c-f). RTM-like cells were found in all conditions except uveal melanoma, melanoma, liver tumors, and normal breast tissue (Supplementary Fig. 5c–f). Also, we observed an RTM-like enrichment in ovarian samples predominantly in normal tissue compared to tumors (Supplementary Fig. 5e).

In addition to classifying macrophages based on origin, we also distinguished 12 clusters based on gene programs (Fig. 4d–g), across the technologies (Supplementary Fig. 5g-i) with greater diversity in tumors than normal samples (Fig. 4e and Supplementary Fig. 5d).

To ascertain the optimal method for mitigating batch effects while conserving the biological variation inherent to distinct cell types, we conducted an extensive analysis of six data integration methods on the macrophage data (Supplementary Fig. 6a), employing a benchmarking framework^26^. This framework assesses the quality of integration, the retention of cell type-specific variation, and the preservation of biological meaning within the integrated data. When considering the metrics laid out by this framework, Harmony^27^ excelled in batch correction but fell short in maintaining cell identity. In contrast, both scVI^28^ and scANVI^29^ achieved high rankings in conserving cellular biology. Among these, scANVI marginally outperformed scVI, benefiting from the use of pre-existing cell type annotations to refine cell type distinctions post-integration. Despite this, scVI was chosen for further analyses due to its robust capability to process unlabeled data, striking a balance between effective integration and biological fidelity (Supplementary Fig. 6b). Employing scVI, we generated an embedding that accurately integrates and represents all 37,891 macrophage cells from 11 studies across our datasets (Supplementary Fig. 6c).

The macrophages subpopulations were stratified according to their functional gene signatures, mostly following a consensus model for TAM diversity^9^. The alveolar-like macrophage (Mac_Alv-like, n = 8816), marked by the expression of *PPARG* and *MCEMP1*, was mostly found in lung samples, as expected (Fig. 4e and Supplementary Fig. 5d-f). The pro-angiogenic macrophage subpopulation (Mac_Angio, n = 3108) displayed high expression of genes associated with angiogenesis, such as *VEGFA*, *VCAN*, and *EREG*, and was found enriched in lung and ovarian tumors (Supplementary Fig. 5d–f). Hypoxia-associated macrophages (Mac_Hypo, n = 1802) were mostly found in tumor samples (lung and ovary) (Supplementary Fig. 5d–f) and presented an enrichment of genes associated with hypoxia, such as *SLC2A1* and *ERO1* (Fig. 4f, g).

Two macrophage subpopulations expressing high levels of interferon-primed genes, such as *CCL8, IDO1*, and *CXCL9*, were named RTM_IFN (n = 2061) and Mac_IFN (n = 1981), which were abundant in ovarian and lung tumor samples, respectively (Fig. 4d–g and Supplementary Fig. 5d–f). Another two macrophage clusters with a lipid-associated (LA) metabolism transcriptional signature (Fig. 4d–g), including genes such as *FABP5*, *LPL*, and *LIPA*, named RTM_LA (n = 3569) and Mac_LA (n = 1249), were found enriched in breast, lung, and colorectal tumors (Supplementary Fig. 5d–f). Another subpopulation expressing high levels of *MKI67* and *STMN1* was classified as Mac_Prolif (n = 2234), and found to be enriched in liver, melanoma, and ovary tumor samples (Fig. 4d-g, Supplementary Fig. 5e,f). We also identified a large population of macrophages in uveal melanoma metastatic samples (Supplementary Fig. 5e), which expressed high levels of *CCR2* (Fig. 4d, f), a receptor for monocyte chemoattractant protein-1, and *LGALS2*. Therefore, this state was named early-stage macrophage (Mac_ES n = 4073).

We identified one monocyte-derived macrophage subpopulation displaying a pro-inflammatory phenotype, expressing the receptor of the chemokine CX3CL1 (*CX3CR1)* and genes from the antigen-presentation pathway (*HLA-A/C*, *HLA-DQA1/B1*). This population was named Mac_AgPres (n = 3965) (Fig. 4d–g). Additionally, a population expressing high levels of resident markers such as *LYVE1, FOLR2*, and *PLTP*, which resembled interstitial macrophages with specific expressions of genes such as *MAF* and *SEPP1*, was identified. This subpopulation was named RTM_Int (n = 3507) and was found to be enriched in normal tissues (Fig. 4e, Supplementary Fig. 5d). The RTM-like macrophage subpopulation was found to express high levels of metallothioneins (Fig. 4d–g), named RTM-like_MT (n = 1209), and was found predominantly in ovary samples (Supplementary Fig. 5e,f). Importantly, the signatures identified were reproducible among different platforms (Supplementary Fig. 7a-b).

By examining the enriched pathways in each macrophage subpopulation, we were able to identify distinct functional roles. Enriched pathways in Mac_IFN, Mac_ES, RTM_IFN, and Mac_AgPres subpopulations were linked to the interferon signaling pathway. Antigen presentation genes were enriched in RTM_IFN, RTM_LA, Mac_LA, and Mac_AgPres, with the latter also expressing genes related to phagocytosis, such as the MyD88-dependent cascade initiated on endosomes and FCGR activation (Fig. 4h). Interestingly, Mac_ES, Mac_IFN, Mac_Hypo, RTM_Int, Mac_Angio, RTM-like_MT, and RTM_IFN showed enrichment of genes involved in the IL-10, −4, or 13 pathways, which have been associated with pro-tumoral processes (Fig. 4h). Mac_Hypo, RTM_LA, Mac_LA, and RTM_IFN displayed pathways related to extracellular matrix remodeling (Fig. 4h and Supplementary Data 6).

Macrophages have the ability to utilize diverse energy sources to fulfill their functions (Fig. 4i). Certain subpopulations, namely Mac_Hypo and Mac_Angio, appear to rely on glucose metabolism since genes related to the glycolytic pathway are enriched. Among these, Mac_Angio also exhibits enrichment in genes involved in the oxidative phosphorylation pathway. Clusters such as the Mac_LA, RTM_LA, and RTM-like_MT expressed more genes associated with fatty acid metabolism. Notably, NAD metabolism is highly enriched in the Mac_ES and Mac_IFN. The Mac_LA, RTM_IFN, and Mac_AgPres expressed an enrichment in genes related to amino acid metabolism. The distinct metabolic profiles of RTM subpopulations appeared to vary significantly from one another. For a comprehensive overview of the major metabolic pathways, please refer to Supplementary Fig. 8.

Our analysis has refined the understanding of macrophage heterogeneity by distinguishing between profiles that were previously grouped together. For instance, we have identified four unique macrophage clusters (Mac_LA, Mac_AgPres, RTM_LA, and Mac_IFN) that express high levels of *TREM2*. Unlike earlier classifications that lumped these as generic *TREM2*^*+*^ macrophage subpopulation (see Supplementary Fig. 9a–h), the subpopulations presented distinct signatures and functional profiles. Similarly, our study challenges the traditional view of RTMs as a uniform group. We have identified at least three diverse RTM profiles, including interstitial, lipid-associated metabolism, and interferon-primed macrophages, each characterized by different tumorigenic behaviors. By assessing cluster purity, we found that our technique produced highly homogeneous clusters compared to previous integration approaches, indicating its effectiveness (Supplementary Fig. 9i–k and Supplementary Data 7). Overall, our approach expanded the catalog of mononuclear phagocytes in the TME, enabling the identification of rare populations. This led to valuable insights into the diversity and complexity of mononuclear phagocyte populations in the TME, especially macrophages (Fig. 4i), underscoring the importance of employing deep scRNA-Seq analysis to uncover poorly studied subpopulations.

In order to validate our findings, we extended our analysis to include 13 independent datasets, creating an additional pan-cancer atlas. This expanded atlas encompasses a comprehensive array of samples (n = 233) across 10 tumor types, incorporating a significant number of myeloid cells (n = 96,514) (Supplementary Fig. 10a–d and Supplementary Data 7). Within this broader dataset, we validated the presence of several DC subpopulations, including the DC_CXCL8 phenotype. The DC4 subpopulation was the only exception, probably because this version of the atlas does not include blood samples (Supplementary Fig. 10e, f). For monocytes, we successfully identified all six subpopulations as initially characterized (Supplementary Fig. 10g, h). In the case of macrophages, we confirmed nine out of ten signatures (Supplementary Fig. 10i–k). The one exception, the RTM-like_MT subpopulation, was noted for its low expression of the metallothionein gene set, indicating a distinct transcriptional profile. Of note, Mac_Alv-like signature was not so evident. This may be attributed to the lack of lung samples within our new integrated dataset. This cross-checking of our results with independent datasets strengthens the trustworthiness of our identified mononuclear phagocyte groups.

### A plethora of macrophage phenotypes and functional states co-exist in the TME

**Fig. 5:**
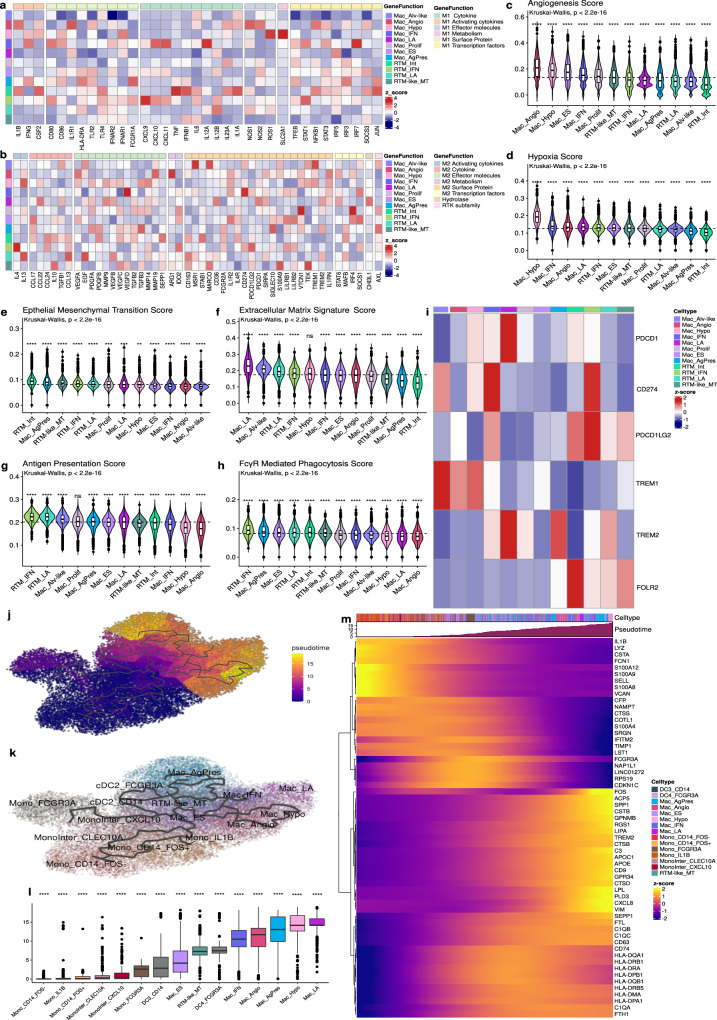
Macrophages exhibited diverse phenotypes and functional states. Heatmap showing the distribution and score signature of (**a**) M1 and (**b**) M2 markers across the Mac subpopulations. The color scale represents the row-wise scaled expression of each gene. Violin plot showing the gene signature of five hallmarks of cancer: **c** angiogenesis, **d** hypoxia, **e** EMT, **f** ECM, **g** antigen presentation, and **h** phagocytosis. Dashed lines represent the average score. **i** Heatmap of immunosuppressive gene signature. Pseudotime analysis of monocytes, macrophages, and dendritic cells derived from monocytes. UMAP color-coded by the (**j**) pseudotime and by the (**k**) subpopulations. **l** Boxplot of the pseudotime across the subpopulations arranged in ascending order. **m** Heatmap showing expression variation of the indicated transcripts (only genes with *q* value = 0 and morans_I > 0.25, are depicted). Box indicates the range from 25th to 75th percentile, with whiskers extending to 1.5 times the interquartile range. Outliers are plotted separately, center indicates the median value. For statistical significance, we performed the Kruskall–Wallis test (p <2.2×10^−16^) followed by Wilcoxon to compare each group against “all” (i.e., base-mean). Ns non-significant.; **p < 0.01; ***p < 0.001; ****p < 0.0001. Source data are provided as a Source Data file.

Since M1 and M2 polarization gene programs are well-known^30–32^ and reflect important macrophage-related features, we attempted to assign these profiles to the 12 macrophage subpopulations identified (Fig. 5a, b). Although the RTM-IFN and Mac_ES were, respectively, the subpopulations displaying the higher scores for M1 and M2 gene signatures (Supplementary 11a, b and Supplementary Data 8), there was no clear overrepresentation of genes associated with either polarization profile in any of the subpopulations. In effect, subpopulations such as the RTM-IFN express high levels of genes encoding pro-inflammatory cytokines such as *CXCL9*, *CXCL10*, and *CXCL11*, as well as immunosuppressive molecules such as PDL-1 and PDL-2 (Fig. 5a, b). Although M1/M2 ratio scores (Supplementary Fig. 11c) discriminate the subpopulations regarding their polarization states, this dichotomy oversimplifies the highly specialized, transcriptomically dynamic, and extremely heterogeneous nature of macrophages in vivo.

To gain further insights into the roles of macrophage subpopulations in the TME, we investigated their associations with cancer hallmark signatures^33^ (Supplementary Data 4). Consistent with their names, Mac_Angio exhibited higher scores for the Angiogenesis signature, while Mac_Hypo showed elevated scores for the Hypoxia signature (Fig. 5c, d). Notably, RTM_Int demonstrated enrichment in signatures associated with the induction of epithelial-mesenchymal transition (Fig. 5e). The extracellular matrix signature in macrophages^34^ was predominantly observed in Mac_LA, Mac_Alv-like, and RTM_LA subpopulations (Fig. 5f and Supplementary Data 4). The RTM_IFN cluster exhibited the highest scores for antigen presentation^33^ as well as for FcγR-mediated phagocytosis signatures (Fig. 5g, h).

To explore the potential immunosuppressive role of macrophages, we examined the co-expression of checkpoint genes and immune regulators. We found that *TREM2*, a marker for immunosuppressive TAMs^35^, was highly expressed in both resident and recruited LA populations, as well as in Mac_AgPres (Fig. 5i). However, only Mac_LA exhibited high levels of co-expression with the *PDCD1* gene (programmed death 1, PD-1). Furthermore, Interferon-primed subpopulations demonstrated elevated expression of *CD274* and *PDCD1LG2*, the genes encoding PD-L1 and PD-L2, respectively (Fig. 5i). RTM_Int cells expressed high levels of both *FOLR2* and PD-L2 coding-gene. *TREM1*, typically highly expressed in macrophages in inflamed tissues^36,37^, was mainly expressed by the Mac_Alv-like, Mac_Angio, and Mac_Hypo subpopulations (Fig. 5i). We also evaluated the myeloid-derived suppressor cell (MDSCs) signature proposed by Alshetaiwi et al (2020) in all immature MDSCs. However, none of the subpopulations resembled that particular signature (Supplementary Fig. 12). Nevertheless, monocytes subpopulations such as Mono_CD14_FOS^-^ and Mono_CD14_FOS^+^ exhibited likely suppressive profiles, based on *CTSD* and *PLA2G7* expression, as well as neutrophils states/subpopulations, based on *CD84, AGR2* and *CLE4E* expressions (Supplementary Fig. 12).

To elucidate the intricate differentiation trajectory in the monocyte-macrophage and monocyte-DC axes, we employed an unsupervised pseudotime analysis strategy. By using Mono_CD14_FOS^-^ as the “root” for trajectory inference, our analysis supported the notion that CD14^hi^ monocytes serve as precursor cells for both Mono_FCG3RA and monocyte-derived cells in tissues (Fig. 5j–m). Intermediate monocytes (MonoInter_CXCL10 and MonoInter_CLEC10A) can potentially give rise to both DCs and Mac_ES subpopulations^38^. The Mac_ES subpopulation appears to be the earliest monocyte-derived macrophage subpopulation ontologically, acting as a crucial precursor for other macrophages, including RTM-like. Two distinct phenotypes, originating from two main branches, were identified as the most distant from the root: 1) Mac_AgPres and 2) Mac_LA, both of which are characterized by high levels of *TREM2* expression. Through the analysis of the genes that co-vary over pseudo-time, we were able to identify a set of monocyte-related genes (*VCAN, SELL*, and *FCN1*) at the beginning of the trajectory and both specific and general phenotype markers of the macrophage lineages (*APOE*, *TREM2*, *SPP1*, *HLA-DRA*, *C1QA*, and *CD63*) in late branches (Fig. 5k–m).

### Deciphering the clinical impact of TREM2^+^ macrophages in the TME

The clinical significance of MDC subpopulations as potential targets has garnered increased interest due to their higher abundance in the TME. To further explore their clinical impact, we conducted an investigation using larger cohorts to assess MDC subpopulations in different tumor types. We utilized a set of 47 subpopulation signatures to estimate the proportions of cell types through deconvolution analysis of bulk RNA-Seq samples obtained from nine tumor types available in The Cancer Genome Atlas (TCGA) database. To validate the applicability of our signature matrix in estimating cell populations within the TCGA data, we calculated the correlation between bulk RNA-Seq and scRNA-Seq data. We observed a significant correlation for all TCGA cohorts (Pearson *R*^2^ > 0.7, *p* value ≤ 0.05) (Supplementary Fig. 13a–i).

**Fig. 6:**
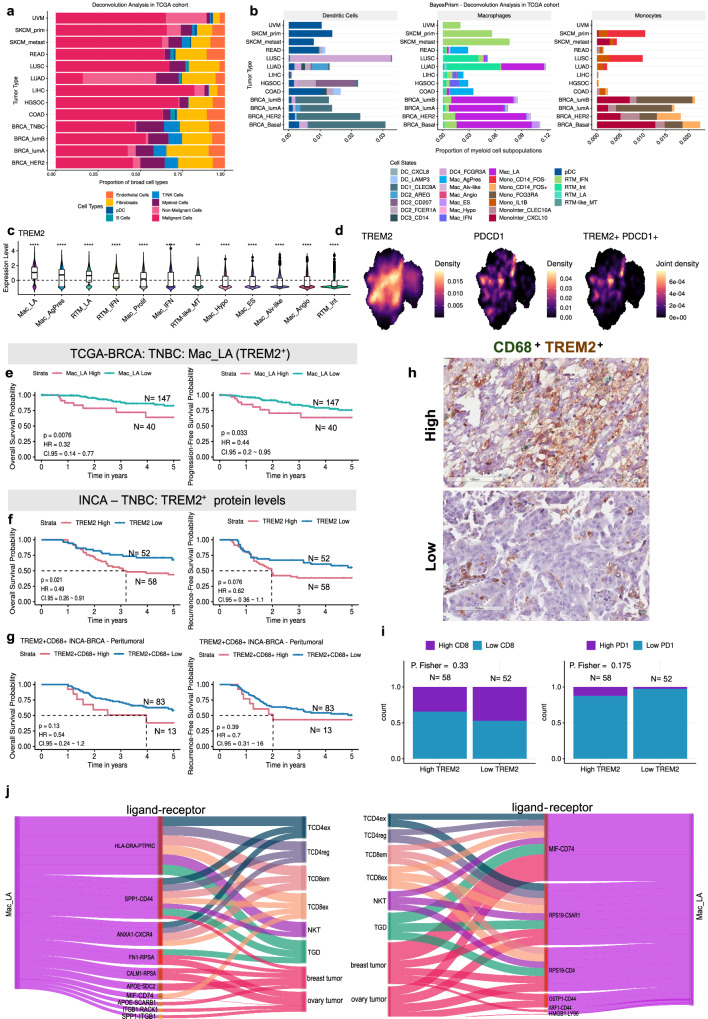
Impact of Mac_LA (TREM2^+^) on clinical outcomes. Bar plot showing the distribution of (**a**) broad cell types and (**b**) mononuclear phagocytes estimated through deconvolution across different tumor types (data sourced from TCGA database). **c** Violin plot of *TREM2* expression across macrophage populations. Dashed lines represent the average score. Box represents the 25th to 75th percentile range, with whiskers extending to 1.5 times the interquartile range. Outliers are plotted separately, and the median is marked in the center. Statistical significance was tested using the Kruskall–Wallis test followed by Wilcoxon tests, with significance levels indicated as **p < 0.01 and ****p < 0.0001. **d** Density plots of PD1 encoding gene and *TREM2* expression and co-expression in macrophages. **e** Overall Survival (OS) and Progression-Free Survival (PFS) associated with Mac_LA estimated through deconvolution in TNBC (TCGA-BRCA cohort, N = 187). HIGH and LOW groups cutoff were determined using the surv_cutpoint R function. OS and Recurrence-Free Survival (RFS) analysis for (**f**) TREM2^+^ and (**g**) CD68^+^TREM2^+^ co-staining markers in the TNBC-INCA cohort (N = 110 and N = 96, respectively). For TREM2 alone, staining was measured through ImageJ software and HIGH and LOW group cutoffs were calculated using the surv_cutpoint R function. For co-staining, levels of staining and groups were determined on the percentage of marked cells by a pathologist. For all Kaplan-Meier curves, the log-rank test was applied, and *p* values ≤ 0.05 were considered significant. **h** IHC representative of CD68^+^ TREM2^+^ expression in TNBC-INCA cohort. Image obtained by Aperio ImageScope v12.4.6.5003. **i** Proportions of CD8^+^ and PD1^+^ markers in the TREM2^+^ HIGH and LOW groups in TNBC-INCA. **j** Sankey diagram representing the putative cell-cell interactions between Mac_LA and other cells. Ligand-receptor pairs are represented in the middle. Line thickness represents ligand and receptor expression-based z-scores. Source data are provided as a Source Data file. INCA Brazilian National Cancer Institute, TCGA The Cancer Genome Atlas, TNBC Triple Negative Breast Cancer, HGSOC High-Grade Serous Ovarian Carcinoma, BRCA Breast Carcinoma, COAD Colon Adenocarcinoma, READ Rectum Adenocarcinoma, LIHC Liver Hepatocellular Carcinoma, SKCM Skin Cutaneous Melanoma, LUAD Lung Adenocarcinoma, LUSC Lung Squamous Cell Carcinoma, UVM Uveal Melanoma.

All the tumor samples were enriched with malignant cells (~50%), except for lung adenocarcinoma (LUAD), which had very similar proportions of malignant cells (16% of total cells) and fibroblasts (19.9% of total cells) and higher content of epithelial cells (43.4% of total cells) (Fig. 6a). The proportions of cell types across tumors are shown in Supplementary Fig. 14a–m. Breast cancer (BRCA) presented a higher proportion of non-epithelial cells, and together with LUAD, these tumors were the most enriched in MDCs (Fig. 6a). Stratifying for mononuclear phagocyte subpopulations, macrophages were among the most abundant cell types (~12% of total) (Fig. 6b). The RTM_IFN subpopulation was more commonly found in different tumor types, especially in skin cutaneous melanoma (SKCM) (7.6% for metastatic and 5.6% for primary tumor) and uveal melanoma (UVM) samples (1.9% of total cells). In contrast, RTM_Int was predominantly found in lung tumors (6.06% in LUAD and 3.9% in lung squamous cell carcinoma) (Fig. 6b). The Mac_LA subpopulation was mainly found in LUAD and BRCA tumors, while Mac_AgPres was mainly found in colorectal tumors (2.6% of total cells) (Fig. 6b).

We then conducted an investigation to assess the impact of *TREM2*^*+*^ macrophage subpopulations (Fig. 6c), particularly Mac_LA, due to its high abundance in tumors (Fig. 6d). Patients with triple-negative breast cancer (TNBC) were categorized into groups based on high and low estimated abundance of Mac_LA in their TME. Those with a high quantity of Mac_LA demonstrated significantly worse overall survival (OS) and progression-free survival (PFS) compared to those with low levels of Mac_LA (OS, log-rank test, p-value = 0.0076; PFS, log-rank test, *p* value = 0.033) (Fig. 6e). However, this trend was divergent in other BRCA subtypes (Supplementary Fig. 15a–c) as well as high-grade serous ovarian carcinoma (HGSOC) samples (Supplementary Fig. 16a, b). We also evaluated global *TREM2* expression and protein levels as clinical biomarkers in different cohorts. Global *TREM2* gene expression was associated with poorer OS compared to the lower expression group in patients diagnosed with different BRCA subtypes (TNBC, log-rank test, p-value = 0.0031, Supplementary Fig. 15d-g) and HGSOC (OS, log-rank test, p-value = 0.013, Supplementary Fig. 16c, d). Upon evaluating TREM2 protein expression globally and specifically within peritumoral macrophages (identified by CD68^+^ and TREM2^+^ co-staining) via immunohistochemistry, we observed that high levels of lipid-associated macrophages (Mac_LA) tend to correlate with poorer clinical outcomes in TNBC. This observation aligns with our survival analysis conducted using deconvolution estimates. However, it was only the overall TREM2 expression levels in the TNBC-INCA cohort that were significantly associated with adverse outcomes (Fig. 6f-h). No significant difference was found in the HGSOC-INCA cohort (Supplementary Fig. 16e, f).

In HGSOC-INCA, the higher intratumoral expression of CD68^+^ and TREM2^+^ was accompanied by increased PD-1 and CD8 levels, suggesting the involvement of the PD-1 receptor in the clinical response associated with TREM2-expressing macrophages (Supplementary Fig. 16g-h). In TNBC-INCA, a divergent pattern was noted. The High TREM2 group exhibited a lower proportion of CD8, suggesting an inverse relationship compared to HGSOC-INCA (Fig. 6i). These distinct predictions underscore the context-dependent variations in the immune microenvironment and the nuanced role of TREM2-expressing macrophages in different cancer types. PD-1 expression was significantly associated with poorer OS and recurrence-free survival (RFS) in TNBC-INCA patients (OS, log-rank test, p-value = 0.0053; RFS, log-rank test, p-value = 0.03) (Supplementary Fig. 17a). No difference in OS and RFS were observed for CD8 and PD-L1 (Supplementary Fig. 17b, c).

To identify communication axes involving Mac_LA, we conducted a ligand-receptor inference analysis between these cells and other key players in tumor progression, including malignant cells and T/NK cells. We first clustered and annotated these cells based on canonical gene markers and DEGs, resulting in 11 cell types (Supplementary Fig. 18a, b). Mac_LA exhibited 714 putative intercellular communications, as indicated by ligand-receptor pairings between Mac_LA and lymphocytes, as well as malignant cells (Supplementary Data 9). Among these interactions, CD8^+^ T cells emerged as the most representative cell type (Supplementary Fig. 18c). The axes of major expression in these interactions were SPP1_CD44, MIF_CD74, ANXA1_CXCR4 and GSTP1_CD44 (Fig. 6i). These findings shed light on potential communication pathways involving Mac_LA and provide valuable insights into its role in tumor microenvironment interactions.

### MDC subpopulations display distinct clinical outcomes depending on their niche

To further investigate the clinical impact of other MDC subpopulations inferred in the TME across different tumor types, we conducted a univariate Cox regression analysis (Supplementary Fig. 19a). Mono_FCG3RA subpopulation, enriched in BRCA tumors, especially the Luminal B subtype (representing 1.22% of total cells) (Fig. 6b), exhibited a significant association with poor OS in TNBC when assessed through uni- and multivariate regression analysis (log-rank p-value = 0.0027, Supplementary Fig. 19a–c), reinforcing its independent value as a prognostic marker. Conversely, in patients with the Luminal A subtype, a higher percentage of this population was correlated with improved OS (log-rank p-value = 0.0011, Supplementary Fig. 19a,c,e), indicating that its clinical impact depends on the specific TME context. Furthermore, Mono_FCG3RA was markedly reduced in TNBC patients with “tumor-free” status (p-value = 0.0095), whereas this was not observed in the Luminal A subtype (Supplementary Fig. 19f).

**Fig. 7:**
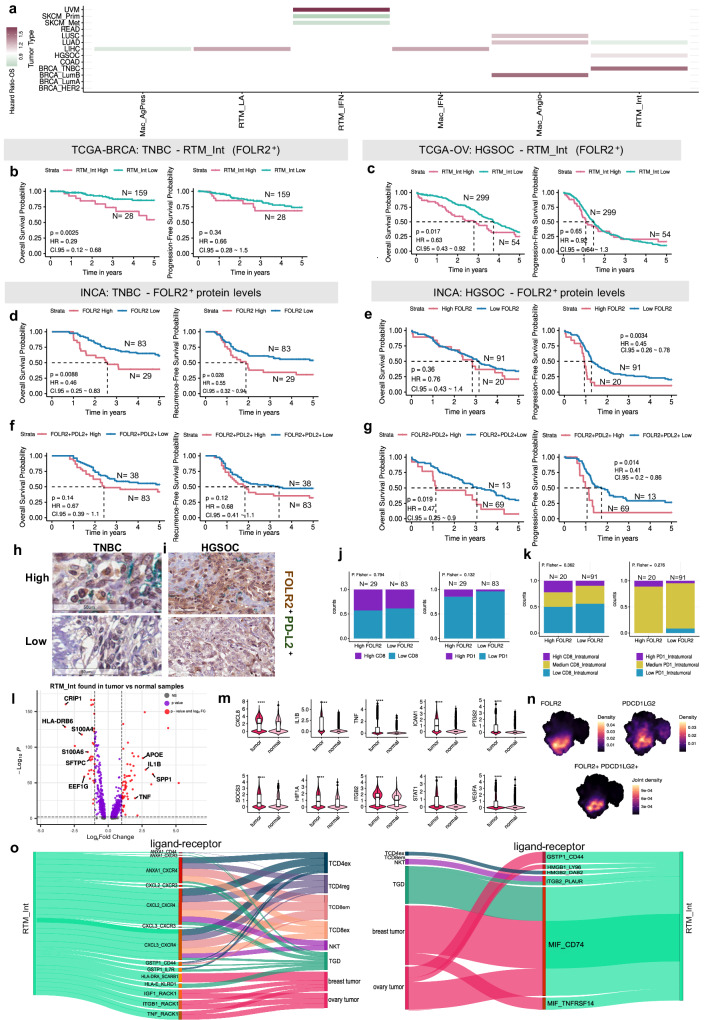
Impact of RTM_Int (FOLR2^+^) on clinical outcomes. **a** Cox univariate analysis (log-rank, p-value <0.05) in macrophage subpopulations. OS and PFS for (**b**) TNBC (N = 187) and (**c**) HGSOC (N = 353), associated with RTM_Int estimated through deconvolution in bulk RNA-Seq samples from TCGA cohort. HIGH and LOW groups cutoff were determined using surv_cutpoint. OS and PFS considering FOLR2 levels for (**d**) TNBC-INCA (N = 112) and (**e**) HGSOC-INCA (N = 111) cohorts. For TNBC-INCA, IHC FOLR2 levels were measured with ImageJ software and groups cutoff were determined using the surv_cutpoint. For HGSOC-INCA, levels of staining and groups were determined by the percentage of marked cells by a pathologist. OS and PFS considering FOLR2^+^ PDL-2^+^ co-staining for (**f**) TNBC-INCA (N = 121) and (**g**) HGSOC-INCA (N = 82) cohorts, measured by HALO software version 3.6. Group cutoffs were calculated using surv_cutpoint. For all Kaplan-Meier curves, the log-rank test was applied, and p-values ≤0.05 were considered significant. IHC representative of FOLR2^+^ PDL-2^+^ co-satining in (**h**) TNBC-INCA and (**i**) HGSOC-INCA cohorts. Image obtained by Aperio ImageScope v12.4.6.5003. Proportions of CD8^+^ and PD1^+^ markers in the FOLR2^+^ HIGH and LOW groups in the (**j**) TNBC-INCA and (**k**) HGSOC-INCA cohorts. **l** Volcano Plot representing DEGs between RTM-Ints found in tumor vs normal samples (Wilcoxon rank sum, FDR adjusted p-value < 0.05, and cutoff |Log2FC| > = 1). **m** Violin plot of DEGs belonging to IL4 and IL13 signaling from the Reactome database. Box indicates 25th to 75th percentile range, with whiskers extending to 1.5 times the interquartile range. Outliers are plotted separately, center indicates median value. Groups were compared using Wilcoxon rank sum test (****p < 0.0001). **n** Density plots highlighting *FOLR2* and *PDCD1* co-expression in macrophage populations. **o** Sankey diagram representing putative cell-cell interactions between RTM_Int and other cells. Ligand-receptor pairs are represented in the middle. Line thickness represents ligand and receptor expression-based z-score. Source data are provided as a Source Data file. TNBC Triple Negative Breast Cancer, INCA Brazilian National Cancer Institute, TCGA The Cancer Genome Atlas, BRCA Breast Cancer, HGSOC High-Grade Serous Ovarian Carcinoma.

Another subpopulation with distinct impacts depending on the TME niche is the RTM_Int. In HGSOC and TNBC, RTM_Int is associated with poor OS, while in lung adenocarcinoma (LUAD) it is linked to improved OS (univariate Cox regression and log-rank, p-value <0.05, Fig. 7a and Supplementary Fig. 19a). No significant clinical impact was observed in other BRCA subtypes (Supplementary Fig. 20a–c). Patients with TNBC and HGSOC were categorized into groups, and those with a high quantity of RTM_Int demonstrated significantly worse OS compared to those with low levels of RTM_Int (log-rank test, p-value = 0.0025, and log-rank test, p-value = 0.017, respectively), but there was no significant difference for progression-free survival (PFS) (log-rank test, p-value = 0.34, and log-rank test, p-value = 0.65, respectively) (Fig. 7b, c). When analyzing FOLR2 as a biomarker, high protein levels were associated with poor OS in TNBC-INCA (log-rank test, p-value = 0.0088, Fig.7d) and poor PFS in HGSOC-INCA (log-rank test, p-value = 0.0034, Fig. 7e). The global expression of *FOLR2* showed distinct results depending on the analyzed cohort (Supplementary Fig. 21a-c). In order to validate the impact of RTM_Int we performed dual staining with FOLR2 and PDL-2, another marker highly present in this macrophage subpopulation. For TNBC-INCA the dual staining showed no significant differences, although the same pattern was observed (Fig. 7f). In HGSOC-INCA the high levels of FOLR2 and PDL-2 were associated with a poor OS (log-rank, p-value = 0.019) and PFS (log-rank, p-value = 0.014), reinforcing the previous findings (Fig. 7g). Representative images of tissue microarrays and the association between FOLR2 and CD8 or PD-1 are provided in Fig. 7h–i.

Due to its dual role in prognosis associated with niche variability, we compared DEGs between RTM_Int cells found in normal and tumor samples. Protumorigenic factors associated with hypoxia and angiogenesis, like *HIF1A* and *VEGFA*, as well as genes related to a pro-tumoral phenotype, such as *SPP1* and *TGFB2*, were highly expressed in this macrophage found in the tumor context (Fig. 7l, m). Since RTM_Int exhibits high levels of *FOLR2* and the immunosuppressive gene *PDCD1LG2* (Fig. 7n), we also investigated the association with other immune markers. Interestingly, an increase in CD8^+^ T cell frequency was observed in tumor samples displaying high global levels of FOLR2 (Fig. 7j) and intratumoral FOLR2 macrophages (Fig. 7k) accompanied by higher levels of PD-1^+^. No significant difference in OS and PFS was observed for PD-1^+^ and CD8^+^ cells, or CD68^+^ macrophages (Supplementary Fig. 22a–h). These findings support the notion that FOLR2^+^ macrophages are reprogrammed during tumor formation by activating immunosuppressive gene programs, potentially leading to the exhaustion of CD8^+^ T lymphocytes in the context of TNBC and ovarian tumors. This highlights RTM_Int as a promising therapeutic target. Analyses of OS (uni and multivariate) from other tumor types are described in Supplementary Data 10 and 11. Representative images of tissue microarrays depicting CD8^+,^ PD1^+^, and KI67 markers and FOLR2^+^ PDL-2^+^ co-expression in both HGSOC-INCA and TNBC-INCA samples are provided in Supplementary Fig. 22i and Supplementary Fig. 23a, b.

In our study, we further explored the clinical impact of RTM_Int by investigating the communication axes between this subpopulation and malignant cells, as well as T/NK cells. We identified 889 intercellular communications involving the RTM_Int cell type (Supplementary Fig. 18c, Supplementary Data 9). The connections with CD8^+^ T and CD4^+^ T cells were particularly enriched, with ANXA1_CXCR4, CXCL2_CXCR4, and CXCL3_CXCR4 axes being prevalent in these communications (Fig. 7o). Additionally, similar to Mac_LA, MIF_CD74 appeared to be an important axis ligand involved in cell-cell communication between RTM_Int and tumor cells (Fig. 7o).

In comparison to previous studies, our method of integrating scRNA-Seq datasets, clustering, and characterizing cell states/subpopulations significantly expanded the catalog of mononuclear phagocytes in the TME. This approach allowed us to identify rare and poorly described subpopulations. Furthermore, our analysis led to the identification of distinct RTM phenotypes, such as interstitial, lipid-associated, and interferon-primed macrophages, as well as to the characterization of a phenotype (RTM-like_MT) where cells simultaneously express resident-tissue and monocyte-derived markers. Moreover, we unveiled the clinical impact of specific TREM2^+^ (Mac_LA) and FOLR2^+^ (RTM_Int) subpopulations in both TNBC and HGSOC patients, which may lead to new possibilities for targeted treatments.

## Discussion

Integration of single-cell data has proven to be a game changer for uncovering valuable biological information from large and complex volumes of data. By pooling data from diverse sources, it is possible to unveil cellular heterogeneity and phenotypes by enhancing the robustness and reliability of the findings. Here we have thoroughly integrated and characterized cell subpopulations in the TME, yielding a large and comprehensive dataset comprising different tumors, technologies, and sample types, generated across multiple conditions and donors. This diversity introduces dataset-specific batch effects. The selection of scVI as the integration tool was based on comprehensive analyses, which included the application of various integration platforms and the evaluation of metrics from recent benchmarking integration studies^26,39,40^. These studies highlight the importance of selecting an appropriate data integration method to ensure the reliability of the resulting atlas. scVI^28^, employing variational autoencoders, is recognized for its capability to process complex datasets by identifying latent variables and conserving biological variance. Nonetheless, scVI may not completely eliminate batch effects or may inadequately delineate batches within certain cell types, reflecting a trade-off between batch effect correction and the preservation of biological diversity. To bolster the robustness and generalizability of our findings, cross-validation with alternative integration methods was performed, and results were corroborated using independent datasets.

Notably, we expanded the classification of myeloid-derived cell types including monocytes, macrophages, DCs, neutrophils, and mast cells to 29 distinct clusters. We endeavored to infer the likely origins of macrophages within our study, differentiating between those that are suggestive of tissue residency and those potentially derived from monocytes while acknowledging the inherent challenges in classifying macrophage ontogeny in human samples. This distinction was drawn using the best available evidence and methodologies, including analysis of specific markers as guided by the current literature^25,41^. However, we recognize the limitations of these approaches, particularly the absence of ways to unequivocally trace cell lineages in human studies. Through a rigorous methodology and extensive curation, we identified more homogeneous clusters compared to previous approaches^20,21^, unraveling high-quality clusters representing a wide range of cells found in the TME of solid tumors, including states less characterized such as Mono_CD14_FOS^+/−^ and RTM-like_MT. These findings offer valuable insights for future research and development of immune checkpoint blockades and target therapies.

Mononuclear phagocytes play a pivotal role in development and homeostasis maintenance^42^. Their remarkable plasticity enables them to actively participate in various critical processes, including organogenesis, tissue repair, and immune defense. In addition to producing essential growth and inflammatory factors that orchestrate lymphocyte responses, they are involved in extracellular matrix maintenance, angiogenesis, tissue enervation, and the clearance of apoptotic cells^43,44^. In the context of tumor development, which shares similarities with organ development, these functions are also highly prevalent. In lung metastasis, mononuclear phagocytes account for more than 60% of the TME cell population, underscoring their significant involvement in the tumor microenvironment and potentially impacting disease progression.

In our study, we identified six distinct states of DCs based on their unique gene signatures. Among them, one cDC type 1, expressing *CLEC9A* and *CADM1*, four subpopulations of cDC type 2, three of them DC2, distinguished by *CD1C* expression, and one DC3, one DC4, one mature subpopulation expressing *LAMP3* and *CCR7* and a poorly described phenotype, DC_CXCL8, that exhibits contrasting prognostic implications in different tumor types. Moreover, we identified six monocytes displaying either pro- or anti-tumoral properties, regulating a variety of processes from angiogenesis to immune modulation in a context-dependent manner. The transcription factor c-Fos has been shown to positively regulate monocyte development^45,46^. Resting monocytes typically have low or undetectable *FOS* mRNA levels, and their activation is associated with a rapid and transient increase in *FOS* mRNA levels^45^. Furthermore, *FOS* overexpression stimulates growth arrest and differentiation into macrophages^45,47^. In addition, the Mono_FCGR3A is associated with a worse prognosis in the TNBC subtype, aligning with the findings of Chen et al., who have linked non-classical monocytes with immunosuppressive functions^48^. However, an opposite impact is observed in the LumA subtype, where the Mono_FCG3RA enrichment is associated with a better prognosis, reinforcing that MDC association with cancer prognosis is context-dependent.

Macrophages are versatile immune cells present in various tissues and their ontogeny and functional diversity have been the subject of extensive research. Studies have revealed that TAMs can be derived from circulating monocytes recruited to the tumor site or from RTMs that adapt to the tumor milieu^49^. These TAMs undergo polarization into various phenotypes influenced by their environment, such as the M1 and M2 states, classically associated with pro-inflammatory and immunosuppressive functions, respectively^50–52^. However, our study challenges the traditional binary division of macrophages since none of the states identified clearly displayed M1 or M2 signatures. Instead, we observed that some populations simultaneously expressed genes associated with both signatures, highlighting the complex nature of macrophage plasticity in the TME. To address this complexity and to follow recent efforts to standardize nomenclatures, our in-depth analysis identified twelve distinct TAM states, each characterized by a unique transcriptional profile and ontogeny. We based our annotation mainly on a recently proposed consensus model of TAM diversity gene signatures^9^, and better-characterized phenotypes that have not been extensively described, such as Mac_LA co-expressing *TREM2* and *PD1*, and three different profiles of RTMs (RTM_LA, RTM_IFN, and RTM_Int), which were frequently lumped together in a single group of cells in multiple TME studies^9,53^. We also reported RTM-like_MT, a subpopulation that displays markers associated with both ontogeny types, supporting the notion that under steady-state conditions, RTMs are mostly maintained via self-renewal processes. However, circulating monocytes can give rise to self-renewing RTMs if an adequate habitat is provided^54,55^.

We also investigated the expression of the members of the Triggering Receptor Expressed on Myeloid cells (TREM) family, *TREM1* and *TREM2*, associated with TAMs^56,57^. Our catalog revealed that TREM2^+^ macrophages^20^ can be further expanded into at least four different cell states including, RTM_LA, Mac_LA, Mac_IFN, and Mac_AgPres. Similarly, *TREM1* was mainly found expressed in into three subpopulations (Mac_Alv-like, Mac_Angio, and Mac_Hypo). These findings indicate that there is no single phenotype for TREM1^+^ or TREM2^+^ macrophages.

The Mac_LA subpopulation stands out for its significant expression of *TREM2* among the other Mac subpopulations, and its specific implication in clinical outcome is notable in TNBC, but not in other BRCA subtypes or HGSOC, despite the association of *TREM2* expression or prtein levels with poor overall survival, consistent with findings from other research groups^58–60^. These results suggest that in different tumor types, other TREM2-expressing macrophage subpopulations might play central roles in tumor progression within their respective microenvironments.

Other subpopulations expressing high levels of *TREM2*, such as RTM_LA and Mac_AgPres, are respectively associated with poor and good prognosis in LIHC. The co-expression of *TREM2* and the receptor *PD-1* in Mac_LA raises the possibility that this subpopulation may indeed have immunosuppressive roles in the TME, in line with previous findings^61^. An important axis of communication between malignant cells and these macrophages is the MIF:CD74 interaction axis. The activation of CD74 receptors in Mac_LA may contribute to the activation of an anti-inflammatory profile^62,63^. In mouse models of sarcoma, colorectal, breast, and gynecological cancers, *TREM2* deletion or blockade with a monoclonal antibody has been shown to reduce tumor growth, enhance antitumor CD8^+^ T cell responses, including the effectiveness of anti-PD–1 treatment, and modified the TAM landscape^61,64^. Our findings strongly support the notion that distinct TAM phenotypes expressing *TREM2* can coexist in the tumor microenvironment, exerting different roles, and contributing to the unclear dual function of *TREM2* in cancer prognosis^65^. Phase I clinical trial has been leading in advanced refractory tumors using specific TREM2 mAb against tumor-associated macrophages expressing TREM2^66^. The complex and context-dependent function of *TREM2*-expressing macrophages in the tumor microenvironment underscores the need for further research to fully understand its contribution to cancer progression and its potential as a therapeutic target. The identification of distinct TAM phenotypes expressing *TREM2* and their differential impact on cancer prognosis opens new avenues for precision medicine approaches and personalized treatment strategies.

In addition to *TREM2-*expressing macrophages, we also investigated the clinical impact of RTM_Int, a subpopulation expressing high levels of folate receptor β (FRβ), which is encoded by the *FOLR2* gene and serves as a marker for resident macrophages in the TME. High levels of this subpopulation are associated with a poor prognosis in TNBC and HGSOC, two very lethal tumor types, although a beneficial role has been seen for other breast cancer subtypes^8^. The presence of cells co-marked for FOLR2 and PDL-2 was significantly associated with poor outcomes in HGSOC-INCA highlighting its importance as a target for immunotherapies in a context-dependent maner^67–69^.

In addition, RTM_Int expresses β1 integrins (*ITGB1*), Insulin-like Growth Factor 1 (*IGF1*), and Tumor necrosis factor (*TNF*) that are involved in interaction with the receptor for activated C kinase (*RACK1*). These pathways are involved, in general, in therapy resistance and cell progression^70–72^. Our results also show the potential of utilizing FOLR2 as a biomarker for patient stratification and prognosis in these tumor types, in line with other studies that evaluated other tumor types^8,64,73,74^. Several trials involving targeting FOLR2 are currently ongoing, reflecting the current level of research interest using this approach^75,76^.

In conclusion, we have characterized in high-resolution the landscape of MDCs in solid tumors by effectively integrating a large dataset through a pipeline that uses cluster-specific gene expression analysis to infer cell type, distribution, function, ontogeny, phenotype, cellular interactions, and disease associations. This comprehensive reference atlas serves as a resource for the scientific community and provides insights into the identities and characteristics of mononuclear phagocytes in the TME, presenting new avenues for understanding and manipulating their behavior in cancer. Our findings also demonstrate that the reprogramming of TAMs is highly dependent on the microenvironment, leading to varied TAM expression profiles across different cancer types, with distinct clinical impacts. This highlights the importance of understanding the specificities of subpopulations/states in the TME context. By shedding light onto the intricate landscape of mononuclear phagocytes in the TME, our work pushes the development of more effective and targeted therapeutic interventions against cancer.

## Methods

### Ethical considerations

This retrospective study received approval from the Ethics in Human Research Committee of the Brazilian National Cancer Institute (INCA) (CAAE 52409221.4.0000.5274 and 61675516.9.0000.5274) in Rio de Janeiro, Brazil, and was conducted in accordance with all applicable regulations concerning the involvement of human study participants. Written informed consent was obtained from each patient prior to the commencement of study procedures. The study adhered to the Good Clinical Practice Guidelines.

### Data download and pre-processing

Pre-processed scRNA-Seq data (Supplementary Data 1) from patients with breast cancer (GSE114727), hepatocellular carcinoma (GSE140228, GSE125449), lung cancer (GSE127465), melanoma (GSE115979), ovarian cancer (GSE154600, GSE72056), uveal melanoma (GSE139829), skin (GSE130973) and metastasis from uveal melanoma and lung (GSE158803) samples were obtained from the public repository Gene Expression Omnibus (GEO) using the GEOquery Bioconductor package^77^. Additionally, datasets from lung, ovarian, and breast cancer were downloaded from Qian et al.^78^, on its platform (blueprint.lambrechtslab.org/). We downloaded datasets from the Human Lung Cell Atlas project^79^ on the Synapse platform (SYN21560407). Finally, the dataset containing PBMCs (10x Genomics standard) was downloaded from the company platform (support.10xgenomics.com/single-cell-gene-expression/datasets/1.1.0/pbmc3k).

Raw gene expression matrices were analyzed with the Seurat package (v4.0)^80^ for each study. We converted all gene symbols to Ensembl format (hg v38). Different filters of quality control were applied by technology: for 10x Genomics data the cutoff points were percentage of mitochondrial genes expressed <10, number of counts (nCounts) per cell >200, and ratio of nCounts by number of features (nFeatures) <5; for Smart-Seq2 were percentage of mitochondrial genes expressed <15, nCounts >200, and ratio nCounts/nFeature <1000; and for inDrop were percentage of mitochondrial genes expressed <15, nCount >200, and ratio nCounts/nFeature <1000. The remaining cells were submitted to the doublet removal step through the Scrublet package v0.2.3^81^. Through the doublet score histogram distribution, a cutoff value was determined for each library by manually setting the threshold (Optimal pK), eliminating cells with a higher probability of being doublets.

### Integration and cluster annotation

The datasets were concatenated with Scanpy (v1.7.2)^82^, and the 3000 highly variably expressed genes were selected using flavor = “Seurat”. The integration was performed with scVI (v0.6.8)^28^ from scvi-tools (v0.19.0)^83^. For training the variational autoencoder neural network, we used the following hyperparameters: n_latent = 20, n_layers=4, dropout_rate = 0.1. After training the scVI model and correcting for batch effects using “study” and “tissue origin” as co-variables in order to remove the technical bias, the clusters were calculated through the Leiden algorithm^84^. Different resolutions were evaluated, ranging from 0.3 to 2.0, and the resolution of 0.6 was selected for the annotation of broad cell types based on gene profiles. The latent space generated by scVI was then projected to a two-dimensional space using the UMAP dimensional reduction method^85^. To determine the broad cell types, we use canonical markers (Supplementary Data 3). We found and removed three undefined/contaminated subsets with low quality (clusters 17, 19, and 29). After this first level of annotation, we applied DecontX^86^, from the celda package (v1.14.1), using default parameters, to estimate and remove ambient RNA contamination in individual droplets. Post DecontX, we repeated the step of first-level cell type annotation, refining the cell type annotations after contamination removal. This approach was designed to ensure the preservation of clusters with minimal contamination and to maintain the integrity of genuine biological signals that could potentially be mistaken for contamination.

For the other levels of annotation, we subset the broad cell types and then rerun highly variable genes and scVI models with the same parameters, except for batch correction, which was performed by each sample. We also exclude samples with a contribution of fewer than ten cells. In order to classify clusters in cell types or states, we apply DEG analysis by Wilcoxon rank-sum and MAST v1.16.0^87^, using logfc.threshold = 0.25, and min.pct = 0.1. The FDR method was used to adjust p-values. In addition, we evaluated the purity of the clusters through the ROGUE algorithm (v1.0)^88^ with default parameters.

### Integration benchmarking

We performed an Integration benchmark on macrophages data comparing six integration tools: Scanorama v1.7.4^89^, Harmony v.1.2.0^27^, BBKNN v.0.2.0^90^, FastMNN v4.0^91^, scanVI^29^ and scVI^28^, that we used to integrate our dataset, and also the unintegrated data. For that we used the scib tool^26^ and its metrics. Those metrics are described below:

Bio conservation metrics:

- Isolated labels: score how well isolated labels are distinguished from all other labels using the average-width silhouette score (ASW);

- KMeans NMI: the normalized mutual information (NMI) is a version of the mutual information corrected by the entropy of clustering and ground truth labels (e.g., cell type). The score ranges between 0 and 1, with 0 representing no sharing and 1 representing perfect sharing of information between clustering and annotated cell labels;

- KMeans ARI: the adjusted rand index (ARI) is a chance-adjusted rand index, which evaluates the pair-wise accuracy of clustering vs. ground truth label assignments. The score ranges between 0 and 1 with larger values indicating better conservation of the data-driven cell identity discovery after integration compared to annotated labels;

- Silhouette label: wrapper for sklearn silhouette function, including average silhouette width (ASW). Values range from [−1, 1] with 1 indicates distinct, compact clusters, 0 indicates overlapping clusters, and −1 indicates core-periphery (non-cluster) structure. By default, the score is scaled between 0 and 1.

Batch correction metrics:

- Silhouette batch: this metric measures the silhouette of a given batch. It assumes that a silhouette width close to 0 represents perfect overlap of the batches, thus the absolute value of the silhouette width is used to measure how well batches are mixed.;

- Graph connectivity: quantify the connectivity of the subgraph per cell type label.

### Copy number variation inference

To differentiate between malignant and non-malignant epithelial cells, our study utilized an analysis of Copy Number Variations (CNVs). We estimated the CNV score for individual cells by applying inferCNVpy software (v0.4.0), available at github.com/icbi-lab/infercnvpy. This tool is an extension of the principles used in inferCNV^92^. We defined the malignancy status of epithelial cells based on their relative CNV scores that were calculated considering all cells in the dataset. Specifically, epithelial cells with a CNV score below the established median threshold of 0.004 were deemed “non-malignant.” In contrast, those exhibiting a CNV score above this median were classified as “malignant,” suggesting the presence of genomic changes commonly associated with cancer cells.

### Unsupervised hierarchical clustering analysis

For the clustering analysis and dendrogram construction, we calculated the mean expression of the 750 most variable genes in MDCs using Seurat’s AverageExpression function. Pearson correlation coefficients were obtained and used to calculate the distance by the formula (1 - Pearson correlation)/2. We then constructed the dendrogram using the pvclust R package^93^, with the correlation method set to “cor”, hierarchical clustering to “average”, and bootstrap replications to 10,000 for stability assessment. Jaccard bootstrap mean values were calculated with the fpc R package’s^94^ cluster boot function, employing 10,000 bootstrap samples and average linkage clustering with k = 5 clusters.

### Comparison with previously identified macrophage subpopulations

In order to validate our findings, we correlated the populations we have defined with those from previous works. Another set of pre-processed scRNA-Seq data was downloaded^20,21^ (Supplementary Data 6). Those two datasets were selected because they are considered robust amounts of data, well-annotated regarding mononuclear phagocytes, and with a great diversity of tumor types, including those we have worked with in this paper. We used the same strategy and quality control filters that were used with our main datasets. The datasets were analyzed separately, using the authors’ annotations for macrophages, monocytes, and DC subpopulations. To perform the correlation, expression data was log-normalized using the *NormalizedData()* function of the Seurat package (v4.0)^80^, followed by the AverageExpression function from the same package to determine the average expression of each gene for each subpopulation. *Cor()* function^95^ from the stats R library was used to correlate the expression of all genes between subpopulations, and ggplot2 v3.4.4^96^ was used to plot the correlation graphs. The purity of clusters was verified using ROGUE^88^ with default parameters.

### External validation: construction of a secondary pan-cancer atlas with updated datasets

#### Data download and pre-processing

Pre-processed single-cell RNA-sequencing (scRNA-Seq) data from various cancer types^57,97–108^ were acquired (Supplementary Data 7) from GEO using the GEOquery Bioconductor package. The data encompassed head and neck cancer (GSE181919), renal cell carcinoma (GSE159115), pancreatic adenocarcinoma (GSE205013), prostate cancer (GSE185344), endometrial cancer (GSE251923), and multiple datasets for breast cancer (GSE176078, GSE161529), ovarian cancer (GSE140819, GSE147082), gastric cancer (GSE163558), and thyroid cancer (GSE184362). Additional datasets for colorectal cancer (E-MTAB-8410) and more datasets for breast (EGAD00001006608) and ovarian cancers (EGAS00001004935) were retrieved from alternative databases.

For the creation of the validation pan-cancer atlas, each dataset was subjected to uniform filtering standards. Quality control parameters were adapted to each sequencing technology: for data generated by 10x Genomics, cells were retained if they displayed less than 10% mitochondrial gene expression, over 200 detected genes (nCounts), and a nCounts to nFeatures ratio of less than 5. For inDrop and Drop-seq technologies, the criteria were less than 15% mitochondrial gene expression, more than 200 nCounts, and a nCounts to nFeatures ratio below 1000. Subsequent to these filters, we applied the Scrublet package for doublet removal, setting the cutoff (Optimal pK) manually based on the distribution of doublet scores to exclude cells with a high probability of being doublets.

##### Integration and myeloid cells subset

For processing, samples originating from the same study were merged using the Scanpy software (version 1.7.2). We then identified the 3000 most variably expressed genes using the “Seurat” flavor setting for selection. Integration across datasets was carried out using scVI (version 0.6.8) as part of the scvi-tools suite (version 0.19.0). The variational autoencoder neural network was trained with specific hyperparameters: the number of latent dimensions was set to 20, the model was constructed with 4 layers, and a dropout rate of 0.1 was implemented to prevent overfitting. Batch effects correction was performed using each sample as co-variables. Cluster determination was subsequently performed using the Leiden algorithm. To visualize the data in a two-dimensional framework, we employed UMAP technique on the latent space generated by scVI.

In our analysis, we explored a range of resolutions from 0.3 to 2.0 to accurately cluster myeloid cells within various datasets. The evaluation revealed that a resolution setting of 0.3 suited most datasets effectively. Nonetheless, for certain studies—namely the Thyroid cancer^100^, the Breast Cancer^104^, and the Endometrial Cancer^109^—alternate resolutions of 0.4, 0.4 and 0.6, respectively, provided better clustering results. Myeloid cell populations were identified using the canonical markers LYZ, AIF1, and CD68. To maintain consistency in gene annotations across all datasets, we standardized them to the human genome hg38 assembly.

Myeloid cell clusters were subsetted and the process was rerun for identifying highly variable genes and the scVI model adhering to the previously established parameters. Additionally, we removed samples contributing fewer than ten cells to refine our dataset. In order to classify mononuclear phagocytes, we apply DEG analysis by Wilcoxon rank-sum, using logfc.threshold = 0.25, and min.pct = 0.1. The FDR method was used to adjust *p* values (<0.05).

To confirm the robustness of the myeloid signatures identified, we integrated the discovery (reference) cohort with the validation cohort using the scANVI algorithm, which leveraged predefined cell type labels to facilitate cell type prediction. After integrating and confirming the cell types within the validation cohort, we recalculated the nearest neighbors and UMAP, and then conducted a reanalysis of gene expression, taking into account the previously identified differentially expressed genes.

### Metabolic characterization

We characterized the metabolic pathways of each cluster using Compass v.0.9.7.1^110^. Cells were subsetted into clusters prior to running. The following parameters were used: --calc-metabolites --microcluster-size 10 --lambda 0. Raw reaction penalties were converted to reaction scores as described in the original publication, and reactions with zero variance were removed. Pairwise comparison was performed for each cluster (e.g., Cluster 1 vs. Cluster 2; Cluster 1 vs. Cluster n; etc) applying Wilcoxon’s test and calculating Cohen’s d effect size between means. The FDR method was used to adjust p-values. Reactions with confidence scores below 4 (most confident) or with an adjusted p-value greater than 0.1 were removed. Cohen’s d median scores were calculated for each pairwise comparison and metabolic pathway. Those median scores were again aggregated by median comparing the median Cohen’s d value across all clusters to obtain a single value per cluster and metabolic pathway, which were z-scored to compare across macrophage clusters.

Reaction scores calculated for each myeloid cluster were pairwise compared between all clusters. Reactions were filtered for adjusted p-value below 0.1 and a measure of the effect size was calculated using the median of Cohen’s values for each metabolic pathway. Next, Cohen’s medians for each metabolic pathway were summarized for each cluster to reflect the activity of the respective pathway in the cluster. The metabolic reactions Glycolysis/Gluconeogenesis, Citric Acid Cycle, NAD Metabolism, Oxidative Phosphorylation, Arginine and Proline Metabolism, Transport, Mitochondrial, Fatty Acid Oxidation, Fatty Acid Synthesis, Glycerophospholipid Metabolism, Glycosphingolipid Metabolism, Sphingolipid Metabolism are used to classified the macrophage based in the metabolic pathway.

### Trajectory analysis through pseudo-time

We employed Monocle 3 (v.1.3.1)^111^ with the UMAP generated by the integration with scVI as a “partition”. We set the parameter use_partition = FALSE of the function “learn graph” in order to create a linear trajectory, with default parameters. To order cells, the root used to infer the pseudotime was the Mono_CD14_FOS^-^ cluster, with default parameters. We also used Monocle 3 to perform gene module analysis based on graph autocorrelation to identify genes that co-vary over pseudotime.

### Scores and pathway enrichment

The top hundred genes identified in the DEGs analysis, sorted by the log fold change for each cluster, were selected to perform the functional enrichment analysis with the curated database Reactome (2021)^112^ through overrepresentation analysis (ORA), which consists of a one-sided Fisher’s exact test, using the clusterProfiler R package (v3.0)^113^. We considered significant pathways those with a Benjamini-Hochberg (BH) adjusted p value ≤0.05. The comparisons were made among the following cell types: Mac_Angio (n = 3108), Mac_Hypo (n = 1802), Mac_ES (n = 4073), Mac_IFN (n = 1981), Mac_Prolif (n = 2234), Mac_LA (n = 1249), Mac_AgPres (n = 3965), Mac_alv-like (n = 8816), RTM_like-MT (n = 1209), RTM_IFN (n = 2061), RTM_LA (n = 3569), RTM_Int (n = 3507). Some pathways were selected for plotting based on the scientific experience of the authors. The redundancy was removed from the enriched pathway set by selecting the pathway with the lowest p-value. All enriched pathways by subpopulations are available in Supplementary Data 5. The gene signature scores were computed by the AddModuleScore_UCell^114^ function. The gene set used for the scores is available in Supplementary Data 4.

### Cell–cell communication

To perform cell communication analyses, we only considered cells detected with more than 200 genes and genes with detection in more than 50 cells. The CellComm algorithm v.1^115^ was used to infer communications between pairs of cell types including macrophages and tumors or lymphocytes. We used as a set of possible intercellular communications the 688 ligands and 857 receptors contained in the NicheNet database^116^ resulting in 12650 possible pairs. In communications between macrophages and tumors, we used cells classified as “MacAlv-like”, “Mac_Angio”, “Mac_Hypo”, “Mac_IFN”, “Mac_LA”, “Mac_ES”, “Mac_AgPres”, “RTM-like_MT”, RTM_IFN”, “RTM_Int” and “RTM_LA” and breast, colorectal, liver, lung, melanoma and ovarian tumor cells. In communications between macrophages and lymphocytes, we used cells classified as macrophages “Mac_IFN”, “Mac_LA”, “Mac_ES”, “RTM_IFN” and “RTM_Int” and lymphocytes “NKT”, “TCD4ex”, “TCD4reg”, “TCD8em”, “TCD8ex” and “TGD”. For consideration of intercellular communication, the expression of ligands and receptors should be greater than 0.25 normalized counts and show a p-value less than 0.01 in a permutation test of cluster annotation containing 1000 permutations. In order to display the main figures we applied the following filters: expression values of the ligands, receptors and mean of both higher than 1 and the p-value lower than 0.002. All interactions are shown in the Supplementary Data 9.

### Deconvolution analysis

To comprehensively understand the heterogeneity of the TME, as well as the cellular composition in each tumor type, we applied deconvolution methods to predict the abundance of cell types by modeling gene expression levels through bulk RNA-Seq data. For these analyses, gene expression data (HTSeq counts), and clinical-pathological information from the database portal TCGA (http://cancergenome.nih.gov/) were downloaded using the TCGAbiolinks v.2.28^117^ package of nine different tumor types, using the identifiers: OV (n = 354 samples), LUAD (n = 510), LUSC (n = 496), UVM (n = 77), SKCM (divided into Metastatic (n = 385) and Primary (n = 103) tumor samples), COAD (n = 454), READ (n = 170), LIHC (n = 369) and BRCA (n = 1195) which comprises Luminal A (n = 569), Luminal B (n = 210), HER2 (n = 81) and TNBC (n = 188) subtypes. The single-cell signatures identified in this work was used as a reference using BayesPrism package^118^ (v2.0) to predict the relative frequency of cell types for each patient of each tumor type. We considered 47 cell signatures, including cell types identified in the first level of annotation, such as fibroblasts, endothelial cells, B lymphocytes, mast cells, pDC, malignant cells, and normal epithelial cells, and cell states/subtypes identified in other levels of clustering, including macrophages (n = 12, except the Mac_Prolif subpopulation), monocytes (n = 6), DC (n = 8), T cells (n = 9), NK cells (n = 2), and neutrophil (n = 3) subpopulations. The gene expression counts matrix was filtered to only contain the 12,854 genes that make up the signatures of the cell types. Only cell types representing more than 1% of in each tumor type were considered. Ribosomal and mitochondrial genes with high expression were removed using the *cleanup.genes()* function. In addition, genes expressed in less than 5 cells were excluded. After that, to get more consistent results, we filtered for protein-coding genes and used the function *get.exp.stat()* to select differentially expressed genes between cell states of different cell types using the default parameters, except for the parameter *pseudo.count* (0.1 for 10x data and 10 for Smart-seq data). Finally, we set up the prism object using the *new.prism()* function for each tumor type by setting the following parameters:

*reference=scRNAfiltered_matrix,mixture=bulk_matrix,input.type* = *“count.matrix”,cell.type.labels = celltype, cell.state.labels = states, key* = *“Malignant Cells”, outlier.cut* = *0.01, outlier.fraction*

 = *0.1*. And then running BayesPrism, *run.prism(prism = prism object, n.cores* = *70)*.

### Study design and patient selection

For the ovary cohort, 193 women diagnosed with HGSOC at INCA between 2001 and 2017, regardless of adjuvant or neoadjuvant treatment, were included in our study. The TNBC-INCA cohort, previously described by our collaborators^119^, consisted of 112 women who underwent neoadjuvant chemotherapy followed by curative surgery between 2010 and 2014. Clinical data regarding sex, age at diagnosis, staging, surgery, histological subtype, chemotherapy, and survival were retrospectively obtained from medical records. Both Brazilian cohorts are described in more detail in Supplementary Data 12. Patient sex information was sourced from medical records for both conditions.

### Immunohistochemistry analysis

The blocks were cut with a microtome into fine slivers of 4-mm sections to form the tissue microarray which encompasses the most representative areas of greatest tumor cellularity in formalin-fixed paraffin-embedded tissue specimens. The immunohistochemistry was processed using a NOVOLINK™ Polymer Detection Systems (Leica), followed by 3,3′-Diaminobenzidine (DAB). Antigen retrieval was performed with the Trilogy (Cell Marque) reagent. The samples were immunostained for Rabbit Monoclonal anti-Ki67 (clone 30-9 at 1:7, Ventana- Roche), Rabbit monoclonal anti-CD8 (cat. 108R-14, clone SP57 at 1:7, Ventana- Roche), Mouse monoclonal anti-PD-1 (cat. 315M-94, clone NAT105, Cell Marque, diluted 1:100), Mouse monoclonal anti-CD68 (cat. 168 M-95, clone KP-1 at 1:20, Ventana- Roche), Mouse monoclonal anti-FOLR2 (cat. MA5-26933, OTI4G6 at 1:200, ThermoFisher), Rabbit monoclonal anti-TREM2 (cat. 91068T, clone D814C, 1:100, Cell Signaling Technology) and Rabbit polyclonal anti-PDL-2 (ab200377, at 1:200, Abcam). For double staining, TREM2 was combined with anti-CD68, and anti-FOLR2 with anti-PDL-2. Briefly, after completing the first immune reaction (TREM2 or PDL-2) with DAB, the second immune reaction (CD86 or FOLR2) was visualized using MACH4 MR-AP (Biocare Medical) green subtract (Polydetector HRP Green Substrate-chromogen Bio-SB - ref BSB0129t). The entire analysis was carried out at the Division of Pathology - INCA by experienced pathologists and the slides were scanned by Aperio ImageScope v12.4.6.5003.

Tissue microarrays were assessed by determining the percent of immune cells and their intra or peritumoral distribution under the supervision of a qualified pathologist. The cutoffs for positive staining were established based on positive and negative controls, as well as her experience as a pathologist. These cutoffs were chosen based on their clinical and pathological significance (Table 1).

**Table 1:** Cutoffs established for high and low expression groups in immunohistochemistry analysis

	HGSOC		TNBC	
	High	Low	High	Low
Cells TREM2+ and CD68+	≥10%	0%	≤1%	≥2%
Cells CD68+	0%	≥10%	-	-
Cells FOLR2+	≤25%^a^	≥75%^a^	-	-
Cells PD-1+	0–1%	3–30%	0–1%	≥1%
Cells CD8+	0–1%^a^	≥3%^a^	0–1%	≥5%

^a^Only cells in the peritumoral region were selected.

FOLR2 and the TREM2 were accessed by ImageJusing the IHC Profiler ImageJ Plugin^120^.The separation of the color layers was based on hematoxylin and 3,3′-Diaminobenzidine. For each patient, we calculated the average of the percentage values obtained from ImageJ for all the spots. Then, the score for each average value was calculated based on the IHC Profiler tutorial, as follows: percentage of high positive (4 × % area of high positive staining), positive (3 × % area of positive staining), and low positive (2 × % area of low positive staining) staining. Finally, the score per sample was defined as the higher value among them.

The expression of the co-markers FOLR2 and PDL-2 in TNBC-INCA and HGSOC-INCA was measured by HALO software version 3.6 (Indica Labs, Albuquerque, NM, USA). Spots were segmented using the TMA module and positive cells (displaying both markers) were quantified via the Multiplex IHC module by defining thresholds for each marker.

In the immunohistochemistry analysis of the TNBC-INCA cohort, each patient was represented by three tissue spots, whereas in the HGSOC-INCA cohort, each patient was represented by two spots. The analysis involved calculating the average staining intensity per patient, ensuring that the data represented a robust measure of the marker being studied across multiple tissue samples.

### Survival and statistical analysis

To assess differences in means (scores and clinical impact), we employed the Kruskal-Wallis method for overall comparisons, complemented by the Wilcoxon test for specific paired comparisons. Survival analysis was visualized using the Kaplan-Meier method, facilitated by the Survival (v3.5) and Survminer (v0.4.9) packages^121,122^. Branches were defined using two approaches depending on how the data was collected. The first approach was the use of the “surv_cutpoint” function from the “survminer” package to define groups. This function determines optimal cutpoints for continuous variables that are used to stratify patients into different risk groups. The “surv_cutpoint” function applies a maximally selected rank statistics method to identify the best-performing threshold based on survival outcomes, ensuring an unbiased separation of groups despite the uneven distribution of sample sizes. This approach was used to define groups in the following cases:

- Macrophage subpopulations estimations in bulk RNA-Seq samples from TCGA that were derived through deconvolution analysis. The cut-off points for Mac_LA, as determined by “surv_cutpoint”, were a score of 0.11 for TNBC samples and 3.26 for HGSOC samples. As for RTM_Int, the cut-off was 9.67 for TNBC samples and 0.0078 for HGSOC samples.
- FOLR2 and TREM2 global levels quantified using the ImageJ software with the IHC Profiler plugin. The cut-off points determined by “surv_cutpoint” were scores equal to 71.5 and 83.8 for TNBC-INCA samples when observing global expression levels of FOLR2 and TREM2, respectively.
- Tissue microarray slides of HGSOC-INCA and TNBC-INCA patients stained with hematoxylin, eosin, and co-marked with PDL-2 and FOLR2 analyzed using HALO software version 3.6 (Indica Labs, Albuquerque, NM, USA). The cut-offs determined by “surv_cutpoint” were 7.5 cells for HGSOC samples and 53.5 for TNBC samples.

For the evaluation of double staining of TREM2 and CD68, the quantification of markers was performed under the supervision of a qualified pathologist. The cutoffs for positive staining were established based on positive and negative controls, as well as her experience as a pathologist. These cutoffs were chosen based on their clinical and pathological significance and described in the Immunohistochemistry section. Hazard ratios for survival analysis were computed using both univariate and multivariate Cox proportional hazards models. Statistical testing throughout the study was conducted on a two-sided basis. To address the challenge of multiple hypothesis testing, the Benjamini–Hochberg procedure was applied to adjust *P* values, and False Discovery Rate (FDR) Q values were subsequently calculated. Results were considered statistically significant at *P* value or FDR *Q* value of <0.05.

Regarding the clinical parameters used, the clinical annotation of TCGA dataset, and the times calculated for OS and PFS were downloaded from the PanCanAtlas study. For the INCA cohorts, OS was calculated considering the day from the diagnosis to the date of death of any cause if the patient was known to be alive on the last day of data collection. The PFS was calculated from the last chemotherapy data or the last registration to recurrence data if the patient was known to be alive or the date of death. The Kaplan-Meier method was used to visualize the difference between the curves of the high and low groups. The statistical analyses were conducted using R project version 3.5.3.

### Inclusion and ethics

The research process involved local researchers in all stages, and all collaborators meeting the authorship criteria mandated by Nature Communication journals have been included as authors, given their vital roles in designing and executing the study. Roles and responsibilities were established among collaborators prior to the research. The study incorporates locally relevant findings, identified in collaboration with local partners. Ethical approval was sought from local ethics review committees where appropriate. Research restrictions or prohibitions in the researchers’ setting did not apply, and participant risks such as stigmatization, incrimination, or discrimination were avoided. Our citations acknowledge the relevance of local and regional research to our study.

### Reporting summary

Further information on research design is available in the Nature Portfolio Reporting Summary linked to this article.

### Supplementary information

Supplementary information

Peer Review File

Description of Additional Supplementary Files

Dataset 1

Dataset 2

Dataset 3

Dataset 4

Dataset 5

Dataset 6

Dataset 7

Dataset 8

Dataset 9

Dataset 10

Dataset 11

Dataset 12

Reporting Summary

### Source data

Source Data

## Data Availability

The published datasets related to the main analysis can be accessed under GEO accession numbers: GSE114727, GSE140228, GSE125449, GSE115978, GSE154600, GSE130973, GSE139829, GSE158803; URLs for additional data are www.synapse.org/#!Synapse:syn21041850/wiki/600865 and lambrechtslab.sites.vib.be/en/dataaccess, as well as support.10xgenomics.com/single-cell-gene-expression/datasets/1.1.0/pbmc3k. The public datasets related to the validation analysis can be accessed under GEO accession numbers: GSE181919, GSE159115, GSE205013, GSE251923, GSE185344, GSE176078, GSE161529, GSE140819, GSE147082, GSE163558, GSE184362; European Genome-phenome Archive study IDs EGAD00001006608 [ega-archive.org/studies/EGAS00001004809], EGAS00001004935 [ega-archive.org/studies/EGAS00001004935] and ArrayExpress accession number E-MTAB-8410 (see also Supplementary Data 1 and 6). The integrated dataset is publicly available and can be downloaded via CELL×GENE | Collections through the following link: cellxgene.cziscience.com/collections/3f7c572c-cd73-4b51-a313-207c7f20f188. Source data are provided with this paper.

## Code availability

The code for reproducing the analyses and figures of this paper is available in the GitHub repository: github.com/bioinformatics-inca/MyeloidDerivedCells_in_TME^123^.
