# Supplementary material for: Single-cell resolution characterization of myeloid-derived cell states with implication in cancer outcome
Source: Nat Commun. 2024 Jul 7;15:5694. doi: 10.1038/s41467-024-49916-4 (PMC11228020; doi:10.1038/s41467-024-49916-4)
Supplement: Supplementary file 1 — Supplementary information [file 41467_2024_49916_MOESM1_ESM.pdf]

Supplementary Information

Single-cell resolution characterization of myeloid-derived cell states with implication in cancer outcome

Gabriela Rapozo Guimarães et al.

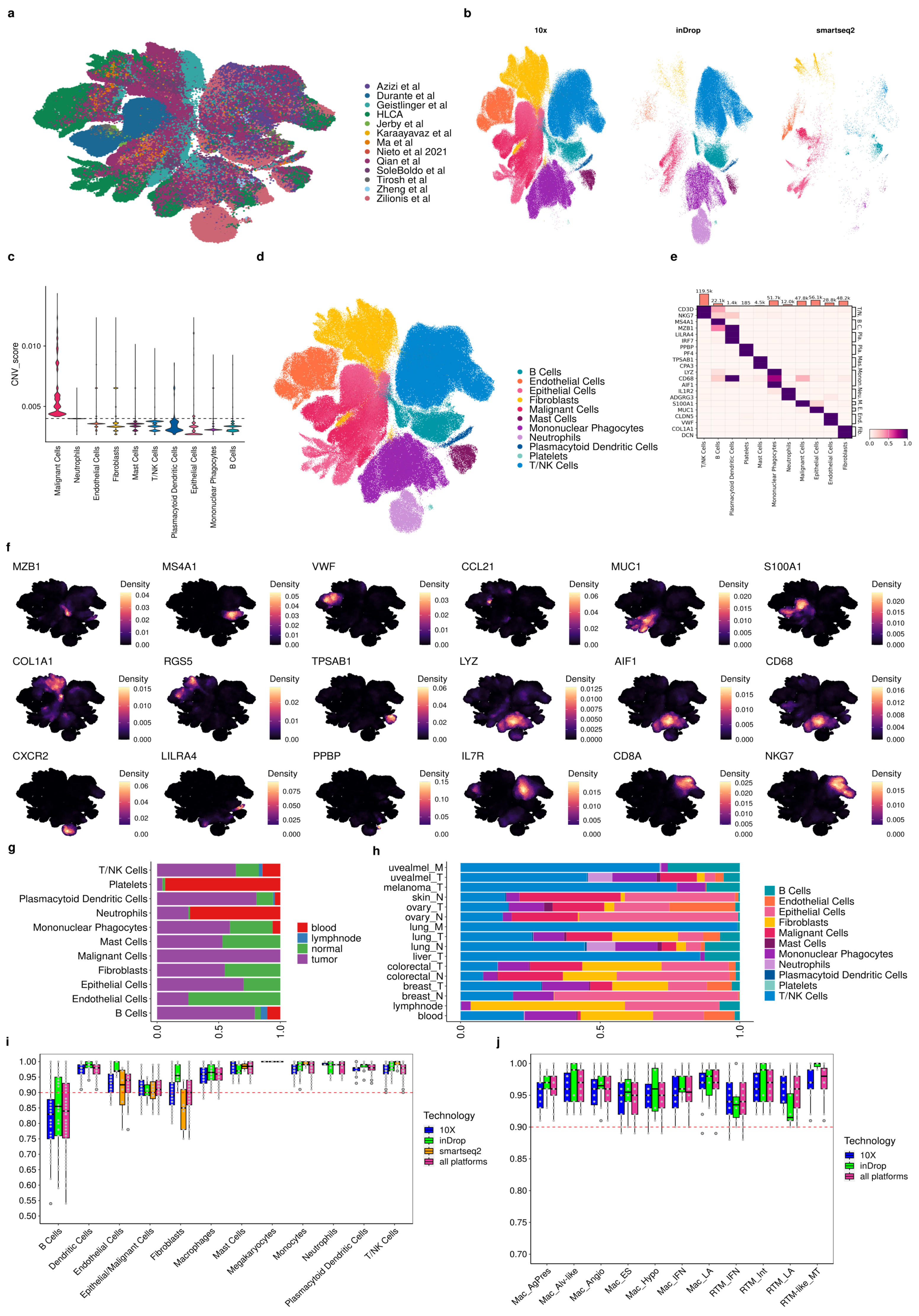
a
Azizi et al
Durante et al
Geistlinger et al
HLCA
Jerby et al
Karaayavaz et al
Ma et al
Nieto et al 2021
Qian et al
SoleBoldo et al
Tirosh et al
Zheng et al
Zilionis et al
b
10x
inDrop
smartseq2
c
CNV_score
Malignant Cells
Neutrophils
Endothelial Cells
Fibroblasts
Mast Cells
T/NK Cells
Plasmacytoid Dendritic Cells
Epithelial Cells
Mononuclear Phagocytes
B Cells
d
B Cells
Endothelial Cells
Epithelial Cells
Fibroblasts
Malignant Cells
Mast Cells
Mononuclear Phagocytes
Neutrophils
Plasmacytoid Dendritic Cells
Platelets
T/NK Cells
e
f
MZB1
MS4A1
VWF
CCL21
MUC1
S100A1
COL1A1
RGS5
TPSAB1
LYZ
AIF1
CD68
CXCR2
LILRA4
PPBP
IL7R
CD8A
NKG7
Density
g
blood
lymphnode
normal
tumor
h
uvealmel_M
uvealmel_T
melanoma_T
skin_N
ovary_T
ovary_N
lung_M
lung_T
lung_N
liver_T
colorectal_T
colorectal_N
breast_T
breast_N
lymphnode
blood
i
Technology
10X
inDrop
smartseq2
all platforms
B Cells
Dendritic Cells
Endothelial Cells
Epithelial/Malignant Cells
Fibroblasts
Macrophages
Mast Cells
Megakaryocytes
Monocytes
Neutrophils
Plasmacytoid Dendritic Cells
T/NK Cells
j
Technology
10X
inDrop
all platforms
Mac_AgPres
Mac_Alv-like
Mac_Angio
Mac_ES
Mac_Hypo
Mac_IFN
Mac_LA
RTM_IFN
RTM_Int
RTM_LA
RTM-like_MT

Supplementary Figure 1. scRNA-seq profiling of 13 data sets across seven human cancer types. (a) Uniform Manifold Approximation and Projection (UMAP) representing all cell populations and their distribution across the studies. (b) UMAP split by technologies, showing the integration by cell similarities. (c) Violin plot showing the copy number variation (CNV) score by broad cell type, the dashed line represents the cut-off applied. (d) UMAP colour-coded by the eleven broad cell types: B cells (n = 22,102) were identified by the expression of MZB1 and MS4A1; Endothelial cells (n = 28,775) were marked by VWF and CCL21; MUC and S100A1 expression allowed the identification of normal (n = 56,060) and malignant (n = 47,827) epithelial cells, which were further distinguished by the amount of CNV events observed in the malignant cells. Fibroblasts (n = 48,159) by COL1A1 and RGS5; Mast cells (n = 4,539), identified by TPSAB1; Mononuclear Phagocytes were positive for LYZ, AIF1, and CD68; Neutrophils (n = 12,033), marked by CXCR2; Plasmacytoid Dendritic Cells (pDC) (n = 1,365) by LILRA4 and IRF7; megakaryocytes (n = 185) marked by PPBP expression; and T and Natural Killer (NK) cells (n = 119,472) identified by the expression of IL7R or CD8A, or NKG7. and expression; and (e) Heatmap showing the markers' genes across the 11 broad cell types and the number of cells per type. The color scale reflects the log10-normalized gene expression range. (f) Density plots highlighting the gene expression of each cell. (g) Bar plot showing the proportion of cell types across the sample types. (h) Bar plot showing the proportion of clusters across conditions and sample types. Each column represents the sample types for a given condition, if any, such as normal (_N), primary tumor (_T), and tumor metastasis (_M). Purity of (i) broad cell type clusters and (j) macrophages post-scVI integration, evaluated using ROGUE scores. Points represent individual samples, with ROGUE values above the recommended threshold of 0.9 (dashed line) indicating high cluster purity. Source data are provided as a Source Data file.

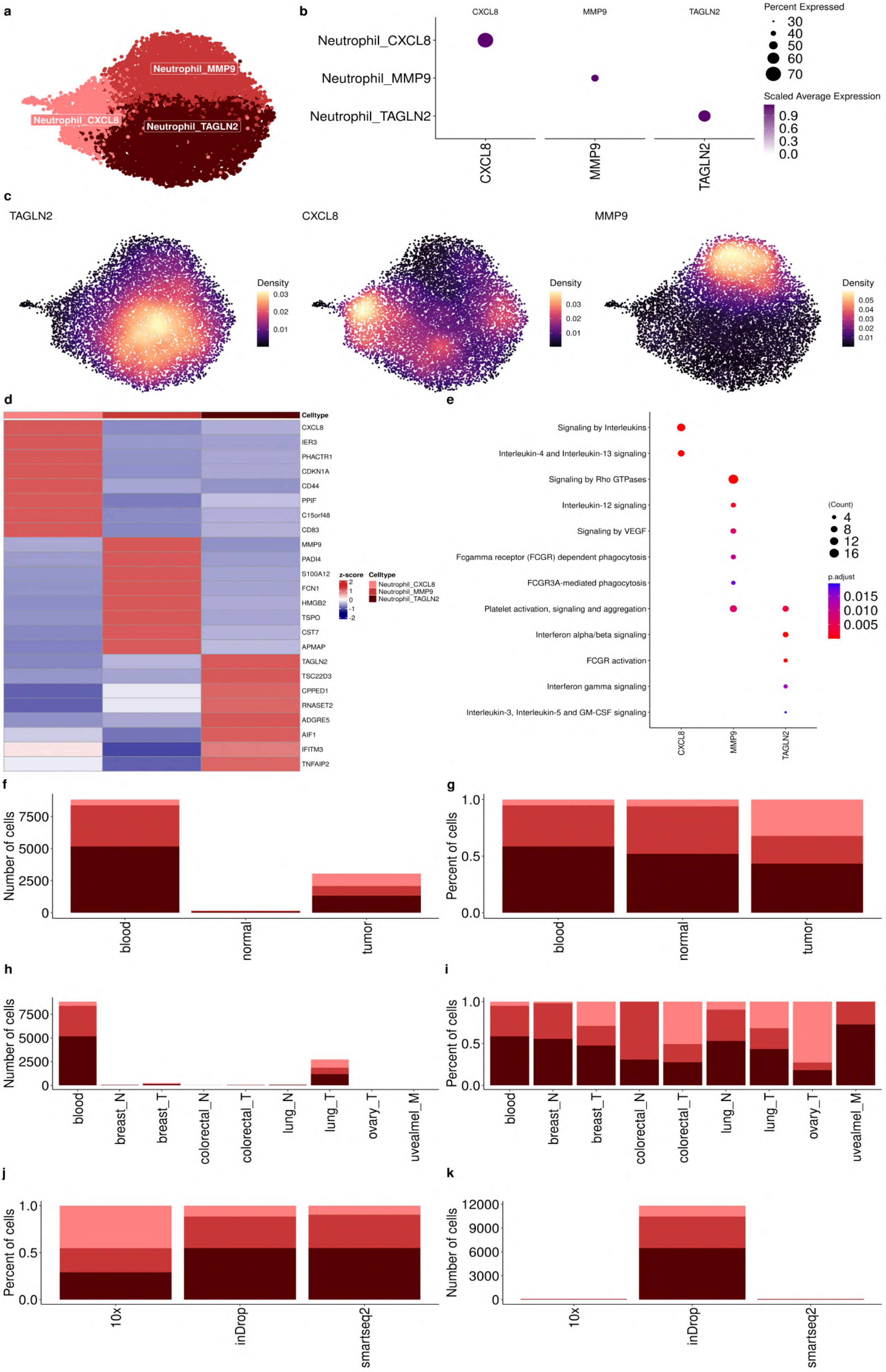

a
Neutrophil_MMP9
Neutrophil_CXCL8
Neutrophil_TAGLN2
b
CXCL8
MMP9
TAGLN2
Percent Expressed
30
40
50
60
70
Scaled Average Expression
0.9
0.6
0.3
0.0
c
TAGLN2
CXCL8
MMP9
Density
d
Celltype
CXCL8
IER3
PHACTR1
CDKN1A
CD44
PPIF
C15orf48
CD83
MMP9
PADI4
S100A12
FCN1
HMGB2
TSPO
CST7
APMAP
TAGLN2
TSC22D3
CPPED1
RNASET2
ADGRE5
AIF1
IFITM3
TNFAIP2
z-score
e
Signaling by Interleukins
Interleukin-4 and Interleukin-13 signaling
Signaling by Rho GTPases
Interleukin-12 signaling
Signaling by VEGF
Fcgamma receptor (FCGR) dependent phagocytosis
FCGR3A-mediated phagocytosis
Platelet activation, signaling and aggregation
Interferon alpha/beta signaling
FCGR activation
Interferon gamma signaling
Interleukin-3, Interleukin-5 and GM-CSF signaling
(Count)
p.adjust
0.015
0.010
0.005
f
Number of cells
blood
normal
tumor
g
Percent of cells
h
breast_N
breast_T
colorectal_N
colorectal_T
lung_N
lung_T
ovary_T
uvealmel_M
i
j
10x
inDrop
smartseq2
k

Supplementary Figure 2. Neutrophils across tumor samples. (a) UMAP of neutrophils subpopulations colored by distinct subpopulations based on gene expression profile. (b) Dot plot showing the mean expression of neutrophil subpopulations. Dot size indicates the fraction of expressing cells, colored based on scaled expression levels. (c) Density plots highlighting the gene expression of each cell. (d) Heatmap showing the markers' genes across neutrophils subpopulation. (e) Enrichment pathways analysis of neutrophil using the Reactome database. The size of each circle represents the number of genes and such circles are colored by p-adjust. (f-k) Bar plot showing the distribution of the number of neutrophils subpopulations across (f-g) sample and (h-i) tumor types, and (j-k) technologies. Source data are provided as a Source Data file.

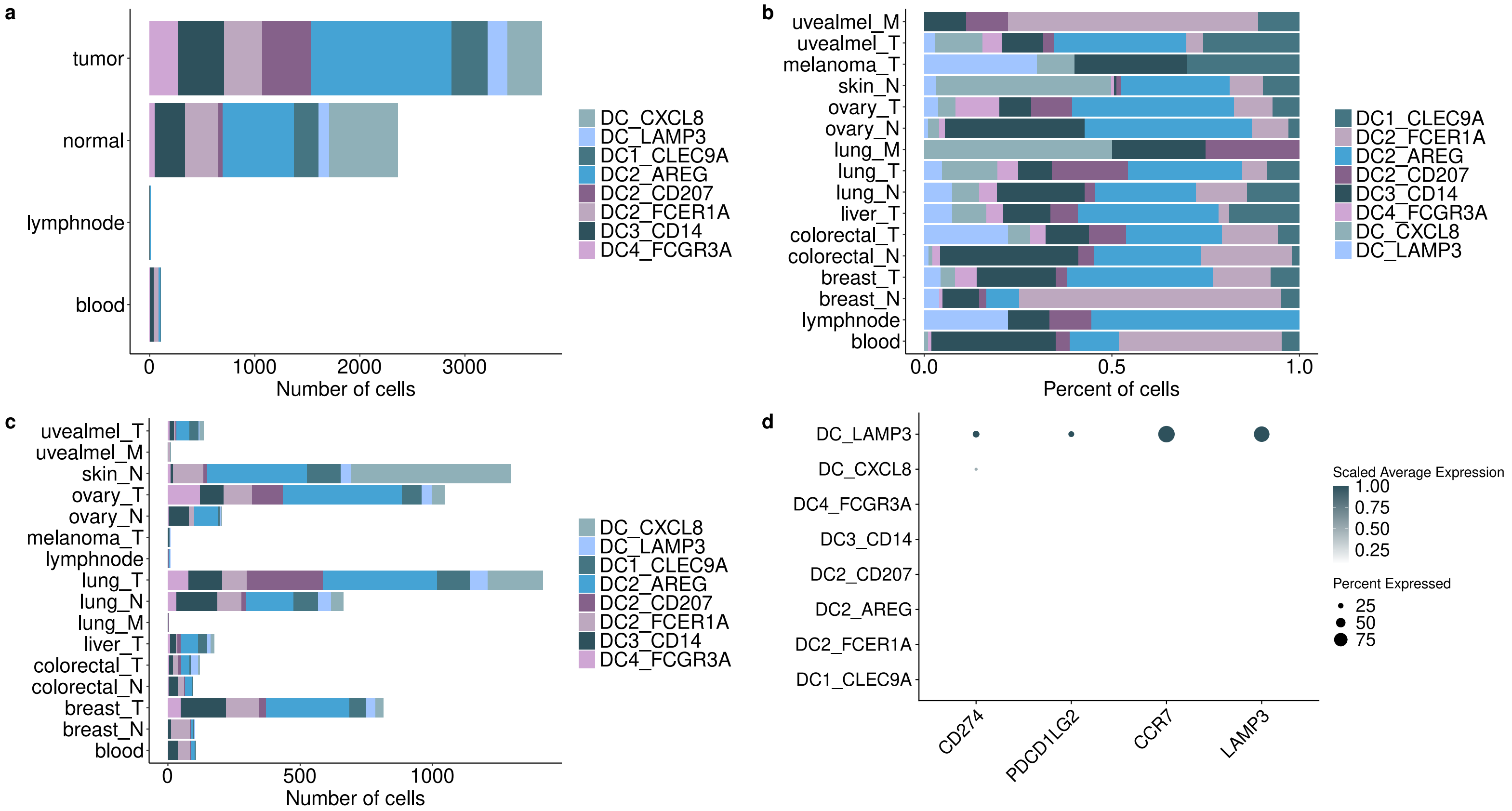

Supplementary Figure 3. Dendritic Cells' distribution across tumor samples. (a-c) Bar plot showing the distribution of DC cells across sample types (a) and conditions (b-c). (d) Dot plot showing the immunosuppressive genes expression in DC_LAMP3. Dot size indicates the percent of expressing cells, and the dot color the scaled average expression. Source data are provided as a Source Data file.

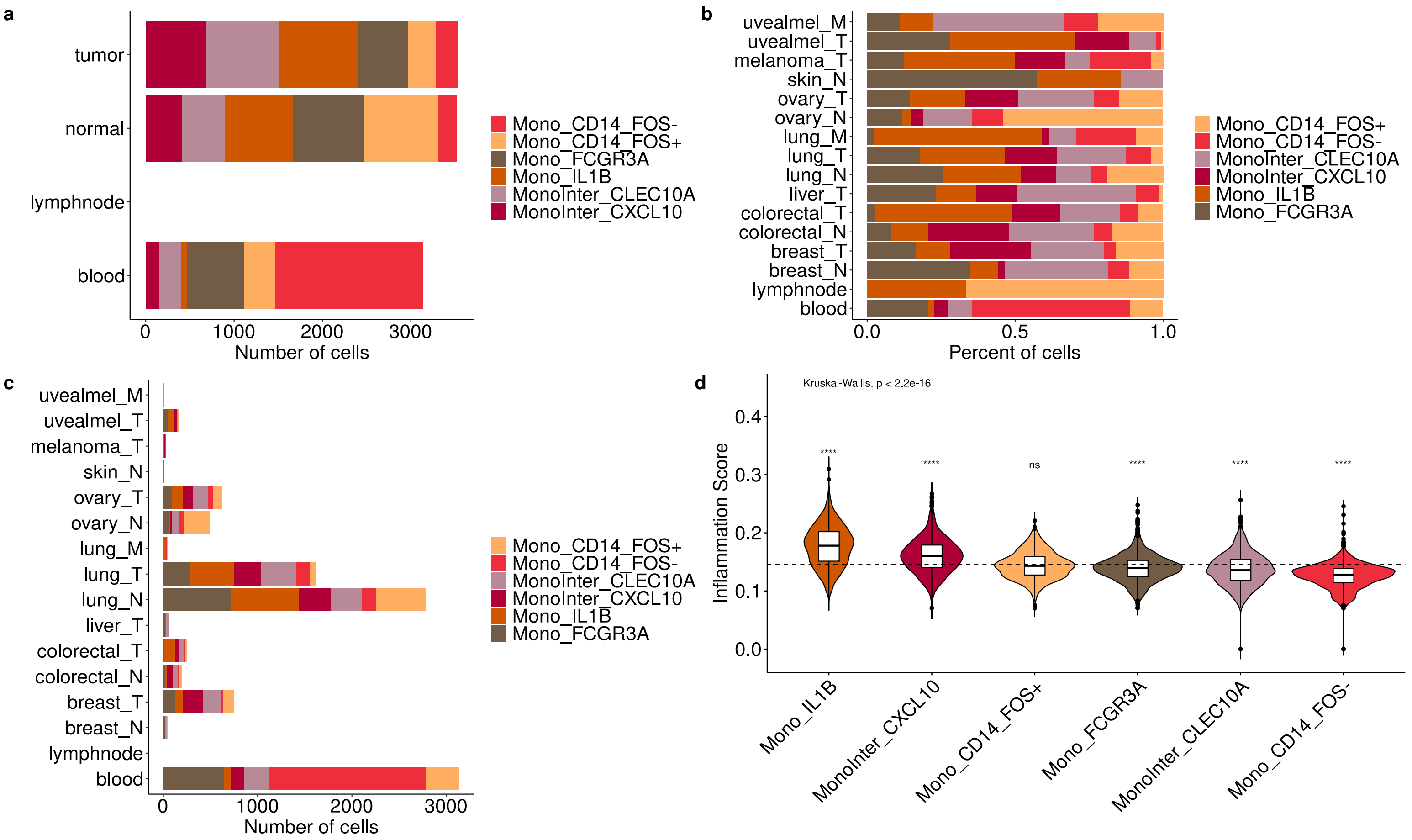

Supplementary Figure 4. Monocytes' distribution across the tumor samples. (a-c) Bar plot showing the distribution of Mono across sample types (a) and conditions (b-c). (d) Violin plot demonstrating the inflammation score among Mono subpopulations. Dashed lines represent the average score. Box indicates the range from 25th to 75th percentile, with whiskers extending to 1.5 times the interquartile range. Outliers are plotted separately, center indicates the median value. For statistical significance, we performed the Kruskall–Wallis test (p < 2.2x10-16) followed by Wilcoxon to compare each group against "all" (i.e. base-mean). Ns non-significant.; ****p < 0.0001. Source data are provided as a Source Data file.

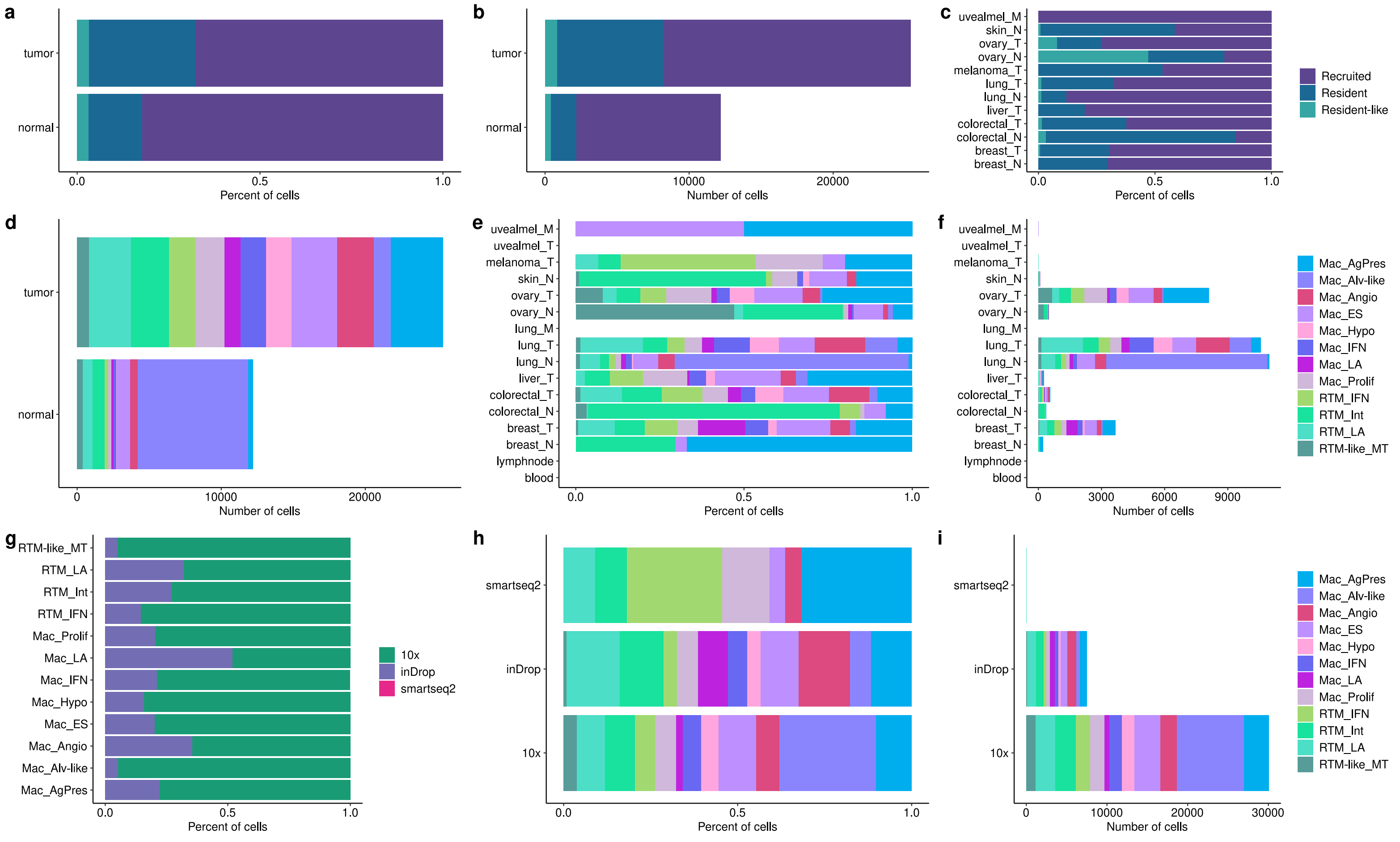

Supplementary Figure 5. Macrophages' distribution across tumor samples. (a-c) Bar plot showing the distribution of monocyte-derived and resident-macrophages across (a-b) samples and (c) conditions. (d-f) Bar plot showing the distribution of Mac subpopulations across (d) samples and (e-f) conditions. (g-i) Bar plot showing the distribution of Mac subpopulations across technologies.

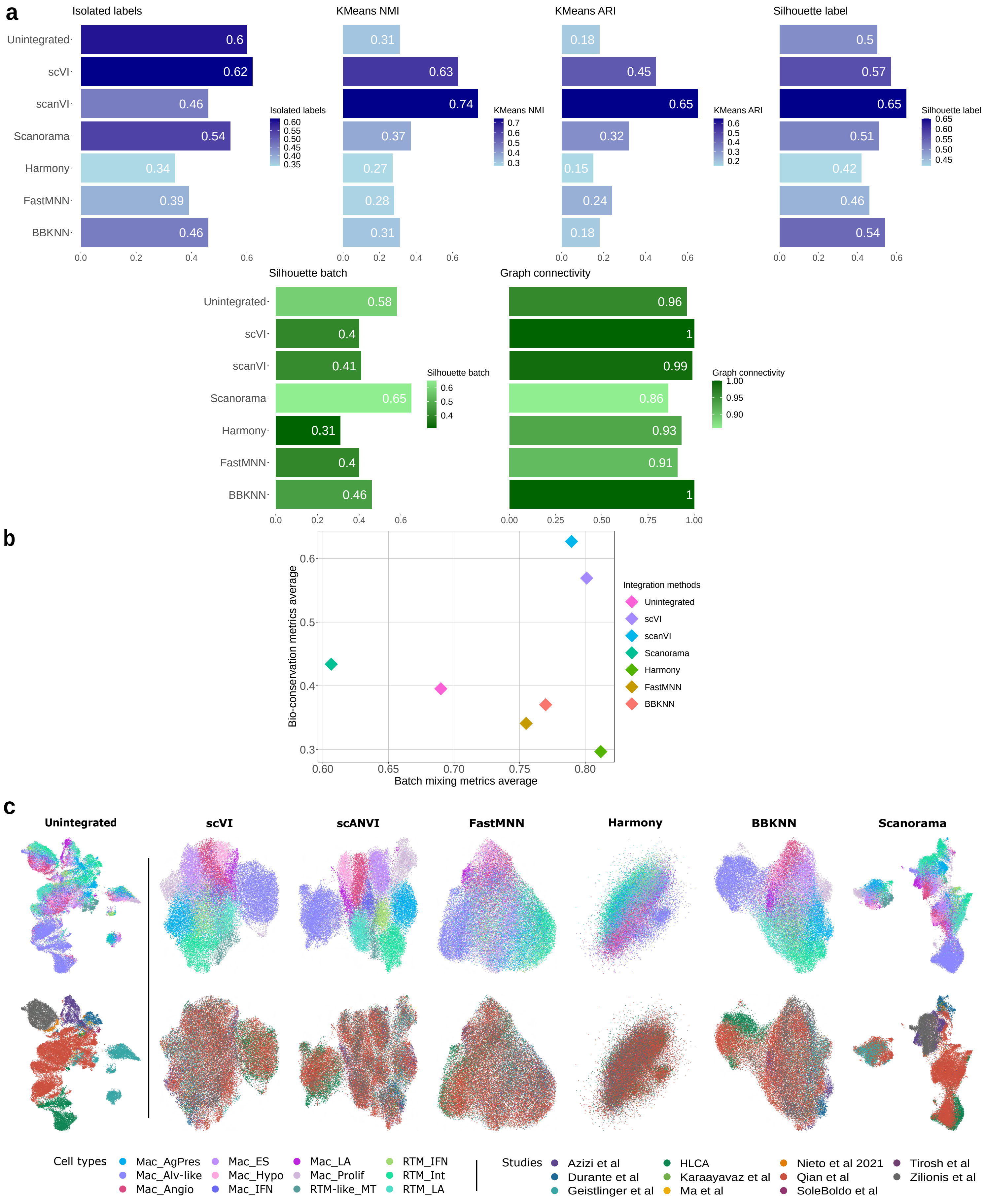

a
Isolated labels
KMeans NMI
KMeans ARI
Silhouette label
Unintegrated
scVI
scanVI
Scanorama
Harmony
FastMNN
BBKNN
0.6
0.62
0.46
0.54
0.34
0.39
0.46
0.31
0.63
0.74
0.37
0.27
0.28
0.31
0.18
0.45
0.65
0.32
0.15
0.24
0.18
0.5
0.57
0.65
0.51
0.42
0.46
0.54
Silhouette batch
Graph connectivity
0.58
0.4
0.41
0.65
0.31
0.4
0.46
0.96
1
0.99
0.86
0.93
0.91
1
b
Bio-conservation metrics average
Batch mixing metrics average
Integration methods
Unintegrated
scVI
scanVI
Scanorama
Harmony
FastMNN
BBKNN
c
Unintegrated
scVI
scANVI
FastMNN
Harmony
BBKNN
Scanorama
Cell types
Mac_AgPres
Mac_Alv-like
Mac_Angio
Mac_ES
Mac_Hypo
Mac_IFN
Mac_LA
Mac_Prolif
RTM-like_MT
RTM_IFN
RTM_Int
RTM_LA
Studies
Azizi et al
Durante et al
Geistlinger et al
HLCA
Karaayavaz et al
Ma et al
Nieto et al 2021
Qian et al
SoleBoldo et al
Tirosh et al
Zilionis et al

Supplementary Figure 6. Performance metrics for different data integration tools applied to the myeloid-derived cells. (a) Each bar chart corresponds to a specific metric used to assess the quality of the data integration described in the methodology section. The color code in the figure corresponds to the performance of each integration tool on the respective metric. Darker colors indicate better performance, while lighter colors represent poorer performance. (b) Scatter plot of the bio-conservation metrics average versus batch mixing metrics average for all the integration methods used in this work. (c) UMAP visualizations of scRNASeq data following integration by different computational methods. Cells are color coded by cell types showing the impact of different integration methods on the preservation of cellular identities while in data is colored by the originating study, highlighting the method's ability to integrate datasets from diverse sources.

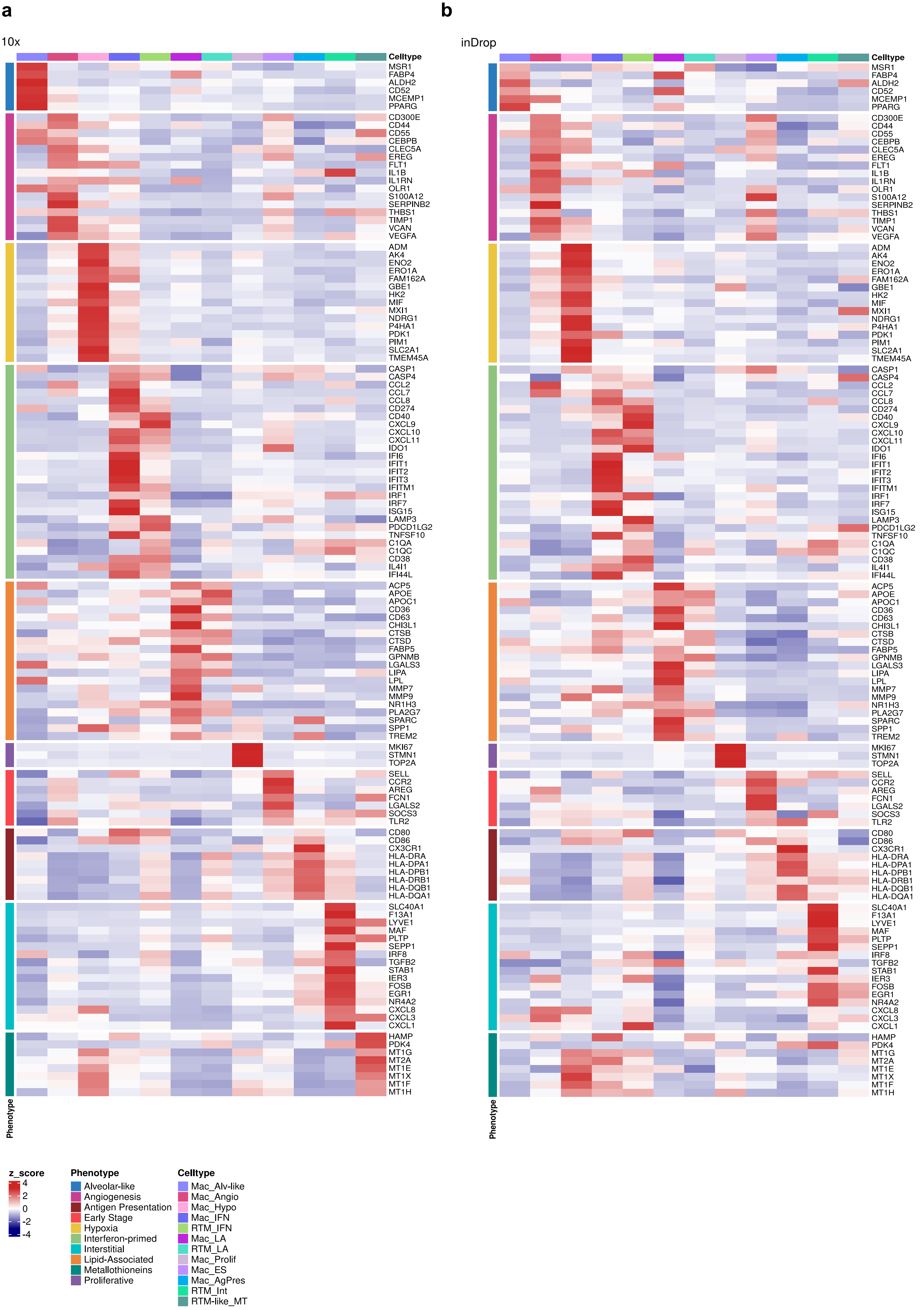
a
10x
Celltype
MSR1
FABP4
ALDH2
CD52
MCEMP1
PPARG
CD300E
CD44
CD55
CEBPB
CLEC5A
EREG
FLT1
IL1B
IL1RN
OLR1
S100A12
SERPINB2
THBS1
TIMP1
VCAN
VEGFA
ADM
AK4
ENO2
ERO1A
FAM162A
GBE1
HK2
MIF
MXI1
NDRG1
P4HA1
PDK1
PIM1
SLC2A1
TMEM45A
CASP1
CASP4
CCL2
CCL7
CCL8
CD274
CD40
CXCL9
CXCL10
CXCL11
IDO1
IFI6
IFIT1
IFIT2
IFIT3
IFITM1
IRF1
IRF7
ISG15
LAMP3
PDCD1LG2
TNFSF10
C1QA
C1QC
CD38
IL4I1
IFI44L
ACP5
APOE
APOC1
CD36
CD63
CHI3L1
CTSB
CTSD
FABP5
GPNMB
LGALS3
LIPA
LPL
MMP7
MMP9
NR1H3
PLA2G7
SPARC
SPP1
TREM2
MKI67
STMN1
TOP2A
SELL
CCR2
AREG
FCN1
LGALS2
SOCS3
TLR2
CD80
CD86
CX3CR1
HLA-DRA
HLA-DPA1
HLA-DPB1
HLA-DRB1
HLA-DQB1
HLA-DQA1
SLC40A1
F13A1
LYVE1
MAF
PLTP
SEPP1
IRF8
TGFB2
STAB1
IER3
FOSB
EGR1
NR4A2
CXCL8
CXCL3
CXCL1
HAMP
PDK4
MT1G
MT2A
MT1E
MT1X
MT1F
MT1H
Phenotype
b
inDrop
Celltype
Phenotype
z_score
4
2
0
-2
-4
Phenotype
Alveolar-like
Angiogenesis
Antigen Presentation
Early Stage
Hypoxia
Interferon-primed
Interstitial
Lipid-Associated
Metallothioneins
Proliferative
Celltype
Mac_Alv-like
Mac_Angio
Mac_Hypo
Mac_IFN
RTM_IFN
Mac_LA
RTM_LA
Mac_Prolif
Mac_ES
Mac_AgPres
RTM_Int
RTM-like_MT

Supplementary Figure 7. Reproducibility of signatures across different platforms. (a) Heatmap showing the gene signature per subpopulation for 10x technology data. The color scale represents the scaled expression of each gene. (b) Heatmap showing the gene signature per subpopulation for inDrop technology data. The color scale represents the scaled expression of each gene. Source data are provided as a Source Data file.

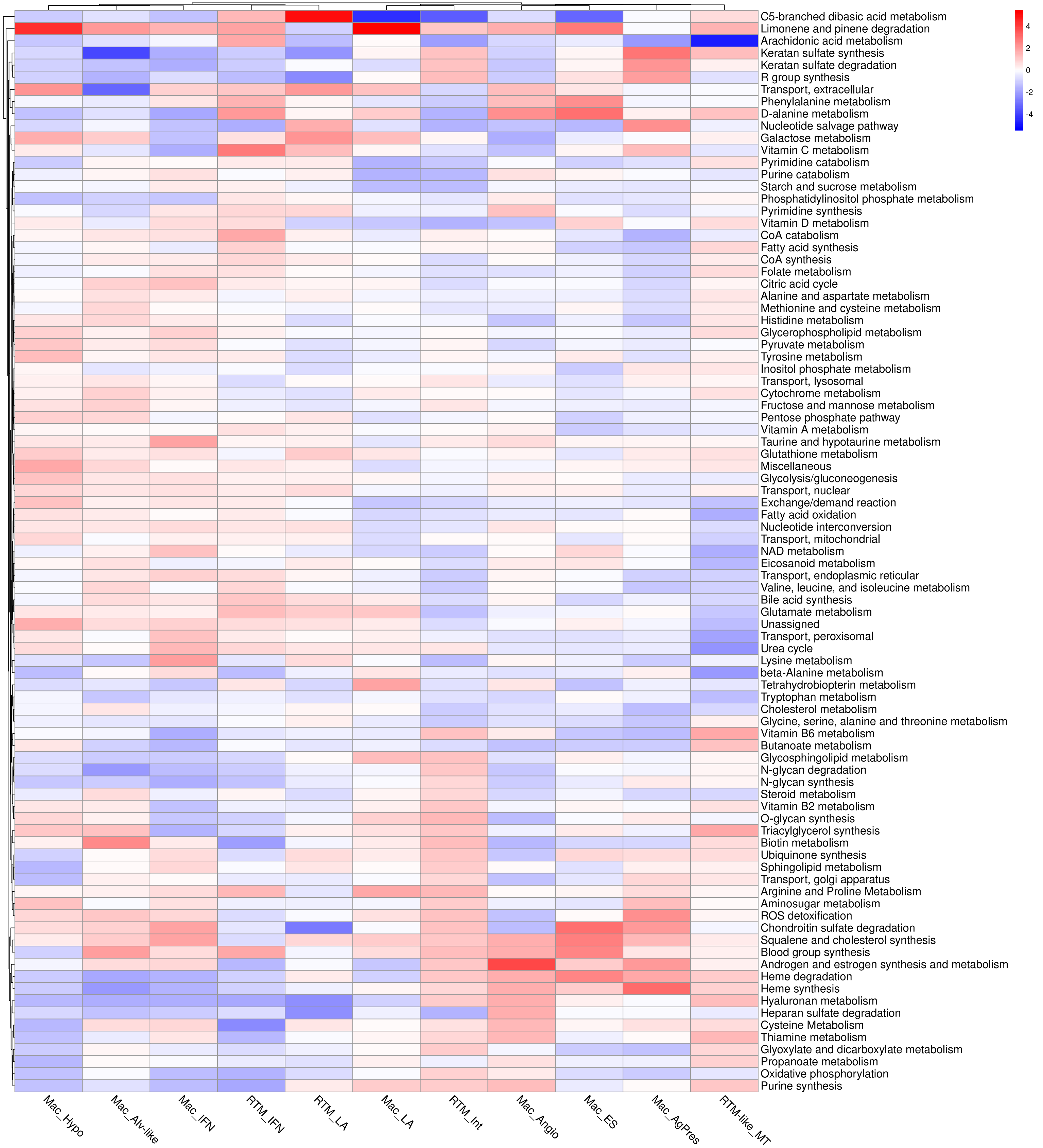
4
2
0
-2
-4
C5-branched dibasic acid metabolism
Limonene and pinene degradation
Arachidonic acid metabolism
Keratan sulfate synthesis
Keratan sulfate degradation
R group synthesis
Transport, extracellular
Phenylalanine metabolism
D-alanine metabolism
Nucleotide salvage pathway
Galactose metabolism
Vitamin C metabolism
Pyrimidine catabolism
Purine catabolism
Starch and sucrose metabolism
Phosphatidylinositol phosphate metabolism
Pyrimidine synthesis
Vitamin D metabolism
CoA catabolism
Fatty acid synthesis
CoA synthesis
Folate metabolism
Citric acid cycle
Alanine and aspartate metabolism
Methionine and cysteine metabolism
Histidine metabolism
Glycerophospholipid metabolism
Pyruvate metabolism
Tyrosine metabolism
Inositol phosphate metabolism
Transport, lysosomal
Cytochrome metabolism
Fructose and mannose metabolism
Pentose phosphate pathway
Vitamin A metabolism
Taurine and hypotaurine metabolism
Glutathione metabolism
Miscellaneous
Glycolysis/gluconeogenesis
Transport, nuclear
Exchange/demand reaction
Fatty acid oxidation
Nucleotide interconversion
Transport, mitochondrial
NAD metabolism
Eicosanoid metabolism
Transport, endoplasmic reticular
Valine, leucine, and isoleucine metabolism
Bile acid synthesis
Glutamate metabolism
Unassigned
Transport, peroxisomal
Urea cycle
Lysine metabolism
beta-Alanine metabolism
Tetrahydrobiopterin metabolism
Tryptophan metabolism
Cholesterol metabolism
Glycine, serine, alanine and threonine metabolism
Vitamin B6 metabolism
Butanoate metabolism
Glycosphingolipid metabolism
N-glycan degradation
N-glycan synthesis
Steroid metabolism
Vitamin B2 metabolism
O-glycan synthesis
Triacylglycerol synthesis
Biotin metabolism
Ubiquinone synthesis
Sphingolipid metabolism
Transport, golgi apparatus
Arginine and Proline Metabolism
Aminosugar metabolism
ROS detoxification
Chondroitin sulfate degradation
Squalene and cholesterol synthesis
Blood group synthesis
Androgen and estrogen synthesis and metabolism
Heme degradation
Heme synthesis
Hyaluronan metabolism
Heparan sulfate degradation
Cysteine Metabolism
Thiamine metabolism
Glyoxylate and dicarboxylate metabolism
Propanoate metabolism
Oxidative phosphorylation
Purine synthesis
Mac_Hypo
Mac_Alv-like
Mac_IFN
RTM_IFN
RTM_LA
Mac_LA
RTM_Int
Mac_Angio
Mac_ES
Mac_AgPres
RTM-like_MT

Supplementary Figure 8. Overview of the main metabolic pathways in the macrophages subpopulations. Heatmap showing the main metabolism signature for each Mac states. The color scale represents the scaled expression of each metabolic pathway. Source data are provided as a Source Data file.

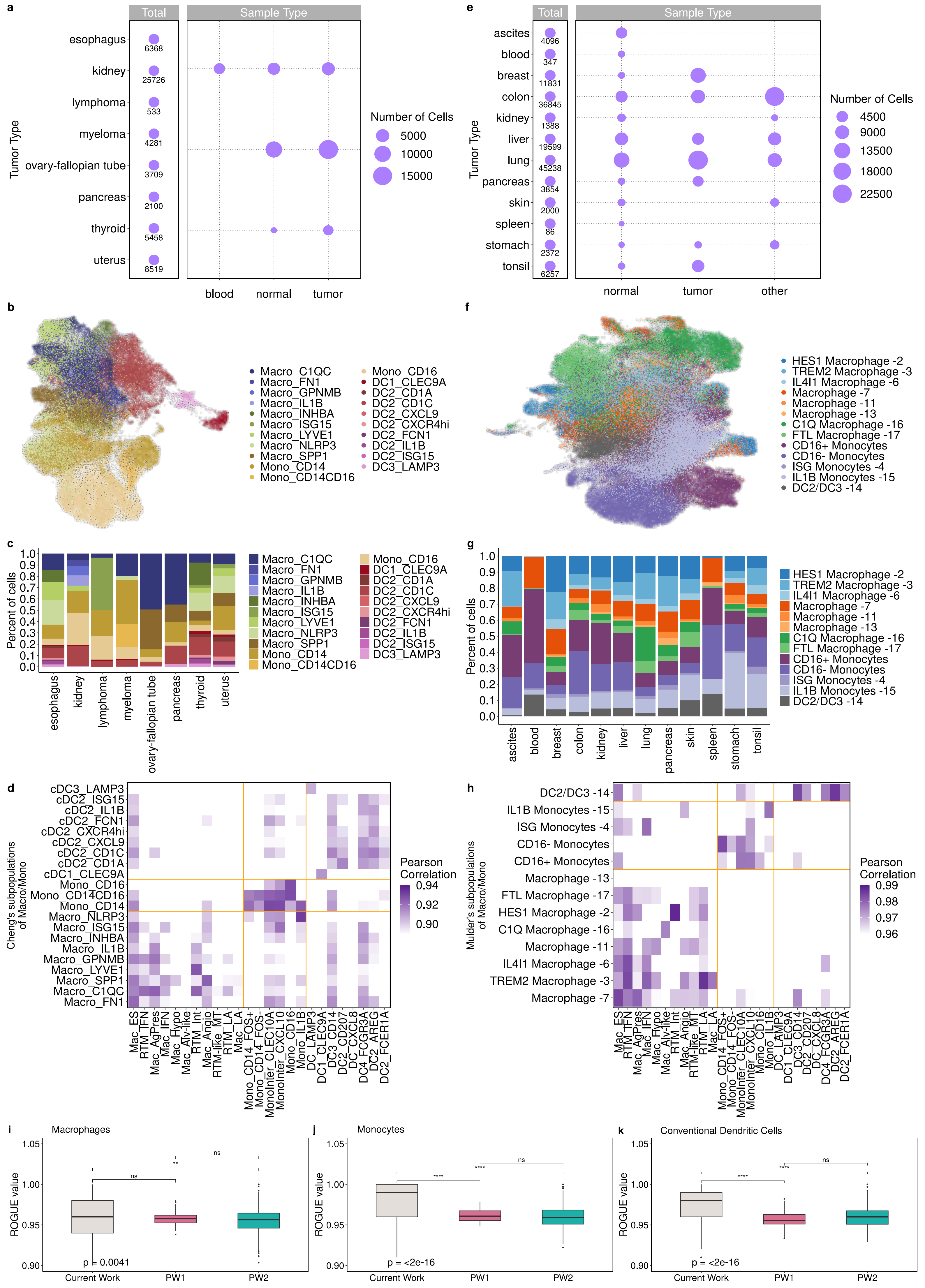
a
Total
Sample Type
Tumor Type
esophagus
6368
kidney
25726
lymphoma
533
myeloma
4281
ovary-fallopian tube
3709
pancreas
2100
thyroid
5458
uterus
8519
blood
normal
tumor
Number of Cells
5000
10000
15000
e
Total
Sample Type
Tumor Type
ascites
4096
blood
347
breast
11831
colon
36845
kidney
1388
liver
19599
lung
45238
pancreas
3854
skin
2000
spleen
86
stomach
2372
tonsil
6257
normal
tumor
other
Number of Cells
4500
9000
13500
18000
22500
b
Macro_C1QC
Macro_FN1
Macro_GPNMB
Macro_IL1B
Macro_INHBA
Macro_ISG15
Macro_LYVE1
Macro_NLRP3
Macro_SPP1
Mono_CD14
Mono_CD14CD16
Mono_CD16
DC1_CLEC9A
DC2_CD1A
DC2_CD1C
DC2_CXCL9
DC2_CXCR4hi
DC2_FCN1
DC2_IL1B
DC2_ISG15
DC3_LAMP3
f
HES1 Macrophage -2
TREM2 Macrophage -3
IL4I1 Macrophage -6
Macrophage -7
Macrophage -11
Macrophage -13
C1Q Macrophage -16
FTL Macrophage -17
CD16+ Monocytes
CD16- Monocytes
ISG Monocytes -4
IL1B Monocytes -15
DC2/DC3 -14
c
Percent of cells
1.0
0.9
0.8
0.7
0.6
0.5
0.4
0.3
0.2
0.1
0.0
esophagus
kidney
lymphoma
myeloma
ovary-fallopian tube
pancreas
thyroid
uterus
g
Percent of cells
ascites
blood
breast
colon
kidney
liver
lung
pancreas
skin
spleen
stomach
tonsil
d
Cheng's subpopulations of Macro/Mono
cDC3_LAMP3
cDC2_ISG15
cDC2_IL1B
cDC2_FCN1
cDC2_CXCR4hi
cDC2_CXCL9
cDC2_CD1C
cDC2_CD1A
cDC1_CLEC9A
Mono_CD16
Mono_CD14CD16
Mono_CD14
Macro_NLRP3
Macro_ISG15
Macro_INHBA
Macro_IL1B
Macro_GPNMB
Macro_LYVE1
Macro_SPP1
Macro_C1QC
Macro_FN1
Mac_ES
RTM_TFN
Mac_AgPres
Mac_IFN
Mac_Hypo
Mac_Alv-like
RTM_Int
Mac_Angio
RTM-like_MT
RTM_LA
Mac_LA
Mono_CD14_FOS+
Mono_CD14_FOS-
MonoInter_CLEC10A
MonoInter_CXCL10
Mono_CD16
Mono_IL1B
DC_LAMP3
DC1_CLEC9A
DC3_CD14
DC2_CD207
DC_CXCL8
DC4_FCGR3A
DC2_AREG
DC2_FCER1A
Pearson Correlation
0.94
0.92
0.90
h
Mulder's subpopulations of Macro/Mono
DC2/DC3 -14
IL1B Monocytes -15
ISG Monocytes -4
CD16- Monocytes
CD16+ Monocytes
Macrophage -13
FTL Macrophage -17
HES1 Macrophage -2
C1Q Macrophage -16
Macrophage -11
IL4I1 Macrophage -6
TREM2 Macrophage -3
Macrophage -7
Pearson Correlation
0.99
0.98
0.97
0.96
i
Macrophages
ROGUE value
1.05
1.00
0.95
0.90
ns
**
ns
p = 0.0041
Current Work
PW1
PW2
j
Monocytes
ROGUE value
****
****
ns
p = <2e-16
Current Work
PW1
PW2
k
Conventional Dendritic Cells
ROGUE value
****
****
ns
p = <2e-16
Current Work
PW1
PW2

Supplementary Figure 9. Cross-referencing new macrophage subpopulations against previous descriptions. Cheng and colleagues' (2021) dataset disposed in a (a) dotplot according to the amount of cells for each tumor type and sample type, (b) in a UMAP showing the subpopulations of mononuclear phagocytes described in their work, (c) a stacked bar plot with each subpopulation of mononuclear phagocytes contribution for each tumor type, and (d) the Pearson correlation of their mononuclear phagocytes subpopulations to the ones described in this work. Mulder and colleagues (2021) dataset disposed in a (e) dotplot according to the amount of cells for each tumor type and sample type, (f) in a UMAP showing the subpopulations of mononuclear phagocytes described in their work, (g) a stacked bar plot with each subpopulation of mononuclear phagocytes contribution for each tumor type, and (h) the Pearson correlation of their mononuclear phagocytes subpopulations to the ones described in this work. Boxplot showing a comparison of ROGUE values between macrophages (i), monocytes (j) and DC (k) populations described by this work, Cheng and colleagues (2021) work (PW1), and Mulder and colleagues (2021) work (PW2). Dashed lines represent the average score. Box indicates the range from 25th to 75th percentile, with whiskers extending to 1.5 times the interquartile range. Outliers are plotted separately, center indicates the median value. For statistical significance, we performed the Kruskall–Wallis test ($p = 0.0041$, $p < 2.2x10-16$, $p < 2.2x10-16$) followed by Wilcoxon to compare each group against "all" (i.e. base-mean). Ns non-significant; **$p < 0.01$; ****$p < 0.0001$. Source data are provided as a Source Data file.

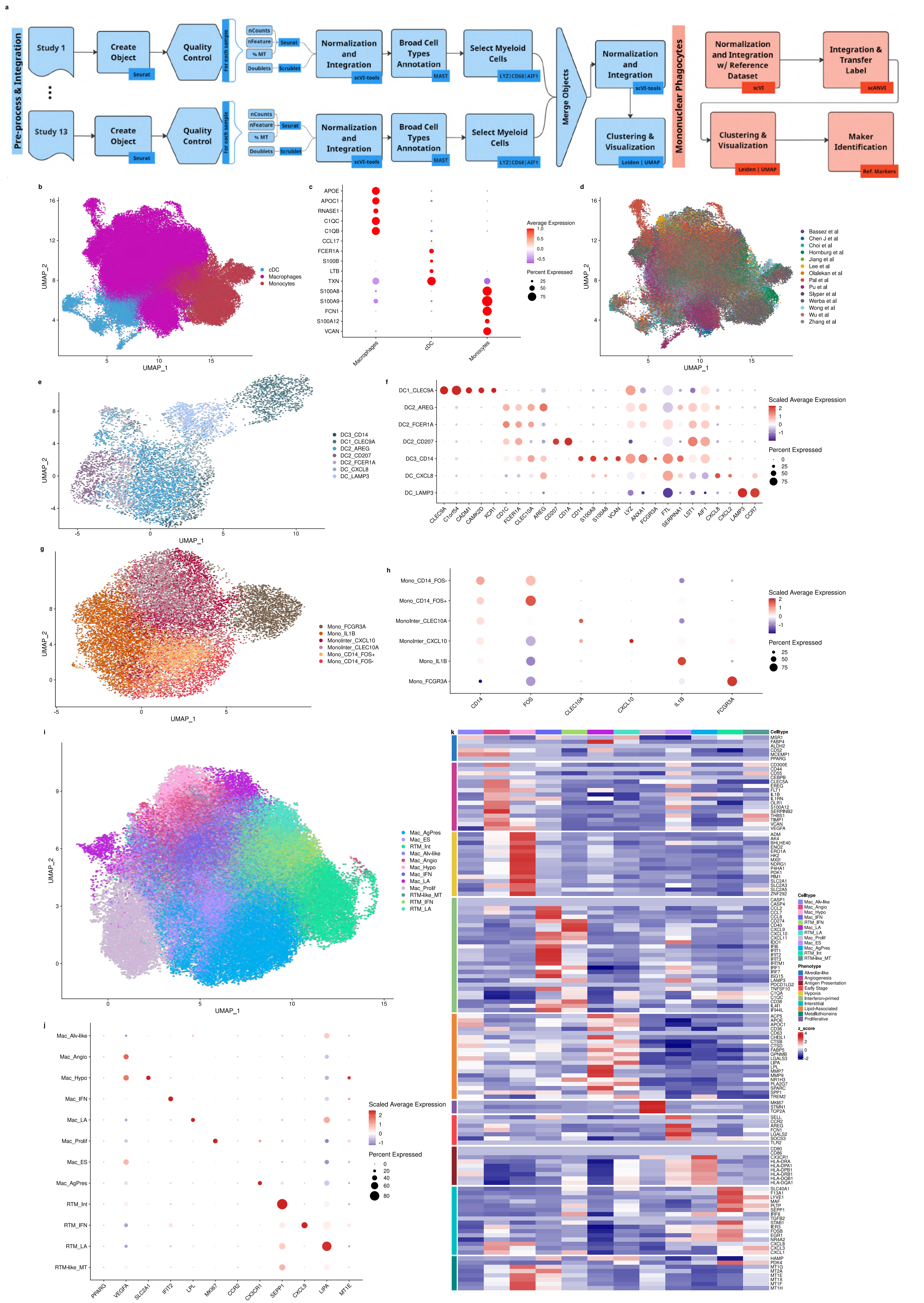
a
Pre-process & Integration
Study 1
Study 13
Create Object
Seurat
Quality Control
For each sample
nCounts
nFeature
% MT
Doublets
Scrublet
Normalization and Integration
scVI-tools
Broad Cell Types Annotation
MAST
Select Myeloid Cells
LYZ|CD68|AIF1
Merge Objects
Clustering & Visualization
Leiden | UMAP
Mononuclear Phagocytes
Normalization and Integration w/ Reference Dataset
scVI
Integration & Transfer Label
scANVI
Maker Identification
Ref. Markers
b
UMAP_1
UMAP_2
cDC
Macrophages
Monocytes
c
Average Expression
Percent Expressed
d
Bassez et al
Chen J et al
Choi et al
Hornburg et al
Jiang et al
Lee et al
Olalekan et al
Pal et al
Pu et al
Slyper et al
Werba et al
Wong et al
Wu et al
Zhang et al
e
DC3_CD14
DC1_CLEC9A
DC2_AREG
DC2_CD207
DC2_FCER1A
DC_CXCL8
DC_LAMP3
f
Scaled Average Expression
Percent Expressed
g
Mono_FCGR3A
Mono_IL1B
MonoInter_CXCL10
MonoInter_CLEC10A
Mono_CD14_FOS+
Mono_CD14_FOS-
h
i
Mac_AgPres
Mac_ES
RTM_Int
Mac_Alv-like
Mac_Angio
Mac_Hypo
Mac_IFN
Mac_LA
Mac_Prolif
RTM-like_MT
RTM_IFN
RTM_LA
j
k
Celltype
Phenotype
Alveolar-like
Angiogenesis
Antigen Presentation
Early Stage
Hypoxia
Interferon-primed
Interstitial
Lipid-Associated
Metallothioneins
Proliferative
z_score

Supplementary Figure. 10. Comprehensive validation within a myeloid cell pan-cancer atlas. (a) Workflow for the external validation analysis. (b) UMAP of 96,514 myeloid-derived cells, color-coded according to cell types and (c) Dot plot showing the top 5 DEGs expression by each myeloid-derived subpopulation. Dot size indicates the percent of expressing cells, and the dot color is the scaled average expression. (d) UMAP color-coded according to the studies that generated each dataset. (e) UMAP of DC subpopulations colored by the 7 states identified. (f) Dot plot showing the mean expression of genes related to DC subpopulations. Dot size indicates the percent of expressing cells, and the dot color the scaled average expression. (g) UMAP of monocytes subpopulations colored by the six states identified. (h) Dot plot showing the mean expression of genes related to monocytes subpopulations. Dot size indicates the percent of expressing cells, and the dot color the scaled average expression. (i) UMAP of macrophages subpopulations colored by the twelve states identified. (j) Dot plot showing the mean expression of genes related to macrophages subpopulations. Dot size indicates the percent of expressing cells, and the dot color the scaled average expression. (k) Heatmap showing the gene signature per subpopulation. The color scale represents the scaled expression of each gene. Source data are provided as a Source Data file.

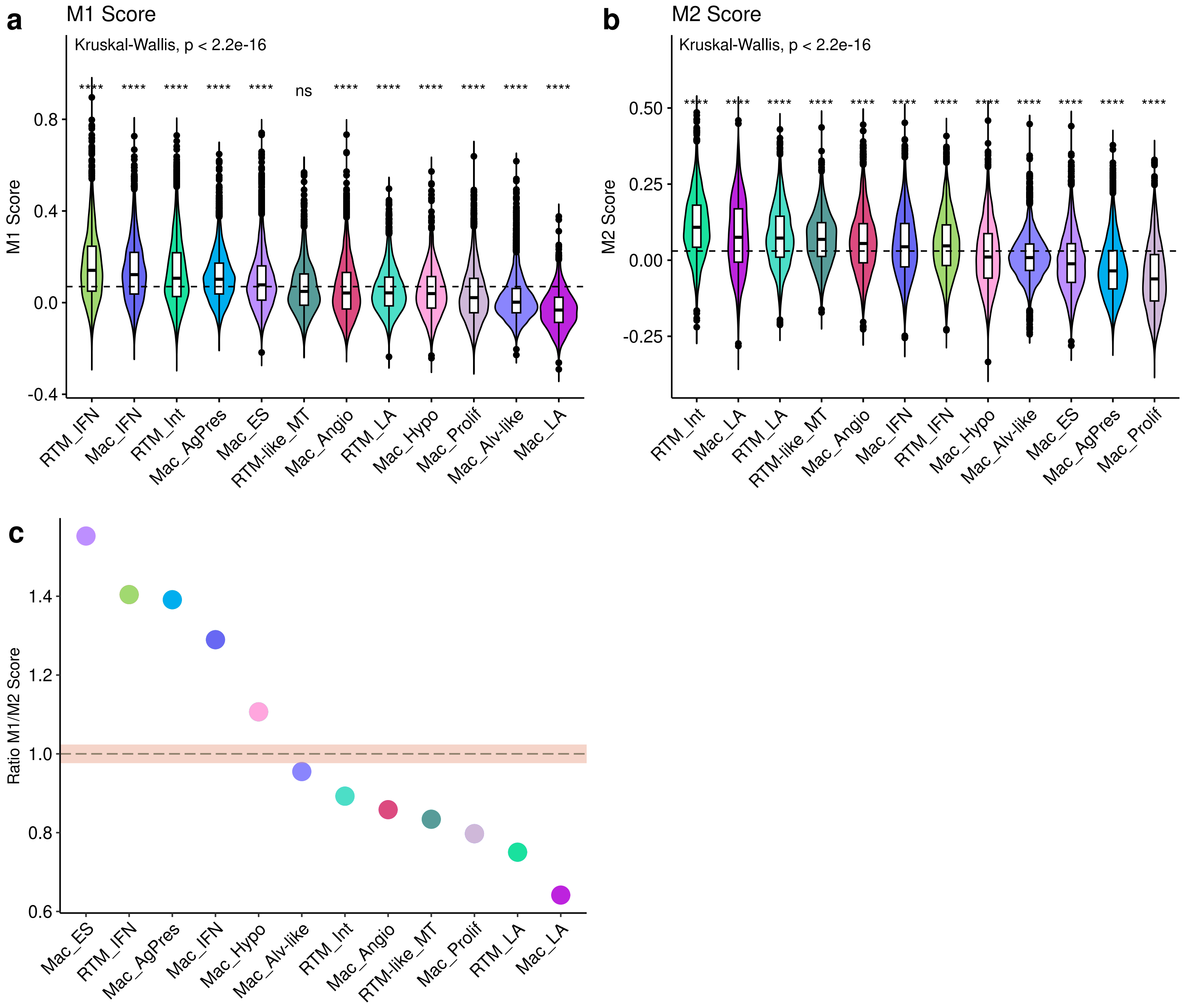

Supplementary Figure 11. Macrophages subpopulations displaying several polarization states. Violin plot showing the distribution and score signature of (a) M1 and (b) M2 markers across the Mac states. Dashed lines represent the average score. Box indicates the range from 25th to 75th percentile, with whiskers extending to 1.5 times the interquartile range. Outliers are plotted separately, center indicates the median value. For statistical significance, we performed the Kruskall–Wallis test (p < 2.2x10-16) followed by Wilcoxon to compare each group against "all" (i.e. base-mean). Ns non-significant. *p < 0.05; ****p < 0.0001. Source data are provided as a Source Data file.

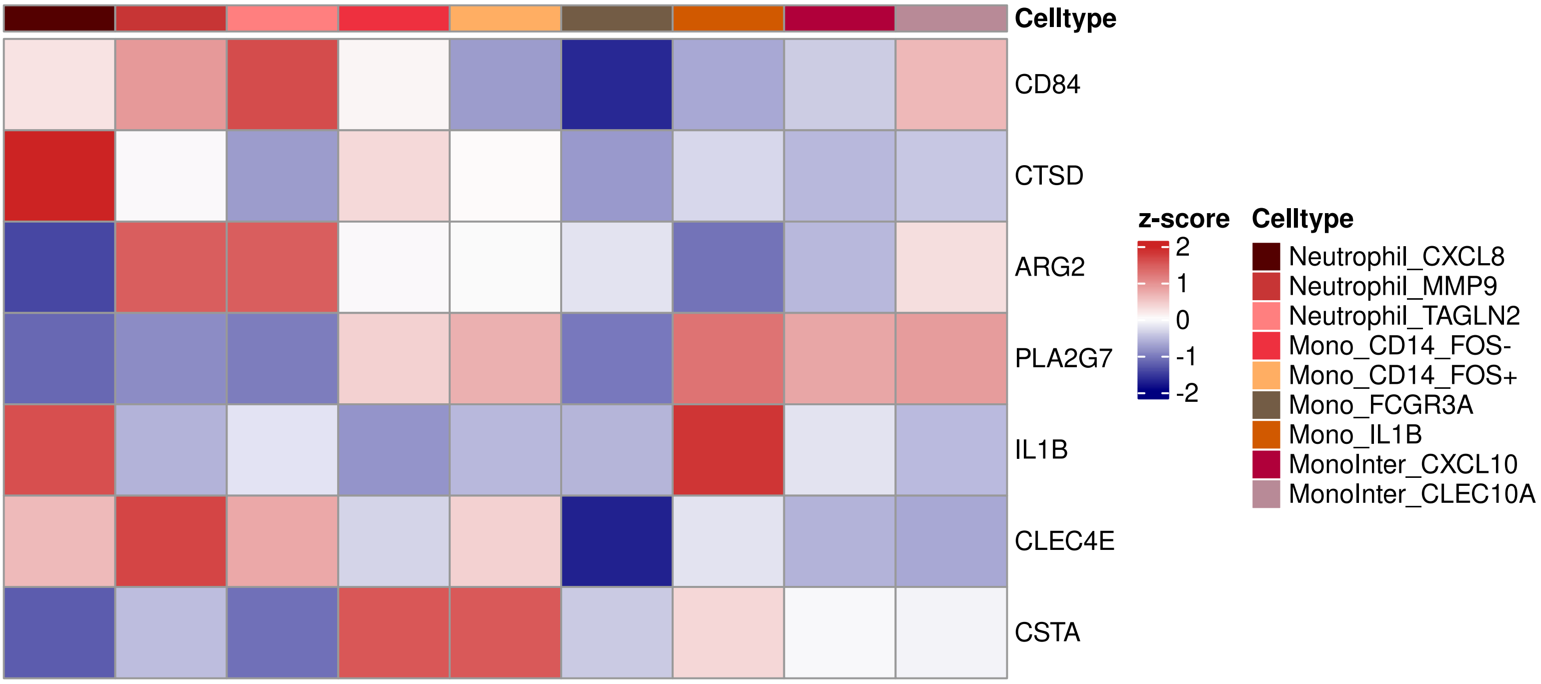

Supplementary Figure 12. Overview of the myeloid-derived suppressor cell signature across myeloid-derived subpopulations. Heatmap showing MDSC signature for neutrophils and monocytes. The color scale represents the scaled expression of each gene. Source data are provided as a Source Data file.

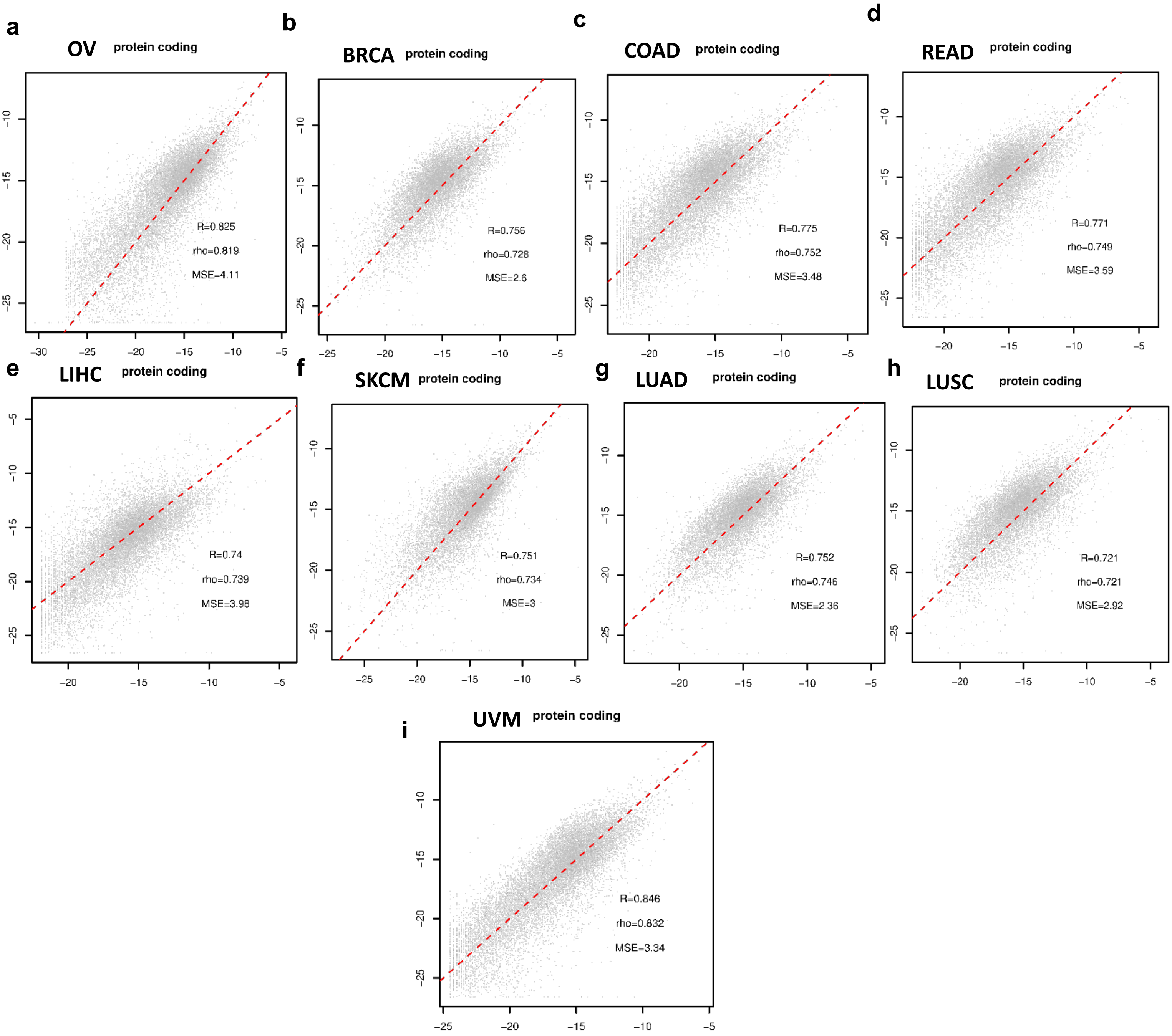

a
OV protein coding
R=0.825
rho=0.819
MSE=4.11
b
BRCA protein coding
R=0.756
rho=0.728
MSE=2.6
c
COAD protein coding
R=0.775
rho=0.752
MSE=3.48
d
READ protein coding
R=0.771
rho=0.749
MSE=3.59
e
LIHC protein coding
R=0.74
rho=0.739
MSE=3.98
f
SKCM protein coding
R=0.751
rho=0.734
MSE=3
g
LUAD protein coding
R=0.752
rho=0.746
MSE=2.36
h
LUSC protein coding
R=0.721
rho=0.721
MSE=2.92
i
UVM protein coding
R=0.846
rho=0.832
MSE=3.34

Supplementary Figure 13. Correlation between bulk RNA-seq and single-cell RNA-seq cohorts. (a-i) Pearson's correlation between bulk RNA-seq and sc-RNA-seq. R2 values were found above 0.7 for all tumor types. OV: Ovarian Carcinoma; BRCA: Breast invasive Carcinoma; COAD: Colon Adenocarcinoma; READ: Rectum Adenocarcinoma; LIHC: Liver Hepatocellular Carcinoma; SKCM: Skin Cutaneous Melanoma; LUAD: Lung Adenocarcinoma; LUSC: Lung Squamous Cell Carcinoma; UVM: Uveal Melanoma.

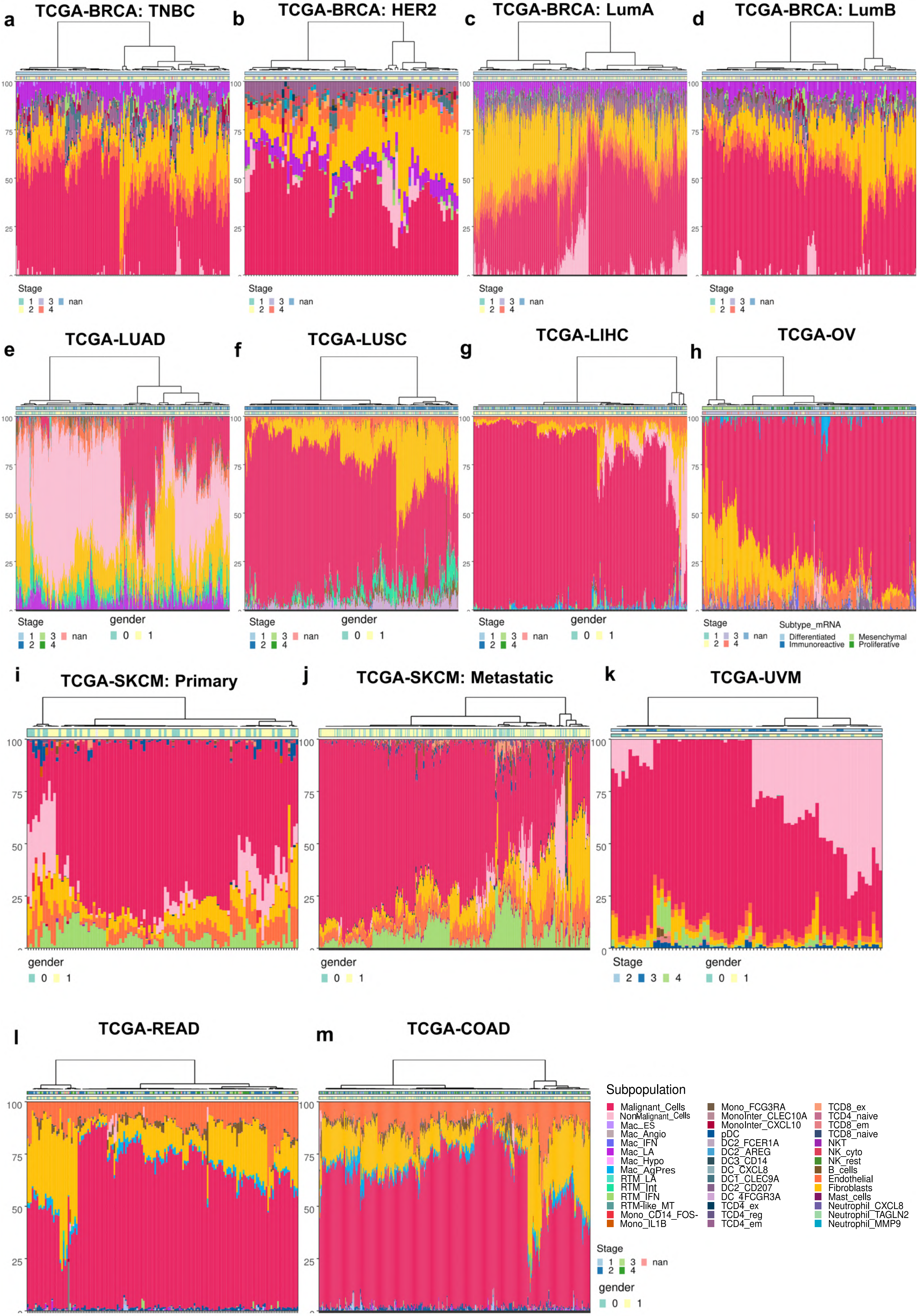

a TCGA-BRCA: TNBC
b TCGA-BRCA: HER2
c TCGA-BRCA: LumA
d TCGA-BRCA: LumB
e TCGA-LUAD
f TCGA-LUSC
g TCGA-LIHC
h TCGA-OV
i TCGA-SKCM: Primary
j TCGA-SKCM: Metastatic
k TCGA-UVM
l TCGA-READ
m TCGA-COAD
Stage
gender
Subtype_mRNA
Differentiated
Immunoreactive
Mesenchymal
Proliferative
Subpopulation
Malignant_Cells
NonMalignant_Cells
Mac_ES
Mac_Angio
Mac_IFN
Mac_LA
Mac_Hypo
Mac_AgPres
RTM_LA
RTM_Int
RTM_IFN
RTM-like_MT
Mono_CD14_FOS-
Mono_IL1B
Mono_FCG3RA
MonoInter_CLEC10A
MonoInter_CXCL10
pDC
DC2_FCER1A
DC2_AREG
DC3_CD14
DC_CXCL8
DC1_CLEC9A
DC2_CD207
DC_4FCGR3A
TCD4_ex
TCD4_reg
TCD4_em
TCD8_ex
TCD4_naive
TCD8_em
TCD8_naive
NKT
NK_cyto
NK_rest
B_cells
Endothelial
Fibroblasts
Mast_cells
Neutrophil_CXCL8
Neutrophil_TAGLN2
Neutrophil_MMP9

Supplementary Figure 14. Proportion of subpopulation cells predicted by deconvolution analysis. (a-m) Bar plot showing the predicted proportion of subpopulation cells for each tumor type. TNBC: Triple Negative Breast Cancer; TCGA: The Cancer Genome Atlas; BRCA: Breast invasive Carcinoma; OV: Ovarian Carcinoma; COAD: Colon Adenocarcinoma; READ: Rectum Adenocarcinoma; LIHC: Liver Hepatocellular Carcinoma; SKCM: Skin Cutaneous Melanoma; LUAD: Lung Adenocarcinoma; LUSC: Lung Squamous Cell Carcinoma; UVM: Uveal Melanoma.

TCGA-BRCA: TNBC: Mac_LA (TREM2 $^{+}$) - Overall Survival and Progression-Free Survival

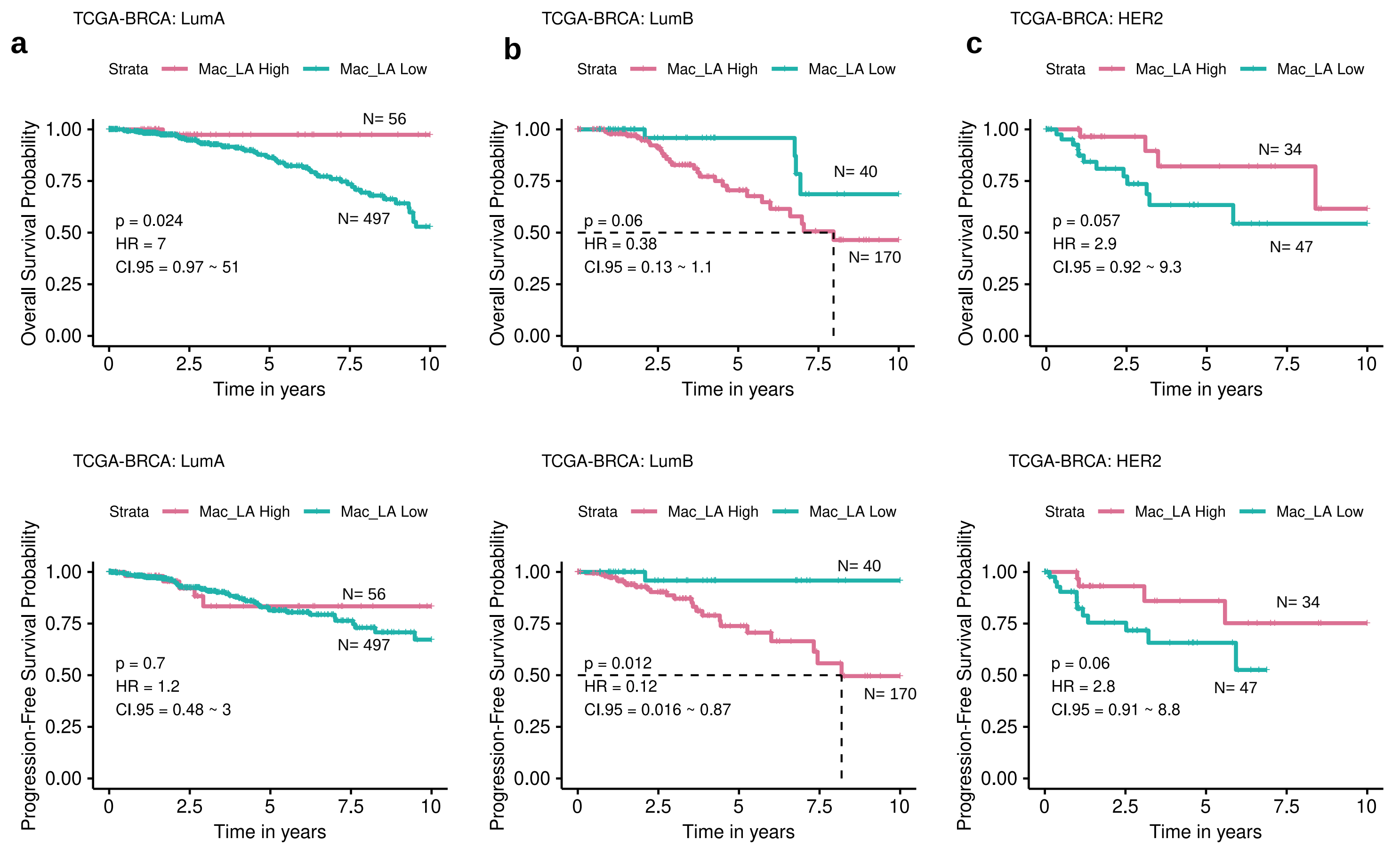

TCGA-BRCA: TREM2 $^{+}$ expression - Overall Survival and Progression-Free Survival

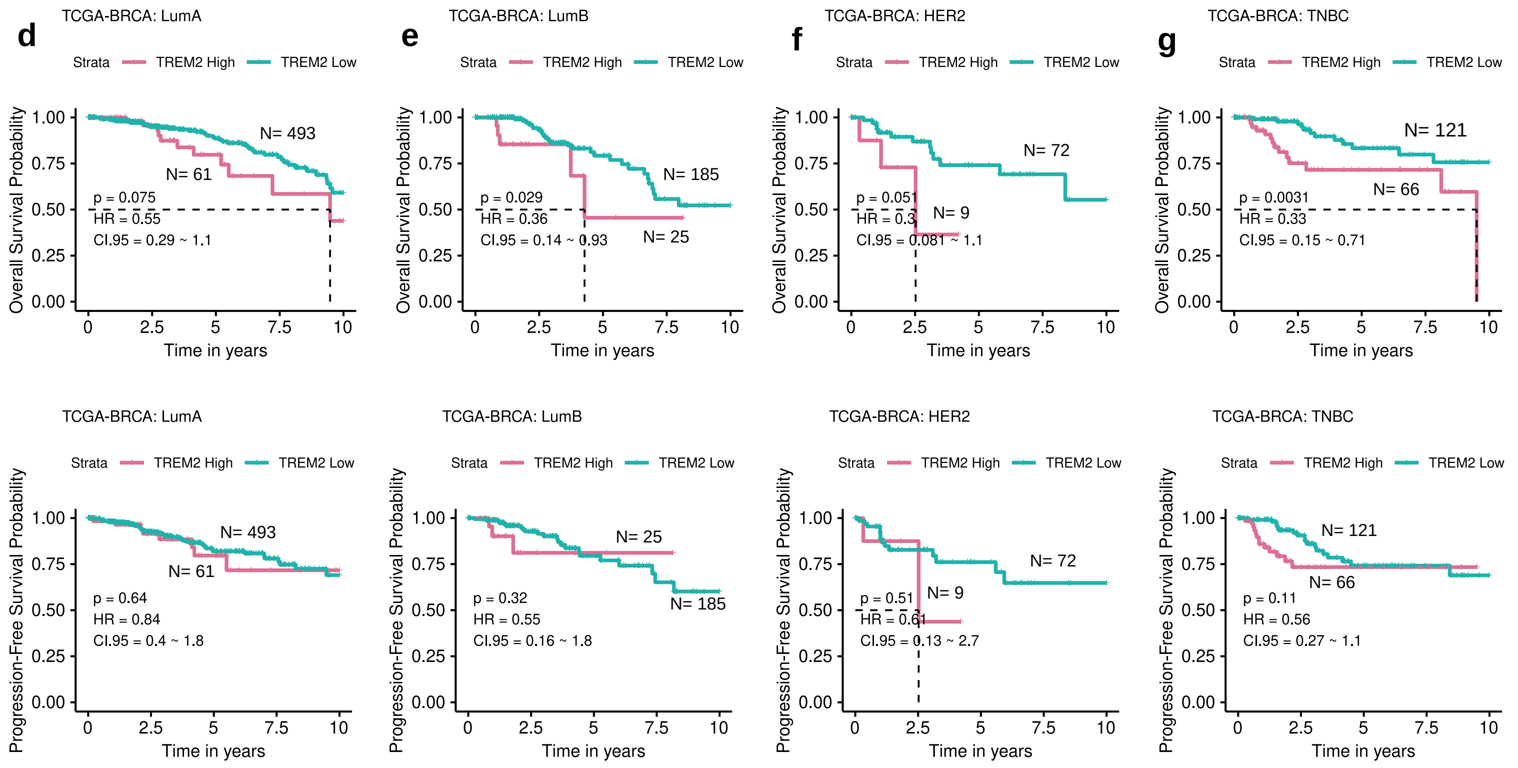

Supplementary Figure 15. Survival analysis from TCGA - breast cancer cohort. Overall and Progression-Free Survival for HIGH and LOW groups of Mac_LA (TREM2) for (a) Luminal A (n = 553; OS log-rank, p-value = 0.024; PFS log-rank, p-value = 0.7), (b) Luminal B (n = 210; OS log-rank, p-value = 0.06; PFS log-rank, p-value = 0.012), and (c) HER2 (n = 81; OS log-rank, p-value = 0.057; PFS log-rank, p-value = 0.06) subtypes; Overall and Progression-Free Survival for HIGH and LOW groups of TREM2+ expression for (d) Luminal A (n = 554; OS log-rank, p-value = 0.075; PFS log-rank, p-value = 0.64), (e) Luminal B (n = 207; OS log-rank, p-value = 0.029; PFS log-rank, p-value = 0.32), (f) HER2 (n = 81; OS log-rank, p-value = 0.051; PFS log-rank, p-value = 0.51), and TNBC (n = 187; OS log-rank, p-value = 0.0031; PFS log-rank, p-value = 0.11) subtypes from TCGA-BRCA cohort. HIGH and LOW groups were determined based on cutoff calculated using the surv_cutpoint R function. TNBC: Triple Negative Breast Cancer; TCGA: The Cancer Genome Atlas; BRCA: Breast invasive Carcinoma. Source data are provided as a Source Data file.

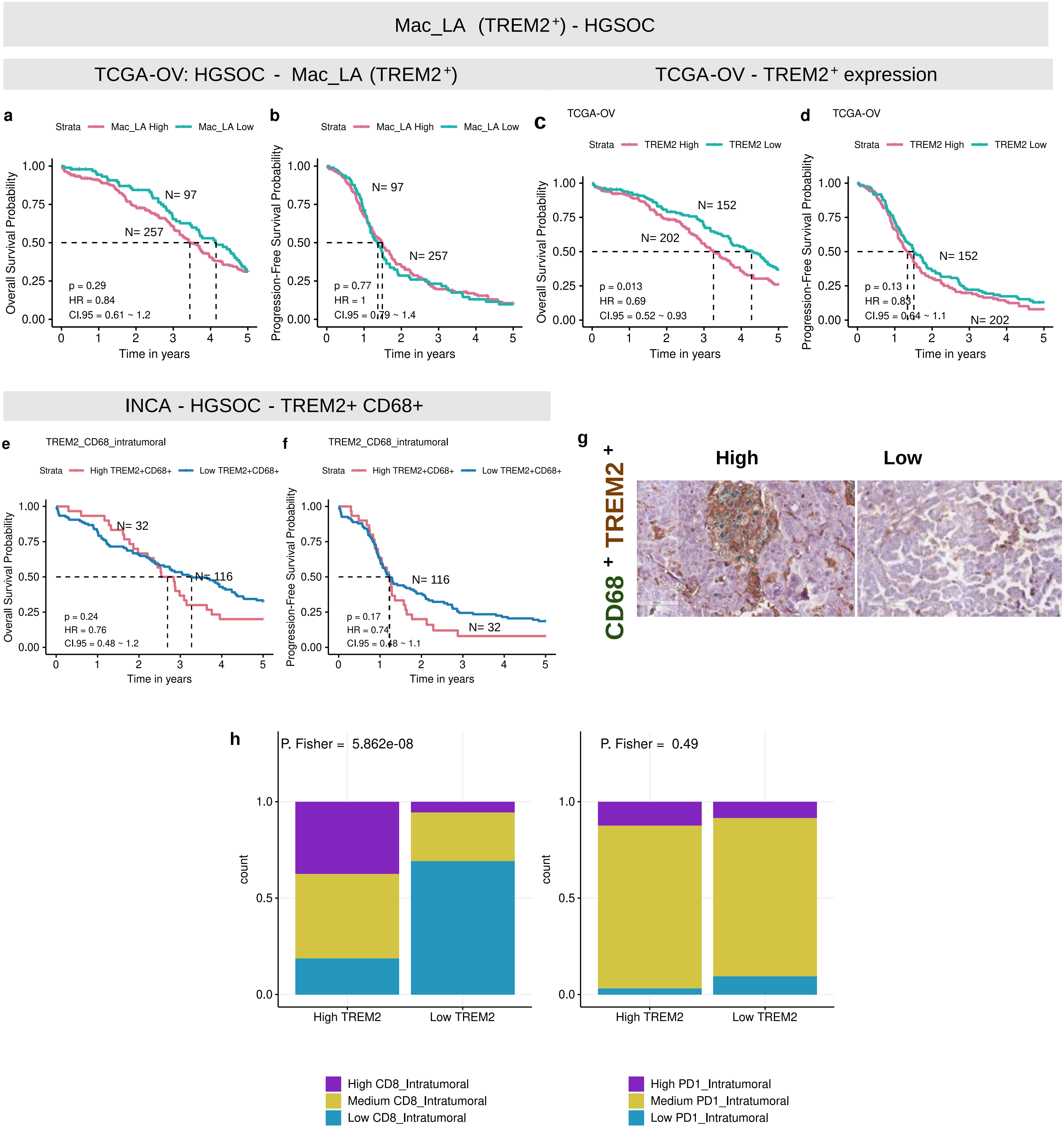

Supplementary Figure 16. Clinical impact of TREM2 on ovary tumors. (a) Overall Survival (log-rank, p-value= 0.29) for HIGH and LOW groups of Mac_LA (TREM2+) and (b) Progression-Free Survival (log-rank, p-value = 0.77) for HGSOC tumors from TCGA cohort (n = 354); (c-d) Overall Survival (log-rank, p-value = 0.013) and Progression-Free Survival (log-rank, p-value = 0.13) for HIGH and LOW groups of TREM2 gene expression (n = 354). Groups were determined based on cutoff calculated using the surv_cutpoint R function. (e) Overall Survival (log-rank, p-value = 0.24) for HIGH and LOW groups of markers TREM2 and CD68 intratumoral and (f) Progression-Free Survival (log-rank, p-value = 0.17) for HGSOC-INCA tumors (n = 148). Groups were determined based on the percentage of marked cells by a pathologist. (g) IHC representative of CD68 and TREM2 expression in HGSOC-INCA cohort. Image obtained by Aperio ImageScope v12.4.6.5003. (h) Proportions of CD8 and PD1 markers in the HIGH and LOW groups of TREM2 and CD68 population in HGSOC-INCA. TCGA: The Cancer Genome Atlas; OV: Ovarian Carcinoma; HGSOC: High Grade Serous Ovarian Carcinoma; INCA: Brazilian National Cancer Institute. Source data are provided as a Source Data file.

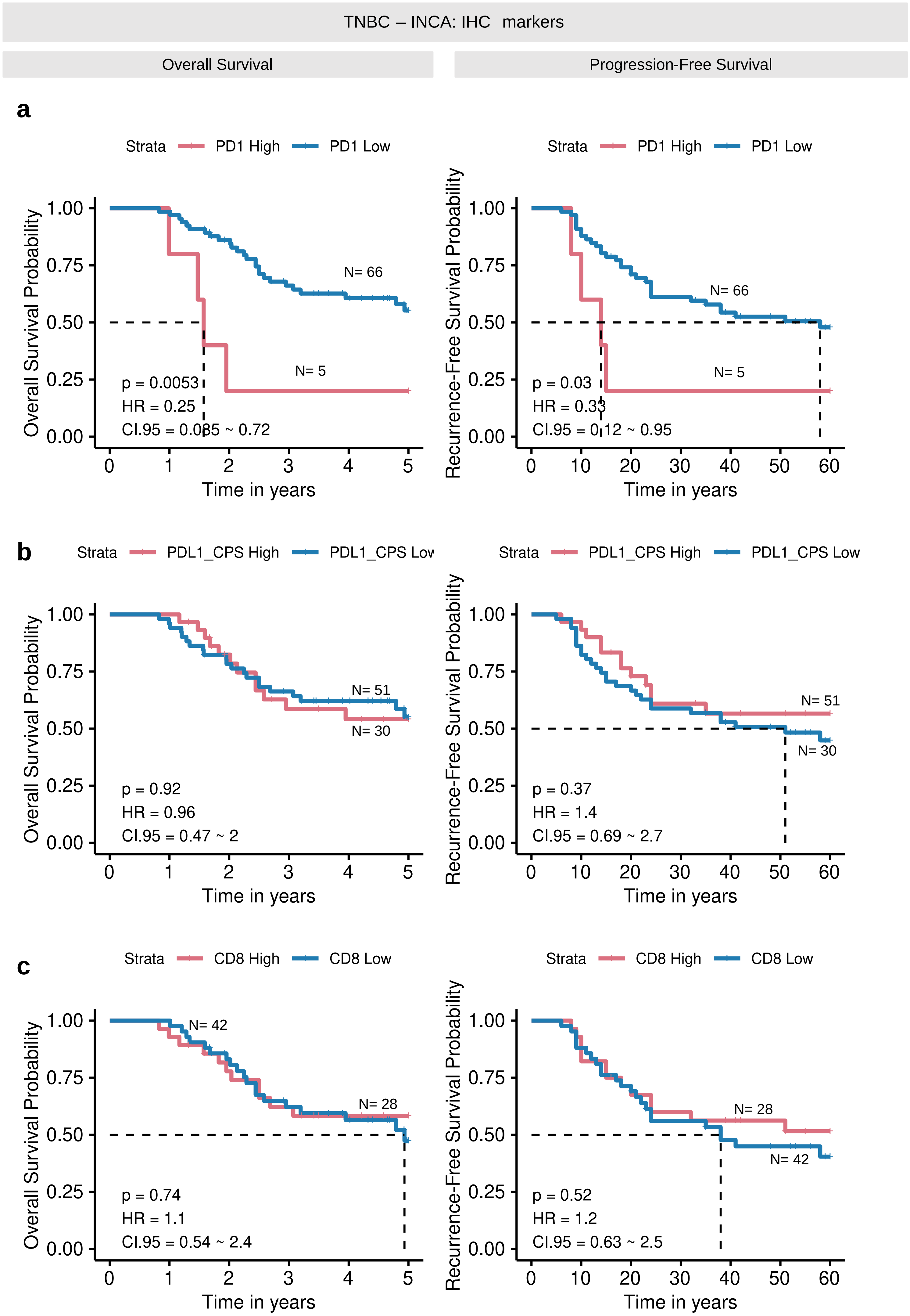
TNBC – INCA: IHC markers
Overall Survival
Progression-Free Survival
a
Strata PD1 High PD1 Low
Overall Survival Probability
Recurrence-Free Survival Probability
N= 66
N= 5
p = 0.0053
HR = 0.25
CI.95 = 0.085 ~ 0.72
p = 0.03
HR = 0.33
CI.95 = 0.12 ~ 0.95
Time in years
b
Strata PDL1_CPS High PDL1_CPS Low
N= 51
N= 30
p = 0.92
HR = 0.96
CI.95 = 0.47 ~ 2
p = 0.37
HR = 1.4
CI.95 = 0.69 ~ 2.7
c
Strata CD8 High CD8 Low
N= 42
N= 28
p = 0.74
HR = 1.1
CI.95 = 0.54 ~ 2.4
p = 0.52
HR = 1.2
CI.95 = 0.63 ~ 2.5

Supplementary Figure 17. Clinical impact of PD-1, PD-L1, and CD8 in the Brazilian TNBC cohort. (a) Overall Survival (log-rank, p-value = 0.0053) and Recurrence-Free Survival (log-rank, p-value = 0.03) for HIGH and LOW groups of marker PD-1 for TNBC-INCA subtypes (n = 71). (b) Overall Survival (log-rank, p-value = 0.92) and Recurrence-Free Survival (log-rank, p-value = 0.37) for HIGH and LOW groups of marker PD-L1 (n = 81). (c) Overall Survival (log-rank, p-value = 0.74) and Recurrence-Free Survival (log-rank, p-value = 0.52) for HIGH and LOW groups of marker CD8 (n = 70). HIGH and LOW groups for the three markers were determined based on the percentage of marked cells by a pathologist. TNBC: Triple Negative Breast Cancer; INCA: Brazilian National Cancer Institute; IHC: Immunohistochemistry. Source data are provided as a Source Data file.

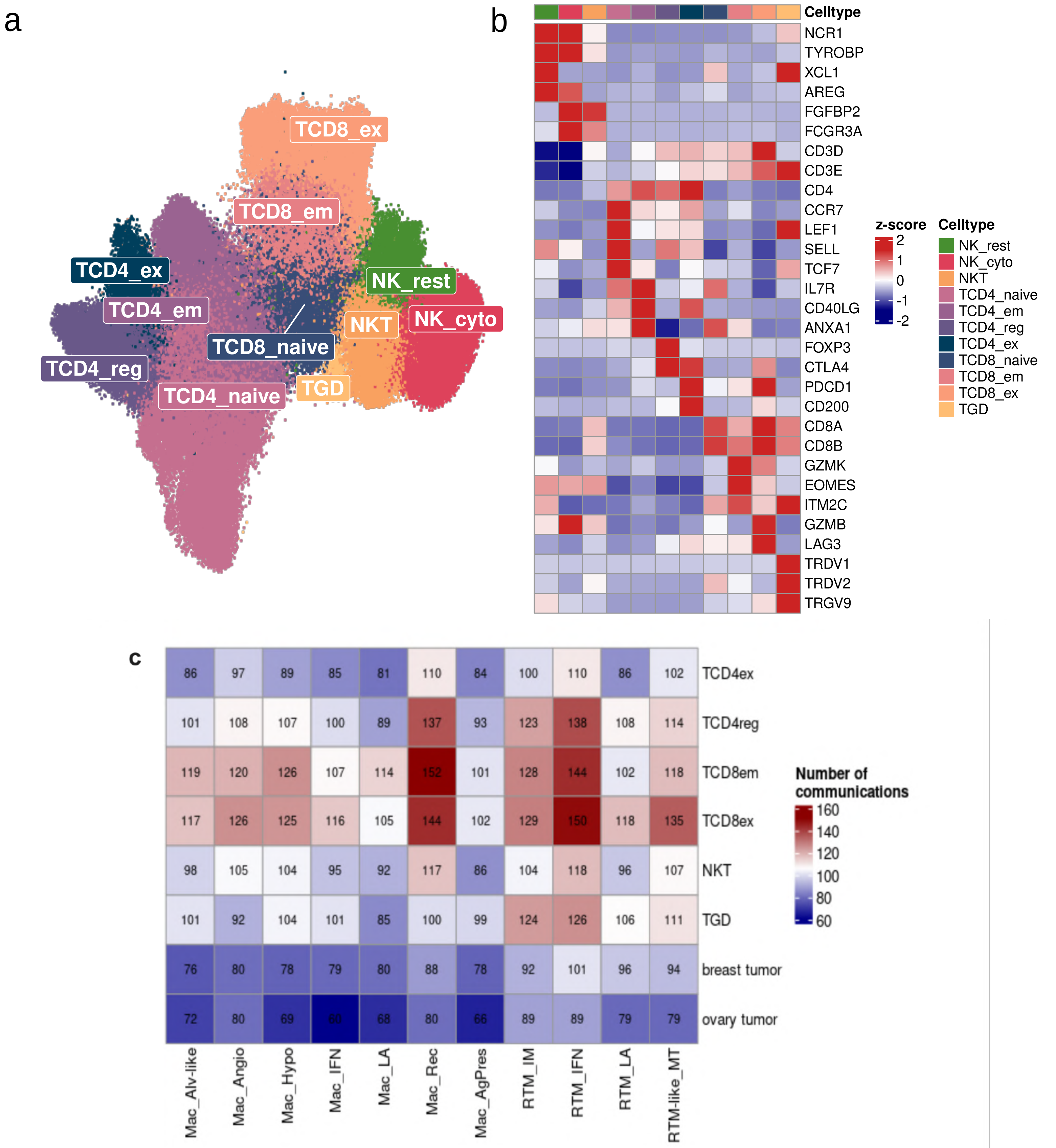

Supplementary Figure. 18. Characterization of T lymphocytes and NK cells subpopulations. (a) UMAP of T and NK subpopulations colored by the eleven states identified. (b) Heatmap showing the average expression of the canonical markers for each subpopulation.The clusters were annotated based on canonical gene markers, yielding 11 broad cell types, as mentioned: NK_rest (XCL1 and AREG); NK_cyto (GZMB, FGFBP2, and FCGR3A); NKT (CD8A, CD8B, and FGFBP2); TCD4_ex (CTLA4, PDCD1, and CD200); TCD4_em (IL7R, CD40LG and ANXA1); TCD4_naive (CCR7, LEF1, SELL and TCF7); TCD4_reg (FOXP3 and CTLA4); TCD8_em (GZMK, EMOS, and ITM2C); TCD8_ex (LAG3 and GZMB); TCD8_naive (IL7R and ANXA1); and TGD (TRDV1, TRDV2, and TRGV9). (c) Overall number of communication duals (both macrophages and T cell subpopulations expressing ligands and receptors) retrieved from CellComm algorithm. Source data are provided as a Source Data file.

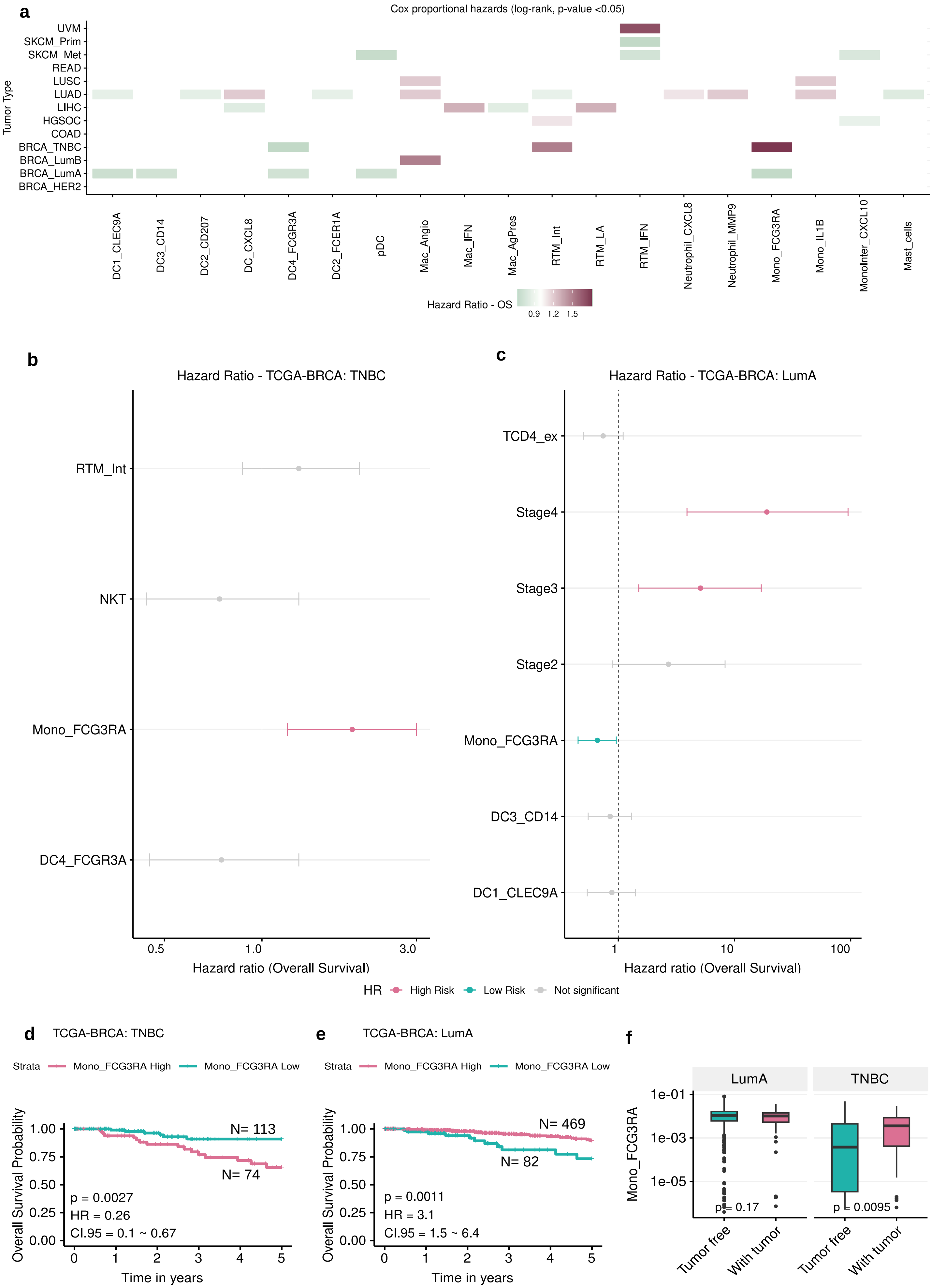

a
Cox proportional hazards (log-rank, p-value <0.05)
Tumor Type
UVM
SKCM_Prim
SKCM_Met
READ
LUSC
LUAD
LIHC
HGSOC
COAD
BRCA_TNBC
BRCA_LumB
BRCA_LumA
BRCA_HER2
DC1_CLEC9A
DC3_CD14
DC2_CD207
DC_CXCL8
DC4_FCGR3A
DC2_FCER1A
pDC
Mac_Angio
Mac_IFN
Mac_AgPres
RTM_Int
RTM_LA
RTM_IFN
Neutrophil_CXCL8
Neutrophil_MMP9
Mono_FCG3RA
Mono_IL1B
MonoInter_CXCL10
Mast_cells
Hazard Ratio - OS
0.9
1.2
1.5
b
Hazard Ratio - TCGA-BRCA: TNBC
RTM_Int
NKT
Mono_FCG3RA
DC4_FCGR3A
0.5
1.0
3.0
Hazard ratio (Overall Survival)
c
Hazard Ratio - TCGA-BRCA: LumA
TCD4_ex
Stage4
Stage3
Stage2
Mono_FCG3RA
DC3_CD14
DC1_CLEC9A
1
10
100
Hazard ratio (Overall Survival)
HR
High Risk
Low Risk
Not significant
d
TCGA-BRCA: TNBC
Strata
Mono_FCG3RA High
Mono_FCG3RA Low
N= 113
N= 74
p = 0.0027
HR = 0.26
CI.95 = 0.1 ~ 0.67
Overall Survival Probability
1.00
0.75
0.50
0.25
0.00
0
1
2
3
4
5
Time in years
e
TCGA-BRCA: LumA
Strata
Mono_FCG3RA High
Mono_FCG3RA Low
N= 469
N= 82
p = 0.0011
HR = 3.1
CI.95 = 1.5 ~ 6.4
Overall Survival Probability
1.00
0.75
0.50
0.25
0.00
0
1
2
3
4
5
Time in years
f
LumA
TNBC
Mono_FCG3RA
1e-01
1e-03
1e-05
p = 0.17
p = 0.0095
Tumor free
With tumor
Tumor free
With tumor

Supplementary Figure 19. Mono_FCGR3A is associated with the clinical outcome in BRCA tumors (TCGA). (a) Cox regression univariate analysis (p-value log-rank < 0.05) of cell subpopulations. (b) Cox regression multivariate analysis for TCGA-BRCA:TNBC cohort; (c) Cox regression multivariate analysis for TCGA-BRCA: Luminal A cohort. Light green = Low Risk (HR < 1; p < 0.05); Pink = High Risk (HR > 1, p < 0.05); Gray = Not significant. (d-e) Overall Survival for HIGH and LOW groups of Mono_FCGR3A for (d) TNBC (n = 187; log-rank, p-value= 0.0027) and (e) Luminal A (n = 551; log-rank, p-value= 0.0011) subtypes. Groups were determined based on cutoff calculated using the surv_cutpoint R function. (f) Box plots demonstrating the clinical impact of Mono_FCGR3A in relation to tumor status (tumor-free and with tumor) in both breast cancer subtypes TNBC and Luminal-A. Dashed lines represent the average score. Box indicates the range from 25th to 75th percentile, with whiskers extending to 1.5 times the interquartile range. Outliers are plotted separately, center indicates the median value. For statistical significance, we performed the Wilcoxon rank sum to compare the groups (Luminal A, p = 0.17; TNBC, p = 0.0095). TNBC: Triple Negative Breast Cancer; TCGA: The Cancer Genome Atlas; BRCA: Breast invasive Carcinoma; OV: Ovarian Carcinoma; COAD: Colon Adenocarcinoma; READ: Rectum Adenocarcinoma; LIHC: Liver Hepatocellular Carcinoma; SKCM: Skin Cutaneous Melanoma; LUAD: Lung Adenocarcinoma; LUSC: Lung Squamous Cell Carcinoma; UVM: Uveal Melanoma. Source data are provided as a Source Data file.

TCGA-BRCA - RTM_Int (FOLR2 $^{+}$) – Overall Survival and Progression-Free Survival

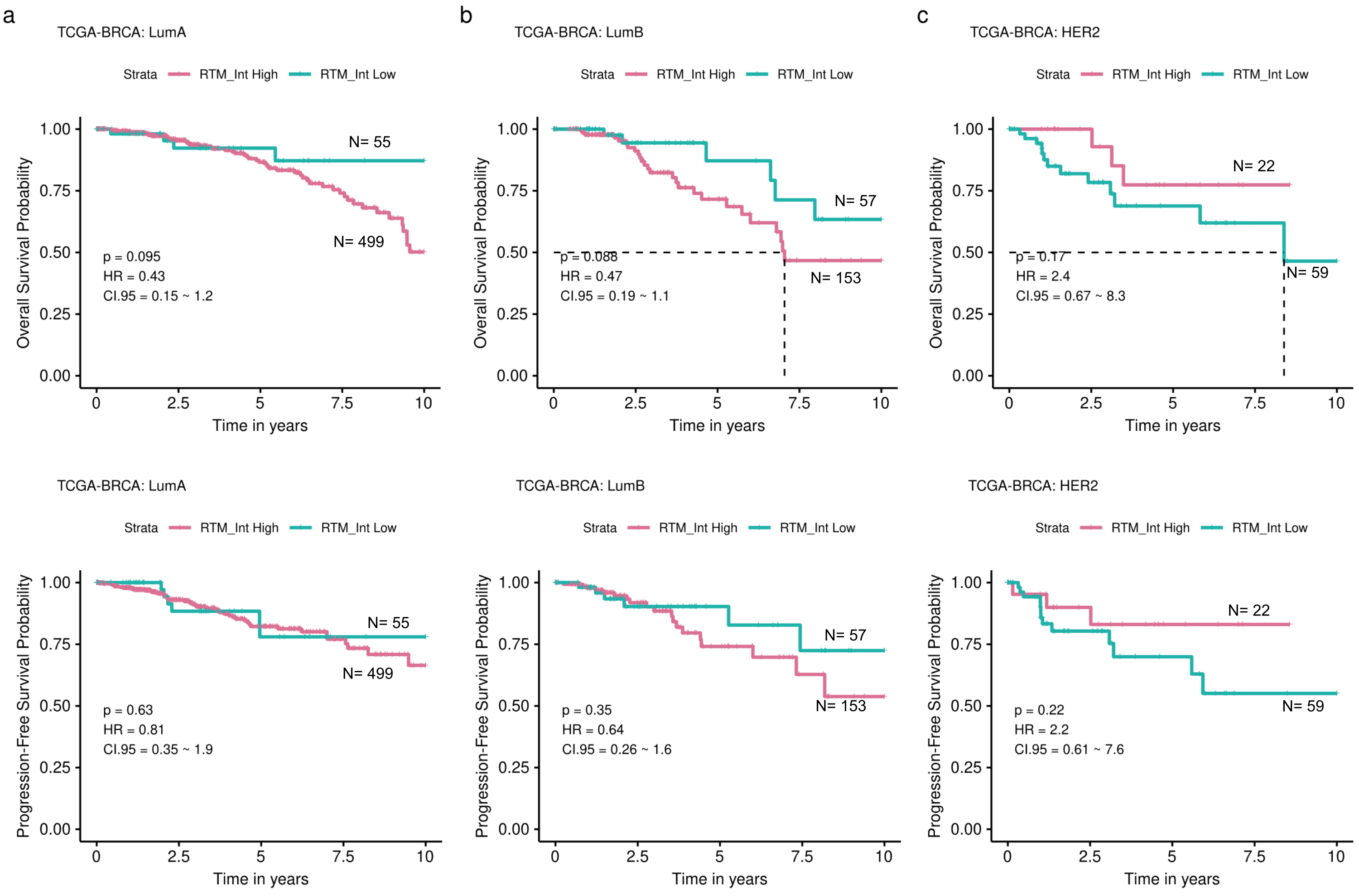

Supplementary Figure. 20. Clinical impact of RTM_Int (FOLR2+) on breast tumors. (a-c) Overall Survival for HIGH and LOW groups in Luminal A (log-rank, p-value = 0.095), Luminal B (log-rank, p-value = 0.088), and HER2 (log-rank, p-value = 0.17); Progression-Free Survival for HIGH and Low groups in Luminal A (n = 554; log-rank, p-value = 0.63), Luminal B (n = 210; log-rank, p-value = 0.35), and HER2 (n = 81; log-rank, p-value = 0.22) in BRCA subtypes. Groups were determined based on cutoff calculated using the surv_cutpoint R function. TCGA: The Cancer Genome Atlas; BRCA: Breast invasive Carcinoma. Source data are provided as a Source Data file.

METABRIC - FOLR2 + (zscore) expression - Overall Survival

a

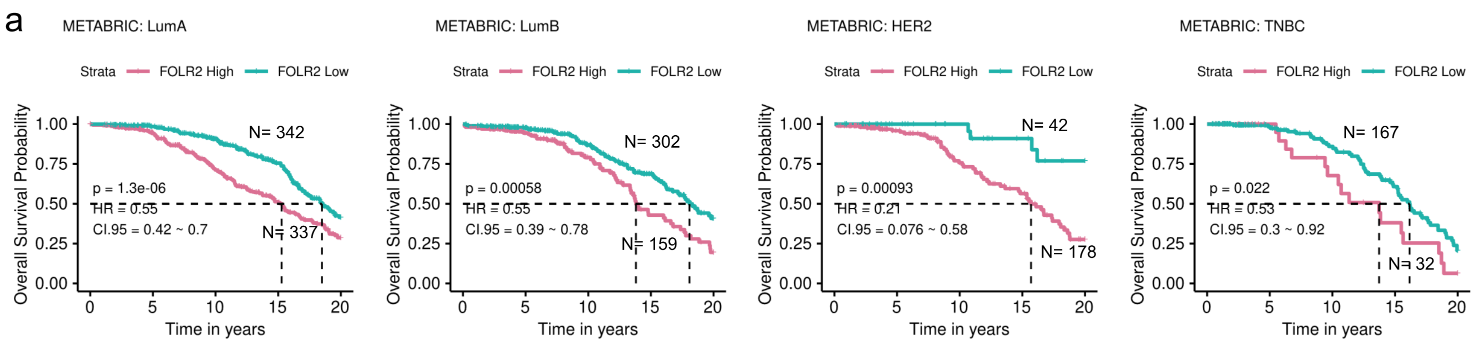

TCGA-BRCA - FOLR2 + expression. - Overall Survival and Progression-Free Survival

b

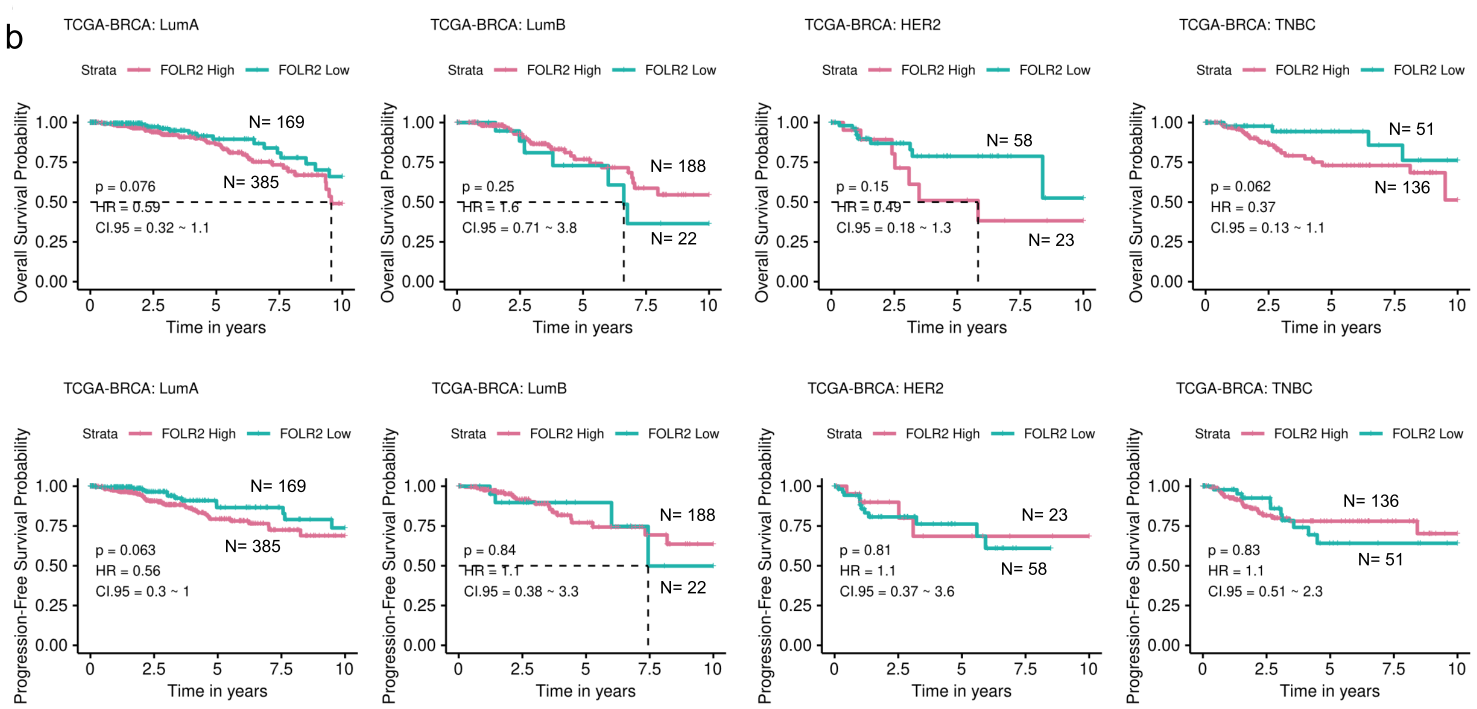

TCGA-OV: HGSOC - FOLR2 + expression - Overall Survival and Progression-Free Survival

c

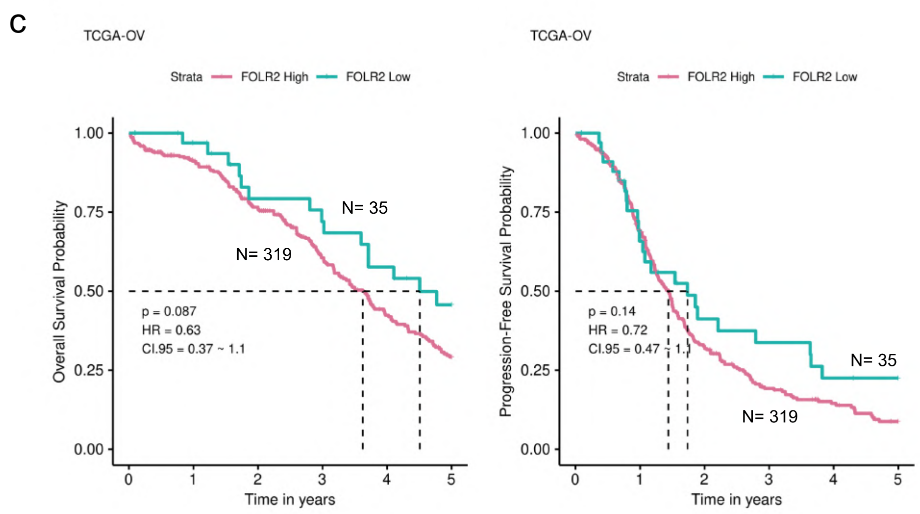

Supplementary Figure. 21. Clinical Impact of FOLR2 gene expression in METABRIC and TCGA cohorts. (a) Overall Survival for HIGH and LOW groups in Luminal A (n = 679; log-rank, p-value = 1.3e-06), Luminal B (n = 461; log-rank, p-value = 0.00058), HER2 (n = 220; log-rank, p-value = 0.00093), and TNBC (n = 199; log-rank, p-value = 0.022) subtypes from METABRIC cohort; (b) Overall Survival for HIGH and LOW groups in Luminal A (n = 554; log-rank, p-value = 0.076), Luminal B (n = 210; log-rank, p-value = 0.25), HER2 (n = 81; log-rank, p-value = 0.15), and Basal (n = 187; log-rank, p-value = 0.062) BRCA and Progression-Free Survival for HIGH and LOW groups in Luminal A (log-rank, p-value = 0.063), Luminal B (log-rank, p-value = 0.84), HER2 (log-rank, p-value = 0.81), and TNBC (log-rank, p-value = 0.83) in BRCA subtypes. (c) Overall Survival for HIGH and LOW groups (log-rank, p-value = 0.087) and Progression-Free Survival for HIGH and LOW groups in HGSOC (n = 354). Groups were determined based on cutoff calculated using the surv_cutpoint R function. TNBC: Triple Negative Breast Cancer; TCGA: The Cancer Genome Atlas; BRCA: Breast invasive Carcinoma; OV: Ovarian Carcinoma; HGSOC: High Grade Serous Ovarian Carcinoma. Source data are provided as a Source Data file.

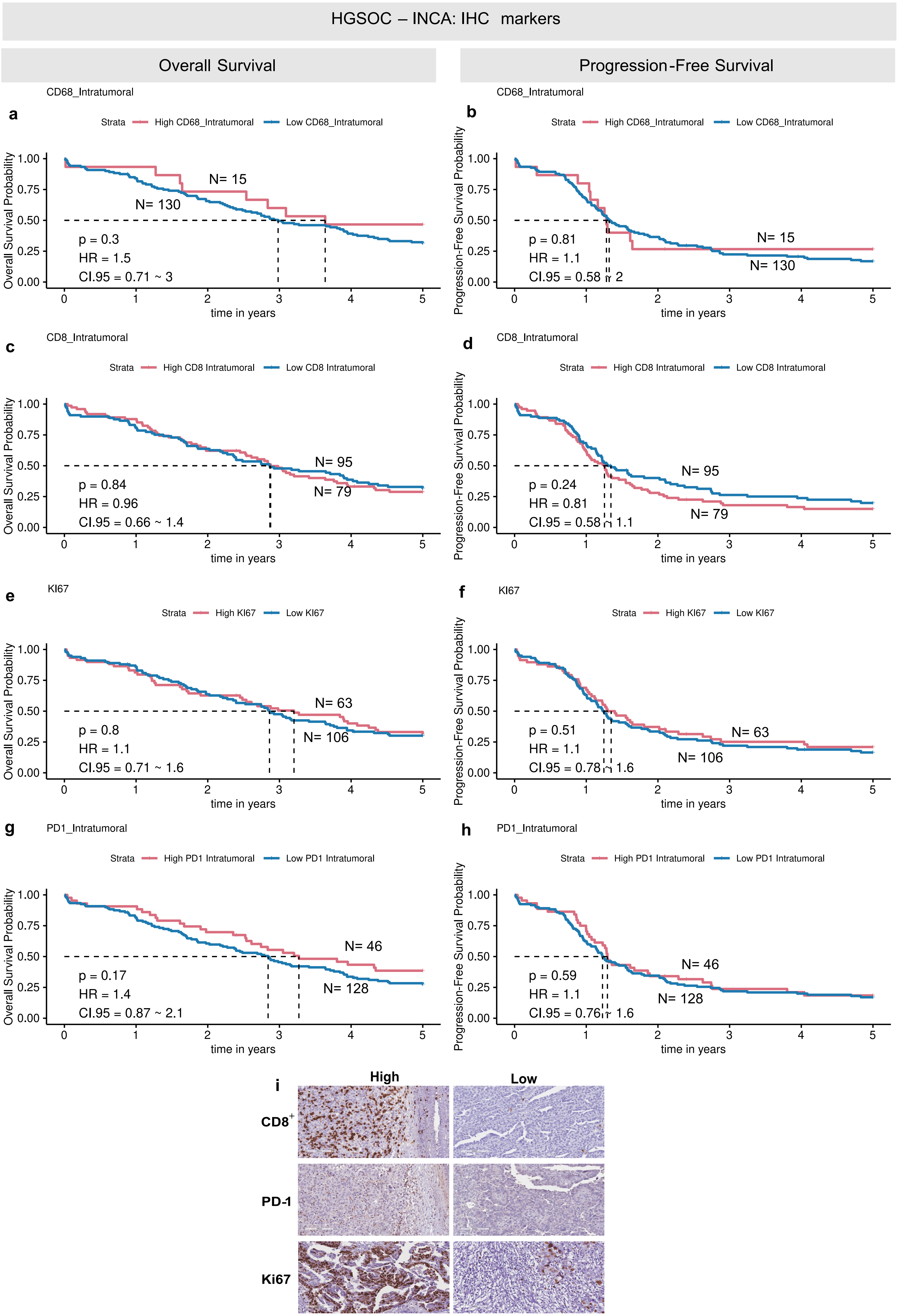
HGSOC – INCA: IHC markers
Overall Survival
Progression-Free Survival
a
CD68_Intratumoral
Strata High CD68_Intratumoral Low CD68_Intratumoral
Overall Survival Probability
N= 15
N= 130
p = 0.3
HR = 1.5
CI.95 = 0.71 ~ 3
time in years
b
CD68_Intratumoral
Strata High CD68_Intratumoral Low CD68_Intratumoral
Progression-Free Survival Probability
p = 0.81
HR = 1.1
CI.95 = 0.58 ~ 2
N= 15
N= 130
time in years
c
CD8_Intratumoral
Strata High CD8 Intratumoral Low CD8 Intratumoral
Overall Survival Probability
N= 95
N= 79
p = 0.84
HR = 0.96
CI.95 = 0.66 ~ 1.4
time in years
d
CD8_Intratumoral
Strata High CD8 Intratumoral Low CD8 Intratumoral
Progression-Free Survival Probability
N= 95
N= 79
p = 0.24
HR = 0.81
CI.95 = 0.58 ~ 1.1
time in years
e
KI67
Strata High KI67 Low KI67
Overall Survival Probability
N= 63
N= 106
p = 0.8
HR = 1.1
CI.95 = 0.71 ~ 1.6
time in years
f
KI67
Strata High KI67 Low KI67
Progression-Free Survival Probability
N= 63
N= 106
p = 0.51
HR = 1.1
CI.95 = 0.78 ~ 1.6
time in years
g
PD1_Intratumoral
Strata High PD1 Intratumoral Low PD1 Intratumoral
Overall Survival Probability
N= 46
N= 128
p = 0.17
HR = 1.4
CI.95 = 0.87 ~ 2.1
time in years
h
PD1_Intratumoral
Strata High PD1 Intratumoral Low PD1 Intratumoral
Progression-Free Survival Probability
N= 46
N= 128
p = 0.59
HR = 1.1
CI.95 = 0.76 ~ 1.6
time in years
i
High
Low
CD8+
PD-1
Ki67

Supplementary Figure. 22. Clinical impact of CD68, CD8, Ki-67, and PD-1 in the Brazilian HGSOC cohort. (a-b) Overall Survival (log-rank, p-value = 0.3) and Recurrence-Free Survival (log-rank, p-value = 0.52) for CD68 HIGH and LOW groups (n = 145). (c-d) Overall Survival (log-rank, p-value = 0.84) and Recurrence-Free Survival (log-rank, p-value = 0.24) for CD8 HIGH and LOW groups (n = 174). (e-f) Overall Survival (log-rank, p-value = 0.8) and Recurrence-Free Survival (log-rank, p-value = 0.51) for Ki67 HIGH and LOW groups (n = 169). (g-h) Overall Survival (log-rank, p-value = 0.17) and Recurrence-Free Survival (log-rank, p-value = 0.59) for PD1 HIGH and LOW groups (n = 171). Groups were determined based on the percentage of marked cells by a pathologist. (i) IHC representative of CD8, PD-1, and Ki67 expression in HGSOC-INCA cohort. Image obtained by Aperio ImageScope v12.4.6.5003. INCA: Brazilian National Cancer Institute; HGSOC: High Grade Serous Ovarian Carcinoma. Source data are provided as a Source Data file.

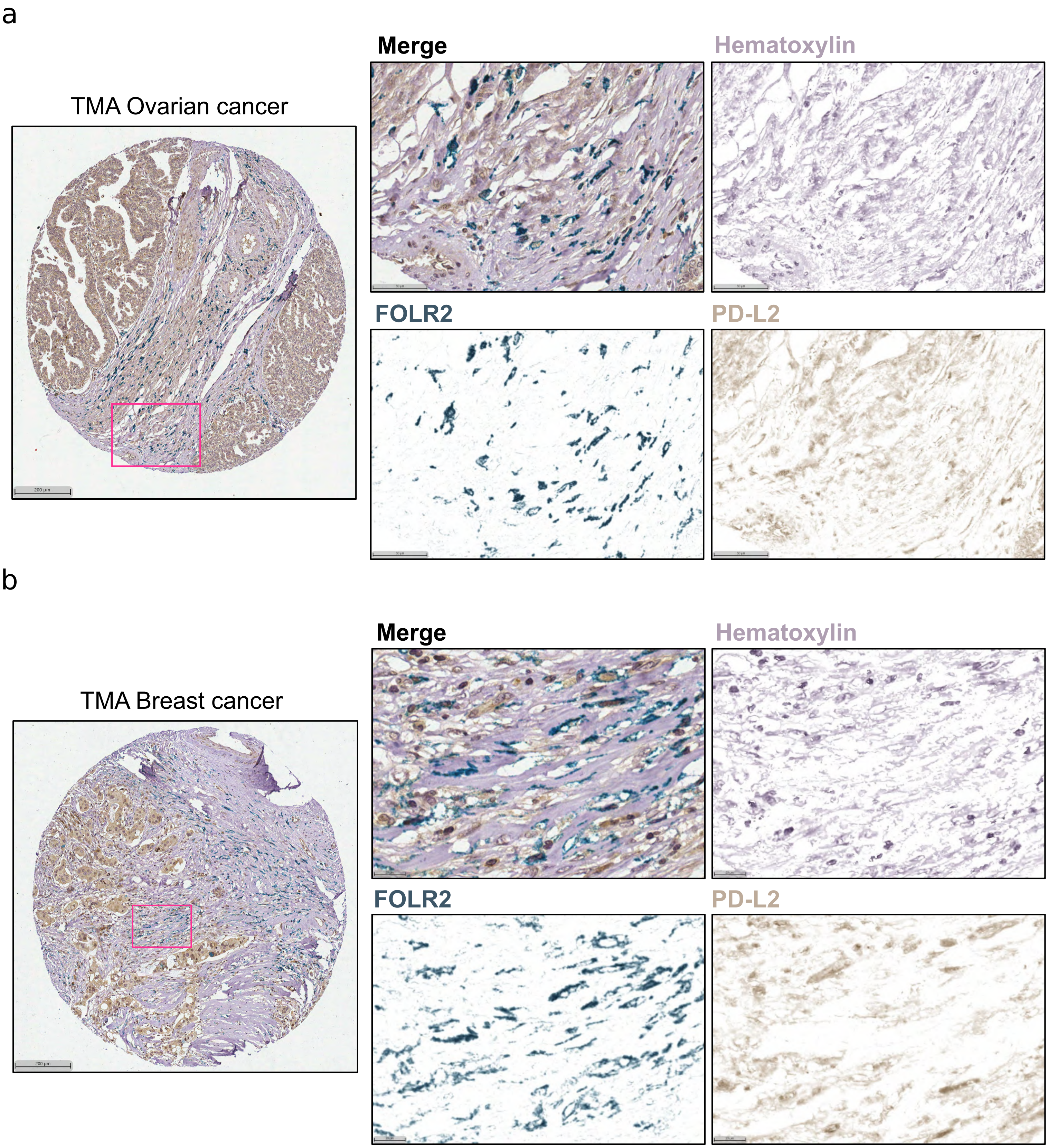

Supplementary Figure 23. Representative tumor spots from HGSOC and TNBC tissue microarrays displaying the FOLR2+ PDL-2+ phenotype. Spot images of a HGSOC-INCA patient (a) and a TNBC-INCA patient (b) with zoomed selected areas (in magenta) showing merge and computationally generated masks for hematoxylin, FOLR2 and PD-L2. Tissue microarray slides for the cohorts were analyzed using HALO software version 3.6 (Indica Labs, Albuquerque, NM, USA). TMA: Tissue microarrays; HGSOC: High Grade Serous Ovarian Carcinoma; TNBC: Triple Negative Breast Cancer.
