# Supplementary material for: Single-cell resolution characterization of myeloid-derived cell states with implication in cancer outcome
Source: Nat Commun. 2024 Jul 7;15:5694. doi: 10.1038/s41467-024-49916-4 (PMC11228020; doi:10.1038/s41467-024-49916-4)
Supplement: Supplementary file 3 — Description of Additional Supplementary Files [file 41467_2024_49916_MOESM3_ESM.pdf]

Description of Additional Supplementary Files

Supplementary Figures

Supplementary Figure 1. scRNA-seq profiling of 13 Dataset sets across seven human cancer types.

Supplementary Figure 2. Neutrophils across tumor samples.

Supplementary Figure 3. Dendritic Cells' distribution across tumor samples.

Supplementary Figure 4. Monocytes' distribution across the tumor samples.

Supplementary Figure 5. Macrophages' distribution across tumor samples.

Supplementary Figure 6. Performance metrics for different Dataset integration tools applied to the myeloid-derived cells.Supplementary

Supplementary Figure 7. Reproducibility of signatures across different platforms.

Supplementary Figure 8. Overview of the main metabolic pathways in the macrophages subpopulations.

Supplementary Figure 9. Cross-referencing new macrophage subpopulations against previous descriptions.

Supplementary Figure. 10. Comprehensive validation within a myeloid cell pan-cancer atlas.

Supplementary Figure 11. Macrophages subpopulations displaying several polarization states.

Supplementary Figure 12. Overview of the myeloid-derived suppressor cell signature across myeloid-derived subpopulations.

Supplementary Figure 13. Correlation between bulk RNA-seq and single-cell RNA-seq cohorts.

Supplementary Figure 14. Proportion of subpopulation cells predicted by deconvolution analysis.

Supplementary Figure 15. Survival analysis from TCGA - breast cancer cohort.

Supplementary Figure 16. Clinical impact of TREM2 on ovary tumors.

Supplementary Figure 17. Clinical impact of PD-1, PD-L1, and CD8 in the Brazilian TNBC cohort.

Supplementary Figure. 18. Characterization of T lymphocytes and NK cells subpopulations.

Supplementary Figure 19. Mono_FCGR3A is associated with the clinical outcome in BRCA tumors (TCGA).

Supplementary Figure. 20. Clinical impact of RTM_Int (FOLR2+) on breast tumors.

Supplementary Figure. 21. Clinical Impact of FOLR2 gene expression in METABRIC and TCGA cohorts.

Supplementary Figure. 22. Clinical impact of CD68, CD8, Ki-67, and PD-1 in the Brazilian HGSOC cohort.

Supplementary Figure 23. Representative tumor spots from HGSOC and TNBC tissue microarrays displaying the FOLR2+ PDL-2+ phenotype.

Supplementary Dataset

Supplementary Dataset 1 – Summary of Datasetsets included in the discovery cohort of the pan-cancer myeloid atlas.

Supplementary Dataset 2 - Clinical information of patients and donors involved in this study.

Supplementary Dataset 3 - Canonical gene markers used to identify cell types.

Supplementary Dataset 4 - Summary of hallmark gene signatures.

Supplementary Dataset 5 - Top 30 differentially expressed genes among clusters.

Supplementary Dataset 6 - Enrichment pathway analysis.

Supplementary Dataset 7 – Summary of datasets used for validation cohort.

Supplementary Dataset 8 - M1 and M2 gene signatures.

Supplementary Dataset 9 - Prediction of communication axes between macrophages and lymphocytes and malignant cells.

Supplementary Dataset 10 - Univariate Cox regression analysis results.

Supplementary Dataset 11 - Multivariate Cox regression analysis results.

Supplementary Dataset 12 - Description of INCA cohorts of TNBC and HGSOC.

Source Dataset

Figure 1g: Distance values between MDC subpopulations based on the average expression of 750 high variable genes.

Figure 2d: Scaled expression of each gene for dendritic cells subpopulation.

Figure 3d: Scaled expression of each gene for monocyte subpopulations.

Figure 4g: Gene signature per macrophage subpopulation.

Figure 5c-h: Signature of five hallmarks of cancer.

Figure 5i: Immunosuppressive gene signature for macrophages subpopulations.

Figure 6a: Distribution of cell types across the TCGA database.

Figure 6b: Distribution of mononuclear phagocytes states across the TCGA database.

Figure 6e: Overall Survival and Progression-Free Survival for HIGH and LOW groups of Mac_LA (TREM2) for TNBC subtypes from TCGA-BRCA cohort.

Figure 6f: Overall survival and Recurrence-Free Survival for the HIGH and LOW groups with the TREM2+ marker from the Brazilian cohort (INCA-RJ/BR) derived ImageJ analysis.

Figure 6g: Overall survival and Recurrence-Free Survival for the HIGH and LOW groups with the TREM2+ CD68+ markers from the Brazilian cohort (INCA-RJ/BR).

Figure 6i: CD8+ and PD1+ markers in the TREM2+ HIGH and LOW groups in the Brazilian TNBC cohort.

Figure 7b: Overall Survival and Progression-Free Survival for HIGH and LOW groups of RTM_Int (FOLR2) for TNBC subtypes from TCGA-BRCA cohort.

Figure 7c: Overall Survival and Progression-Free Survival for HIGH and LOW groups of RTM_Int (FOLR2) from TCGA-HGSOC cohort.

Figure 7d: Overall Survival and PFS in HIGH and LOW FOLR2+ groups from TNBC Brazilian cohort derived ImageJ analysis.

Figure 7e: Overall Survival and PFS in HIGH and LOW FOLR2+ intratumoral groups from HGSOC Brazilian cohort derived ImageJ analysis. Groups were determined by a pathologist based on percentage of marked cells.

Figure 7f: OS and PFS in HIGH and LOW FOLR2+/PDL-2+ co-staining groups for TNBC subtype from Brazilian cohort derived HALO analysis.

Figure 7g: OS and PFS in HIGH and LOW FOLR2+/PDL-2+ co-staining groups for HGSOC brazilian cohort derived HALO analysis.

Figure 7i: Proportions of CD8+ and PD1+ markers in the FOLR2+ HIGH and LOW groups in the TNBC Brazilian cohort.

Figure 7k: CD8+ and PD1+ markers proportions in HIGH and LOW FOLR2+ groups in the HGSOC Brazilian cohort.

Supp Figure 1i,j: Purity of (i) broad cell type clusters and (j) macrophages post-scVI integration, evaluated using ROGUE scores.

Supp Figure 2d: markers' genes across neutrophils subpopulation.

Supp Figure 4d: the inflammation score among Mono subpopulations.

Supp Figure 7a: the gene signature per subpopulation for 10x technology Dataset.

Supp Figure 7b: the gene signature per subpopulation for inDrop technology Dataset.

Supp Figure 8: the main metabolism signature for each Mac states.

Supp Figure 9d: Pearson correlation of their mononuclear phagocytes subpopulations to the ones described in this work with Mulder and colleagues (2021) Datasetset.

Supp Figure 9h: Pearson correlation of their mononuclear phagocytes subpopulations to the ones described in this work with Cheng and colleagues Datasetset.

Supp Figure 10k: Comprehensive validation within a myeloid cell pan-cancer atlas - the gene signature per macrophage subpopulation.

Supp Figure 11: Macrophages subpopulations displaying several polarization states.

Supp Figure 12: Overview of the myeloid-derived suppressor cell signature across myeloid-derived subpopulations.

Supp Figure 15: Survival analysis from TCGA - breast cancer cohort.

Supp Figure 16: Clinical impact of TREM2 on ovary tumors. (a) Overall Survival for HIGH and LOW groups of Mac_LA (TREM2+) and (b) Progression-Free Survival for HGSOC tumors from TCGA cohort; (c-d) Overall Survival and Progression-Free Survival for HIGH and LOW groups of TREM2 gene expression.

Supp Figure 16e-h: Overall Survival for HIGH and LOW groups of markers TREM2 and CD68 intratumoral and (f) Progression-Free Survival for HGSOC tumors from Brazilian cohort. Groups were determined based on the percentage of marked cells by a pathologist. (h) Proportions of CD8 and PD1 markers in the HIGH and LOW groups of TREM2 and CD68 population in the Brazilian HGSOC cohort.

Supp Figure 17: Clinical impact of PD-1, PD-L1, and CD8 in the Brazilian TNBC cohort.

Supp Figure 18: Average expression of canonical markers for each T cell subpopulation.

Supp Figure 19: Overall Survival for HIGH and LOW groups of Mono_FCGR3A for (d) TNBC and (e) Luminal A subtypes. Groups were determined based on cutoff calculated using the surv_cutpoint R function.

Supp Figure 20: Clinical impact of RTM_Int (FOLR2+) on breast tumors.

Supp Figure 21a: Overall Survival for HIGH and LOW groups in Luminal A, Luminal B, HER2, and TNBC subtypes from METABRIC cohort.

Supp Figure 21b: Overall Survival for HIGH and LOW groups in Luminal A, Luminal B, HER2, and BasalBRCA and Progression-Free Survival for HIGH and LOW groups in Luminal A, Luminal B , HER2, and TNBC in BRCA subtypes.

Supp Figure 21c: Overall Survival for HIGH and LOW groups and Progression-Free Survival for HIGH and LOW groups in TCGA-HGSOC for FOLR2 expression.

Supp Figure 22: Clinical impact of CD68, CD8, Ki-67, and PD-1 in the Brazilian HGSOC cohort.
