# Supplementary material for: Single-cell resolution characterization of myeloid-derived cell states with implication in cancer outcome
Source: Nat Commun. 2024 Jul 7;15:5694. doi: 10.1038/s41467-024-49916-4 (PMC11228020; doi:10.1038/s41467-024-49916-4)
Supplement: Supplementary file 16 — Reporting Summary [file 41467_2024_49916_MOESM16_ESM.pdf]

nature portfolio

Corresponding author(s): Mariana Boroni

Last updated by author(s): May 18, 2024

Reporting Summary

Nature Portfolio wishes to improve the reproducibility of the work that we publish. This form provides structure for consistency and transparency in reporting. For further information on Nature Portfolio policies, see our Editorial Policies and the Editorial Policy Checklist.

Statistics

For all statistical analyses, confirm that the following items are present in the figure legend, table legend, main text, or Methods section.

n/a	Confirmed	
☐	☒	The exact sample size (*n*) for each experimental group/condition, given as a discrete number and unit of measurement
☐	☒	A statement on whether measurements were taken from distinct samples or whether the same sample was measured repeatedly
☐	☒	The statistical test(s) used AND whether they are one- or two-sided<br>*Only common tests should be described solely by name; describe more complex techniques in the Methods section.*
☐	☒	A description of all covariates tested
☐	☒	A description of any assumptions or corrections, such as tests of normality and adjustment for multiple comparisons
☐	☒	A full description of the statistical parameters including central tendency (e.g. means) or other basic estimates (e.g. regression coefficient) AND variation (e.g. standard deviation) or associated estimates of uncertainty (e.g. confidence intervals)
☐	☒	For null hypothesis testing, the test statistic (e.g. *F*, *t*, *r*) with confidence intervals, effect sizes, degrees of freedom and *P* value noted<br>*Give P values as exact values whenever suitable.*
☐	☒	For Bayesian analysis, information on the choice of priors and Markov chain Monte Carlo settings
☐	☒	For hierarchical and complex designs, identification of the appropriate level for tests and full reporting of outcomes
☐	☒	Estimates of effect sizes (e.g. Cohen's *d*, Pearson's *r*), indicating how they were calculated

Our web collection on statistics for biologists contains articles on many of the points above.

Software and code

Policy information about availability of computer code

Data collection	Pre-processed scRNA-Seq data from patients with breast cancer (GSE114727), hepatocellular carcinoma (GSE140228, GSE125449), lung cancer (GSE127465), melanoma (GSE115979), ovarian cancer (GSE154600, GSE72056), uveal melanoma (GSE139829), skin (GSE130973) and metastasis from uveal melanoma and lung (GSE158803) samples were obtained from the public repository Gene Expression Omnibus (GEO) using the GEOquery Bioconductor package. Additionally, datasets from lung, ovarian, and breast cancer were downloaded from Qian et al., on its platform (blueprint.lambrechtslab.org/). We downloaded datasets from the Human Lung Cell Atlas project on the Synapse platform (SYN21560407). Finally, the dataset containing PBMCs (10x Genomics standard) was downloaded from the company platform (support.10xgenomics.com/single-cell-gene-expression/datasets/1.1.0/pbmc3k). Pre-processed single-cell RNA sequencing (scRNA-Seq) data from various cancer types were acquired (Supplementary Table 6) from the GEO using the GEOquery Bioconductor package. The data encompassed head and neck cancer (GSE181919), renal cell carcinoma (GSE159115), pancreatic adenocarcinoma (GSE205013), prostate cancer (GSE185344), endometrial cancer (GSE251923), and multiple datasets for breast cancer (GSE176078, GSE161529), ovarian cancer (GSE140819, GSE147082), gastric cancer (GSE163558), and thyroid cancer (GSE184362). Additional datasets for colorectal cancer (E-MTAB-8410) and more datasets for breast (EGAD00001006608) and ovarian cancers (EGAS00001004935) were retrieved from alternative databases. Bulk RNA-Seq are obtained from TCGA and METABRIC. Regarding immunohistochemistry analysis: for the ovary cohort, 193 all women diagnosed with HGSOC at Brazilian National Cancer Institute (INCA) between 2001 and 2017, regardless of adjuvant or neoadjuvant treatment, were included in our study. The TNBC cohort, previously described by our collaborators, consisted of 112 women who underwent neoadjuvant chemotherapy and thereafter curative surgery between 2010 and 2014.
Data analysis	The following libraries were used in this work:<br>Scrublet package v0.2.3, Scanpy v1.7.2, scVI v0.6.8, scvi-tools v0.19.0, MAST v1.16.0, Seurat V4.0, Scanorama v1.7.9, Harmony v.1.2.0, BBKNN v.0.2.0, FastMNN v4, inferCNVpy software v0.4.0, ggplot2 v3.4.4, Compass v.0.9.7.1, Monocle 3 (1.3.1), clusterProfiler R package (v3.0), CellComm algorithm, TCGAbiolinks v2.28, BayesPrism package (v2.0), HALO software version 3.6, ImageJ Plugin, Survival (v3.5), Survminer

(v0.4.9)

For manuscripts utilizing custom algorithms or software that are central to the research but not yet described in published literature, software must be made available to editors and reviewers. We strongly encourage code deposition in a community repository (e.g. GitHub). See the Nature Portfolio guidelines for submitting code & software for further information.

Data

Policy information about availability of data

All manuscripts must include a data availability statement. This statement should provide the following information, where applicable:

- Accession codes, unique identifiers, or web links for publicly available datasets
- A description of any restrictions on data availability
- For clinical datasets or third party data, please ensure that the statement adheres to our policy

The published datasets related to the main analysis can be accessed under GEO accession numbers: GSE114727 [www.ncbi.nlm.nih.gov/geo/query/acc.cgi?acc=GSE114727], GSE140228 [www.ncbi.nlm.nih.gov/geo/query/acc.cgi?acc=GSE140228], GSE125449 [www.ncbi.nlm.nih.gov/geo/query/acc.cgi?acc=GSE125449], GSE115978 [www.ncbi.nlm.nih.gov/geo/query/acc.cgi?acc=GSE115978], GSE154600 [www.ncbi.nlm.nih.gov/geo/query/acc.cgi?acc=GSE154600], GSE130973 [www.ncbi.nlm.nih.gov/geo/query/acc.cgi?acc=GSE130973], GSE139829 [www.ncbi.nlm.nih.gov/geo/query/acc.cgi?acc=GSE139829], GSE158803 [www.ncbi.nlm.nih.gov/geo/query/acc.cgi?acc=GSE158803]; URLs for additional data are www.synapse.org/#!Synapse:syn21041850/wiki/600865 and lambrechtslab.sites.vib.be/en/dataaccess, as well as support.10xgenomics.com/single-cell-gene-expression/datasets/1.1.0/pbmc3k. The public datasets related to the validation analysis can be accessed under GEO accession numbers: GSE181919 [www.ncbi.nlm.nih.gov/geo/query/acc.cgi?acc=GSE181919], GSE159115 [www.ncbi.nlm.nih.gov/geo/query/acc.cgi?acc=GSE159115], GSE205013 [www.ncbi.nlm.nih.gov/geo/query/acc.cgi?acc=GSE205013], GSE251923 [www.ncbi.nlm.nih.gov/geo/query/acc.cgi?acc=GSE251923], GSE185344 [www.ncbi.nlm.nih.gov/geo/query/acc.cgi?acc=GSE185344], GSE176078 [www.ncbi.nlm.nih.gov/geo/query/acc.cgi?acc=GSE176078], GSE161529 [www.ncbi.nlm.nih.gov/geo/query/acc.cgi?acc=GSE161529], GSE140819 [www.ncbi.nlm.nih.gov/geo/query/acc.cgi?acc=GSE140819], GSE147082 [www.ncbi.nlm.nih.gov/geo/query/acc.cgi?acc=GSE147082], GSE163558 [www.ncbi.nlm.nih.gov/geo/query/acc.cgi?acc=GSE163558], GSE184362 [www.ncbi.nlm.nih.gov/geo/query/acc.cgi?acc=GSE184362]; European Genome-phenome Archive study IDs EGAD00001006608 [ega-archive.org/studies/EGAS00001004809], EGAS00001004935 [ega-archive.org/studies/EGAS00001004935] and ArrayExpress E-MTAB-8410 (see also Supplementary Data 1, and 6). The integrated dataset is publicly available and can be downloaded via CELL×GENE | Collections through the following link: cellxgene.cziscience.com/collections/3f7c572c-cd73-4b51-a313-207c7f20f188.

Research involving human participants, their data, or biological material

Policy information about studies with human participants or human data. See also policy information about sex, gender (identity/presentation), and sexual orientation and race, ethnicity and racism.

Reporting on sex and gender	Regarding the Immunohistochemistry analysis cohort, patients from the National Cancer Institute in Rio de Janeiro-Brazil (INCA cohort) who has donated samples to the National Bank Tumor were identified to participate in this study. We analyzed two cohorts: the ovary cohort comprises 193 all women, and the TNBC cohort comprises 112 women, listed in Supplementary Table 11. The integrated single-cell data comprises 142 participants, all clinical data used in this work have been listed in Supplementary Tables 1 and 6.
Reporting on race, ethnicity, or other socially relevant groupings	All enrolled women from INCA cohort in this study are Latin Americans from Brazil.
Population characteristics	This retrospective study was approved by the Ethics in Human Research Committee of the Brazilian National Cancer Institute (INCA), Rio de Janeiro, Brazil, and was conducted following the Good Clinical Practice Guidelines. For the ovary cohort, 193 all women (age range from 23-84 years old) diagnosed with HGSOC at INCA between 2001 and 2017, regardless of adjuvant or neoadjuvant treatment, and tumor staging, were included in our study . The TNBC cohort, previously described by our collaborators (da Silva, J. L. et al. Prognostic Influence of Residual Tumor-Infiltrating Lymphocyte Subtype After Neoadjuvant Chemotherapy in Triple-Negative Breast Cancer. Front. Oncol. 11, 636716 (2021)) consisted of 112 women (age mean of 50,5 years old) who underwent neoadjuvant chemotherapy and thereafter curative surgery between 2010 and 2014, and clinical stages II and III. Clinical data regarding age at diagnosis, staging, surgery, histological subtype, chemotherapy, and survival were retrospectively obtained from the medical records and are described in the Supplementary Table 11 in the manuscript.
Recruitment	Ovarian and Breast Cancer Patients from the National Cancer Institute in Rio de Janeiro-Brazil (INCA cohort) who have donated samples to the National Bank Tumor were identified to participate in this study. All participants signed the Informed consent form available at Brazilian National Tumor Bank.
Ethics oversight	This retrospective study was approved by the Ethics in Human Research Committee of the Brazilian National Cancer Institute (INCA) (CAAE 52409221.4.0000.5274 and 61675516.9.0000.5274), Rio de Janeiro, Brazil, and was conducted following the Good Clinical Practice Guidelines.

Note that full information on the approval of the study protocol must also be provided in the manuscript.

Field-specific reporting

Please select the one below that is the best fit for your research. If you are not sure, read the appropriate sections before making your selection.

[x] Life sciences [ ] Behavioural & social sciences [ ] Ecological, evolutionary & environmental sciences

For a reference copy of the document with all sections, see nature.com/documents/nr-reporting-summary-flat.pdf

Life sciences study design

All studies must disclose on these points even when the disclosure is negative.

Sample size	Single-cell donors totalized 142 participants comprising both health and cancer patients in the test dataset, while 88 samples from an independent cohort were used to build a second atlas to validate our finds.<br>Bulk RNA-Seq data from TCGA comprised of 5161 among the following cancer types: OV (n=354 samples), LUAD (n =510), LUSC (n=496), UVM (n=77), SKCM (divided into Metastatic (n=385) and Primary (n= 103) tumor samples), COAD (n=454), READ (n=170), LIHC (n = 369) and BRCA (n = 1195) which comprises Luminal A (n= 569), Luminal B (n=210), HER2 (n=81) and TNBC (n= 188) subtypes. Bulk RNA-Seq from the METABRIC cohort included 1565 participants with Luminal A (n=679), Luminal B (n=461), HER2 (n=220), and TNBC (n=199).<br>Regarding the IHC cohort, we included 193 women diagnosed with high grade serous ovarian cancer and 112 women diagnosed with triple negative breast cancer.
Data exclusions	For single-cell analysis, different filters of quality control were applied by technology to remove low-quality cells from each dataset: for 10x Genomics data the cutoff points were percentage of mitochondrial genes expressed <10, number of counts (nCounts) per cell >200, and ratio of nCounts by number of features (nFeatures) <5; for Smart-Seq2 were percentage of mitochondrial genes expressed <15 , nCounts > 200, and ratio nCounts/nFeature < 1000; and for inDrop were percentage of mitochondrial genes expressed <15, nCount >200, and ratio nCounts/nFeature <1000. The remaining cells were submitted to the doublet removal step through the Scrublet package. Through the doublet score histogram distribution, a cutoff value was determined for each library by manually setting the threshold (Optimal pK), eliminating cells with a higher probability of being doublets.<br>For all bulk RNA-Seq datasets we excluded normal samples.<br>Regarding immunohistochemistry analysis, samples in tissue microarray slides that did not fail experimentally were included in the study.
Replication	For single cell analysis, two independent atlas were building using independent datasets, validating the founds in different tumor types. For tissue microarray, each patient was represented for two to three spots, depending of the cohort, as stated in the methodology section.
Randomization	Randomization was not relevant for this study as there were not multiple groups requiring randomization.
Blinding	No blinded investigation was performed in this study.

Reporting for specific materials, systems and methods

We require information from authors about some types of materials, experimental systems and methods used in many studies. Here, indicate whether each material, system or method listed is relevant to your study. If you are not sure if a list item applies to your research, read the appropriate section before selecting a response.

Materials & experimental systems

n/a	Involved in the study	
☐	☒	Antibodies
☒	☐	Eukaryotic cell lines
☒	☐	Palaeontology and archaeology
☒	☐	Animals and other organisms
☐	☒	Clinical data
☒	☐	Dual use research of concern
☒	☐	Plants

Methods

n/a	Involved in the study	
☒	☐	ChIP-seq
☒	☐	Flow cytometry
☒	☐	MRI-based neuroimaging

Antibodies

Antibodies used	Rabbit Monoclonal anti-Ki67 (clone 30-9 at 1:7, Ventana- Roche),<br>Rabbit monoclonal anti-CD8 (cat. 108R-14,clone SP57 at 1:7, Ventana- Roche),<br>Mouse monoclonal anti-PD-1 (cat. 315M-94, clone NAT105, Cell Marque, diluted 1:100),<br>Mouse monoclonal anti-CD68 (cat. 168M-95, clone KP-1 at 1:20, Ventana- Roche),<br>Mouse monoclonal anti-FOLR2 anti-mouse (cat. MA5-26933, OTI4G6 at 1:200, ThermoFisher),<br>Rabbit monoclonal anti-TREM2 (cat. 91068T, clone D814C, 1:100, Cell Signaling Technology),<br>Rabbit polyclonal anti-PDL-2 (ab200377, at 1:200, Abcam, anti-rabbit).<br>For double staining, TREM2 was combined with anti-CD68, and anti-FOLR2 with anti-PDL-2. Briefly, after completing the first immune reaction (TREM2 or PDL-2) with DAB, the second immune reaction (CD86 or FOLR2) was visualized using MACH4 MR-AP (Biocare Medical) green subtract (Polydetector HRP Green Substrate-chromogen Bio-SB - ref BSB0129t).
Validation	Each antibody was validated as per the manufacturer's instructions. Antibodies were all validated by verifying staining for an antigen-expressing tissue but not staining for non-expressing tissue. The dilution of antibodies was titrated to obtain the best concentration to use that stained positive cells, but not negative cells. The antibodies were tested in different dilutions following manufacturer instructions. PD-1, CD8, FOLR2 and PDL-2 were evaluated in the amygdala, CD68 and TREM2 in melanoma, and Ki67 in follicular lymphoma.

Clinical data

Policy information about clinical studies

All manuscripts should comply with the ICMJEguidelines for publication of clinical research and a completedCONSORT checklist must be included with all submissions.

Clinical trial registration

Provide the trial registration number from ClinicalTrials.gov or an equivalent agency.

Study protocol

Note where the full trial protocol can be accessed OR if not available, explain why.

Data collection

For the ovary cohort, 193 all women diagnosed with HGSOC at INCA between 2001 and 2017, regardless of adjuvant or neoadjuvant treatment, were included in our study. The TNBC cohort, previously described by our collaborators consisted of 112 women who underwent neoadjuvant chemotherapy and thereafter curative surgery between 2010 and 2014. Clinical data regarding age at diagnosis, staging, surgery, histological subtype, chemotherapy, and survival were retrospectively obtained from the medical records. The two INCA cohorts are described in more detail in Supplementary Data 11.

Outcomes

Outcomes were defined as Overall Survival and Progression-free Survival for ovary cohort and Overall Survival and Event-Free Survival for the breast cohort. In both cases, events were considered in a five-year interval.

Plants

Seed stocks

Report on the source of all seed stocks or other plant material used. If applicable, state the seed stock centre and catalogue number. If plant specimens were collected from the field, describe the collection location, date and sampling procedures.

Novel plant genotypes

Describe the methods by which all novel plant genotypes were produced. This includes those generated by transgenic approaches, gene editing, chemical/radiation-based mutagenesis and hybridization. For transgenic lines, describe the transformation method, the number of independent lines analyzed and the generation upon which experiments were performed. For gene-edited lines, describe the editor used, the endogenous sequence targeted for editing, the targeting guide RNA sequence (if applicable) and how the editor was applied.

Authentication

Describe any authentication procedures for each seed stock used or novel genotype generated. Describe any experiments used to assess the effect of a mutation and, where applicable, how potential secondary effects (e.g. second site T-DNA insertions, mosiacism, off-target gene editing) were examined.
