# Supplementary material for: Single-cell resolution characterization of myeloid-derived cell states with implication in cancer outcome
Source: Nat Commun. 2024 Jul 7;15:5694. doi: 10.1038/s41467-024-49916-4 (PMC11228020; doi:10.1038/s41467-024-49916-4)
Supplement: Supplementary file 2 — Peer Review File [file 41467_2024_49916_MOESM2_ESM.pdf]

nature portfolio

Peer Review File

Single-cell resolution characterization of myeloid-derived cell states with implication in cancer outcome

Open Access This file is licensed under a Creative Commons Attribution 4.0 International License, which permits use, sharing, adaptation, distribution and reproduction in any medium or format, as long as you give appropriate credit to the original author(s) and the source, provide a link to the Creative Commons license, and indicate if changes were made. In the cases where the authors are anonymous, such as is the case for the reports of anonymous peer reviewers, author attribution should be to 'Anonymous Referee' followed by a clear attribution to the source work. The images or other third party material in this file are included in the article's Creative Commons license, unless indicated otherwise in a credit line to the material. If material is not included in the article's Creative Commons license and your intended use is not permitted by statutory regulation or exceeds the permitted use, you will need to obtain permission directly from the copyright holder. To view a copy of this license, visit http://creativecommons.org/licenses/by/4.0/.

REVIEWERS' COMMENTS

Reviewer #1 (Remarks to the Author): expertise in macrophage polarisation and signatures

Guimaraes et al mine multiple public scRNAseq datasets, generated via different techniques, and integrate them to provide an overview of all possible myeloid cells present in the tumor microenvironment of multiple cancer types. This is an overall interesting effort, with some valuable information, but also several issues:
1) I don't doubt the rigorous technique and analysis presented by the authors, but not all populations they claim to be novel are effectively novel.
For example, monocyte-derived hypoxic macrophages (ie Mac-Hypo) are very well known since along time and also IFN-primed and lipid metabolism-associated macrophages (both RTM and mono-derived) have been described in human GBM tumors (Pombo Antunes et al, Nature Neurosci, 24(4):595-610, 2021) and other tumor types (Masetti et al, J Exp Med. 2022 Feb 7;219(2):e20210564). The authors should therefore be more careful when stating that a population is "new" and should refer to previous articles reporting similar populations.
2) A general issue is the overinterpretation of data at some levels, especially when assigning functions to macrophages based on the expression of one or two markers. For example, the immunosuppressive capacity of macrophages is linked to many more molecules and pathways than TREM and PD-L alone, so the authors may underestimate the immunosuppressive capacity of macrophages with a lower expression of those genes.
3) "cDC2_CD14 and cDC2_FCGR3A are likely monocyte-derived". That's a contradiction: cDC are defined as cells derived from a committed cDC progenitor, so these cells are never monocyte-derived. I would call them MoDC instead of cDC2. The same remark is valid for the statement at line 372.
4) One should be careful to designate macrophages as tissue-resident or monocyte-derived or something in between, based on a few markers in the human system. In mouse, hard evidence can be provided for the cells' ontogeny thanks to lineage-tracing technologies, which are not possible in human. With this caveat, the authors should mention the difficulty of making firm statements on ontology in humans.
5) The name 'recruited macrophage' (Mac_Rec) is weird because all Mac clusters are supposed to be monocyte-derived, and hence, are by definition recruited to the tumor
6) The MDSC cell signature should certainly not be evaluated in macrophage populations, since MDSCs are by definition immature myeloid cells with either a monocytic or granulocytic profile. This signature should be tested on the monocyte and neutrophil clusters, not the macrophage clusters.
7) In HGSOC, TREM and CD68 were associated with higher CD8 and PD1. The latter can be a sign of T-cell activation or T-cell exhaustion. Which option is it?

Reviewer #3 (Remarks to the Author): expert in single cell RNA-seq data integration

Upon reviewing the manuscript regarding the role of tumor-associated myeloid-derived cells (MDCs) in cancer prognosis and treatment response, I recognize the authors' effort in this area. The authors have integrated scRNA-Seq datasets from seven distinct cancers, an undertaking worthy of note. The resulting atlas, showcasing 29 MDC subpopulations within the tumor microenvironment (TME), is extensive. However, the validity of this atlas warrants meticulous scrutiny.

My reservations stem from the analysis based on public data integrating three types of scRNA-Seq analyses. The authenticity of these populations remains a point of contention for me. The findings might be influenced by underlying technical biases.

Below are my comments and suggestions:

Major Comments:

In light of my primary concerns, and given the inherent technical biases when integrating data

from diverse public datasets, I recommend that the authors validate the existence and marker genes of the newly identified cell states. Leveraging some high-quality, uncorrected individual Smart-seq datasets could enhance the credibility of the conclusions drawn.

Given the integral role of data integration in this study, I suggest the authors employ an additional 1-2 atlas-tailored integration methods to ascertain the reproducibility of the identified cell states.

From a methodological perspective, it is conventionally advised to execute ambient RNA removal (DecontX) before tackling batch effect removal. It is crucial for the authors to justify the post-clustering DecontX analysis.

Fig 1a: The repeated step of first-level cell type annotation post DecontX is not elaborated in either the main text or the methods section.

This study used ovarian normal tissue. I would suggest considering using the fallopian tube as the normal tissue as the fallopian tube epithelium is regarded as the cell of origin for most HGSOC cases.

The authors should address the likelihood that the observed M1/M2 continuum might stem from single-cell data noise or artificial impacts from preprocessing, particularly during the data integration phase.

Minor comment:

In Supplementary fig 2c, the legend bar appears absent.

At Line 309, the authors have referred to certain cell states validated by other research groups. It would be valuable to explicitly say which of these states have been validated by other research groups and which ones are newly identified by the current study. Integrating this information into a diagram (e.g. Fig 4j) will be helpful.

While Fig4j is quite informative, the text size of this figure and others could be increased for better readability. Moreover, incorporating details on which cell states have been experimentally validated previously and which have not would enrich the diagram.

Reviewer #4 (Remarks to the Author): expertise in immune subtypes in scRNA-seq data

Guimaraes et al conducted pan-cancer single cell analysis on tumor-associated myeloid derived cells (MDCs) in 7 different tumor types with 3 different platform, identifying some subpopulations with possible prognostic value. Most of the findings have been reported by a published pan-cancer single cell transcriptional atlas of tumor infiltrating myeloid cells (Cheng et al, Cell 2021). Given this study included a smaller sample size, careful comparison between this study and the previous pan-cancer MDC analysis should be conducted to indicate the possible novelty of this study.

Major
1. This study integrated 7 different tumor types (6 epithelia tumor type and melanomas) The sample size is relatively small to exclude the batch effect of different studies. At least two studies are needed for each cancer type to exclude the possible bias of the studies included. What is the criteria to select the studies included in this study? If the authors do not want to discuss the differences and shared characteristics between epithelial tumor and non-epithelial tumor, it is better to delete the melanomas related study.
2. Three different sequencing platforms were included, although the main clustering is similar between different platform, subpopulation and molecular alterations may be impacted by different platforms.
3. Findings on several cell types (e.g., mast cells, FOLR2+ TAMs) have been reported in the previous study (Cheng et al, Cell 2021), what is the difference or novelty of this study on these cell types?

4. Whether the author check if the TREM+ TAMs can match to a certain cell type with another marker co-expressed with TREM in previous study, if no, explain why, if yes, what is the difference of novelty of this study on this cell type.
5. Low quality cells in Fig S1c should be deleted before performing clustering.
6. Figure 1b, Y axis represents tumor type, but PBMC is not a tumor type mentioned in this study. In addition, PBMCs were not mentioned at all in the result and discussion part, what is the point to add a small number of PBMC cells in this study.
7. Subpopulation displayed in Figure 1g is important for the subsequent analysis in this study, illustration with Figure 1g is not enough.
8. Neutrophil is fragile, and the proportion of neutrophil can greatly impact by the process of operating single cell preparation. Because this study integrated the public resource of single cell data with different handling situation, it is not suitable to investigate the neutrophils.
9. Figure 2a is redundant, and annotation of some DC cells may be incorrect. For instance, positive rate of CD1C is too low for cDC in Figure 1e, suggesting an involvement of cDC1, cDC2, pDC and moDC. Additionally, cDC2_CD14 is moDC in Figure 2c, further support the mislabeling of Figure 1
10. Experimental validation with mIF should be conducted in clinical samples.

Minor
1. The number of patients and samples should be included in Table S1
2. The quality of most figures is low (e.g., Figure 1a, 1f), some words are hard to read.
3. Patient number of each group should be labeled in KM curves
4. inferCNV was mentioned in the Methods part, but no CNV analysis results have been illustrated

RESPONSE TO REVIEWERS' COMMENTS:

Reviewer #1 (Remarks to the Author):

Guimaraes et al mine multiple public scRNAseq datasets, generated via different techniques, and integrate them to provide an overview of all possible myeloid cells present in the tumor microenvironment of multiple cancer types. This is an overall interesting effort, with some valuable information, but also several issues:

1) I don't doubt the rigorous technique and analysis presented by the authors, but not all populations they claim to be novel are effectively novel.
For example, monocyte-derived hypoxic macrophages (ie Mac-Hypo) are very well known since along time and also IFN-primed and lipid metabolism-associated macrophages (both RTM and mono-derived) have been described in human GBM tumors (Pombo Antunes et al, Nature Neurosci, 24(4):595-610, 2021) and other tumor types (Masetti et al, J Exp Med. 2022 Feb 7;219(2):e20210564). The authors should therefore be more careful when stating that a population is “new” and should refer to previous articles reporting similar populations.

Thank you for your feedback on our manuscript. We greatly appreciate the time and expertise you have dedicated to reviewing our work, and we are particularly encouraged by your recognition of the rigorous technique and analysis we employed.

Regarding your concern about the novelty of certain myeloid cell populations that we have identified, we acknowledge your point. We understand the importance of accurately representing the novelty of our findings, especially in the context of previous studies such as those by (Pombo Antunes et al. 2021) and (Masetti et al. 2022) that you have referenced, as well as studies conducted by (Cheng et al. 2021; Qian et al. 2020).

However, a key advancement of our research lies in our ability to differentiate between cell states that have often been grouped together in prior studies. In multiple TME studies, populations such as IFN-primed (Molgora et al. 2023), TREM2 (Katzenelenbogen et al. 2020; Mulder et al. 2021), or FOLR2 macrophages (Nalio Ramos et al. 2022; Xiang et al. 2023) have been frequently aggregated into single categories (Casanova-Acebes et al. 2021; Ma, Black, and Qian 2022a). However, in our work, we acknowledged previous work where some of these populations have been already described, and, for some of these previously described populations, our analysis gave a level of granularity that offered insights into the complexity of these myeloid cells and how their behavior depends on the TME.

For instance, we identified at least four distinct macrophage states expressing elevated levels of *TREM2*, previously generically classified as *TREM2* macrophage subpopulations. Importantly, our investigation revealed that these subpopulations exhibit disparate functional profiles, with one associated with lipid metabolism and another linked to antigen presentation gene sets. The clinical impact of these TREM2+ subpopulations varied significantly, contingent upon the tumor niche and displayed phenotype. A notable example of our findings is the identification of a specific population, named MAC_LA, characterized by high co-expression of TREM2 and PD-1.

While prior studies have demonstrated that *TREM2* macrophages exhibit a lipid metabolism-associated phenotype, our research uniquely reveals that this specific subpopulation co-express *PD-1*. It is noteworthy that, among the four subpopulations expressing high levels of *TREM2*, only MAC_LA expressed *PD-1*, highlighting nuanced differences within these cell types and underscoring their distinct clinical impacts. This novel finding aligns with previous works indicating that deficiency or blockade of TREM2^{+} macrophages can enhance responses to anti-PD1 therapies (Molgora et al., 2020; Molgora et al., 2023).

The same meticulous characterization was undertaken for resident-tissue macrophages, challenging the conventional identification of these macrophages as a singular entity. Our findings reveal the presence of at least three distinct profiles within the TME, each exhibiting either pro- or anti-tumorigenic activity. Notably, we report, for the first time, the association of a resident-tissue macrophage expressing elevated levels of *FOLR2* and *SEPP1* with poor prognosis in triple-negative breast cancer, thereby unveiling new prospects for the development of targeted therapies. This granular analysis marks a significant advancement in understanding the intricate complexity and heterogeneity of the TME, serving as a foundational reference for future research endeavors.

In response to the valuable feedback from the reviewers, we have duly acknowledged prior studies that identified similar cell populations in the revised version of the macrophages diagram (Fig 4j) and produced a new supplementary table describing previous works that have identified similar subpopulations (Table Supplementary 3). Furthermore, we have modified the manuscript title and adjusted our language to more accurately reflect the degree of novelty in our findings.

2) A general issue is the overinterpretation of data at some levels, especially when assigning functions to macrophages based on the expression of one or two markers. For example, the immunosuppressive capacity of macrophages is linked to many more molecules and pathways than TREM and PD-L alone, so the authors may underestimate the immunosuppressive capacity of macrophages with a lower expression of those genes.

We thank the reviewer for pointing this out. We would like to clarify that we have proposed macrophage functions based on functional signatures (as shown in Figure 4g and Supplementary Table 4). Regarding the immunosuppressive capacity of macrophages in our study, we used at least three distinct approaches:

1) We conducted a comprehensive analysis of the literature to identify a broad spectrum of M2 markers described in previous works. This approach allowed us to cross-validate the expression of known markers in our identified cell populations, enhancing the reliability of our functional assignments. It also helped in contextualizing our findings within the existing body of knowledge (Figure 5a).
2) We extended our analysis to include markers associated with immune checkpoints, such as PD1, PD-L1, and PD-L2 (Figure 5l).
3) Finally, we performed a general pathway enrichment analysis (Supplementary Table 5 and Figure 4h) to understand the broader biological pathways and processes in which these macrophages are involved. This approach provided a holistic view of the macrophage function in the tumor microenvironment, moving beyond the limitations of marker-based analysis. Enriched pathways associated with immunosuppression such as PD-1 or IL-10 pathway were found in the following subpopulations:
    a) **IL-10:** RTM_Int, Mac_Angio, Mac_INF, Mac_ES, Mac_Hypo, RTM_like_MT, RTM_INF,
    b) **PD-1:** Mac_AgPres, RTM_INF

By combining these approaches, we were able to mitigate the risk of overinterpretation based on a limited set of markers, providing a more comprehensive understanding of what we referred to as the immunosuppressive capacity of macrophages in the tumor microenvironment.

We hope that these points clarify our methodology and address your concerns regarding the potential underestimation of macrophage immunosuppressive populations. Functional analysis of these sub-populations needs to be performed by future studies to confirm their immunosuppressive role.

3) “cDC2_CD14 and cDC2_FCGR3A are likely monocyte-derived”. That's a contradiction: cDC are defined as cells derived from a committed cDC progenitor, so these cells are never monocyte-derived. I would call them MoDC instead of cDC2. The same remark is valid for the statement at line 372.

Thank you for your important observation regarding the classification of cDC2_CD14 and cDC2_FCGR3A cells. Thanks for pointing out the traditional definition of conventional dendritic cells (cDCs) as being derived from a committed cDC progenitor, and not from monocytes. We appropriately classified these cells as monocyte-derived dendritic cells (MoDCs).

In light of your feedback, we have revised our manuscript and replaced the terms "cDC2_CD14" and "cDC2_FCGR3A" with "MoDC_CD14" (line 192) and "MoDC_FCGR3A (line 193)."

4) One should be careful to designate macrophages as tissue-resident or monocyte-derived or something in between, based on a few markers in the human system. In mouse, hard evidence can be provided for the cells' ontogeny thanks to lineage-tracing technologies, which are not possible in human. With this caveat, the authors should mention the difficulty of making firm statements on ontology in humans.

Thank you for your thoughtful comment on the classification of macrophages as tissue-resident or monocyte-derived. We understand and acknowledge the complexities and limitations of categorizing macrophage ontogeny in humans, as compared to the more definitive lineage-tracing technologies available in mouse models.

In our study, we have approached the classification of macrophage origin with caution, based on recent advancements in the field. Specifically, we referred to the comprehensive study published in Nature (Bian et al. 2020) as a guide for identifying a collection of markers indicative of macrophage origin. This paper provides a detailed characterization of macrophage populations in multiple anatomical sites, offering insights into the markers associated with tissue residency.

Based on this resource, we carefully selected a panel of markers related to the two distinct waves of yolk sac-derived macrophages that contribute to TRM populations in human embryos, in addition to markers related to tissue residency or monocyte-derivation to infer the likely origin of the macrophages in our study:

First wave: *CD163*
Second wave: *MPO*, *HLA-DRA*
TRM related: *TCF12*, *MS4A4A*, *GATM*, *LYVE1*, *PLTP*, *FOLR2*
Monocyte fate: *CCR2*
Monocyte related: *VCAN, S100A6, CD52*

This panel has been added in Figure 4 to help clarify these classifications:

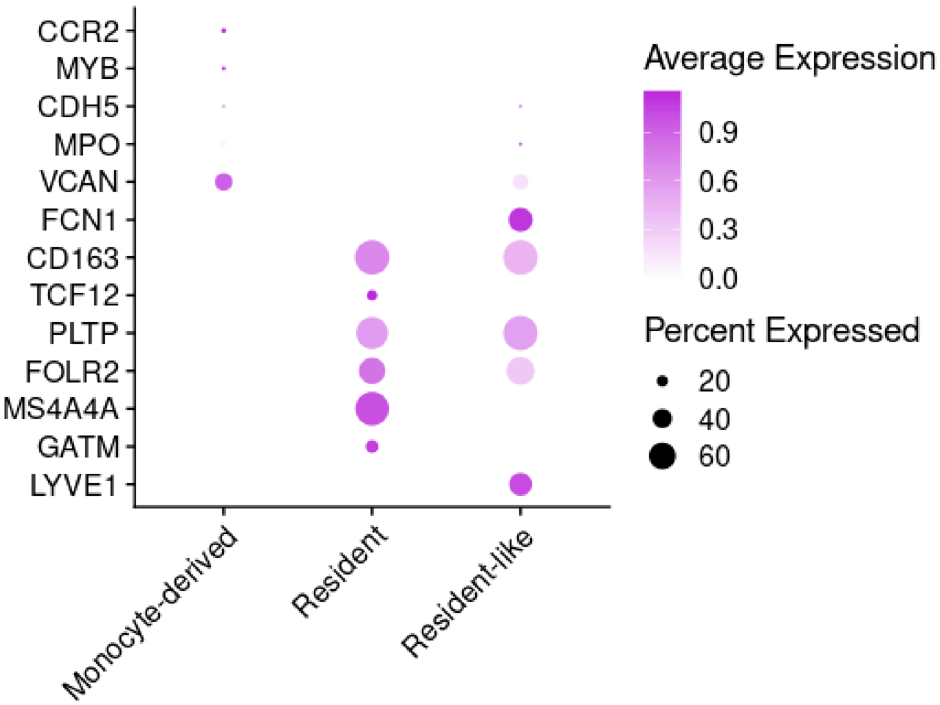

Panel 4b: Dot plot visualizing the differential expression of marker genes associated with macrophage ontogeny. Each dot's color intensity indicates the average gene expression level, while the size reflects the percentage of cells expressing the gene within the population.

While we understand that this approach does not provide the same level of certainty as lineage tracing in mouse models, we believe it is currently one of the best methods available for human samples. Nevertheless, we agree with your suggestion that our manuscript should explicitly address the inherent challenges in making definitive statements about macrophage ontogeny in humans. In our revised manuscript, we have included a discussion on the limitations and challenges of classifying macrophage ontogeny in human samples, highlighting the absence of lineage-tracing technologies and emphasizing that our classifications are based on the best available evidence while acknowledging the need for caution in interpreting these findings due to the limitations of current human-specific methodologies.

In addition, the following statement was added to the manuscript:
Line 581 "We endeavored to infer the likely origins of macrophages within our study, differentiating between those that are suggestive of tissue residency and those potentially derived from monocytes, while acknowledging the inherent challenges in classifying macrophage ontogeny in human samples. This distinction was drawn using the best available evidence and methodologies, including analysis of specific markers as guided by current literature (Bian et al. 2020). However, we recognize the limitations of these approaches, particularly the absence of unequivocal lineage-tracing methods in human studies."

5) The name 'recruited macrophage' (Mac_Rec) is weird because all Mac clusters are supposed to be monocyte-derived, and hence, are by definition recruited to the tumor

Thank you for your observation regarding our use of the term 'recruited macrophage' for the Mac_Rec cluster in our manuscript. Our initial choice of the term was influenced by the nomenclature used in a study by (Qian et al. 2020). The Mac_Rec cluster is akin to the

C3_CCR2 subpopulation identified by Qian et al., which was characterized as a group of immature macrophages closely related to monocytes and called "recruited" by the authors. This subpopulation notably expressed *CCR2*, indicative of an early stage in macrophage development. However, we acknowledge your concern that the term 'recruited macrophage' might lead to confusion, given the general understanding of macrophage recruitment in tumor environments. To avoid any ambiguity and to ensure clear communication of our findings, we have decided to rename this macrophage cluster as 'Early stage macrophage ' (Mac_ES).

6) The MDSC cell signature should certainly not be evaluated in macrophage populations, since MDSCs are by definition immature myeloid cells with either a monocytic or granulocytic profile. This signature should be tested on the monocyte and neutrophil clusters, not the macrophage clusters.

Thank you for your feedback regarding the evaluation of the MDSC signature. We understand and agree with your point that the MDSC cell signature, being characteristic of immature myeloid cells with either a monocytic or granulocytic profile, may not be appropriately evaluated in macrophage populations. In our initial analysis, we sought to comprehensively test the MDSC signature across all myeloid-derived cells, including macrophages, to explore it broadly. However, we recognize the need for a more focused and accurate approach. Accordingly, we have revised our analysis to exclude macrophages, mast, and dendritic cells of this analysis (Supplementary Fig. 9).

Line 398: We substituted the previous text with this: "*We also evaluated the myeloid-derived suppressor cell (MDSCs) signature proposed by (Alshetaiwi et al. 2020) in all immature MDC. However, none of these subpopulations resembled that particular signature (Supplementary Fig. 9). Nevertheless some monocytes exhibited higher suppressive profiles, such as Mono_CD14_FOS⁻, based on IL1B expression, and MonoInter_CXCL10 and MonoInter_CLEC10A, based on CD84 expression (Supplementary Fig. 9)*."

7) In HGSOC, TREM and CD68 were associated with higher CD8 and PD1. The latter can be a sign of T-cell activation or T-cell exhaustion. Which option is it?

Thank you for your query regarding the association of macrophage expression of TREM2, CD68, and PD-1 with higher CD8 levels in HGSOC. In our study, we have shown in Figure 5I and Figure 6D that the phenotype named Mac_LA highly co-expresses *TREM2* and *PD-1*.

It is interesting to note that patients with high $TREM2^+$ and $CD68^+$ macrophages presented an enrichment of $PD\text{-}1^+$ inside the tumor (Figure 6h). We also observed that patients with more $TREM2^+$ macrophages have less $CD8^+$ and more $PD\text{-}1^+$, while patients with less $TREM2^+$ macrophages have more $CD8^+$ and less $PD\text{-}1^+$. Therefore, we hypothesized that the expression of $PD\text{-}1^+$ observed in this experiment is highly associated with the increase of the macrophage populations Mac_LA (*CD68*, *TREM2*, and *PD-1*), rather than $CD8^+$ T cells.
Through survival analysis, illustrated by Kaplan-Meier curves, our analysis indicates that high levels of protein TREM2 in HGSOC, as well as the high predicted amount of Mac_LA (*CD68*, *TREM2*, and *PD-1*) observed through deconvolution analysis, is associated with a poor prognosis (Figura S14C and figure 6E), but not $PD\text{-}1^+$ levels solely (Figure S20G). In consonance, high $CD8^+$ levels in HGSOC didn't impact overall survival.

It is crucial to note that while $CD8^+$ T cell infiltration is often considered a marker of immune activation, however, in the context of *PD-1* expression on macrophages, it could potentially represent a more complex interplay where T cells are drawn into the tumor microenvironment but face an immunosuppressive milieu. This could result in a scenario where T cells are present but may not be fully activated or may even be rendered exhausted due to the suppressive signals emanating from PD1-expressing macrophages.

In summary, our interpretation, backed by emerging evidence, suggests that PD-1$^+$ expression on macrophages in HGSOC is indicative of an immunosuppressive environment, potentially influencing the functional state of co-localized CD8$^+$ T cells. However, to provide further insights into this matter, future studies will be necessary to incorporate immunohistochemistry strategies to co-mark PD-1$^+$ and TREM2$^+$, since PD-1$^+$ can be expressed by different cells in the TME, including tumor cells.

Reviewer #3 (Remarks to the Author):

Upon reviewing the manuscript regarding the role of tumor-associated myeloid-derived cells (MDCs) in cancer prognosis and treatment response, I recognize the authors' effort in this area. The authors have integrated scRNA-Seq datasets from seven distinct cancers, an undertaking worthy of note. The resulting atlas, showcasing 29 MDC subpopulations within the tumor microenvironment (TME), is extensive. However, the validity of this atlas warrants meticulous scrutiny.

My reservations stem from the analysis based on public data integrating three types of scRNA-Seq analyses. The authenticity of these populations remains a point of contention for me. The findings might be influenced by underlying technical biases.

Below are my comments and suggestions:

Major Comments:

In light of my primary concerns, and given the inherent technical biases when integrating data from diverse public datasets, I recommend that the authors validate the existence and marker genes of the newly identified cell states. Leveraging some high-quality, uncorrected individual Smart-seq datasets could enhance the credibility of the conclusions drawn.

Thank you for your comprehensive review and insightful comments regarding our manuscript. We deeply appreciate your recognition of our effort in integrating scRNA-Seq datasets from distinct cancers, and we acknowledge the importance of addressing your concerns to ensure the validity of our atlas.

Your reservation regarding the potential technical biases in integrating diverse public scRNA-Seq datasets is well understood. We agree that the authenticity of the identified cell populations requires rigorous validation, especially when utilizing data from multiple sources.

That is the reason why we have also previously compared our main findings with the cell populations found in two other previous works (Mulder et al. 2021; Cheng et al. 2021) (Supplementary Figure 7) to strengthen the credibility of our findings.

In addition, we have validated the newly identified cell states existence and marker genes using independent datasets of ovarian and breast tumors obtained from additional publicly available data.

Similarly, within an independent breast dataset (n = 11,170), integrated across three diverse studies (Bassez et al. 2021; Pal et al. 2021; Wu et al. 2021), the presence of Mac_IFN (IFIT2+), Mac_LA (LPL+), RTM_IFN (CXCL9+), and RTM_Int (SEPP1+) was also confirmed. These findings were depicted in Supplementary Figure 8 a-d. For ovarian tumors (n = 8,987), the validation involved an integrated dataset derived from five distinct studies (Schelker et al. 2017; Olalekan et al. 2020; Slyper et al. 2020; Hornburg et al. 2021; Zhang et al. 2022). In this comprehensive dataset, there was a notable correspondence with four macrophage types identified in the primary dataset. These included: Mac_IFN (IFIT2+), Mac_Angio (VEGFA+), RTM_Int (SEPP1+), and RTM-like_MT (MT2E+). This overlap was illustrated in Supplementary Figure 8 e-f.

This cross-validation across independent datasets from ovarian and breast tumors reinforces the reliability of the identified macrophage subtypes and their associated markers, suggesting a broader relevance of these cell states and markers in different tumor contexts.

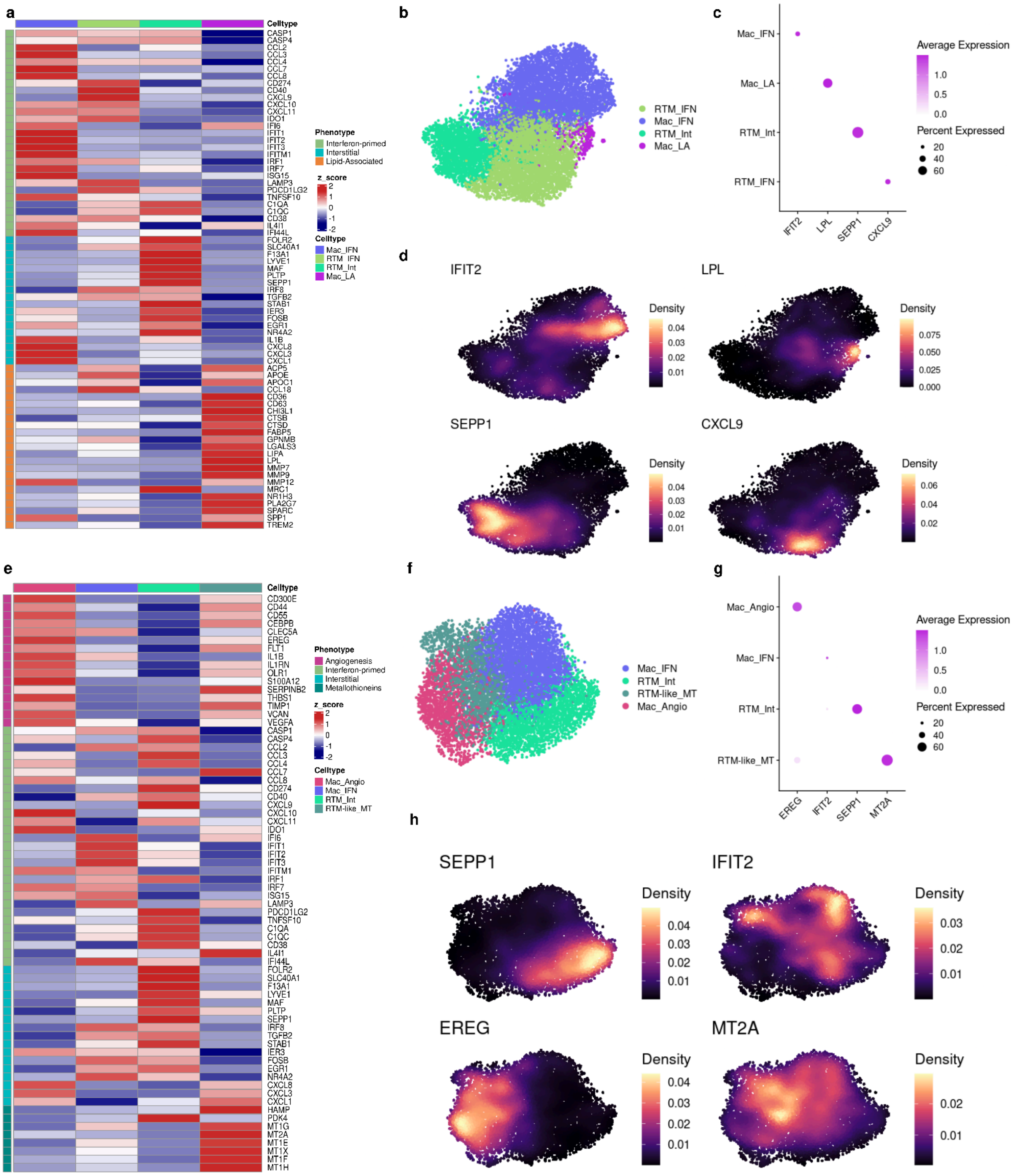

Supplementary Figure 8. Independent Validation in Breast and Ovarian Cancer Cohorts. (a) Heatmap showing the interferon-primed, interstitial, and lipid-associated gene signature across Mac_IFN, RTM_IFN, RTM_Int, and Mac_LA found in breast cancer cohort. (b) UMAP of macrophages colored by the states identified. (c) Dot plot showing the mean expression of genes related to the Mac subpopulation. The dot size indicates the percent of expressing cells, and the dot color is the scaled average expression. (d) Density plots highlighting the gene expression of each cell. (e) Heatmap showing the angiogenesis, interferon-primed, interstitial, and metallothioneins gene signature across Mac_Angio, Mac_IFN, RTM_Int, and RTM-like_MT. (f) UMAP of macrophages colored by the states identified. (g) Dot plot showing the mean expression of genes related to the Mac subpopulation. The dot size indicates the percent of expressing cells, and the dot color is the scaled average expression. (h) Density plots highlighting the gene expression of each cell.

Given the integral role of data integration in this study, I suggest the authors employ an additional 1-2 atlas-tailored integration methods to ascertain the reproducibility of the identified cell states.

Thank you for your suggestion concerning the data integration approach in our study. Initially, we chose scVI (single-cell Variational Inference) for data integration, primarily influenced by the study published in Nature Methods (2021) (Luecken et al. 2022). This paper highlights scVI's advanced capabilities in integrating scRNA-Seq data from varied sources, effectively managing batch effects and technical variations while preserving the biological integrity of the data. The robustness and versatility of scVI, as demonstrated in this and other studies, made it an ideal choice for our initial analysis. Following the reviewer's suggestion, we have employed an additional integration with BBKNN (Polański et al. 2020)for comparison and the scib tool (https://github.com/theislab/scib) to analyze the performance of the integration methods.

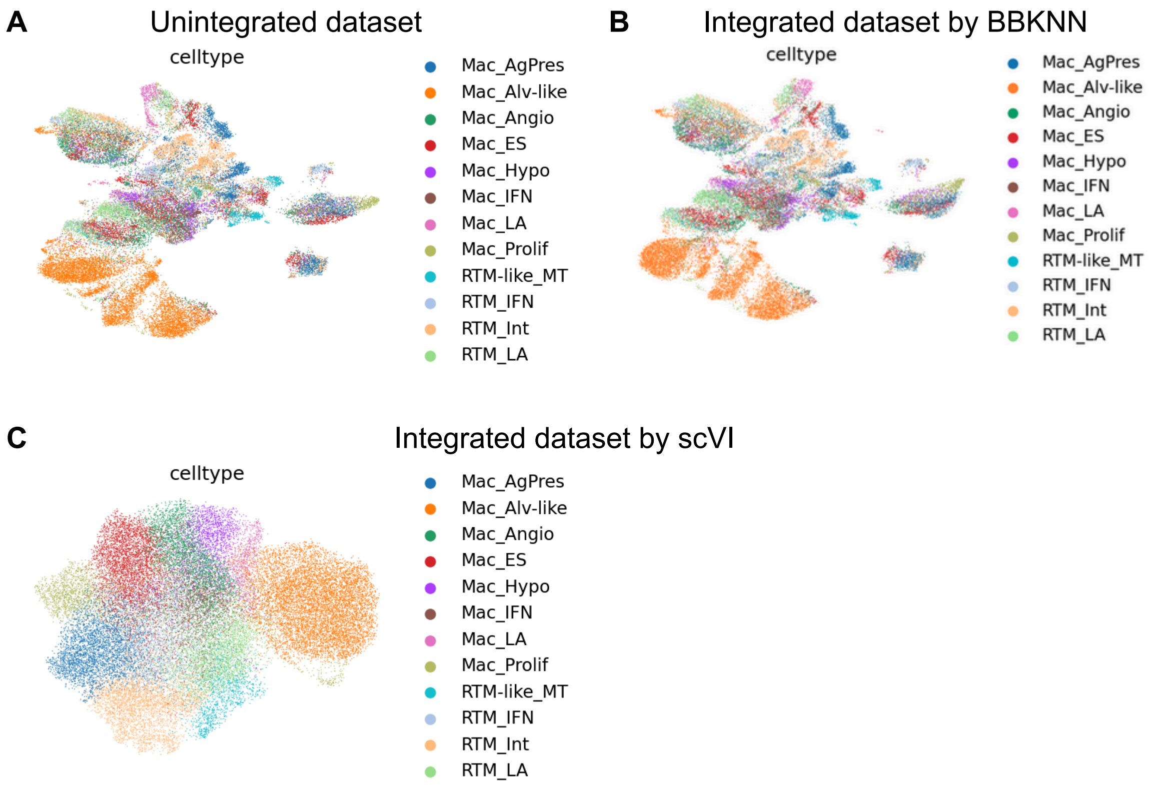

Figure for reviewers 1: Comparison of integration approaches. Panel A shows the UMAP of an unprocessed, unintegrated dataset displaying all the macrophages subpopulations. Panels B and C depict the data following integration with two different computational approaches: batch correction by BBKNN (Panel B) and by scVI (Panel C). Each point represents a single cell, colored according to the identified macrophage subtype.

Scib tool retrieved many metrics related to the biological conservation of the data and the batch correction performed by the chosen integration method. By doing that we could affirm that our integration approach using scVI was successful since most of the metrics values outperformed the other integration method, not only in batch correction but also in biological conservation (Table 1).

	Biological Conservation				Batch Correction		
	Unintegrated dataset	Integrated dataset by BBKNN	Integrated dataset by scVI		Unintegrated dataset	Integrated dataset by BBKNN	Integrated dataset by scVI
ARI_cluster/label(1)	0.17	0.18	0.45	graph_conn(6)	0.93	0.93	1.0
ASW_label(2)	0.55	0.56	0.62	ASW_label/batch (7)	0.58	0.58	0.41
isolated_label_F1(3)	0.50	0.49	0.68				
NMI_cluster/label(4)	0.29	0.3	0.63				
isolated_label_silhouette(5)	0.49	0.49	0.57				

(1) The adjusted rand index is a chance-adjusted rand index, which evaluates the pair-wise accuracy of clustering vs. ground truth label assignments. The score ranges between 0 and 1 with larger values indicating better conservation of the data-driven cell identity discovery after integration compared to annotated labels.
(2) Score how well-isolated labels are distinguished from all other labels using the average-width silhouette score (ASW)
(3) Score how well-isolated labels are distinguished from other labels by data-driven clustering. The F1 score is used to evaluate clustering with respect to the ground truth labels.
(4) The normalized mutual information is a version of the mutual information corrected by the entropy of clustering and ground truth labels (e.g. cell type). The score ranges between 0 and 1, with 0 representing no sharing and 1 representing perfect sharing of information between clustering and annotated cell labels.
(5) Average silhouette width (ASW). Wrapper for sklearn silhouette function values range from [-1, 1] with
    (a) 1 indicates distinct, compact clusters
    (b) 0 indicates overlapping clusters
    (c) -1 indicates core-periphery (non-cluster) structure
(6) Graph Connectivity. Quantify the connectivity of the subgraph per cell type label. The final score is the average for all cell type labels, according to the equation: where stands for all cells in the largest connected component and stands for all cells of cell type.
(7) Batch ASW. Modified average silhouette width (ASW) of batch. This metric measures the silhouette of a given batch. It assumes that a silhouette width close to 0 represents the perfect overlap of the batches, thus the absolute value of the silhouette width is used to measure how well batches are mixed.

From a methodological perspective, it is conventionally advised to execute ambient RNA removal (DecontX) before tackling batch effect removal. It is crucial for the authors to justify the post-clustering DecontX analysis. Fig 1a: The repeated step of first-level cell type annotation post DecontX is not elaborated in either the main text or the methods section.

Thank you for pointing out the methodological approach regarding the use of DecontX for ambient RNA removal. We understand your concern since also in our experience, ambient RNA strongly influences the integration.

The decision to employ DecontX after the initial clustering step was made to address ambient RNA interference while preserving the integrity of clusters with minimal contamination. DecontX effectively removes contamination by considering observed expression as a combination of native transcript counts from the actual cell population and contaminating transcript counts from other cell populations in the assay. Accurate cell type labeling is crucial for DecontX to effectively identify contamination. The function is applied as follows: decontX(x = sce, z = sce$celltype, batch = sce$batch_key), where 'z' represents the "broad celltype" (cell cluster labels), and 'batch' corresponds to each sample. In cases where cell annotations are unavailable for the input matrices, DecontX treats the entire input background as empty droplets. It is important to note that DecontX can account for the batch effect in its modeling while performing the task of removing ambient RNA interference.

By applying DecontX post-first round clustering, we aimed to conduct a targeted decontamination, focusing on clusters with evident ambient RNA contamination. This approach was designed to ensure the preservation of clusters with minimal contamination and to maintain the integrity of genuine biological signals that could potentially be mistaken for contamination. We hypothesized that clustering different datasets while accounting for batch effects would provide a clearer distinction between true biological variation and technical noise, allowing for more precise decontamination. To clarify the methodology employed in this work, we have improved our methodological workflow scheme (Figure 1a).

In regards to the repeated step of first-level cell type annotation post DecontX serves the purpose of refining the cell type annotations after contamination removal. For these other annotation levels, we selected subsets of broad cell types (i.e., macrophages or monocytes) and re-ran analyses on highly variable genes and scVI models, maintaining consistent parameters, and correcting for batch effects, which was performed individually for each sample. We have elaborated this better in the main text (line 804): “Post DecontX, we repeated the step of first-level cell type annotation, refining the cell type annotations after contamination removal. This approach was designed to ensure the preservation of clusters with minimal contamination and to maintain the integrity of genuine biological signals that could potentially be mistaken for contamination.”

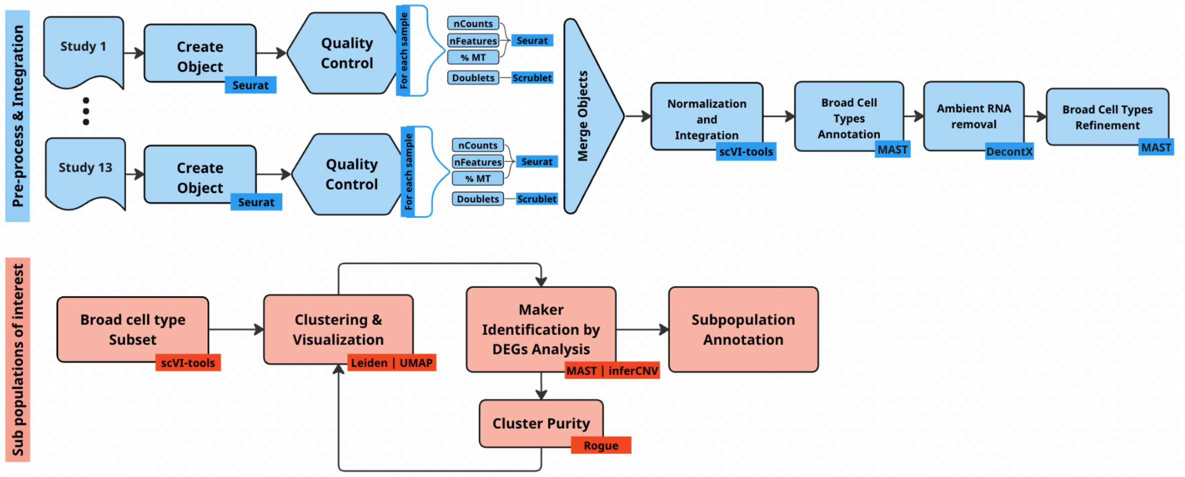

Figure 1a - Workflow for Integrative Analysis of Single-Cell RNA-Sequencing Data outlining a comprehensive pipeline from preprocessing multiple single-cell RNA-sequencing studies through quality control, data integration, and normalization, to the identification and annotation of cell subpopulations. Key analytic tools are noted at each step, including Seurat for object creation and quality control, scVI-tools for data normalization and integration, and various other methods for cell type annotation, ambient RNA removal, and cluster purity assessment.

This study used ovarian normal tissue. I would suggest considering using the fallopian tube as the normal tissue as the fallopian tube epithelium is regarded as the cell of origin for most HGSOC cases.

We appreciate the suggestion but normal data was not explored in this manuscript in order to compare with malignant cell data. Besides, this kind of tissue was not available in the public data when the project started.

The authors should address the likelihood that the observed M1/M2 continuum might stem from single-cell data noise or artificial impacts from preprocessing, particularly during the data integration phase.

Thank you for raising this point regarding the potential impact of single-cell data noise and preprocessing artifacts on the observed M1/M2 macrophage continuum.

Firstly, it is important to recognize the inherent complexity in macrophage phenotypes as revealed by recent omics studies. The traditional dichotomy of M1 and M2 polarization, largely based on in vitro and ex vivo models, is increasingly understood to be an oversimplification. Current research, particularly in the field of tumor-associated macrophages, underscores the molecular diversity and multifunctionality of macrophages (Ma, Black, and Qian 2022b). These studies challenge the notion of fixed macrophage polarization, revealing the dynamic and context-dependent nature of these cells, influenced by a myriad of factors including the microenvironment, cytokines, and metabolic and epigenetic regulation (Murray 2017).

In our study, we have been meticulous in our approach to data integration and preprocessing to minimize the introduction of artifacts. We leveraged well-established M1 and M2 markers, as documented in the literature (Zhao et al. 2019; Boutilier and Elsawa 2021), to investigate the polarization profiles of macrophage subpopulations. Our analyses identified profiles such as RTM_IFN and Mac_ES, which align more closely with the M1 phenotype, and RTM_LA and Mac_LA, which exhibit M2 characteristics. Significantly, a majority of macrophages displayed an intermediate profile (as depicted in Supplementary Figure 8c), a finding in line with the observations made by Sathe et al. (Sathe et al. 2020).

To address the concern about data noise and preprocessing artifacts specifically, we employed multiple rigorous quality control measures and normalization techniques. Furthermore, our validation using independent datasets for both ovarian and breast tumors strengthens the argument against the likelihood of these findings being mere artifacts of data processing.

In conclusion, while we acknowledge the potential for single-cell data noise and preprocessing impacts, our comprehensive approach and the consistency of our findings with the existing literature lead us to believe that the observed spectrum of macrophage states is not attributable to such artifacts. Rather, it supports the emerging perspective that macrophages exhibit a broad, transcriptionally heterogeneous range of states, challenging the traditional binary M1/M2 paradigm.

Minor comment:

In Supplementary fig 2c, the legend bar appears absent.

Thank you for your suggestion. We have added the legend bar in In Supplementary fig 2c.

At Line 309, the authors have referred to certain cell states validated by other research groups. It would be valuable to explicitly say which of these states have been validated by other research groups and which ones are newly identified by the current study. Integrating this information into a diagram (e.g. Fig 4j) will be helpful.

Thank you for your concern. We added this information in Supplementary Table 3.

While Fig4j is quite informative, the text size of this figure and others could be increased for better readability. Moreover, incorporating details on which cell states have been experimentally validated previously and which have not would enrich the diagram.

Thank you for your concern. We modified the text size in Figure 4j.

Reviewer #4 (Remarks to the Author): expertise in immune subtypes in scRNA-seq data

Guimaraes et al conducted pan-cancer single cell analysis on tumor-associated myeloid derived cells (MDCs) in 7 different tumor types with 3 different platforms, identifying some subpopulations with possible prognostic value. Most of the findings have been reported by a published pan-cancer single cell transcriptional atlas of tumor infiltrating myeloid cells (Cheng et al, Cell 2021). Given this study included a smaller sample size, careful comparison between this study and the previous pan-cancer MDC analysis should be conducted to indicate the possible novelty of this study.

Major
1. This study integrated 7 different tumor types (6 epithelial tumor types and melanomas). The sample size is relatively small to exclude the batch effect of different studies. At least two studies are needed for each cancer type to exclude the possible bias of the studies included. What is the criteria to select the studies included in this study? If the authors do not want to discuss the differences and shared characteristics between epithelial tumor and non-epithelial tumor, it is better to delete the melanomas related study.

We acknowledge your consideration. In Supplementary Tables 1 and 2, we have outlined all the studies incorporated in our research. In summary, we were able to integrate data from 8 datasets, comprising a total of 149 samples and 392 thousand cells. This sample size is in line with other notable integrative studies, such as the "Integrated multi-omics single-cell atlas of the human retina" (Li et al. 2023), which incorporated data from 8 studies, totaling 144 samples and 2 million cells. Addressing your concern about batch effects due to the sample size, we employed robust methodologies for batch effect control, as detailed in the response to reviewer 2. The efficacy of our batch effect mitigation was evaluated using the scib benchmarking tool (Luecken et al. 2022), ensuring the reliability of our integrative analysis.

The selection criteria for these studies were established based on the availability of single-cell datasets at the project's initiation. The majority of tumor types were thoughtfully chosen, with at least two studies representing each (e.g., breast cancer - three datasets, uveal melanoma - two datasets, ovary - two datasets, lung - three datasets, melanoma - two datasets, liver - two datasets from one study, comprising smart-seq and 10x). Only skin and colorectal data were represented by one single source.

In response to your suggestion to exclude the melanoma-related study, we appreciate your perspective. While we acknowledge the distinct cell-type progenitors for malignant cells in melanoma, we recognize its relevance as a valuable model for studying the tumor microenvironment profile. This aligns with the primary focus of our study, particularly considering the high tumor mutational burden associated with melanoma. We believe the inclusion of melanoma enriches the diversity of our dataset and contributes valuable insights to the broader understanding of tumor microenvironments.

2. Three different sequencing platforms were included, although the main clustering is similar between different platform, subpopulation and molecular alterations may be impacted by different platforms.

We appreciate your observation regarding the use of three different sequencing platforms in our study. Although these platforms generally yielded a consistent main clustering pattern, we acknowledge the potential for variations in subpopulation identification and molecular alterations due to platform-specific technical differences. In response, we've implemented robust measures to mitigate these effects, detailed in the Methods section of our manuscript. This includes treating platform variation as a batch effect in our analyses, thus accounting for

any discrepancies that might arise. Additionally, we've enhanced transparency by including supplementary figures (particularly Supplementary Figure 5) to demonstrate the distribution of macrophage subpopulations across these platforms, confirming that none were specific to any single platform. These steps, along with our comprehensive validation and robustness checks, including cross-referencing with external datasets, ensure that our findings are not significantly skewed by the choice of sequencing platform, thereby maintaining the reliability and generalizability of our results.

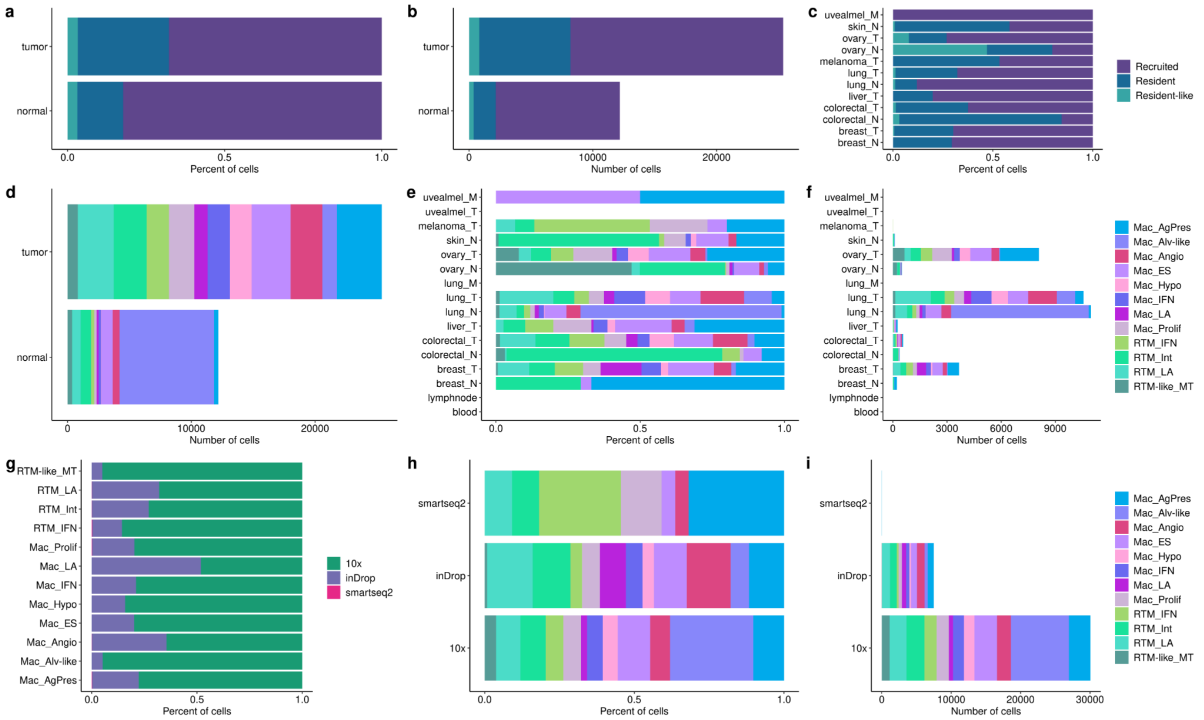

Supplementary Figure 5. Macrophages' distribution across tumor samples. (a-c) Bar plot showing the distribution of monocyte-derived and resident-macrophages across (a-b) samples and (c) conditions. (d-f) Bar plot showing the distribution of Mac subpopulations across (d) samples and (e-f) conditions. Bar plot showing the distribution of Mac subpopulations across (g-i) technologies .

3. Findings on several cell types (e.g., mast cells, FOLR2+ TAMs) have been reported in the previous study (Cheng et al, Cell 2021), what is the difference or novelty of this study on these cell types?

Thank you for highlighting the importance of distinguishing our findings from those reported by other studies. Indeed we have included a full section of analysis to highlight the commonalities and differences between the populations found in our study and by (Cheng et al. 2021) as well as (Mulder et al. 2021), which was initially presented through Supplementary Figure 7.

Our study employs a distinct methodology from Cheng et al. 2021. We integrated all tumor types before cell type analysis, enhancing the identification of rare cell types, such as the RTM-like-mt. This approach allows for a comprehensive examination of the tumor microenvironment. Other differences can be pointed out such as within the monocyte subpopulation, our analysis reveals greater diversity, including two distinct $CD14^{+}$ monocyte subpopulations (e.g. FOS+ and FOS-), two intermediate monocyte subpopulations (MonoInter_CLEC10A and MonoInter_CXCL10), and exclusive subpopulations expressing

FCGR3A (Mono_FCGR3A) and IL1B (Mono_IL1B), in contrast to Cheng et al. that identified two groups of monocytes named classical CD14hiCD16− and non-classical CD14+CD16hi. Among dendritic subpopulations, our study goes beyond the identification of well-described populations like cDC_LAMP3 and cDC1_CLEC9A, which were also noted by Cheng et al. In addition to these, we unveil a novel subpopulation, that we named as cDC2_CXCL8, unexplored in Cheng et al.'s work. This unique contribution expands the understanding of dendritic cell diversity within the tumor microenvironment.

Furthermore, our analysis extends into functional signatures within resident macrophage profiles, such as Lipid-associated metabolism and Interferon signatures followed by detailed functional analysis that associates all identified cell subpopulations with overall survival using deconvolution methods. Additionally, our main findings were validated through immunohistochemistry analysis using two Brazilian cohorts. The outcome showed for the first time the association of RTM-IM subpopulation with poor overall survival in TNBC.

In essence, the novelty of our study lies within the characterization of a greater collection of MDCS profiles, including rare ones, and an in-depth functional analysis of these cell types within the tumor microenvironment, exploring their clinical implications, and validating our findings through immunohistochemistry using independent cohorts of individuals with genetic backgrounds (Brazilian cohort) not represented at the Chen et al. work. While Cheng et al. provided a foundational understanding, our work builds upon it to offer a more nuanced and clinically relevant perspective on the roles of these cells in cancer.

4. Whether the author check if the TREM+ TAMs can match to a certain cell type with another marker co-expressed with TREM in previous study, if no, explain why, if yes, what is the difference of novelty of this study on this cell type.

Thank you for raising this point. In our analysis, we distinguished and deeply characterized several TREM2+ TAM subpopulations, including Mac_LA, Mac_AgPres, RTM_LA, and Mac_IFN. Our previous analysis showed that the Mac_LA population, characterized by the highest *TREM2* expression, did not demonstrate high correlation with populations identified in Cheng et al.’s study (Fig 4j). However, the other TREM2+ subpopulations (Mac_AgPres, RTM_LA, and Mac_IFN) showed a strong correlation with two specific populations identified by Cheng et al., namely Macro_GPNMB and Macro_C1QC, both of which exhibit high TREM2 expression according to our analysis.

To further explore the distinctions and similarities between these populations, we conducted a comparative analysis of the gene signatures and co-expression patterns. We observed that Macro_GPNMB and Macro_C1QC subpopulations, and in less extension also Macro_FN1 and Macro_IL1B, all identified by Cheng et al., express genes related to lipid metabolism such as *LPL, APOE,* and *LIPA,* which are also found in our high *TREM2* populations (Mac_LA and RTM_LA). Additionally, these subpopulations express genes associated with antigen presentation, such as *CX3CR1* and *HLA-DPA*. This suggests that the phenotypes reported by Cheng are somewhat composite, encompassing elements of both lipid-associated (LA) signatures and antigen-presentation characteristics, which our study has delineated more distinctly. Furthermore, our analysis also identified FOLR2, a marker of residency, as a distinguishing factor for these populations. This adds a layer of novelty to our research, as it suggests a more nuanced and refined classification of TAM subpopulations and their functional signatures made by our group. In addition, in response to the previous reviewer, we have updated our manuscript to acknowledge previous studies that have identified similar cell populations. This is reflected in the revised macrophage diagram (Figure 4j) and a new supplementary table (Supplementary Table 3), which provides a comprehensive overview of prior research findings related to similar subpopulations. This approach underscores our commitment to situating our findings within the broader context of

existing literature, while also highlighting the unique contributions of our study in terms of detailed subpopulation characterization and the integration of residency markers.

A

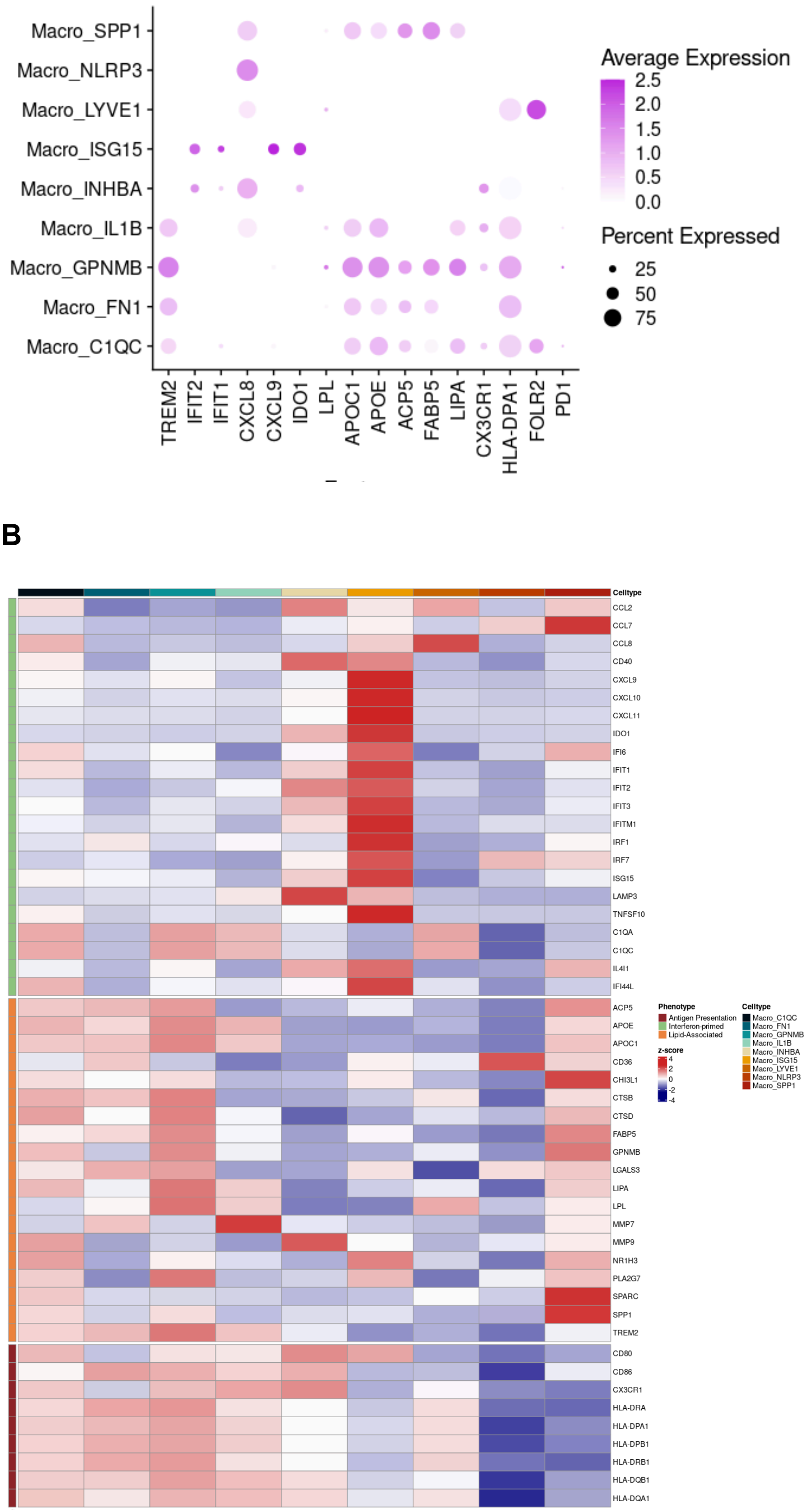

Figure for reviewers 2: Depicting functional signatures in other macrophage populations identified by Cheng et al. 2020. Panel A depicts a dot plot visualizing the expression levels of markers genes related to TREM2+ TAM subpopulations, including Mac_LA, Mac_AgPres, RTM_LA, and Mac_IFN across the macrophage subpopulations identified by Cheng et al. 2020, with dot size indicating the percentage of cells expressing the gene and color intensity representing average expression level.

Panel B presents a heatmap displaying the z-score normalized expression of Antigen presentation, Lipid-associated metabolism and Interferon-primed genes signatures across the macrophage subpopulations, with color gradients reflecting gene expression levels from low (blue) to high (red).

5. Low quality cells in Fig S1c should be deleted before performing clustering.

Thank you for your comment. The low-quality cells have been excluded as reported in the methodology section. The low-quality cluster shown in Fig S1c was placed for representational purposes only, as done by Qian et al. However, to make it clear, we have added this info in the Supplementary Figure 1 legend.

6. Figure 1b, Y axis represents tumor type, but PBMC is not a tumor type mentioned in this study. In addition, PBMCs were not mentioned at all in the result and discussion part, what is the point to add a small number of PBMC cells in this study.

Thank you for your valuable feedback. The addition of PBMCs was intended to enrich the monocytic population, primarily to serve as a reference for performing pseudotime analysis, particularly in the context of monocyte-derived macrophage differentiation.

7. Subpopulation displayed in Figure 1g is important for the subsequent analysis in this study, illustration with Figure 1g is not enough.

We apologize but we couldn't understand your comment and we would like further clarification. Could you please provide additional details or examples to help us better understand your suggestion?

8. Neutrophil is fragile, and the proportion of neutrophil can greatly impact by the process of operating single cell preparation. Because this study integrated the public resource of single cell data with different handling situation, it is not suitable to investigate the neutrophils.

We appreciate your insight into the fragility of neutrophils and the potential impact of the single-cell preparation process on their proportions. We have taken into account the capabilities of the technologies used in our study, specifically inDrop and Smart-seq, which have been shown to effectively recover neutrophils. These methods are acknowledged for their efficiency in capturing a wide range of cell types, including fragile populations like neutrophils, as evidenced in previous publications (Zilionis et al. 2019; Salcher et al. 2022). Consistently, in our dataset, neutrophils were predominantly retrieved from inDrop (n = 11823), followed by 10x (n = 117) and Smart-seq2 (n = 93). However, we acknowledge the possibility of bias, especially in the enrichment of neutrophils in specific tumor microenvironments like the lung. In response to this, we have included additional data in Supplementary Figure 2 (h-k) of our manuscript and added a paragraph in the discussion (lines 598-601) that highlights the potential biases in neutrophil recovery and emphasizes the need for cautious interpretation of our results concerning neutrophil analysis:

"Nevertheless, the absence of neutrophils in normal tissues and other tumors used in this work might introduce a potential bias, since most of the data came from InDrop and SmartSeq technology, which did not include all the tissues analyzed, leading to the observation of only pro-tumorigenic activities in these cells."

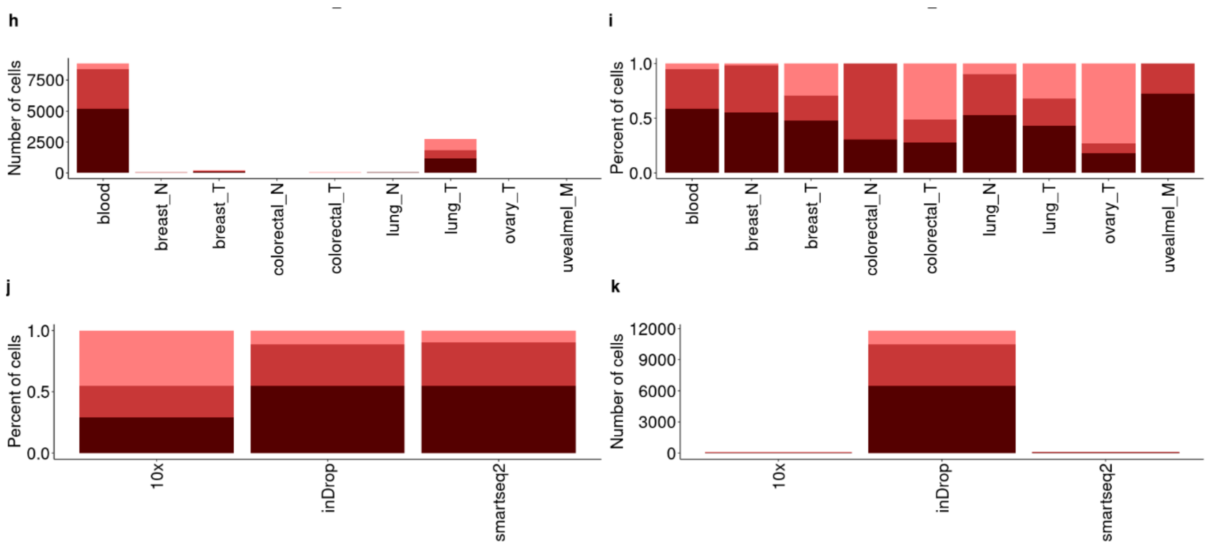

Bar plot showing the distribution of the number of neutrophil subpopulations across (f-g) sample and (h-i) tumor types, and (j-k) technologies.

9. Figure 2a is redundant, and annotation of some DC cells may be incorrect. For instance, positive rate of CD1C is too low for cDC in Figure 1e, suggesting an involvement of cDC1, cDC2, pDC and moDC. Additionally, cDC2_CD14 is moDC in Figure 2c, further support the mislabeling of Figure 1

Thank you for your observations regarding the dendritic cell (DC) annotations in our manuscript. This issue was also highlighted by another reviewer, and we have taken steps to address it. We have revised the nomenclature of the DC cells to more accurately reflect their characteristics, particularly in response to your insight about the low CD1C positive rate and the mislabeling of cDC2_CD14 as moDC: "cDC2_CD14" and "cDC2_FCGR3A" by "MoDC_CD14" and "MoDC_FCGR3A". The revised manuscript now includes these changes, ensuring that the DC annotations are correct and align with the observed marker expressions.

10. Experimental validation with mIF should be conducted in clinical samples.

We appreciate your suggestion for experimental validation using multiplex immunofluorescence (mIF) in clinical samples. Regrettably, the current unavailability of mIF technology in our setting poses a constraint, preventing us from conducting this specific experimental validation. However, we would like to emphasize that we have addressed the need for validation through alternative means. In addition to the immunohistochemistry analysis in two independent Brazilian cohorts, we have validated the signatures identified in this study using other datasets representing ovarian and breast tumor microenvironments. While mIF would undoubtedly be a valuable addition, we have strived to ensure robust validation through the resources and datasets available.

Minor
1. The number of patients and samples should be included in Table S1
2. The quality of most figures is low (e.g., Figure 1a, 1f), some words are hard to read.
3. Patient number of each group should be labeled in KM curves
4. inferCNV was mentioned in the Methods part, but no CNV analysis results have been illustrated

Thank you for your valuable feedback. We took the following steps to address your minor comments:

1. We have included the number of patients and samples in Table S1 for clarity.
2. We have improved the resolution and readability of Figures 1a and 1f to ensure all details are easily interpretable.
3. Patient numbers have been added to each group in the Kaplan-Meier curves for better context.
4. The CNV analysis results, as referenced in our methods, have been added to the revised manuscript (Supplementary Figure 1c). In addition, the following were added to the manuscript, line 145: "*Malignant Cells were distinguished from Non-Malignant Cells by the median of the copy number variation (CNV) estimative considering non malignant cells from TME as reference (Supplementary Fig. 1c).*"

References

Alshetaiwi, Hamad, Nicholas Pervolarakis, Laura Lynn McIntyre, Dennis Ma, Quy Nguyen, Jan Akara Rath, Kevin Nee, et al. 2020. “Defining the Emergence of Myeloid-Derived Suppressor Cells in Breast Cancer Using Single-Cell Transcriptomics.” *Science Immunology* 5 (44). https://doi.org/10.1126/sciimmunol.aay6017.

Bassez, Ayse, Hanne Vos, Laurien Van Dyck, Giuseppe Floris, Ingrid Arijs, Christine Desmedt, Bram Boeckx, et al. 2021. “A Single-Cell Map of Intratumoral Changes during Anti-PD1 Treatment of Patients with Breast Cancer.” *Nature Medicine* 27 (5): 820–32.

Bian, Zhilei, Yandong Gong, Tao Huang, Christopher Z. W. Lee, Lihong Bian, Zhijie Bai, Hui Shi, et al. 2020. “Deciphering Human Macrophage Development at Single-Cell Resolution.” *Nature* 582 (7813): 571–76.

Boutilier, Ava J., and Sherine F. Elsawa. 2021. “Macrophage Polarization States in the Tumor Microenvironment.” *International Journal of Molecular Sciences* 22 (13): 6995.

Casanova-Acebes, María, Erica Dalla, Andrew M. Leader, Jessica LeBerichel, Jovan Nikolic, Blanca M. Morales, Markus Brown, et al. 2021. “Tissue-Resident Macrophages Provide a pro-Tumorigenic Niche to Early NSCLC Cells.” *Nature* 595 (7868): 578–84.

Cheng, Sijin, Ziyi Li, Ranran Gao, Baocai Xing, Yunong Gao, Yu Yang, Shishang Qin, et al. 2021. “A Pan-Cancer Single-Cell Transcriptional Atlas of Tumor Infiltrating Myeloid Cells.” *Cell* 184 (3): 792–809.e23.

Hornburg, Milena, Mélanie Desbois, Shan Lu, Yinghui Guan, Amy A. Lo, Susan Kaufman, Ashley Elrod, et al. 2021. “Single-Cell Dissection of Cellular Components and Interactions Shaping the Tumor Immune Phenotypes in Ovarian Cancer.” *Cancer Cell* 39 (7): 928–44.e6.

Katzenelenbogen, Yonatan, Fadi Sheban, Adam Yalin, Ido Yofe, Dmitry Svetlichnyy, Diego Adhemar Jaitin, Chamutal Bornstein, et al. 2020. “Coupled scRNA-Seq and Intracellular Protein Activity Reveal an Immunosuppressive Role of TREM2 in Cancer.” *Cell* 182 (4): 872–85.e19.

Li, Jin, Jun Wang, Ignacio L. Ibarra, Xuesen Cheng, Malte D. Luecken, Jiaxiong Lu, Aboozar Monavarfeshani, et al. 2023. “Integrated Multi-Omics Single Cell Atlas of the Human Retina.” *Research Square*, November. https://doi.org/10.21203/rs.3.rs-3471275/v1.

Luecken, Malte D., M. Büttner, K. Chaichoompu, A. Danese, M. Interlandi, M. F. Mueller, D. C. Strobl, et al. 2022. “Benchmarking Atlas-Level Data Integration in Single-Cell Genomics.” *Nature Methods* 19 (1): 41–50.

Ma, Ruo-Yu, Annabel Black, and Bin-Zhi Qian. 2022a. “Macrophage Diversity in Cancer Revisited in the Era of Single-Cell Omics.” *Trends in Immunology* 43 (7): 546–63.

Masetti, Michela, Roberta Carriero, Federica Portale, Giulia Marelli, Nicolò Morina, Marta Pandini, Marta Iovino, et al. 2022. “Lipid-Loaded Tumor-Associated Macrophages Sustain Tumor Growth and Invasiveness in Prostate Cancer.” *The Journal of Experimental Medicine* 219 (2). https://doi.org/10.1084/jem.20210564.

Molgora, Martina, Yizhou A. Liu, Marco Colonna, and Marina Cella. 2023. “TREM2: A New Player in the Tumor Microenvironment.” *Seminars in Immunology* 67 (May): 101739.

Mulder, Kevin, Amit Ashok Patel, Wan Ting Kong, Cécile Piot, Evelyn Halitzki, Garett Dunsmore, Shabnam Khalilnezhad, et al. 2021. “Cross-Tissue Single-Cell Landscape of Human Monocytes and Macrophages in Health and Disease.” *Immunity* 54 (8): 1883–1900.e5.

Murray, Peter J. 2017. “Macrophage Polarization.” *Annual Review of Physiology* 79 (1): 541–66.

Nalio Ramos, Rodrigo, Yoann Missolo-Koussou, Yohan Gerber-Ferder, Christian P. Bromley, Mattia Bugatti, Nicolas Gonzalo Núñez, Jimena Tosello Boari, et al. 2022. “Tissue-Resident FOLR2+ Macrophages Associate with CD8+ T Cell Infiltration in Human Breast Cancer.” *Cell* 185 (7): 1189–1207.e25.

Olalekan, Susan, Bingqing Xie, Rebecca Back, Heather Eckart, and Anindita Basu. 2020. “Elucidating the Immune Response in Metastatic Ovarian Cancer Using Single Cell Transcriptomics.” *Journal of Immunology* 204 (1_Supplement): 243.16–243.16.

Pal, Bhupinder, Yunshun Chen, François Vaillant, Bianca D. Capaldo, Rachel Joyce, Xiaoyu Song, Vanessa L. Bryant, et al. 2021. “A Single-Cell RNA Expression Atlas of Normal, Preneoplastic and Tumorigenic States in the Human Breast.” *The EMBO Journal* 40 (11): e107333.

Polański, Krzysztof, Matthew D. Young, Zhichao Miao, Kerstin B. Meyer, Sarah A. Teichmann, and Jong-Eun Park. 2020. “BBKNN: Fast Batch Alignment of Single Cell Transcriptomes.” *Bioinformatics* 36 (3): 964–65.

Pombo Antunes, Ana Rita, Isabelle Scheyltjens, Francesca Lodi, Julie Messiaen, Asier Antoranz, Johnny Duerinck, Daliya Kancheva, et al. 2021. “Single-Cell Profiling of Myeloid Cells in Glioblastoma across Species and Disease Stage Reveals Macrophage Competition and Specialization.” *Nature Neuroscience* 24 (4): 595–610.

Qian, Junbin, Siel Olbrecht, Bram Boeckx, Hanne Vos, Damya Laoui, Emre Etlioglu, Els Wauters, et al. 2020. “A Pan-Cancer Blueprint of the Heterogeneous Tumor Microenvironment Revealed by Single-Cell Profiling.” *Cell Research* 30 (9): 745–62.

Salcher, Stefan, Gregor Sturm, Lena Horvath, Gerold Untergasser, Christiane Kuempers, Georgios Fotakis, Elisa Panizzolo, et al. 2022. “High-Resolution Single-Cell Atlas Reveals Diversity and Plasticity of Tissue-Resident Neutrophils in Non-Small Cell Lung Cancer.” *Cancer Cell* 40 (12): 1503–20.e8.

Sathe, Anuja, Susan M. Grimes, Billy T. Lau, Jiamin Chen, Carlos Suarez, Robert J. Huang, George Poultsides, and Hanlee P. Ji. 2020. “Single-Cell Genomic Characterization Reveals the Cellular Reprogramming of the Gastric Tumor Microenvironment.” *Clinical Cancer Research: An Official Journal of the American Association for Cancer Research* 26 (11): 2640–53.

Schelker, Max, Sonia Feau, Jinyan Du, Nav Ranu, Edda Klipp, Gavin MacBeath, Birgit Schoeberl, and Andreas Raue. 2017. “Estimation of Immune Cell Content in Tumour Tissue Using Single-Cell RNA-Seq Data.” *Nature Communications* 8 (1): 2032.

Slyper, Michal, Caroline B. M. Porter, Orr Ashenberg, Julia Waldman, Eugene Drokhlyansky, Isaac Wakiro, Christopher Smillie, et al. 2020. “A Single-Cell and Single-Nucleus RNA-Seq Toolbox for Fresh and Frozen Human Tumors.” *Nature Medicine* 26 (5): 792–802.

Wu, Sunny Z., Ghamdan Al-Eryani, Daniel Lee Roden, Simon Junankar, Kate Harvey, Alma Andersson, Aatish Thennavan, et al. 2021. “A Single-Cell and Spatially Resolved Atlas of Human Breast Cancers.” *Nature Genetics* 53 (9): 1334–47.

Xiang, Chan, Min Zhang, Zhanxian Shang, Shengnan Chen, Jikai Zhao, Bowen Ding, Dong Jiang, et al. 2023. “Single-Cell Profiling Reveals the Trajectory of FOLR2-Expressing Tumor-Associated Macrophages to Regulatory T Cells in the Progression of Lung Adenocarcinoma.” *Cell Death & Disease* 14 (8): 493.

Zhang, Kaiyang, Erdogan Pekcan Erkan, Sanaz Jamalzadeh, Jun Dai, Noora Andersson, Katja Kaipio, Tarja Lamminen, et al. 2022. “Longitudinal Single-Cell RNA-Seq Analysis Reveals Stress-Promoted Chemoresistance in Metastatic Ovarian Cancer.” *Science Advances* 8 (8): eabm1831.

Zhao, Chen, Adam C. Mirando, Richard J. Sové, Thalyta X. Medeiros, Brian H. Annex, and Aleksander S. Popel. 2019. “A Mechanistic Integrative Computational Model of Macrophage Polarization: Implications in Human Pathophysiology.” *PLoS Computational Biology* 15 (11). https://doi.org/10.1371/journal.pcbi.1007468.

Zilionis, Rapolas, Camilla Engblom, Christina Pfirschke, Virginia Savova, David Zemmour, Hatice D. Saatcioglu, Indira Krishnan, et al. 2019. “Single-Cell Transcriptomics of Human and Mouse Lung Cancers Reveals Conserved Myeloid Populations across Individuals and Species.” *Immunity* 50 (5): 1317–34.e10.

REVIEWER COMMENTS

Reviewer #1 (Remarks to the Author):

The authors addressed my concerns

Reviewer #3 (Remarks to the Author):

The authors have conducted thorough work addressing the concerns about the accuracy of the myeloid-derived cell atlas in their paper. Their efforts to compare their results with past research and to validate the newly found cell states and genes against separate datasets are significant steps towards making their findings more reliable. The authors have worked to reduce potential errors and confirm their results. Below are my comments:

1) The study continues to rely heavily on a single method, scVI, for integrating data from different sources, even though it performed well in previous benchmarking studies. It is crucial to understand that no matter how well a method performs, it can still introduce errors, and its effectiveness may vary across different scenarios. This major limitation should be further discussed in the manuscript.

2) When responding to the suggestion to use additional methods for data integration, the authors chose to use BBKNN. However, BBKNN is not typically recommended for the integration of complex datasets such as those in this study. I recommend that the authors look at comprehensive studies by Luecken et al. (https://doi.org/10.1038/s41592-021-01336-8) and Sikkema et al. (https://doi.org/10.1038/s41591-023-02327-2) to identify and apply the most suitable methods for atlas-level data integration. Specifically, applying these methods to the macrophage data, a key focus of the paper, would help verify the reproducibility of the identified cell states and patterns.

3) Moreover, the paper should clearly mention the recommendation from Liu et al. (2020) that a Rogue value above 0.9 is preferred to avoid confusion. Also, following the advice from the Rogue study to calculate the purity of integrated cell populations within individual protocols/platforms would strengthen the paper's findings.

4) I suggest that the unintegrated spaces (such as the subfigure A in "Figure for reviewers 1") should be included in the supplementary figures, for readers to observe the significant changes in data structure before and after integration.

The paper offers new and interesting biological insights about hallmark signatures in macrophages and clinical implications of macrophage states. By clearly stating the biases and limitations, the authors can greatly improve the narratives of their manuscript. This would not only make the paper stronger but also help the community better understand and trust the results.

Reviewer #4 (Remarks to the Author):

Overall, Guimaraes et al. have addressed the comments and suggestions from this reviewer either perfunctorily or refused to conduct additional analysis. Their responses have rendered the study less reliable, as the authors have made several errors. Furthermore, the revised sections were not highlighted or mentioned in the response letter (e.g., lines xxx to xxx), making it difficult to follow. Below is a point-by-point review of my previous comments/suggestions.

Major Comments:

1. The authors cited an example (Li et al.) to argue that they have collected a sufficient number of samples for pan-cancer analysis. However, this comparison is flawed for several reasons. Firstly, the Li et al. study used in-house data focused on a single tissue type (retina) by using a single

transcriptomic platform, whereas the current study spans seven different tissue types across three platforms, likely introducing greater batch effects. Secondly, the Li et al. study did not incorporate data from other studies.
For comparison, a pan-cancer study by Cheng et al. (Cell 2021) analyzed 338 samples across 15 cancer types, identifying 138,161 myeloid cells (excluding neutrophils), compared to 71,622 cells (including 12,033 neutrophils) from 138 patients in the present study. Additionally, none of the studies included in the current analysis were published after 2020, indicating that the authors conducted a similar analysis with a smaller sample/cell size three years after the initial study (Cheng et al., Cell 2021) without incorporating new or in-house data. Moreover, the revised Supplementary Table 1 reveals that one study included only one patient, three studies included 3 or 5 patients, and more than 60% of the studies included fewer than 10 patients. Regarding cancer types, three studies focused on breast cancer using three different platforms, but the reproducibility of the main findings across different data sources has not been demonstrated for breast cancer or any other cancer type. For lung cancer, three studies were included, one of which only involved normal samples. This raises the question of whether the observed differences between normal and tumor samples are due to natural variation or batch effects. Therefore, the selection of studies was NOT "thoughtfully chosen," and the claim of identifying rare and novel populations in this study is dubious given the small sample and cell sizes.
Considering the reliability of the integrative analysis, the authors' response was simply referred to as "as detailed in the response to reviewer 2." Firstly, I found no response to reviewer 2, leading me to assume the authors meant Reviewer #3. Secondly, disparate clustering patterns were observed with different integration methods (e.g., BBKNN and scVI), indicating that the integrative analysis results are heavily dependent on the bioinformatic tools used, thus significantly reducing the study's reliability. Alternative integration strategies, such as fastmnn and harmony, should be explored to address these inconsistencies. Moreover, the similarity between the patterns of the unintegrated dataset and the integrated dataset by BBKNN suggests the use of the same integration strategy with different parameters rather than comparison between unintegrated and integrated dataset.
As a suggestion, including a validation pan-cancer cohort with multiple recently published scRNA-seq studies (no fewer than those in the previously published pan-cancer study) could help validate the novel findings of this study. For instance, a recent pan-cancer scRNA-seq study included 1,223 samples for analysis on NK cells (PMID: 37607536), almost ten times the number included in this study, and the scRNA-seq data from these samples are publicly available for validation.

2. With three different platforms included, it's crucial to determine whether the main findings of this study are replicable across all platforms. The newly provided Figure S5i shows a very small number of cells from smart-seq. Given that 38 out of 138 patients (27.5%) in this study are based on smart-seq, it raises the question of whether the findings can be validated with samples sequenced by this platform. Additionally, significant platform-based bias, as indicated in Supplementary Figures 1b, 2j-2k, etc., suggests that proportion-related analysis may be significantly impacted by different platforms, rendering it unreliable.

3. The rationale for integrating all tumor types before cell type analysis to enhance the identification of rare cell types is unclear. If there is a reference, it should be cited; if not, the same process as in the previous study should be used, and cells selected through these two different approaches should be compared to validate this hypothesis. Some subclusters (e.g., cDC2_CXCL8) have been identified, but it remains to be seen whether they are real or can be validated. Additionally, how the study defines rare clusters, the number of cells in the smallest subcluster, and how to confirm their actual existence rather than being artifacts of noise or incorrect integration across different platforms require clarification. The cDC2_CXCL8 subcluster's low CD1C/CLEC10A expression raises questions about its classification. Despite being named cDC2_CXCL8, CXCL8 expression was not evident in Figure 2b. Furthermore, the genetic background is not a focal point of this study, given that the patients included in this pan-cancer analysis are not Brazilian.

4. More discussion is needed on why the novel subcluster was not identified in Cheng et al.'s work, which included more cells than the current study.

5. Re-clustering after filtering low-quality cells may affect the cluster pattern. Since all tumor types

were integrated before cell type analysis, it is unclear whether re-clustering was performed after filtering low-quality cells. If so, a new UMAP figure should be provided.

6. According to Supplementary Table 1, PBMCs were derived from only one patient in one study, which included no tumor samples. Comparing PBMCs from a healthy donor in one study with tumor samples from other studies is meaningless and introduces potential bias from batch effects.

7. Figure 1g, key for subclustering separation criteria, lacks detailed explanation in the main text, such as how each subcluster is defined, the cell number in each subcluster, and the gene markers for each subcluster.

8. Are the neutrophils primarily derived from blood (Supplementary 2h)? Which study provided blood cells according to Supplementary Table 1? The total cell number of neutrophils is very small in normal samples, and the tissue types between normal and tumor samples differ, with the proportion of CXCL8+ neutrophils varying across different platforms (e.g., high in 10x). These factors hinder drawing the conclusion that "This last population was found to be proportionally more prevalent in tumors than in blood or normal tissues, particularly in ovarian and colorectal tumors." As a suggestion, the entire neutrophil section should be excluded from the manuscript, as no reliable results have been presented.

9. Figure 2a is redundant, as Figure 2c provides the subcluster information. Moreover, the location of cells in UMAP can partially reflect the relationship distance between different subclusters. However, the positioning of moDC in the middle of different cDC2 subclusters suggests that different cDC2 subclusters are more similar to moDC rather than to each other, indicating potential issues in the integration process. A thorough check of the matrix is necessary, for example, the number of coding genes listed in the matrix file (~25000 or significantly more).

10. Dual-staining IHC is acceptable for validation (as shown in Figure 6g), and similar experiments should be conducted for FOLR2+ macrophages. Additionally, how to estimate the abundance of Mac_LA and RTM_INT in TCGA is lacking in method part? Considering many non-macrophage cells exhibit high expression of TREM2 according to Figure 6g, why use TREM2+/FOLR2+ protein levels rather than TREM2+/FOLR2+ macrophage levels for OS/PFS analysis?

Minor Comments:

1. The number of myeloid cells, besides the total cells included in this study, should be presented in Supplementary Table 1.
2. The font is still small in many figures, making it difficult to read.
3. Since the number of different groups was not even, what criteria were used to separate different groups?
4. What is the cell type of malignant cells? Were TME cells included?

RESPONSE TO REVIEWERS' COMMENTS

We are grateful for the reviewer's constructive comments and valuable observations.

Reviewer #1 (Remarks to the Author):

The authors addressed my concerns

Reviewer #3 (Remarks to the Author):

The authors have conducted thorough work addressing the concerns about the accuracy of the myeloid-derived cell atlas in their paper. Their efforts to compare their results with past research and to validate the newly found cell states and genes against separate datasets are significant steps towards making their findings more reliable. The authors have worked to reduce potential errors and confirm their results. Below are my comments:

1) The study continues to rely heavily on a single method, scVI, for integrating data from different sources, even though it performed well in previous benchmarking studies. It is crucial to understand that no matter how well a method performs, it can still introduce errors, and its effectiveness may vary across different scenarios. This major limitation should be further discussed in the manuscript.

Thank you for your comments and for acknowledging the efforts we have made to enhance the reliability of the myeloid-derived cell atlas. We recognize the concern regarding our reliance on scVI for data integration and the potential for method-specific biases.

In response to your comment, we have supplemented our analysis with additional validation using alternative integration methods, such as Scanorama, Harmony, FastMNN, BBKNN and scanVI, to provide a comparative perspective on the integration results (please check the answer for the second point raised -

Supplementary Fig 6). This helped to highlight the consistency of the cell states and gene signatures identified by scVI, and how some of them are conserved regardless of the methodology used, thereby reinforcing the reliability of our findings.

We have also expanded our discussion in the manuscript to address this concern more thoroughly. We now include a section that elaborates on the inherent limitations associated with the exclusive use of a single computational tool, in this case, scVI, for data integration. We acknowledge that despite scVI's robust performance in previous benchmarking studies, the potential for method-specific biases and errors cannot be entirely eliminated.

On line 606 we have added: *This diversity introduces dataset-specific batch effects. The selection of scVI as the integration tool was based on comprehensive analyses, which included the application of various integration platforms and the evaluation of metrics from recent benchmarking integration studies (Luecken et al. 2022; Ryu et al. 2023; Sikkema et al. 2023). These studies highlight the importance of selecting an appropriate data integration method to ensure the reliability of the resulting atlas. scVI (Lopez et al. 2018), employing variational autoencoders, is recognized for its capability to process complex datasets by identifying latent variables and conserving biological variance. Nonetheless, scVI may not completely eliminate batch effects or may inadequately delineate batches within certain cell types, reflecting a trade-off between batch effect correction and the preservation of biological diversity. To bolster the robustness and generalizability of our findings, cross-validation with alternative integration methods was performed, and results were corroborated using independent datasets.*

2) When responding to the suggestion to use additional methods for data integration, the authors chose to use BBKNN. However, BBKNN is not typically recommended for the integration of complex datasets such as those in this study. I recommend that the authors look at comprehensive studies by Luecken et al. and Sikkema et al. to identify and apply the most suitable methods for atlas-level data integration. Specifically, applying these methods to the macrophage data, a key focus of the paper, would help verify the reproducibility of the identified cell states and patterns.

We appreciate your recommendation to consult the studies by (Luecken et al. 2022) and (Sikkema et al. 2023) for identifying more suitable methods for atlas-level data integration.

We agree that the complexity of our dataset warrants the application of more advanced integration methods that are specifically designed to handle large-scale and heterogeneous data. We acknowledge that while BBKNN has been a useful tool in certain contexts, its limitations in dealing with complex datasets such as ours might have restricted our ability to fully capture the nuanced relationships among cell states.

In response to your suggestion, we have revisited our data integration strategy and explored additional methods and compared them to help identify those that are better suited for atlas-level integration as highlighted in the studies by (Luecken et al. 2022) and (Sikkema et al. 2023). Specifically, we have applied these advanced integration methods to our macrophage data.

Importantly, the results from these advanced integration methods corroborate our initial findings, thus reinforcing the reproducibility and reliability of the cell states and patterns we have identified in our atlas.

We have updated our manuscript to include these new analyses and to discuss the rationale behind the choice of these methods, their implementation, and the implications of the findings obtained through their application. We also added a new Supplementary Figure 6, that includes the main benchmark results, as follows:

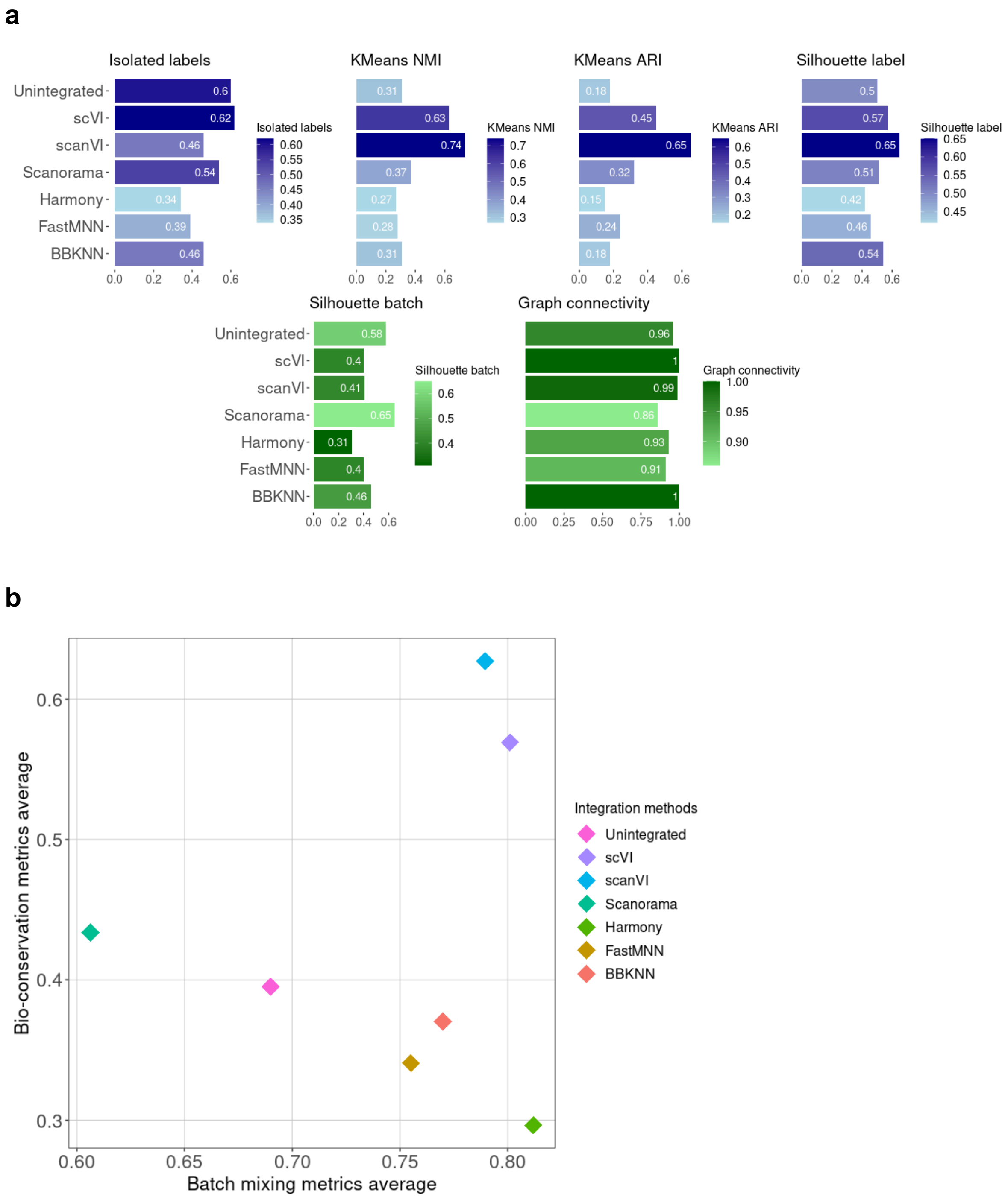

Supplementary Figure 6: Performance metrics for different data integration tools applied to the myeloid-derived cells. a) Each bar chart corresponds to a specific metric used to assess the quality of the data integration described in the methodology section. The color code in the figure corresponds to the performance of each integration tool on the respective metric. Darker colors indicate better performance, while lighter colors represent poorer performance b) Scatter plot of the bio-conservation metrics average versus batch mixing metrics average for all the integration methods used in this work.

In light of the new benchmark result, we have added the following paragraph:
Line 274: *To ascertain the optimal method for mitigating batch effects while conserving the biological variation inherent to distinct cell types, we conducted an extensive analysis of six data integration methods on the macrophage data (Supplementary Figure 6A), employing the benchmarking framework outlined by (Luecken et al. 2022). This framework assesses the quality of integration, the retention of cell type-specific variation, and the preservation of biological meaning within the integrated data. When considering the metrics laid out by this framework, Harmony (Hie, Bryson, and Berger 2019; Korsunsky et al. 2019)excelled in batch correction but fell short in maintaining cell identity. In contrast, both scVI (Lopez et al. 2018)and scANVI (Xu et al. 2021)achieved high rankings in conserving cellular biology. Among these, scANVI marginally outperformed scVI, benefiting from the use of pre-existing cell type annotations to refine cell type distinctions post-integration. Despite this, scVI was chosen for further analyses due to its robust capability to process unlabeled data, striking a balance between effective integration and biological fidelity (Supplementary Figure 6B). Employing scVI, we generated an embedding that accurately integrates and represents all 37891 macrophage cells from 11 studies across our datasets (Supplementary Figure 6C).*

We also added a new section in the methodology to cover the "Integration Benchmarking" topic (line 808).

3) Moreover, the paper should clearly mention the recommendation from Liu et al. (2020) that a Rogue value above 0.9 is preferred to avoid confusion. Also, following the advice from the Rogue study to calculate the purity of integrated cell populations within individual protocols/platforms would strengthen the paper's findings.

In response to this feedback, we have updated our manuscript and figures to better align with the recommendations from (Liu et al. 2020) regarding Rogue values. Specifically, we now evaluate the purity of integrated cell populations across different protocols and platforms and emphasize the necessity of achieving Rogue values greater than 0.9 to ensure the clarity and reliability of cell population assignments, reinforcing this guidance directly within our text.

I

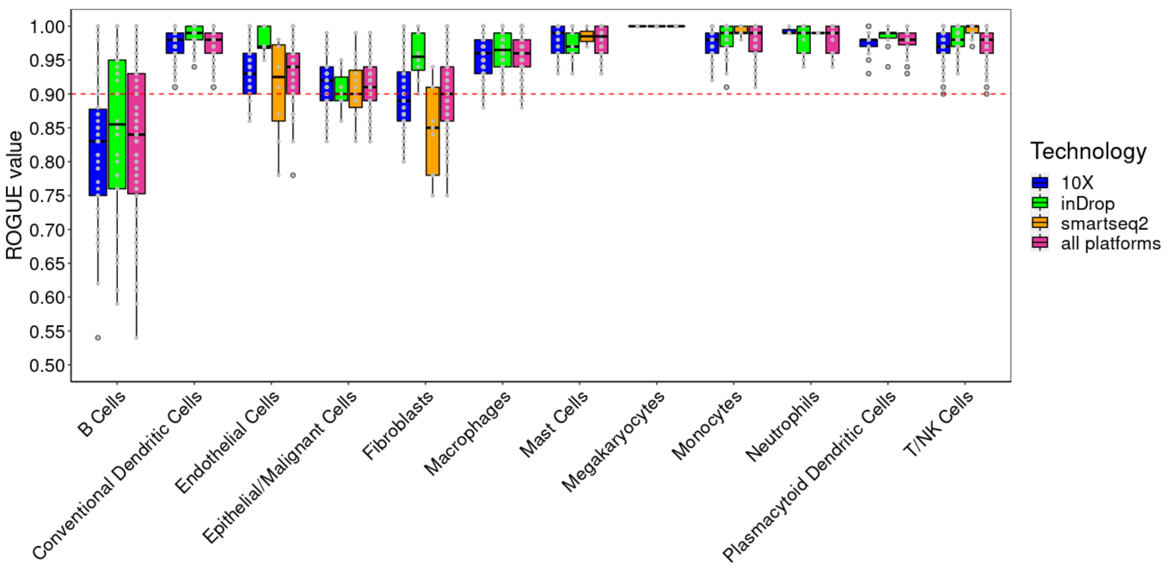

J

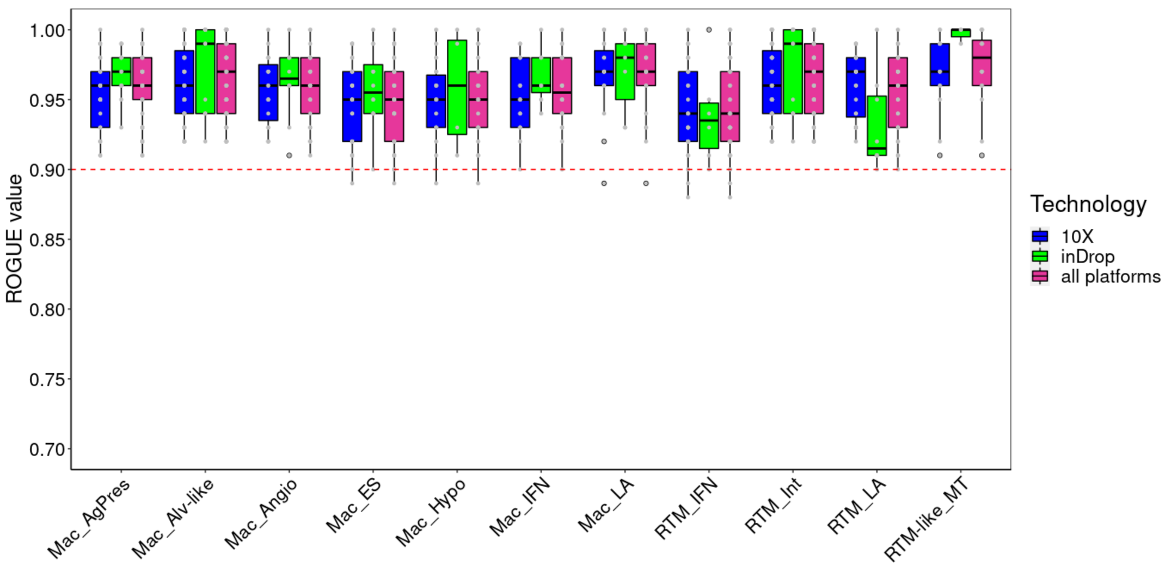

Supplementary Figure 1: Purity of (i) broad cell type clusters and (j) macrophages post-scVI integration, evaluated using ROGUE scores. Points represent individual samples, with ROGUE values above the recommended threshold of 0.9 (dashed line) indicating high cluster purity.

We have also added in line 181 the following phrase for clarity: *(values above 0.9 are recommended as a pure cluster);*

In addition, Supplementary Figure 7i was replaced by the following plots to better account the purity of clusters separated the ROGUE values from macrophages, monocytes and cDCs of our, (Cheng et al. 2021), and (Mulder et al. 2021) datasets.

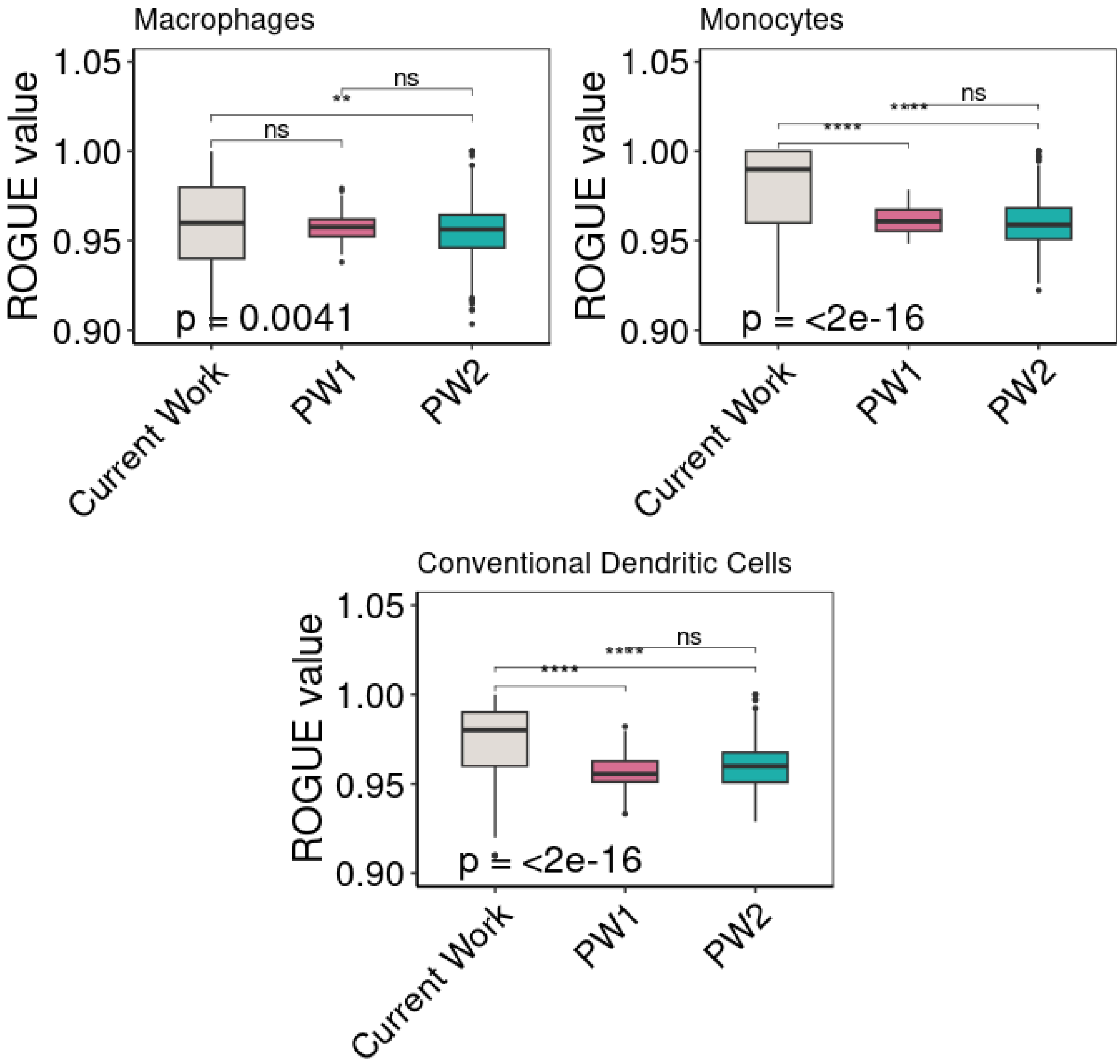

Supplementary Figure 7: Boxplot showing a comparison of ROGUE values between macrophages (i), monocytes (j) and cDC (k) populations described by this work, Cheng and colleagues (2021) work (PW1), and Mulder and colleagues (2021) work (PW2). Statistical significance for values of $p < 0.05$.

4) I suggest that the unintegrated spaces (such as the subfigure A in "Figure for reviewers 1") should be included in the supplementary figures, for readers to observe the significant changes in data structure before and after integration.

We appreciate your suggestion. In response to your recommendation, we have added the unintegrated data representation to the Supplementary Figure 6, and also the integrations performed with the other methods for comparison, as follows:

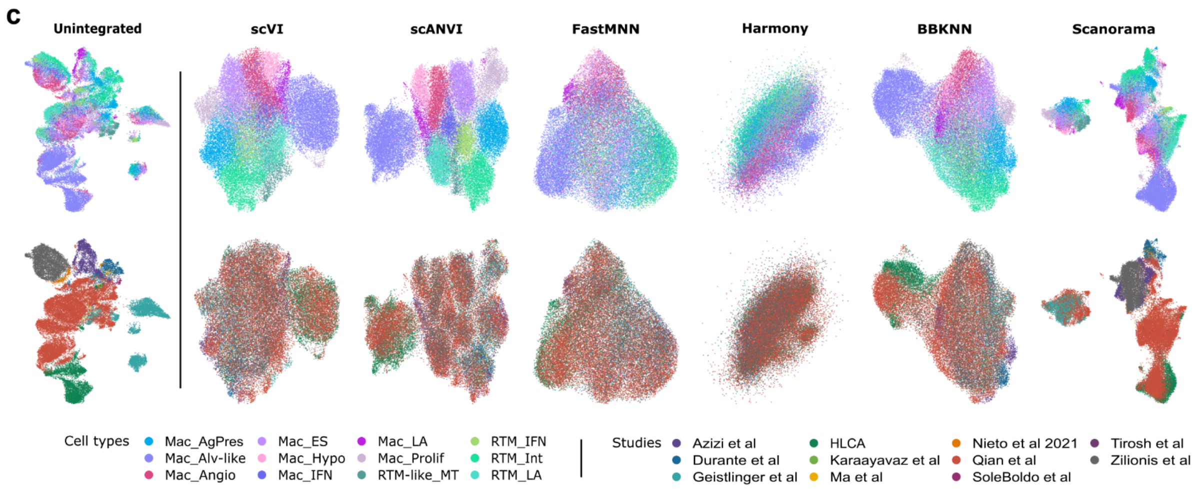

Supplementary Figure 6: Comparative Analysis of Data Integration Methods for scRNA-Seq. (c) UMAP visualizations of scRNASeq data following integration by different computational methods. Cells are color coded by cell types showing the impact of different integration methods on the preservation of cellular identities while in data is colored by the originating study, highlighting the method's ability to integrate datasets from diverse sources.

We agree that this inclusion will greatly benefit readers, offering a more complete picture of our integration approach and its effect on the underlying data structure, thereby facilitating a deeper appreciation of the methodological advancements presented in our study.

The paper offers new and interesting biological insights about hallmark signatures in macrophages and clinical implications of macrophage states. By clearly stating the biases and limitations, the authors can greatly improve the narratives of their manuscript. This would not only make the paper stronger but also help the community better understand and trust the results.

In response to this feedback, including the recommendation from Reviewer 4, we have undertaken a careful revision of our manuscript to enhance its clarity,

transparency, and scientific robustness. A significant amendment is the removal of the section on neutrophil populations, prompted by the observation that these cells are underrepresented in our dataset, acknowledging that the limited data on neutrophils may not provide a sufficiently comprehensive or accurate portrayal of their roles and states.

Furthermore, we have expanded our discussion on potential biases and limitations inherent in our study. This includes a more detailed examination of the methodological constraints associated with data integration techniques, particularly those related to the use of scVI, and how these might influence the results. We have also elaborated on the challenges posed by batch effects, sample diversity, and the granularity of cell state identification, which could impact the interpretation of macrophage hallmark signatures.

Reviewer #4 (Remarks to the Author):

Overall, Guimaraes et al. have addressed the comments and suggestions from this reviewer either perfunctorily or refused to conduct additional analysis. Their responses have rendered the study less reliable, as the authors have made several errors. Furthermore, the revised sections were not highlighted or mentioned in the response letter (e.g., lines xxx to xxx), making it difficult to follow. Below is a point-by-point review of my previous comments/suggestions.

Major Comments:

1. The authors cited an example (Li et al.) to argue that they have collected a sufficient number of samples for pan-cancer analysis. However, this comparison is flawed for several reasons. Firstly, the Li et al. study used in-house data focused on a single tissue type (retina) by using a single transcriptomic platform, whereas the current study spans seven different tissue types across three platforms, likely introducing greater batch effects. Secondly, the Li et al. study did not incorporate data from other studies.
For comparison, a pan-cancer study by Cheng et al. (Cell 2021) analyzed 338 samples across 15 cancer types, identifying 138,161 myeloid cells (excluding neutrophils), compared to 71,622 cells (including 12,033 neutrophils) from 138 patients in the present study. Additionally, none of the studies included in the current analysis were published after 2020, indicating that the authors conducted a similar analysis with a smaller sample/cell size three years after the initial study (Cheng et al., Cell 2021) without incorporating new or in-house data. Moreover, the revised Supplementary Table 1 reveals that one study included only one patient, three studies included 3 or 5 patients, and more than 60% of the studies included fewer than 10 patients. Regarding cancer types, three studies focused on breast cancer using three different platforms, but the reproducibility of the main findings across different data sources has not been demonstrated for breast cancer or any other cancer type. For lung cancer, three studies were included, one of which only involved normal samples. This raises the question of whether the observed differences between normal and tumor samples are due to natural variation or batch effects. Therefore, the selection of studies was NOT "thoughtfully chosen," and the claim of

identifying rare and novel populations in this study is dubious given the small sample and cell sizes.

Considering the reliability of the integrative analysis, the authors' response was simply referred to as "as detailed in the response to reviewer 2." Firstly, I found no response to reviewer 2, leading me to assume the authors meant Reviewer #3. Secondly, disparate clustering patterns were observed with different integration methods (e.g., BBKNN and scVI), indicating that the integrative analysis results are heavily dependent on the bioinformatic tools used, thus significantly reducing the study's reliability. Alternative integration strategies, such as fastmnn and harmony, should be explored to address these inconsistencies. Moreover, the similarity between the patterns of the unintegrated dataset and the integrated dataset by BBKNN suggests the use of the same integration strategy with different parameters rather than comparison between unintegrated and integrated dataset.

As a suggestion, including a validation pan-cancer cohort with multiple recently published scRNA-seq studies (no fewer than those in the previously published pan-cancer study) could help validate the novel findings of this study. For instance, a recent pan-cancer scRNA-seq study included 1,223 samples for analysis on NK cells (PMID: 37607536), almost ten times the number included in this study, and the scRNA-seq data from these samples are publicly available for validation.

We would like to thank all the valuable contributions made by Reviewer 4 and apologize if our first round of responses didn't meet the expectations. We appreciate the detailed comparison to related studies and the emphasis on the importance of methodological robustness and sample diversity in pan-cancer analyses. We have addressed his/her concerns point by point:

1. Comparison with other atlas studies, study design, and sample size:

We recognize the reviewer's concerns regarding the comparison with larger atlas studies and the potential for batch effects given our dataset's diversity. Our dataset, while smaller than some comprehensive studies like Cheng et al., was selected for its representativeness and diversity across a wide range of solid tumors. The inclusion of studies was driven by data availability at the beginning of the project and the feasibility of integrating datasets to investigate myeloid-derived cell states across various cancer types.

A key aspect of our approach is the method used for data integration and analysis. Unlike the methodology in Cheng et al., we performed data integration prior to cell-type identification. This approach increases our ability to identify rare cell phenotypes that might be overlooked in individual datasets, as they could be obscured by more prevalent cell states. Recent works have shown that integration strategies boost the statistical power to detect rare subpopulations (Butler et al. 2018; Stuart et al. 2019; Sikkema et al. 2023).

The strength and applicability of our findings are further supported by the validation of identified cell states and marker genes against independent datasets. These external datasets, also sourced from publicly available studies, validate our discoveries, confirming the relevance and reliability of the populations and signatures we have identified. Also in response to the reviewer's critique about new datasets, we have now included an analysis using a 3rd independent validation cohort (Supplementary Figure 10) comprising 13 additional independent datasets, 233 samples spanning 10 different tumor types and includes a substantial compilation of myeloid cells, totaling 96,514 (referenced in Supplementary Figure 10a-d and Supplementary Table 6). Our validation efforts within this dataset confirmed the existence of various dendritic cell (DC) subpopulations, notably the cDC_CXCL8 phenotype, all six previously identified monocyte subpopulations and 9 out of 10 macrophages signatures were also validated , with the exception of RTM-like_MT subpopulation. This thorough validation against independent datasets enhances the credibility of our identified macrophage groups.

The independent datasets utilized include:

	Authors	Cancer types	Number of samples	Number of myeloid cells	Platforms
Dataset 1	Cheng et al. 2021	Lung, kidney, breast, stomach, skin, liver, colorectal, nasopharynge	210	60,293	10x and Smart-seq 2

		a, esophagea, ovary or fallopian tube , pancreas, thyroid, uterine corpus , lymphoma, myeloma			
Dataset 2	Mulder et al. 2021	liver, spleen, tonsil, head/neck/tonsil, skin, colon, lung, kidney, pancreas, ascites, stomach, breast, and blood.	45	133,869	Dropseq, 10x, Smart-seq 2, CITE-Seq, CEL-seq2
Dataset 3	Olalekan et al. 2020; Slyper et al. 2020; Hornburg et al. 2021; Zhang et al. 2022 Bassez et al. 2021; Pal et al. 2021; Wu et al. 2021 Chen et al. 2024; Pu et al. 2021; Werba et al. 2023; Zhang et al. 2021; Choi et al. 2023; Jiang et al. 2022; Lee et al. 2020; e Wong et al. 2022.	Ovary breast, endometrium, thyroid, pancreas, kidney, head and neck, gastric, colorectal, and prostate.	233	96,514	10x, drop-seq

2. Integration Methodology and Reliability:

The reviewer's observation regarding the dependency of integration results on the bioinformatic tools used is well-taken and was also raised by reviewer 3. As shown by benchmarking atlas-level data integration studies in single-cell (Luecken et al. 2022; Ryu et al. 2023), the choice of the methodology should take in account the complexity of the data and the capability of the method to correct the batch effect while preserving biological variance. In light of this, we have expanded our analysis to include alternative integration strategies, such as Scanorama, Harmony,

FastMNN, BBKNN and scanVI, (please check the response 2 to the reviewer 3), to compare with scVI, a well recommended tool to perform integration, especially for sufficiently large datasets without labels. This extended analysis provided a more robust comparison of integration outcomes and enhanced the reliability of our findings. We have updated our results section (lines 274 and Supplementary Fig. 6), discussion (line 606), and methodology (line 808) in order to reflect these additional analyses and have made efforts to ensure that the comparison between unintegrated and integrated datasets is more transparent and methodologically sound.

3. Reproducibility Across Different Data Sources:

We have also addressed the concern regarding the reproducibility of our findings across different platforms (New Supplementary Figure 6). These analyses, presented in the revised figures and tables, confirm that the identified myeloid-derived cell states are reproducible across different datasets and are not artifacts of batch effects.

On line 326 we have added: Importantly, the signatures identified were reproducible among different platforms *(Supplementary Fig. 7).*

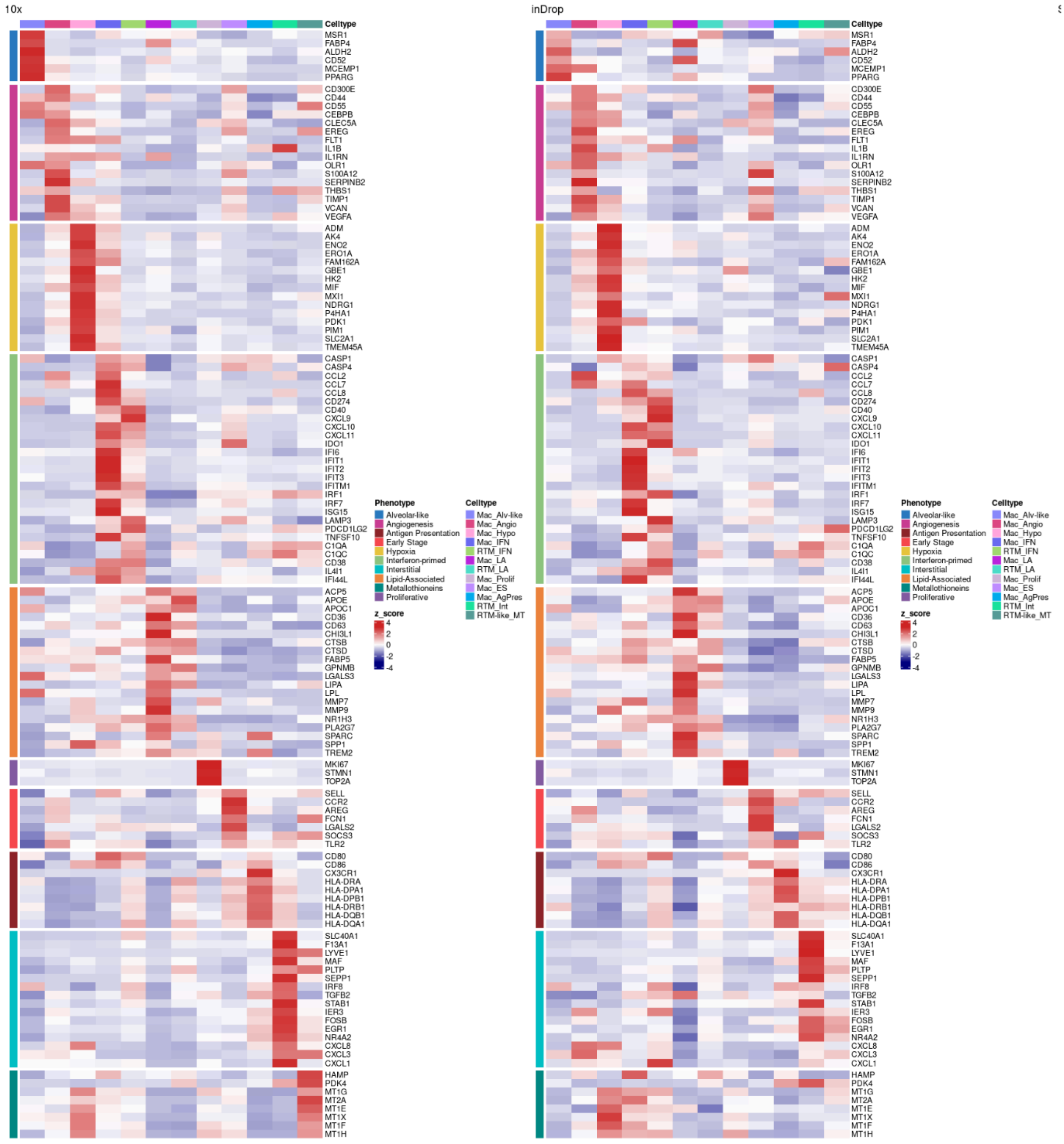

Supplementary Figure 7: Reproducibility of signatures across different platforms. Heatmap displaying the z-score normalized expression of the ten functional signatures considering a) 10x and b) InDrop technologies separated. Color gradients reflect gene expression levels from low (blue) to high (red).

4. Validation with an External Pan-Cancer Cohort:

We acknowledge the value of validating our findings using an external pan-cancer cohort. Following the reviewer's suggestion, we have sought publicly available

scRNA-seq datasets published in the last few years. This validation effort is now described in our revised manuscript as follows:

Line 369: *In order to validate our findings, we extended our analysis to include 13 independent datasets, creating an additional pan-cancer atlas. This expanded atlas encompasses a comprehensive array of samples (n=233) across 10 tumor types, incorporating a significant number of myeloid cells (n=96,514) (Supplementary Figure10a-d and Supplementary Table 6). Within this broader dataset, we validated the presence of several dendritic cell (DC) subpopulations, including the cDC_CXCL8 phenotype. The cDC4 subpopulation was the only exception, probably because this version of the atlas does not include blood samples (Supplementary Figure 10e-f). For monocytes, we successfully identified all six subpopulations as initially characterized (Supplementary Figure 10g-h). In the case of macrophages, we confirmed 9 out of 10 signatures (Supplementary Figure 10i-k). The one exception, the RTM-like_MT subpopulation, was noted for its low expression of the metallothionein gene sets, indicating a distinct transcriptional profile. Of note, Mac_Alv-like signature was not so evident, may be attributed to the lack of lung samples within our new integrated dataset. This cross-checking of our results with independent datasets strengthens the trustworthiness of our identified mononuclear phagocytes groups.*

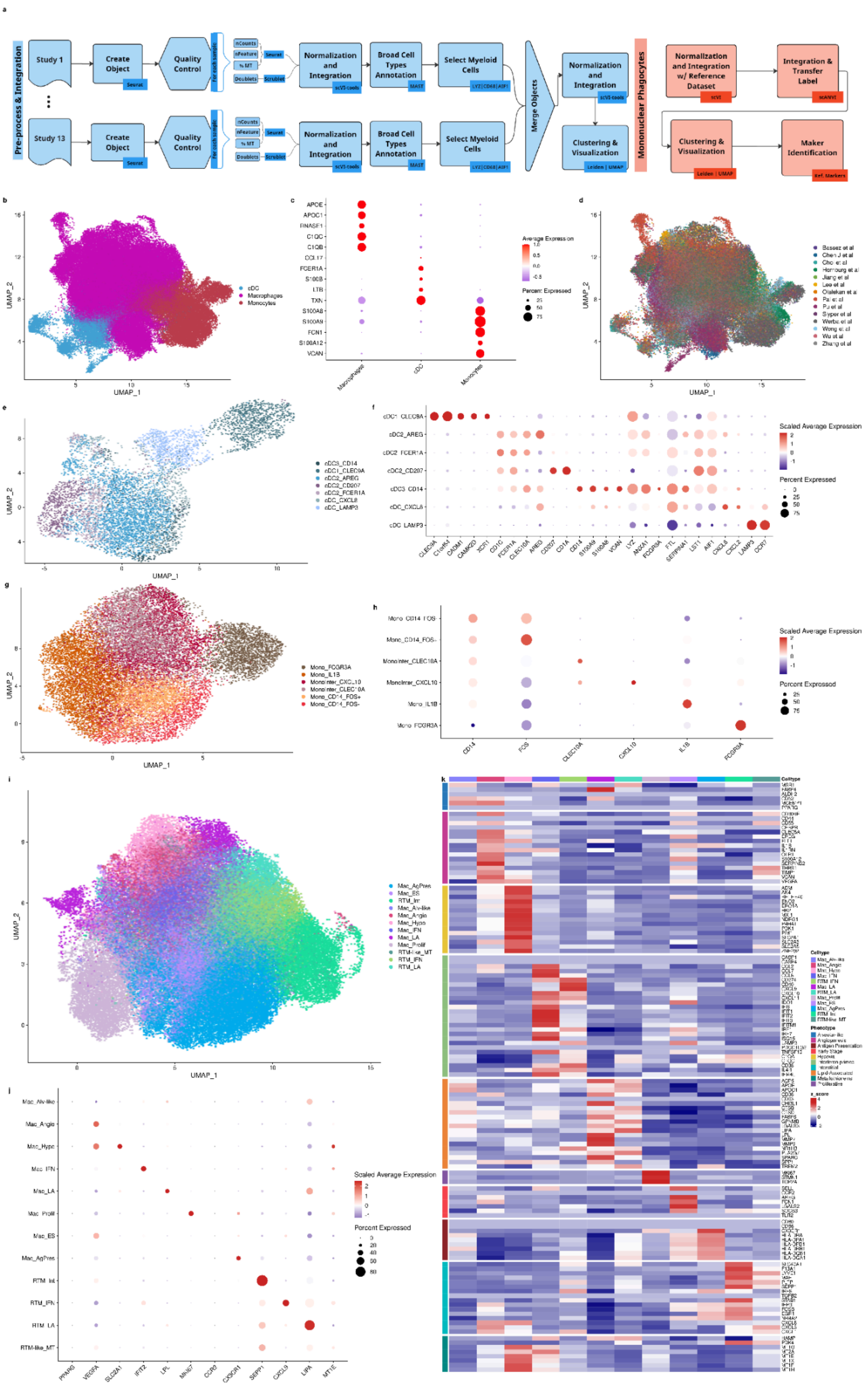
a
Pre-process & Integration
Study 1
Study 13
Create Object
Seurat
Quality Control
Normalization and Integration
scVI-tools
Broad Cell Types Annotation
MAST
Select Myeloid Cells
Merge Objects
Normalization and Integration
Clustering & Visualization
Leiden | UMAP
Mononuclear Phagocytes
Normalization and Integration w/ Reference Dataset
Integration & Transfer Label
scANVI
Clustering & Visualization
Maker Identification
b
UMAP_1
UMAP_2
cDC
Macrophages
Monocytes
c
APOE
APOC1
RNASE1
C1QC
C1QB
CCL17
FCER1A
S100B
LTB
TXN
S100A8
S100A9
FCN1
S100A12
VCAN
Macrophages
cDC
Monocytes
Average Expression
Percent Expressed
d
Basaez et al
Chen J et al
Cho et al
Hornburg et al
Jiang et al
Lee et al
Olalekan et al
Pal et al
Pu et al
Syper et al
Wong et al
Wu et al
Zhang et al
e
cDC3_CD14
cDC1_CLEC9A
cDC2_AREG
cDC2_CD207
cDC2_FCER1A
cDC_CXCL8
cDC_LAMP3
f
Scaled Average Expression
Percent Expressed
g
Mono_FCGR3A
Mono_IL1B
Monointer_CXCL10
Monointer_CLEC10A
Mono_CD14_FOS+
Mono_CD14_FOS-
h
CD14
FOS
CLEC10A
CXCL10
IL1B
FCGR3A
Scaled Average Expression
Percent Expressed
i
Mac_AgPres
Mac_ES
RTM_Inf
Mac_Alv-like
Mac_Angio
Mac_Hypo
Mac_IFN
Mac_LA
Mac_Prolif
RTM-like_MT
RTM_IFN
RTM_LA
j
PPARG
VEGFA
SLC2A1
IFIT2
LPL
MKI67
CCR2
CX3CR1
SEPP1
CXCL9
LIPA
MT1E
Scaled Average Expression
Percent Expressed
k
Celltype
Phenotype
z_score

Supplementary Figure. 10. Comprehensive validation within a novel myeloid cell pan-cancer atlas (a) Workflow for the external validation analysis. (b) UMAP of 96,514 myeloid-derived cells, color-coded according to cell types and (c) Dot plot showing the top 5 DEGs expression by each myeloid-derived subpopulation. Dot size indicates the percent of expressing cells, and the dot color is the scaled average expression. (d) UMAP color-coded according to the studies that generated each dataset. (e) UMAP of DC subpopulations colored by the 7 states identified. (f) Dot plot showing the mean expression of genes related to DC subpopulations. Dot size indicates the percent of expressing cells, and the dot color the scaled average expression. (g) UMAP of monocytes subpopulations colored by the six states identified. (h) Dot plot showing the mean expression of genes related to monocytes subpopulations. Dot size indicates the percent of expressing cells, and the dot color the scaled average expression. (i) UMAP of macrophages subpopulations colored by the twelve states identified. (j) Dot plot showing the mean expression of genes related to macrophages subpopulations. Dot size indicates the percent of expressing cells, and the dot color the scaled average expression. (k) Heatmap showing the gene signature per subpopulation. The color scale represents the scaled expression of each gene.

We hope that these revisions and clarifications address the reviewer's concerns and enhance the overall rigor and reliability of our study.

2. With three different platforms included, it's crucial to determine whether the main findings of this study are replicable across all platforms. The newly provided Figure S5i shows a very small number of cells from smart-seq. Given that 38 out of 138 patients (27.5%) in this study are based on smart-seq, it raises the question of whether the findings can be validated with samples sequenced by this platform. Additionally, significant platform-based bias, as indicated in Supplementary Figures 1b, 2j-2k, etc., suggests that proportion-related analysis may be significantly impacted by different platforms, rendering it unreliable.

We acknowledge the necessity of validating that our key findings are reproducible across different technological platforms. The point raised regarding Figure S5i, noting the sparse representation of macrophages derived from the Smart-seq platform, is well taken. However, as depicted in Figure S1b, Smart-seq has contributed significantly to the identification of other cell types within the tumor microenvironment (TME), including fibroblasts, B cells, and a subset of myeloid-derived cells. Moreover, our ROGUE analysis, which assesses the purity of clusters across the three technologies, indicates that, apart from a minor discrepancy

in the purity of endothelial cells and fibroblasts, the other cell types show comparable purity levels to those identified using other platforms (Please refer to the response to question 3 from reviewer 3 and Supplementary Figures 1i,j).

The apparent discrepancies in cell type proportions are more reflective of the diversity of tumor types that has been sequenced using each technology rather than a bias inherent to any specific platform. We have demonstrated that cell type signatures are consistent across 10x and InDrop platform, underscoring the robustness and reproducibility of these molecular signatures irrespective of the sequencing technology used (as depicted in the "**Supplementary Figure 7"**).

While the limited Smart-seq data for macrophages in our compiled dataset (and literature in general, since few cells are generally profiled with this technique) precludes a comprehensive validation of findings for this platform, the collective evidence from our analyses across other platforms bolsters the reliability of our conclusions. Additional studies specifically focusing on Smart-seq data are warranted to fully explore its compatibility with other platforms.

3. The rationale for integrating all tumor types before cell type analysis to enhance the identification of rare cell types is unclear. If there is a reference, it should be cited; if not, the same process as in the previous study should be used, and cells selected through these two different approaches should be compared to validate this hypothesis. Some subclusters (e.g., cDC2_CXCL8) have been identified, but it remains to be seen whether they are real or can be validated. Additionally, how the study defines rare clusters, the number of cells in the smallest subcluster, and how to confirm their actual existence rather than being artifacts of noise or incorrect integration across different platforms require clarification. The cDC2_CXCL8 subcluster's low CD1C/CLEC10A expression raises questions about its classification. Despite being named cDC2_CXCL8, CXCL8 expression was not evident in Figure 2b. Furthermore, the genetic background is not a focal point of this study, given that the patients included in this pan-cancer analysis are not Brazilian.

In response to the reviewer's question on the rationale for integrating all tumor types before cell type analysis to enhance the identification of rare cell types, we draw

upon recent advancements in scRNA-seq integration methodologies and literature to support our approach. The integration of diverse tumor types is strategically aimed at amplifying the statistical power necessary for uncovering and characterizing rare cell populations within tumors. This methodology is substantiated by findings that integrating various scRNA-seq technologies markedly improves the detection of rare cell phenotypes, some as scarce as 0.4%, and facilitates the identification of transcriptomic markers indicative of cell state (Butler et al. 2018).

Moreover, our rationale is further validated by empirical evidence where data integration across multiple datasets has enabled the identification of exceptionally rare cell populations, such as tissue-resident plasmacytoid dendritic cells (0.07% of cells detected) and mesothelial cells (0.05% of cells detected), which were not robustly identifiable in analyses restricted to individual datasets (Stuart et al. 2019).

In addition, the Human Lung Cell Atlas (HLCA) core's strategy of combining datasets has proven to be a powerful tool for identifying rare cell types, such as ionocytes, tuft cells, and neuroendocrine cells, among others. These cell types, with prevalence as low as 0.01%, were only identifiable through the integration of multiple datasets, highlighting the enhanced power of dataset integration to uncover rare populations that are otherwise difficult to detect in individual analyses (Sikkema et al. 2023).

In our analysis, we were able to identify myeloid-derived subpopulations in the following proportion, been cDC_LAMP3 the rarest subpopulation identified:

**Myeloid cells**	**# of cells**	**% (total)**	**% (myeloid)**
cDC_LAMP3	285	0,07	0,55
cDC4_FCGR3A	314	0,08	0,61
cDC2_CD207	505	0,13	0,98
cDC1_CLEC9A	580	0,15	1,12
cDC2_FCER1A	726	0,19	1,40
cDC3_CD14	765	0,20	1,48

cDC_CXCL8	993	0,25	1,92
RTM-like_MT	1209	0,31	2,34
Mac_LA	1230	0,31	2,38
MonoInter_FOS-	1238	0,32	2,40
Mono_CD14_FOS+	1497	0,38	2,90
MonoInter_FOS+	1559	0,40	3,02
Mono_IL1B	1739	0,44	3,36
Mac_Hypo	1798	0,46	3,48
Mac_IFN	1979	0,50	3,83
Mono_FCGR3A	2018	0,51	3,90
cDC2A_AREG	2044	0,52	3,95
RTM_IFN	2051	0,52	3,97
Mono_CD14_FOS-	2150	0,55	4,16
Mac_Angio	3103	0,79	6,00
RTM_Int	3504	0,89	6,78
RTM_LA	3569	0,91	6,91
Mac_AgPres	3949	1,01	7,64
Mac_ES	4069	1,04	7,87
Mac_Alv-like	8813	2,25	17,05
**TOTAL**	51687		100,00

To further substantiate our hypothesis, we aimed to conduct a comparative analysis focusing on macrophage subpopulations identified in Cheng's dataset. Our premise is based on the observation that despite Cheng et al., 2020 working with a more extensive pan cancer dataset than ours, they did not identify the same populations. We propose that this discrepancy arises from their approach of not integrating the data, which potentially diminishes the ability to identify more homogeneous clusters.

A closer examination of the 10 signatures in their dataset reveals that some identified populations appear to be a combination of multiple signatures (Figure 1A for reviewers), suggesting a loss of specificity in cell type identification, while for Mulder et al. 2021, whos has also has taken an integrative approach, more defined populations can be found (Figure 1B for reviewers).

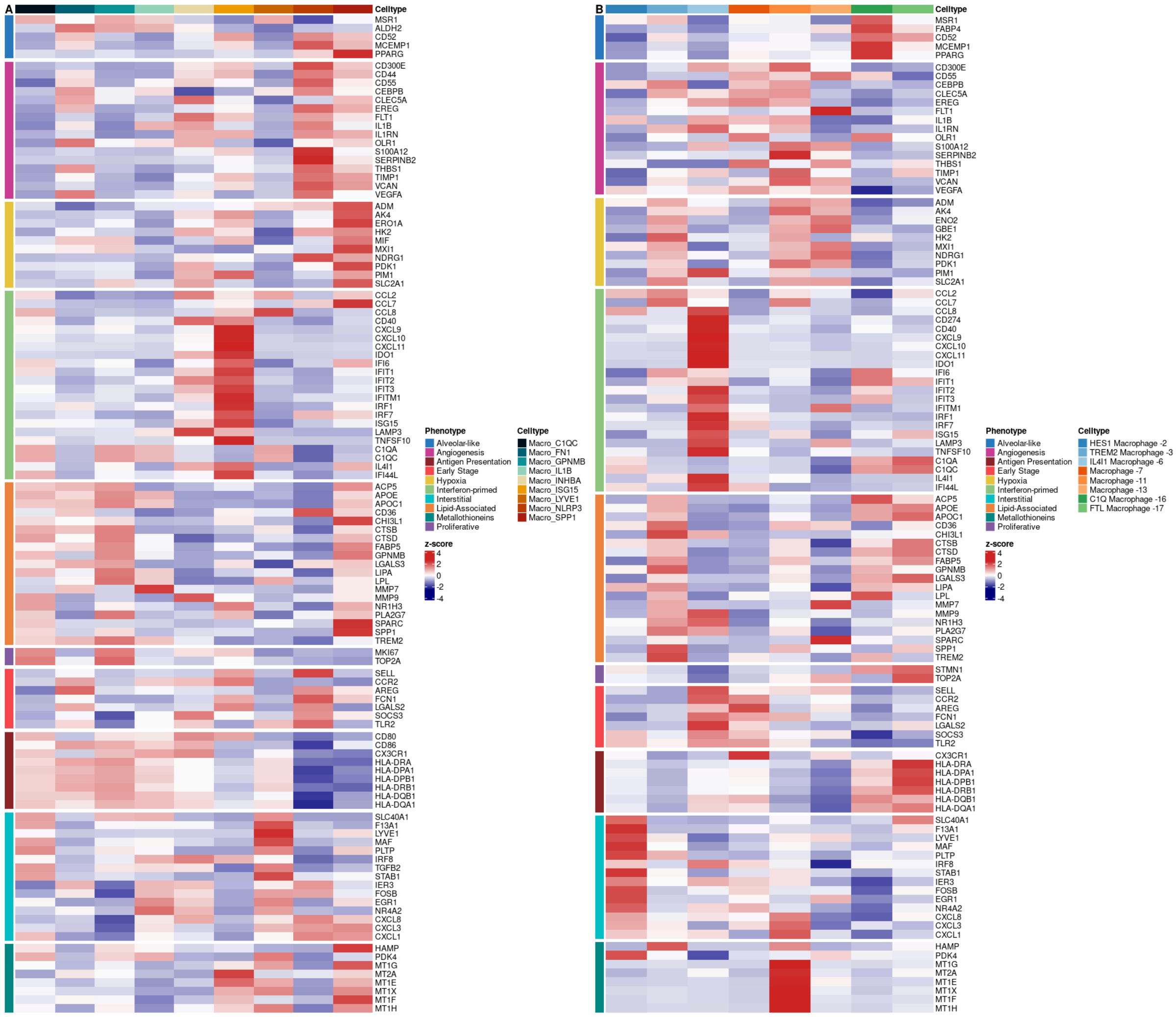

Figure 1 for reviewers: **Depicting functional signatures in macrophage populations identified by Cheng et al. 2021 (a) and Mulder et al. 2021 (b).** Heatmap displaying the z-score normalized expression of the ten functional signatures described in our work for subpopulations identified by Cheng et al. 2021 and Mulder et al. 2021. Color gradients reflect gene expression levels from low (blue) to high (red).

Our intention was to incorporate Cheng's dataset into our analysis through data integration, hypothesizing that this would unveil distinct macrophage subpopulations

not previously identified due to the analytical limitations of non-integrated approaches. However, this effort was constrained by the availability of only normalized data from their study. Without access to either raw data or raw counts, integrating Cheng's dataset into our analysis proved infeasible.

We also thank the reviewer for the comments regarding the cDC2_CXCL8 subcluster and its characterization in our manuscript. We acknowledge the need to clearly demonstrate the expression of key markers for accurate subcluster identification.

While CXCL8 expression was not prominently displayed in Figure 2b, it is indeed presented in our dot plots and heatmaps (Figures 2e and 2d), where the expression is clearly annotated. To address the concerns you have raised, we have prepared a revised density plot visualization (**Figure 2 for reviewers)**, which now more accurately depicts the CXCL8 expression pattern within the cDC_CXCL8 subcluster.

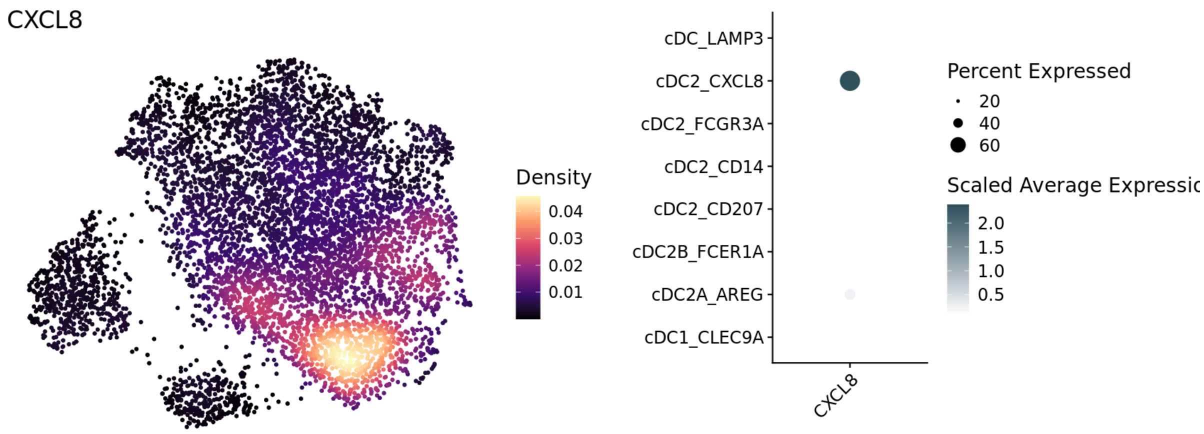

Figure 2 for reviewers: UMAP Visualization Highlighting CXCL8 Expression in Dendritic Cell Subpopulations. A) UMAP plot illustrates the density of *CXCL8* expression across various dendritic cell subpopulations. The color gradient represents expression density, with warmer colors indicating higher expression levels. B) Dot plot showing the *CXCL8* expression across various dendritic cell subpopulations. Dot size is associated with the percentage of cells expressing CXCL8 within each subpopulation, and color intensity with the scaled average expression level.

Regarding the classification of this subpopulation, although the comparison of gene expression profiles, as presented in the heatmap of Figure 2d, has shown that cDC_CXCL8 shares several differentially expressed genes with the cDC2_AREG population, it notably does not express *CD1C* (Figure 1e). Given this fact and also the points raised about the moDCs, we have decided to revisit the DCs classification, using as reference the seminal work of (Villani et al. 2017) using single-cells (**Figure 3 for reviewer**). This has not only enabled us to rename dendritic cell subpopulations with greater precision but also to clarify the novelty of the cDC-CXCL8 signature. Recognizing the complexity of dendritic cell heterogeneity, we agree that additional studies are required to fully decipher the cDC_CXCL8 subcluster's origin and relationship to established DC subtypes. The result section describing the DCs subpopulations has been updated to reflect the changes accordingly (lines 191-205).

A

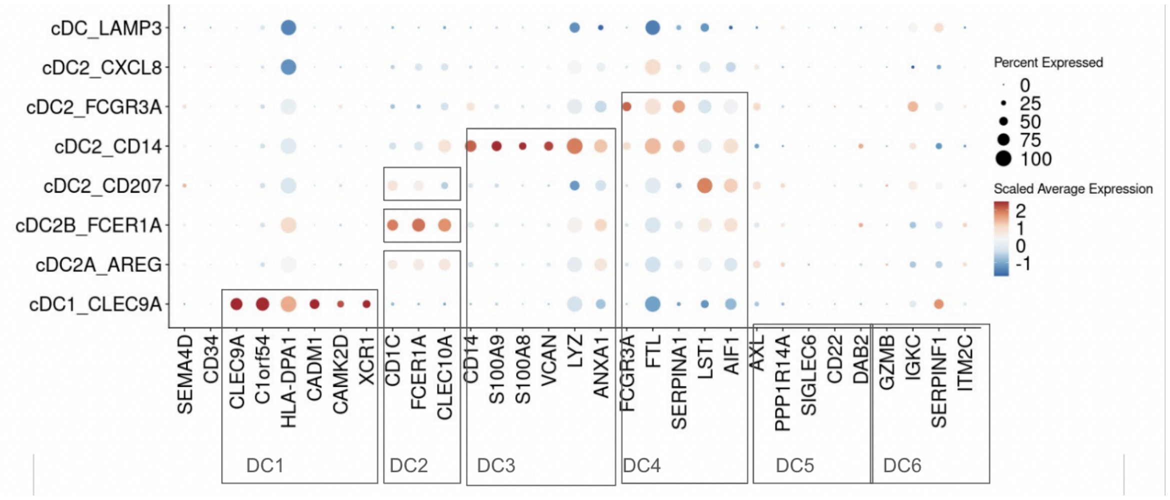

B

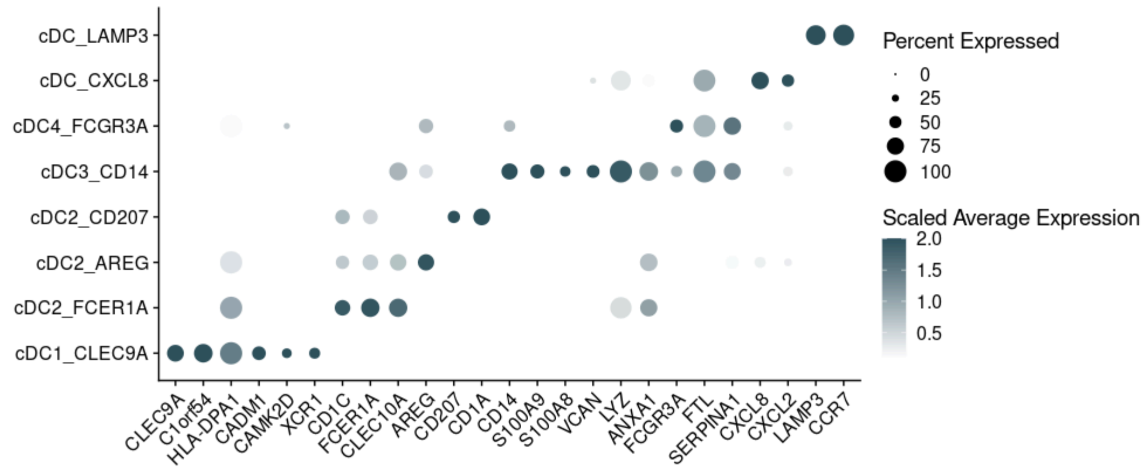

Figure 3 for reviewers: Expression Profiles of Dendritic Cell Subclusters. Dot plot representing gene expression profiles across various subclusters of dendritic cells (DCs) identified in our work. The rows denote genes associated **A)** with specific DC subtypes (DC1-DC6) defined by Vilani et al., and **B)** New DC's nomenclature based on signature's defined by Vilani et al of the populations identified in this work. The size of each dot corresponds to the percentage of cells within a subcluster expressing the gene (key at top right), and the color scale indicates the gene's scaled average expression level within that subcluster (key at bottom right).

In terms of validation, the cDC_CXCL8 subpopulation was identified in the independent validation cohort (Supplementary Figure 10 e-f).

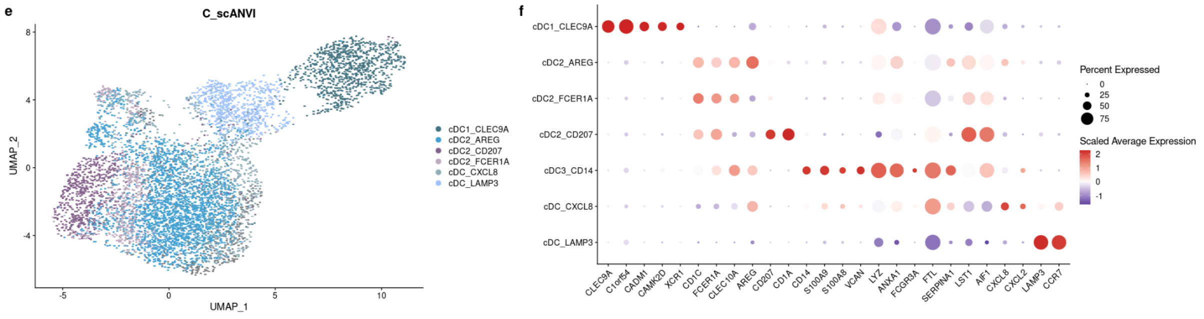

Supplementary Figure. 10. Comprehensive validation within a novel myeloid cell pan-cancer atlas (e) UMAP of DC subpopulations colored by the 7 states identified. (f) Dot plot showing the mean expression of genes related to DC subpopulations. Dot size indicates the percent of expressing cells, and the dot color the scaled average expression.

4. More discussion is needed on why the novel subcluster was not identified in Cheng et al.'s work, which included more cells than the current study.

The identification of novel subclusters in our study, in contrast to the work of (Cheng et al. 2021), despite their dataset encompassing a larger number of cells, can be attributed to several key factors.

Firstly, our study employed an integrated analysis strategy that combines data across multiple datasets, including those from different tumor types. This approach significantly enhances the statistical power to detect rare cell populations by increasing the sample heterogeneity and, consequently, the resolution at which cell populations can be distinguished (Stuart et al. 2019); (Butler et al. 2018); (Emert et al. 2021). The absence of data integration can result in a decreased ability to identify subtle, yet biologically significant, differences among cell populations. The novel subclusters identified in our study, therefore, may have emerged due to this heightened sensitivity to rare cell types that can often be masked in larger, but not integrated, datasets.

When checking if macrophage subpopulations identified in the previous work bear any of the signatures determined in our work, we observed that some cell types were distinctly displaying exclusive phenotype signatures such as the INF-primed and angiogenesis, while other signatures were diffused in their clusters (**Figure 1 for reviewers**). Our analysis suggests that some of the cell populations identified by Cheng et al. 2020 might represent composite signatures of what, upon deeper analysis with integrated datasets, can be resolved into distinct subpopulations.

To better prove our hypothesis we would need to integrate Cheng et al.'s dataset. However, our study had access to normalized data, which, while enabling certain types of comparative analysis, restricts the ability to perform a fully integrated analysis involving raw counts. The integration of Cheng et al. 's dataset with ours was not feasible due to these data availability issues, potentially limiting our capacity to directly compare our findings with theirs.

In regards to sample composition, we have build the following table so we can directly compare the two cohorts:

	Number of cells		
	Cheng et al.	Guimarães et al.	
		Exploratory cohort	Validation cohort
**Dendritic cells**	11,153 (18.50%)	6,212 (12.02%)	7,308 (7.65%)
**Macrophages**	25,310 (41.98%)	35,274 (68.25%)	70,285 (73.59%)
**Monocytes**	20,231 (33.55%)	10,201 (19.74%)	18,921 (19.81%)
**Total**	60,293	51,687	95,514
**Tumor Types**	BRCA<br>CRC<br>ESCA<br>HCC<br>KIDNEY<br>LUNG<br>LYM<br>MEL<br>MYE<br>NPC<br>OV-FTC<br>PAAD<br>STAD<br>THCA<br>UCEC	BRCA<br>COAD<br>LIHC<br>LUAD<br>LUSC<br>NSCLC<br>OV<br>SKCM<br>UVM	BLCA<br>BRCA<br>HCC<br>HNSC<br>KIDNEY<br>OV<br>PAAD<br>PRAD<br>STAD<br>THCA<br>UCEC

As observed by the table, Cheng et al.'s work comprises different tumor types and smaller percentage of macrophages, but higher of monocytes. These differences could also impact the differences in the results obtained in each work, besides the integration approach.

5. Re-clustering after filtering low-quality cells may affect the cluster pattern. Since all tumor types were integrated before cell type analysis, it is unclear whether re-clustering was performed after filtering low-quality cells. If so, a new UMAP figure should be provided.

Thank you for highlighting the importance of clarifying our methodology regarding the filtering of low-quality cells.

Indeed, after integrating all samples for cell type analysis, we filtered out low-quality cells to ensure the reliability and accuracy of our findings. We agree with your suggestion and we have replaced the previous UMAP by this updated version obtained after re-clustering to accurately capture the refined cellular landscape (Supplementary Figure 1d) in our revised manuscript.

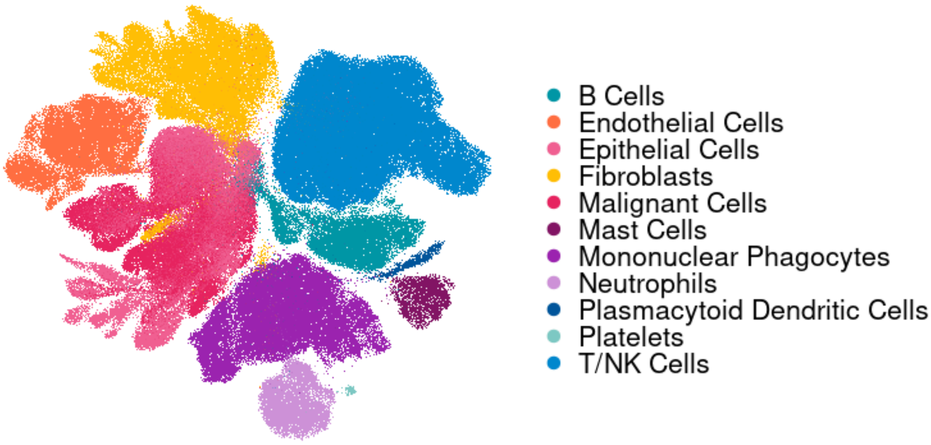

Supplementary Figure 1d: UMAP colour-coded by the eleven broad cell types: B cells (n = 22,102) were identified by the expression of MZB1 and MS4A1; Endothelial cells (n = 28,775) were marked by VWF and CCL21; MUC and S100A1 expression allowed the identification of normal (n = 56,060) and malignant (n = 47,827) epithelial cells, which were further distinguished by the amount of copy number variation (CNV) events observed in the malignant cells. Fibroblasts (n = 48,159) by COL1A1 and RGS5; Mast cells (n = 4,539), identified by TPSAB1; Mononuclear Phagocytes were positive for LYZ, AIF1, and CD68; Neutrophils (n = 12,033), marked by CXCR2; Plasmacytoid Dendritic Cells (pDC) (n = 1,365) by LILRA4 and IRF7; megakaryocytes (n = 185) marked by PPBP expression; and T and Natural Killer (NK) cells (n = 119,472) identified by the expression of IL7R or CD8A, or NKG7.

6. According to Supplementary Table 1, PBMCs were derived from only one patient in one study, which included no tumor samples. Comparing PBMCs from a healthy donor in one study with tumor samples from other studies is meaningless and introduces potential bias from batch effects.

Thank you for highlighting the issue with the representation of PBMC samples in our study. We recognize the oversight in our Supplementary Table 1 and 2 and the potential implications for our analysis's clarity and integrity.

To address your concern, we have corrected the information in Supplementary Table 2 to accurately reflect the PBMC samples' inclusion and context in our study. We have 4 studies (3 of them including tumor samples as well) included in our integrated dataset with a total of 11 PBMC samples. This update aligns our tables with the data presented in Figure 1b, ensuring consistency across our paper. We have also replaced "blood" by "PBMC" to have consistency in the supplementary table 1 and 2.

7. Figure 1g, key for sub clustering separation criteria, lacks detailed explanation in the main text, such as how each subcluster is defined, the cell number in each subcluster, and the gene markers for each subcluster.

We recognize the reviewer's concerns regarding the methodological parameters used to generate this representative figure of the myeloid-derived cells subpopulations. In order to better clarify it, we have replaced the figure by a new one, adjusted the legend and insert the following paragraph in the result section:

Line 170: *Additional unsupervised hierarchical clustering analysis of the MDCs subpopulations, anchored in 750 highly variable genes, substantiates the delineation of the principal five broad cell types (Fig. 1g). The dendrogram reinforce the proximity of mononuclear phagocytes and elucidates a closer gene expression similarity between macrophages and conventional dendritic cells compared to their expression relationship with monocytes. This observation suggests shared functional or ontogenic pathways between macrophages and dendritic cells.*

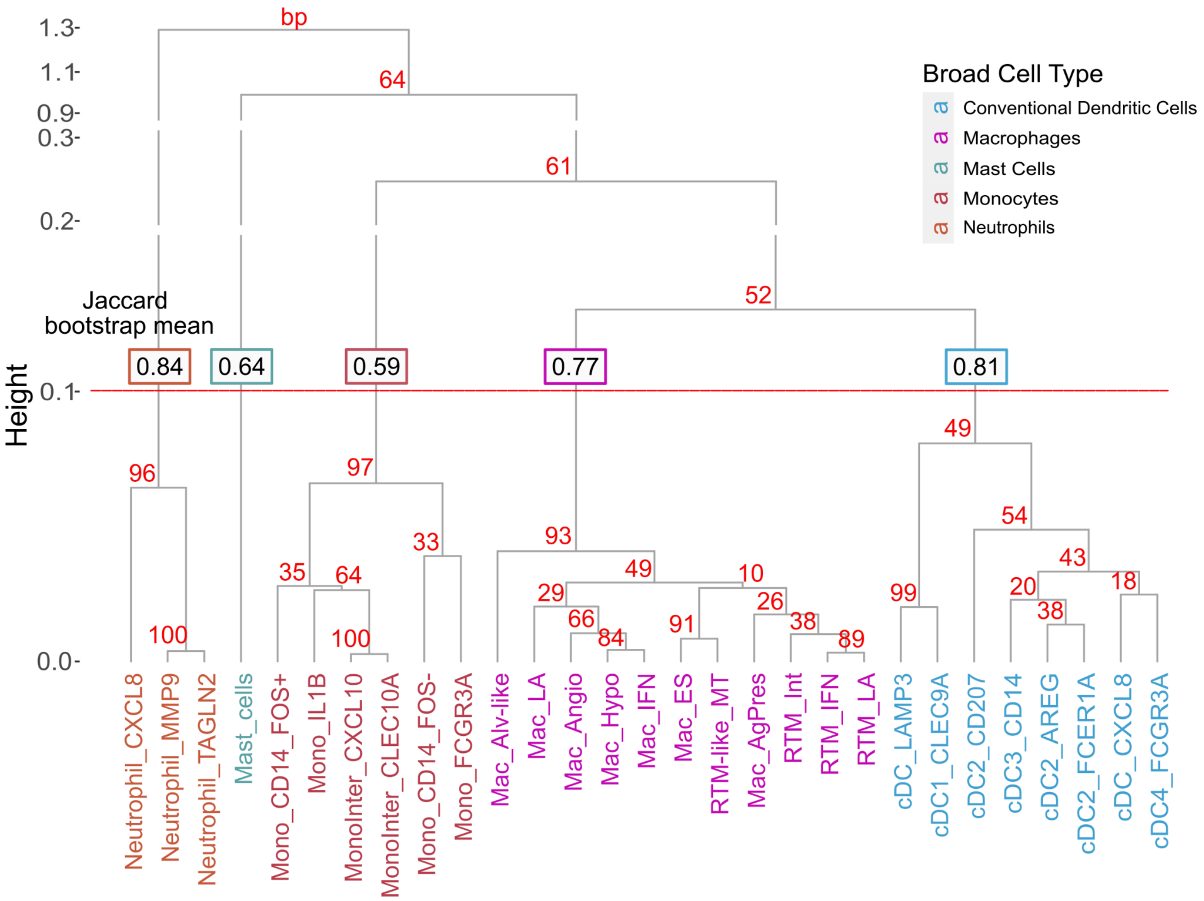

Figure 1g: Dendrogram represents the hierarchical clustering of MDCs based on their gene expression similarity. The height of the branches indicates the distance or dissimilarity between clusters, with a lower height reflecting greater similarity. The Jaccard bootstrap mean values are overlaid on the dendrogram, providing a measure of cluster stability based on resampling. Values closer to 1 indicate higher confidence. The dashed line across the dendrogram serves as a cut-off threshold for defining distinct clusters based on Jaccard similarity. The colored boxes correspond to broad cell types categorized by their predominant function or phenotype. Each terminal node of the dendrogram is labeled with the subcluster name derived from the expression of key marker genes. The values in red on each node represent the bootstrap percentage related to the confidence of the node's position. A total of 10.000 replicates were used for bootstrapping.

We also added a new item on methodology section related to clustering analysis mentioned above (line 850)

8. Are the neutrophils primarily derived from blood (Supplementary 2h)? Which study provided blood cells according to Supplementary Table 1? The total cell number of neutrophils is very small in normal samples, and the tissue types between normal and tumor samples differ, with the proportion of CXCL8+ neutrophils varying across different platforms (e.g., high in 10x). These factors hinder drawing the conclusion that "This last population was found to be proportionally more prevalent in tumors than in blood or normal tissues, particularly in ovarian and colorectal tumors." As a suggestion, the entire neutrophil section should be excluded from the manuscript, as no reliable results have been presented.

Upon reflecting on your feedback regarding the neutrophil analysis, we recognize the challenges and limitations you've outlined. Neutrophils were mainly recovered from blood or lung tumor samples from InDrop sequencing technology and we agree about the concerns raised, presenting significant hurdles for drawing robust conclusions about the prevalence of CXCL8+ neutrophils.

Given these considerations, we agree with your suggestion and have decided to remove the neutrophil section from our manuscript. We are grateful for your insightful feedback.

In line 186, we have replaced the previous text to: *Mast cells and neutrophils (Fig. 1c-e, and Supplementary Fig. 2) corresponded to the less frequent MDC subpopulations identified in our study, and given their low representativeness, they have not been further studied here in detail.*

9. Figure 2a is redundant, as Figure 2c provides the subcluster information. Moreover, the location of cells in UMAP can partially reflect the relationship distance between different subclusters. However, the positioning of moDC in the middle of different cDC2 subclusters suggests that different cDC2 subclusters are more similar to moDC rather than to each other, indicating potential issues in the integration process. A thorough check of the matrix is necessary, for example, the number of coding genes listed in the matrix file (~25000 or significantly more).

Thank you for pointing this out. In the last round of revision, it was suggested by reviewer 1 and reviewer 4 that we should have changed the name of the subpopulations previously named as “cDC2_CD14" and "cDC2_FCGR3A" to MoDCs. Because this change seems to have caused some confusion, we have re-examined our populations in the light of the signatures identified by (Villani et al. 2017). This has led us to align our previously named “cDC2_CD14" and "cDC2_FCGR3A" populations more precisely with the DC3 and DC4 subpopulations, respectively, as characterized in their study.

Regarding the populations ontogeny, (Villani et al. 2017) highlighted the potential for DC1, DC2, and DC3 subpopulations to originate from a circulating cDC progenitor, which they described as expressing CD100+CD34int. This finding enriches our understanding of DC3's ontogeny, providing an alternative to the well-recognized pathway from monocytes CD14+. Furthermore, corroborating evidence from the study by Brown et al. supports the notion that CDC2A and CDC2B share a common progenitor and are shaped into their distinct phenotypes by local environmental cues. In addition, the study by (Brown et al. 2019) reveals that T-bet–negative DCs (cDC2B) are marked by upregulation of genes typically associated with monocytes, such as Csf1r, Ccr2, and Cx3cr1. This molecular signature may contribute to the observed proximity of these DC populations in UMAP visualizations, as their shared lineage or functional attributes bring them closer within the multidimensional space. Important to mention that the non supervised hierarchical clustering analysis also supports the proximities seen in the UMAP (**Figure 1g).**

Based on this literature and our subsequent analyses, we have decided to rename the DC subpopulations once again, adopting the nomenclature that accurately reflects the signatures reported by (Villani et al. 2017). This adjustment ensures that our classification system remains consistent with the current literature and does not misrepresent the developmental lineage of these important immune cells.

A

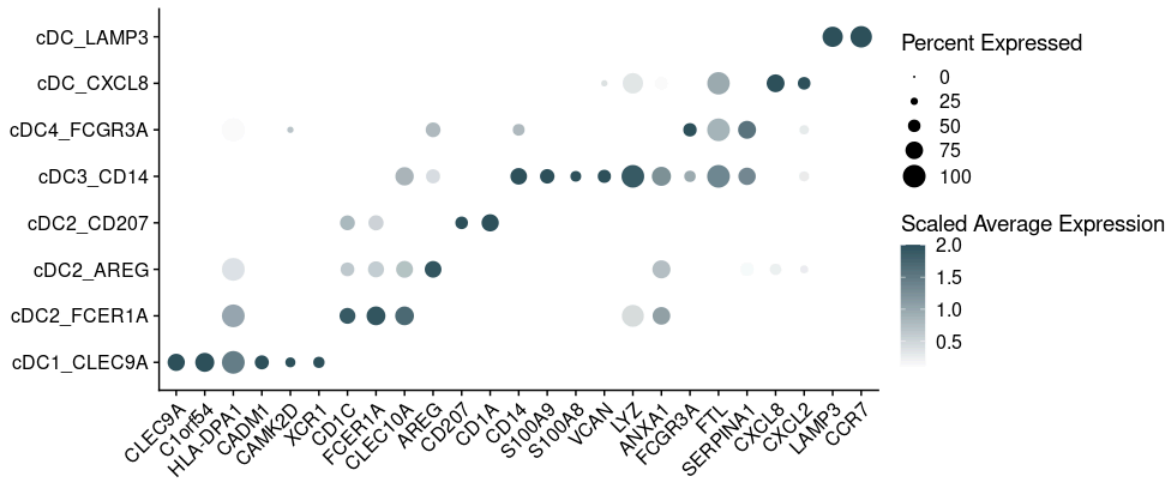

B

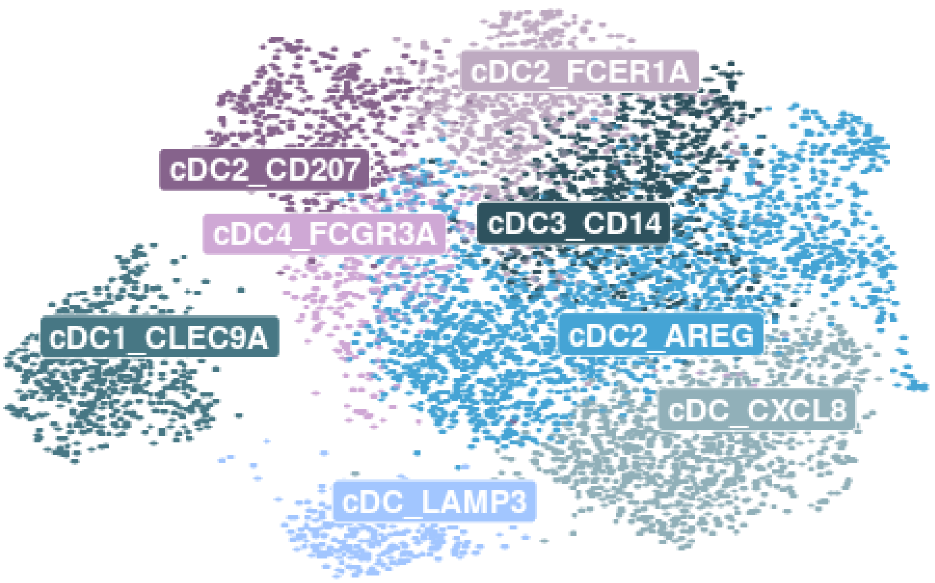

Figure 2: Expression Profiles of Dendritic Cell Subclusters. A) Dot plot representing gene expression profiles across various subclusters of dendritic cells (DCs) identified in our work that was renamed based on Vilani et al., . The size of each dot corresponds to the percentage of cells within a subcluster expressing the gene (key at top right), and the color scale indicates the gene's scaled average expression level within that subcluster (key at bottom right). **B)** UMAP represents cell population color by its cell types.

10. Dual-staining IHC is acceptable for validation (as shown in Figure 6g), and similar experiments should be conducted for FOLR2+ macrophages. Additionally, how to estimate the abundance of Mac_LA and RTM_INT in TCGA is lacking in method part? Considering many non-macrophage cells exhibit high expression of TREM2 according to Figure 6g, why use TREM2+/FOLR2+ protein levels rather than TREM2+/FOLR2+ macrophage levels for OS/PFS analysis?

Thank you for your feedback. Addressing your points:

In our revised manuscript, we have extended our analysis to include the validation of TREM2+ macrophage populations and their correlation with overall survival and progression-free survival in the triple-negative breast cancer (TNBC) cohort (Figure 6e-f). This approach is similar to the one we previously applied to the high-grade serous ovarian cancer (HGSOC) cohort (Supplementary Figure S16e). During this validation process, the quantification of TREM2 and CD68 co-staining was meticulously overseen by a board-certified pathologist. The establishment of thresholds for positive staining incorporated controls and leveraged the pathologist's expert judgment, with particular emphasis on their relevance to clinical and pathological outcomes.

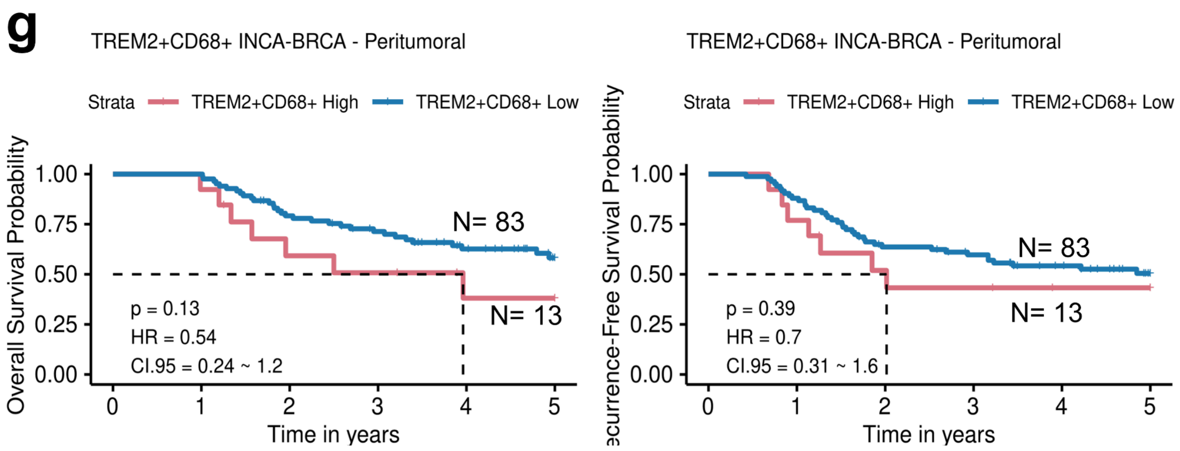

Figure 6. Impact of Mac_LA (TREM2+ CD68+) on clinical outcomes. Overall Survival (log-rank, p-value = 0.13) and Progression-Free Survival (log-rank, p-value = 0.39) in HIGH and LOW groups of TNBC samples co-stained for TREM2+ CD68+ from INCA cohort.

Addressing FOLR2, we encountered compatibility issues for dual staining with CD68, as both antibodies we have are rabbit-derived. However, considering that FOLR2 is a gene exclusively expressed in myeloid cells (Figure for reviewer 1), predominantly found in macrophages, we pursued an alternative validation strategy. We performed dual staining with FOLR2 and PDL2, another marker indicative of the RTM_Int macrophage subpopulation. This approach not only overcame the antibody compatibility issue but also significantly enhanced the specificity and accuracy of the impact mediated by RTM_Int subpopulation.

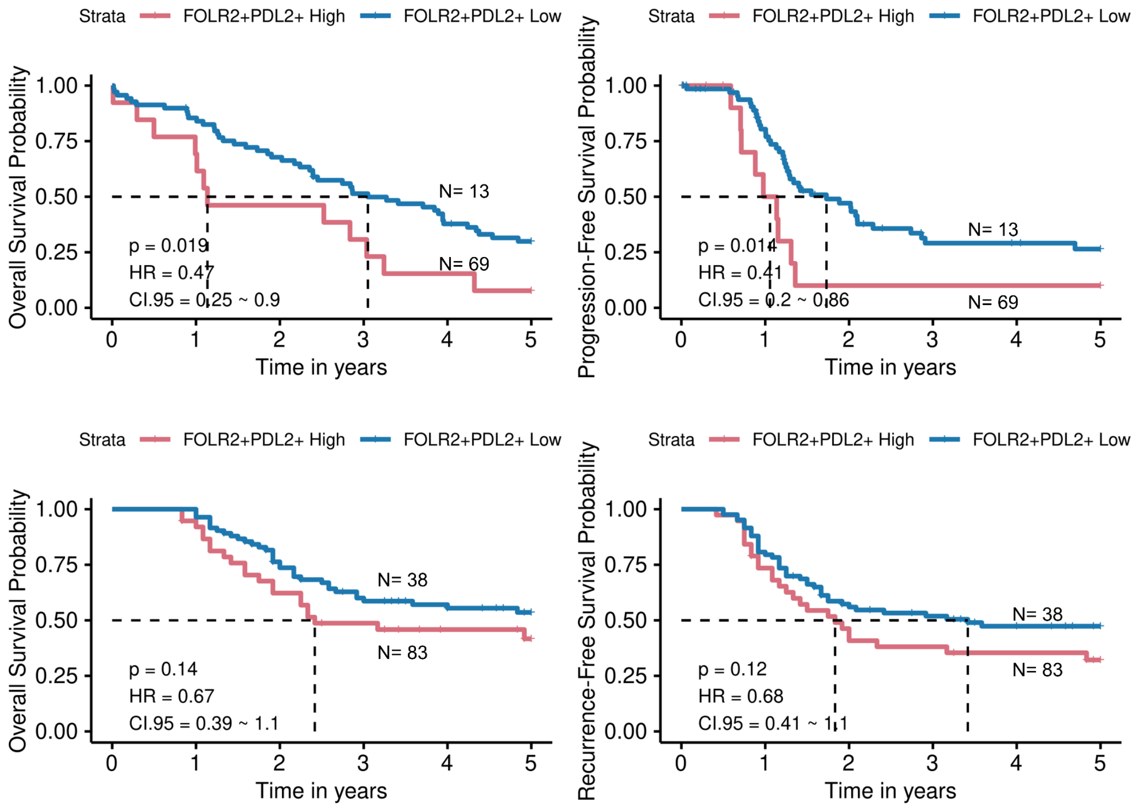

Figure 7. Impact of RTM_Int (FOLR2+ PDL2+) on clinical outcomes. Top row, Overall Survival (log-rank, p-value = 0.019) and Progression-Free Survival (log-rank, p-value = 0.014) in HIGH and LOW groups of HGSOC samples co-stained for FOLR2+ PDL2+ from INCA cohort. Bottom row, Overall Survival (log-rank, p-value = 0.14) and Event-Free Survival (log-rank, p-value = 0.12) in HIGH and LOW groups of TNBC samples co-stained for FOLR2+ PDL2+ from INCA cohort.

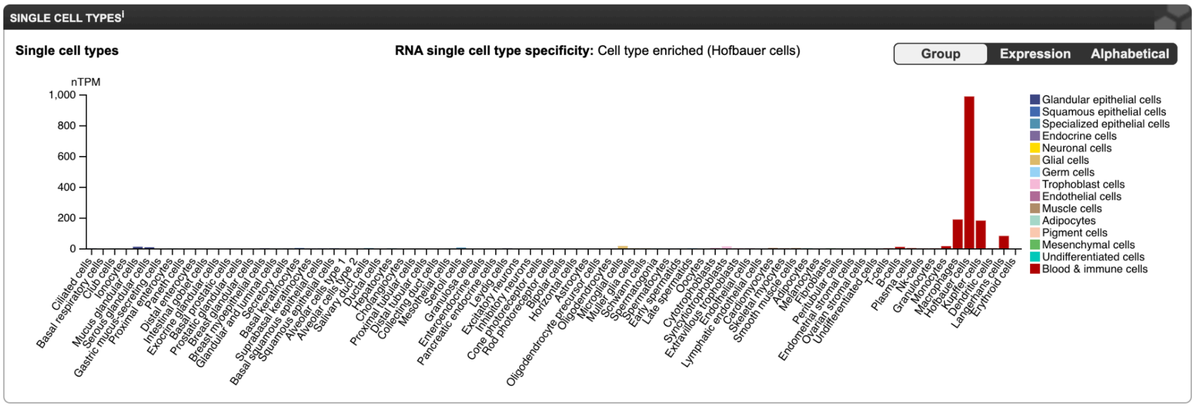

Figure 3 for reviewers: Single-Cell Type Specificity of FOLR2 Gene Expression. This bar graph illustrates the single-cell type specificity of FOLR2 gene expression across various cell types, as sourced from the Human Protein Atlas. The y-axis represents normalized Transcript Per Million (nTPM) values, indicating the expression level of the FOLR2 gene. Each bar corresponds to a different cell type, labeled on the x-axis, with colors designating distinct cell categories as defined in the key. Hofbauer cells are marked as the cell type with enriched expression of FOLR2, followed by macrophages. Data retrieved from the Human Protein Atlas (https://proteinatlas.org/ENSG00000165457-FOLR2/single+cell+type).

Regarding the point raised about the quantification of macrophage subpopulations in TCGA data, the process for determining the presence of specific macrophage subpopulations, such as Mac_LA (Lipid-Associated Macrophages) and RTM_INT (Interstitial Resident Macrophages), is described in the "Deconvolution Analysis" section. This includes detailing the deconvolution technique employed to discern these groups based on their unique transcriptional characteristics derived from single-cell data.

Lines 487 and 549 have been adjusted to include the new validations.

Minor Comments:

1. The number of myeloid cells, besides the total cells included in this study, should be presented in Supplementary Table .

Thank you for your comment. We have updated Supplementary Table 1 to include the number of myeloid cells, alongside the total cell count, to provide a complete overview of the cell populations analyzed in our study.

2. The font is still small in many figures, making it difficult to read.

Thank you for pointing out the issue with the font size in our figures. We have increased the font size to ensure readability and resubmit the revised figures with our manuscript (ex: Figure 1, Figure 6 and 7). Your feedback is greatly appreciated.

3. Since the number of different groups was not even, what criteria were used to separate different groups?

For the survival analysis, branches were defined using two approaches depending on how the data was collected:

1)The first approach was the use of the `surv_cutpoint` function from the `survminer` package to define groups. This function determines optimal cutpoints for continuous variables that are used to stratify patients into different risk groups. The `surv_cutpoint` function applies a maximally selected rank statistics method to identify the best-performing threshold based on survival outcomes, ensuring an unbiased separation of groups despite the uneven distribution of sample sizes. This approach was used to define groups in the following cases:

- Macrophage subpopulations estimations in bulk RNA-Seq samples from TCGA that were derived through deconvolution analysis. The cut-off points for Mac_LA, as determined by "surv_cutpoint", were a score of 0.11 for TNBC samples and 3.26 for HGSOC samples. As for RTM_Int, the cut-off was 9.67 for TNBC samples and 0.0078 for HGSOC samples.
- FOLR2 and TREM2 global levels quantified using the ImageJ software with the IHC Profiler plugin. The plugin facilitated the separation of color layers based on hematoxylin and 3,3′-Diaminobenzidine (DAB) staining. For each patient, the mean final score was calculated according to manual instructions taking the highest value among the calculated scores for high positive, positive, and low positive staining areas (Varghese et al. 2014).The cut-off points determined by "surv_cutpoint" were scores equal to 71.5 and 83.8 for TNBC samples when observing global expression levels of FOLR2 and TREM2, respectively.

- Tissue microarray slides of ovarian and breast cancer patients stained with hematoxylin, eosin, and co-marked with PDL-2 and FOLR2 were analyzed using HALO software version 3.6 (Indica Labs, Albuquerque, NM, USA). Spots were segmented using the TMA module and positive cells (displaying both markers) were quantified via the Multiplex IHC module by defining thresholds for each marker. Cell density per micrometer area for each spot was determined considering cell phenotype. The cut-offs determined by `surv_cutpoint` were 7.5 cells for HGSOC samples and 53.5 for TNBC samples.

2) For the evaluation of double staining of TREM2 and CD68, the quantification of markers was performed under the supervision of a qualified pathologist. The cutoffs for positive staining were established based on positive and negative controls, as well as her experience as a pathologist. These cutoffs were chosen based on their clinical and pathological significance. The precise cutoff values used in our analysis are detailed within our manuscript and were consistently applied across all evaluated slides to maintain methodological consistency.
For the HGSOC tissue microarrays cohort, the markers and their corresponding thresholds for classification in the were as follows:

- TREM2 and CD68 co-staining: Samples were classified as low if 0 cells were positive and high if 10% or more cells exhibited positive staining.
- FOLR2: Low expression was defined as 25% or fewer cells being positive, while high expression was indicated by more than 75% of cells showing positive staining.
- PD-1: Expression was considered low if between 0 to 1% of cells were positive, and high if 3 to 30% of cells showed positive staining.
- CD8: A low classification corresponded to 0 to 1% positive cells, and a high classification was applied if 3% or more cells were positive.

For the TNBC tissue microarrays cohort, the markers and their corresponding thresholds for classification in the were as follows:

- TREM2 and CD68 co-staining: Peritumoral samples were classified as low if less than 1% of the cells were positive and high if 2% or more cells stained positive.

- PD-1: Expression was considered low if between 0 to 1% of cells were positive, and high if 1% or more of cells showed positive staining.

- CD8: A low classification corresponded to 0 to 1% positive cells, and a high classification was applied if 5% or more cells were positive.

Lines 1059 to 1116 have been modified to enhance clarity.

4. What is the cell type of malignant cells? Were TME cells included?

In our investigation, we conducted an analysis of Copy Number Variations (CNVs) to categorize epithelial cells, identified in our dataset, into malignant or non-malignant groups. This was performed through the inferCNVpy software (version 0.4.0), as described at github.com/icbi-lab/infercnvpy. This software extends the original inferCNV approach, which leverages scRNA-seq data to deduce CNVs based on variations in gene expression levels that may be indicative of gene copy number alterations. Such patterns can pinpoint cell subpopulations with shared CNV signatures, potentially indicating clonal expansions in cancers.

Our methodology proceeded in distinct phases: initially, we computed CNV scores for all cells within our dataset, encompassing epithelial, immune, and stromal cells. Subsequently, we focused on epithelial cells, establishing a threshold for malignancy classification based on the median CNV score derived from all cell types, which was determined to be 0.004 (as illustrated in Supplementary Fig. 1c). Epithelial cells exhibiting CNV scores surpassing this median value were categorized as malignant, inferring the existence of genomic aberrations commonly associated with cancerous cells. Conversely, cells falling below this median score were classified as non-malignant, suggesting a lack of such cancer-specific genomic alterations.

For clarity, line 839-848 has been changed to: *To differentiate between malignant and non-malignant epithelial cells, our study utilized an analysis of Copy Number Variations (CNVs). We estimated the CNV score for individual cells by applying inferCNVpy software (v0.4.0), available at github.com/icbi-lab/infercnvpy. This tool is an extension of the principles used in inferCNV (Tickle et al. 2019). We defined the malignancy status of epithelial cells based on their relative CNV scores that were calculated considering all cells in the dataset. Specifically, epithelial cells with a CNV*

score below the established median threshold of 0.004 were deemed "non-malignant." In contrast, those exhibiting a CNV score above this median were classified as "malignant," suggesting the presence of genomic changes commonly associated with cancer cells.

Additional Changes

We have updated Figure 7 (element l and m) to a new volcano plot to more effectively convey the differences in gene expression between RTM_Int found in tumor tissues versus normal tissues. Violin plots were also updated to illustrate the expression variance within each group.

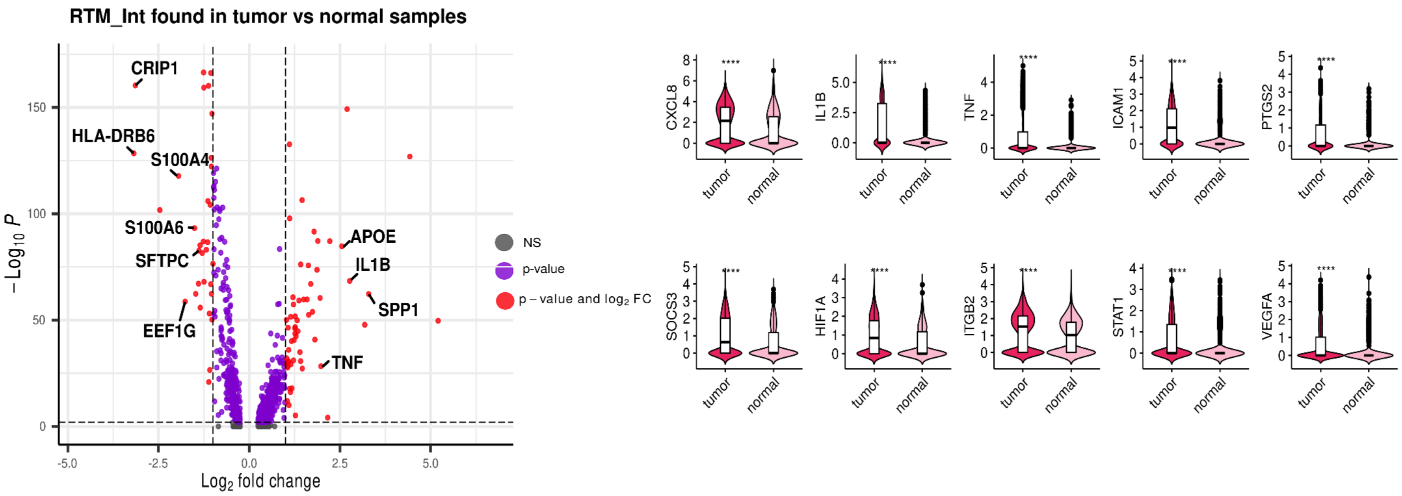

Figure 7. Impact of RTM_Int (FOLR2+) on clinical outcomes. (l) Volcano Plot between RTM-IMs found in tumor vs normal samples (p-value =< 0.05, cutoff FC =< 1). (m) Violin plot of genes belonging to the IL4 and IL13 signaling according to Reactome database that are differentially expressed across the conditions (**** p < 0.0001).

REFERENCES

Bassez, Ayse, Hanne Vos, Laurien Van Dyck, Giuseppe Floris, Ingrid Arijs, Christine Desmedt, Bram Boeckx, et al. 2021. “A Single-Cell Map of Intratumoral Changes during Anti-PD1 Treatment of Patients with Breast Cancer.” *Nature Medicine* 27 (5): 820–32.

Brown, Chrysothemis C., Herman Gudjonson, Yuri Pritykin, Deeksha Deep, Vincent-Philippe Lavallée, Alejandra Mendoza, Rachel Fromme, et al. 2019. “Transcriptional Basis of Mouse and Human Dendritic Cell Heterogeneity.” *Cell* 179 (4): 846–63.e24.

Butler, Andrew, Paul Hoffman, Peter Smibert, Efthymia Papalexi, and Rahul Satija. 2018. “Integrating Single-Cell Transcriptomic Data across Different Conditions, Technologies, and Species.” *Nature Biotechnology* 36 (5): 411–20.

Cheng, Sijin, Ziyi Li, Ranran Gao, Baocai Xing, Yunong Gao, Yu Yang, Shishang Qin, et al. 2021. “A Pan-Cancer Single-Cell Transcriptional Atlas of Tumor Infiltrating Myeloid Cells.” *Cell* 184 (3): 792–809.e23.

Emert, Benjamin L., Christopher J. Cote, Eduardo A. Torre, Ian P. Dardani, Connie L. Jiang, Naveen Jain, Sydney M. Shaffer, and Arjun Raj. 2021. “Variability within Rare Cell States Enables Multiple Paths toward Drug Resistance.” *Nature Biotechnology* 39 (7): 865–76.

Hie, Brian, Bryan Bryson, and Bonnie Berger. 2019. “Efficient Integration of Heterogeneous Single-Cell Transcriptomes Using Scanorama.” *Nature Biotechnology* 37 (6): 685–91.

Hornburg, Milena, Mélanie Desbois, Shan Lu, Yinghui Guan, Amy A. Lo, Susan Kaufman, Ashley Elrod, et al. 2021. “Single-Cell Dissection of Cellular Components and Interactions Shaping the Tumor Immune Phenotypes in Ovarian Cancer.” *Cancer Cell* 39 (7): 928–44.e6.

Korsunsky, Ilya, Nghia Millard, Jean Fan, Kamil Slowikowski, Fan Zhang, Kevin Wei, Yuriy Baglaenko, Michael Brenner, Po-Ru Loh, and Soumya Raychaudhuri. 2019. “Fast, Sensitive and Accurate Integration of Single-Cell Data with Harmony.” *Nature Methods* 16 (12): 1289–96.

Liu, Baolin, Chenwei Li, Ziyi Li, Dongfang Wang, Xianwen Ren, and Zemin Zhang. 2020. “An Entropy-Based Metric for Assessing the Purity of Single Cell Populations.” *Nature Communications* 11 (1): 3155.

Lopez, Romain, Jeffrey Regier, Michael B. Cole, Michael I. Jordan, and Nir Yosef. 2018. “Deep Generative Modeling for Single-Cell Transcriptomics.” *Nature Methods* 15 (12): 1053–58.

Luecken, Malte D., M. Büttner, K. Chaichoompu, A. Danese, M. Interlandi, M. F. Mueller, D. C. Strobl, et al. 2022. “Benchmarking Atlas-Level Data Integration in Single-Cell Genomics.” *Nature Methods* 19 (1): 41–50.

Mulder, Kevin, Amit Ashok Patel, Wan Ting Kong, Cécile Piot, Evelyn Halitzki, Garett Dunsmore, Shabnam Khalilnezhad, et al. 2021. “Cross-Tissue Single-Cell Landscape of Human Monocytes and Macrophages in Health and Disease.” *Immunity* 54 (8): 1883–1900.e5.

Olalekan, Susan, Bingqing Xie, Rebecca Back, Heather Eckart, and Anindita Basu. 2020. “Elucidating the Immune Response in Metastatic Ovarian Cancer Using Single Cell Transcriptomics.” *Journal of Immunology* 204 (1_Supplement): 243.16–243.16.

Pal, Bhupinder, Yunshun Chen, François Vaillant, Bianca D. Capaldo, Rachel Joyce, Xiaoyu Song, Vanessa L. Bryant, et al. 2021. “A Single-Cell RNA Expression

Atlas of Normal, Preneoplastic and Tumorigenic States in the Human Breast." *The EMBO Journal* 40 (11): e107333.
Ryu, Yeonjae, Geun Hee Han, Eunsoo Jung, and Daehee Hwang. 2023. "Integration of Single-Cell RNA-Seq Datasets: A Review of Computational Methods." *Molecules and Cells* 46 (2): 106–19.
Schelker, Max, Sonia Feau, Jinyan Du, Nav Ranu, Edda Klipp, Gavin MacBeath, Birgit Schoeberl, and Andreas Raue. 2017. "Estimation of Immune Cell Content in Tumour Tissue Using Single-Cell RNA-Seq Data." *Nature Communications* 8 (1): 2032.
Sikkema, Lisa, Ciro Ramírez-Suástegui, Daniel C. Strobl, Tessa E. Gillett, Luke Zappia, Elo Madissoon, Nikolay S. Markov, et al. 2023. "An Integrated Cell Atlas of the Lung in Health and Disease." *Nature Medicine* 29 (6): 1563–77.
Slyper, Michal, Caroline B. M. Porter, Orr Ashenberg, Julia Waldman, Eugene Drokhlyansky, Isaac Wakiro, Christopher Smillie, et al. 2020. "A Single-Cell and Single-Nucleus RNA-Seq Toolbox for Fresh and Frozen Human Tumors." *Nature Medicine* 26 (5): 792–802.
Stuart, Tim, Andrew Butler, Paul Hoffman, Christoph Hafemeister, Efthymia Papalexi, William M. Mauck 3rd, Yuhan Hao, Marlon Stoeckius, Peter Smibert, and Rahul Satija. 2019. "Comprehensive Integration of Single-Cell Data." *Cell* 177 (7): 1888–1902.e21.
Tickle, T., I. Tirosh, C. Georgescu, M. Brown, and B. Haas. 2019. "inferCNV of the Trinity CTAT Project." *Klarman Cell Observatory, Broad Institute of MIT and Harvard, Cambridge, MA, USA*.
Varghese, Frency, Amirali B. Bukhari, Renu Malhotra, and Abhijit De. 2014. "IHC Profiler: An Open Source Plugin for the Quantitative Evaluation and Automated Scoring of Immunohistochemistry Images of Human Tissue Samples." *PloS One* 9 (5): e96801.
Villani, Alexandra-Chloé, Rahul Satija, Gary Reynolds, Siranush Sarkizova, Karthik Shekhar, James Fletcher, Morgane Griesbeck, et al. 2017. "Single-Cell RNA-Seq Reveals New Types of Human Blood Dendritic Cells, Monocytes, and Progenitors." *Science* 356 (6335). https://doi.org/10.1126/science.aah4573.
Wu, Sunny Z., Ghamdan Al-Eryani, Daniel Lee Roden, Simon Junankar, Kate Harvey, Alma Andersson, Aatish Thennavan, et al. 2021. "A Single-Cell and Spatially Resolved Atlas of Human Breast Cancers." *Nature Genetics* 53 (9): 1334–47.
Xu, Chenling, Romain Lopez, Edouard Mehlman, Jeffrey Regier, Michael I. Jordan, and Nir Yosef. 2021. "Probabilistic Harmonization and Annotation of Single-Cell Transcriptomics Data with Deep Generative Models." *Molecular Systems Biology* 17 (1): e9620.
Zhang, Kaiyang, Erdogan Pekcan Erkan, Sanaz Jamalzadeh, Jun Dai, Noora Andersson, Katja Kaipio, Tarja Lamminen, et al. 2022. "Longitudinal Single-Cell RNA-Seq Analysis Reveals Stress-Promoted Chemoresistance in Metastatic Ovarian Cancer." *Science Advances* 8 (8): eabm1831.

REVIEWER COMMENTS

Reviewer #3 (Remarks to the Author):

The authors have addressed all my comments.

Reviewer #4 (Remarks to the Author):

The revised manuscript has largely improved. However, further improvements are needed, which are listed below.

1. Different platforms render the proportion analysis unreliable (e.g., 2f, 3f, and 4e), because the proportion of clusters are largely impacted by the different platforms. It better to deletion the figures and descriptions on comparisons for proportion. If it is important for the study, separating the illustration in terms of platforms.
2. Although renamed in DCs, it is still strange that cDC3 and cDC4 located between different cDC2 subclusters. And the author needs to reply to the number of the genes in the matrix. Technically, different studies may provide different gene symbols for the same gene. Carefully check the gene symbols are particularly important. For instance, xxx, xxx.1 and xxx-1 would be treated as three different genes when integrating the matric data. In addition, MT, ribosome proteins related genes and lncRNAs, variation of which are more likely induced by sample preparation rather than marker for clustering, would greatly impact the clustering process, particularly the small clusters and study-specific clusters. After integrating the gene symbols and filtering these genes, mostly 25000-30000 genes were left for clustering.
3. I didn’t find a supplementary table that list all the top signature genes (normally top 20-30 genes those are significantly enriched) for each cluster, which should be listed as a supplementary table.

RESPONSE TO REVIEWERS' COMMENTS

We are grateful for the reviewer's constructive comments and valuable observations.

Reviewer #3 (Remarks to the Author):

The authors have addressed all my comments.

Reviewer #4 (Remarks to the Author):

The revised manuscript has largely improved. However, further improvements are needed, which are listed below.

We greatly appreciate your detailed feedback and suggestions for further improvements to our manuscript. Please find below our responses to your comments:

1. Different platforms render the proportion analysis unreliable (e.g., 2f, 3f, and 4e), because the proportion of clusters are largely impacted by the different platforms. It better to deletion the figures and descriptions on comparisons for proportion. If it is important for the study, separating the illustration in terms of platforms.

We acknowledge your concern about the variability introduced by different platforms. In response, we have opted to consider your suggestion to segregate the illustrations based on the platform used, thereby minimizing potential biases on interpretation. Figures 2, 3 and 4 and their respective legends have been revised to incorporate this modification.

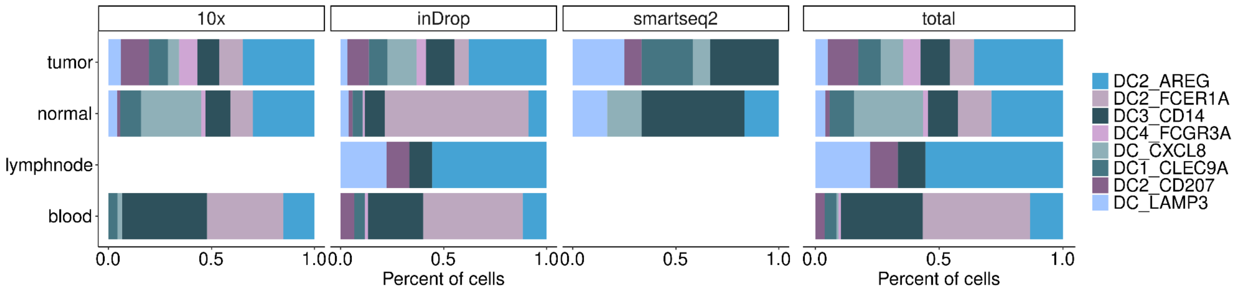

Figure 2e: Bar plot showing the distribution of DC cells across sample types by three different sequencing platforms (10x, inDrop, Smart-seq2), with an aggregated total.

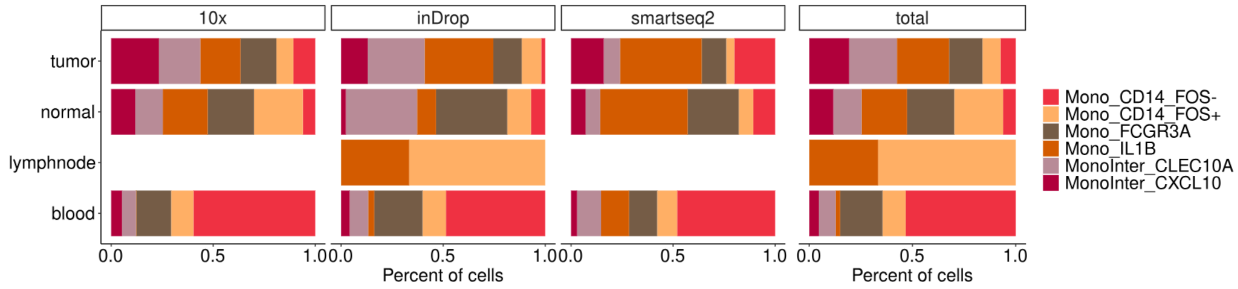

Figure 3f: Bar plot showing the distribution of monocytes across sample types by three different sequencing platforms (10x, inDrop, Smart-seq2), with an aggregated total.

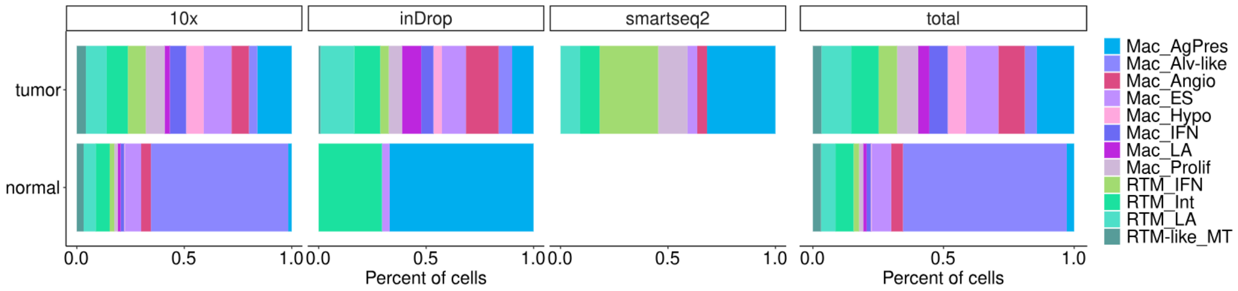

Figure 4e: Bar plot showing the distribution of macrophages across sample types by three different sequencing platforms (10x, inDrop, Smart-seq2), with an aggregated total.

2. Although renamed in DCs, it is still strange that cDC3 and cDC4 located between different cDC2 subclusters. And the author needs to reply to the number of the genes in the matrix. Technically, different studies may provide different gene symbols for the same gene. Carefully check the gene symbols are particularly important. For instance, xxx, xxx.1 and xxx-1 would be treated as three different genes when integrating the matric data. In addition, MT, ribosome proteins related genes and lncRNAs, variation of which are more likely induced by sample preparation rather than marker for clustering, would greatly impact the clustering process, particularly the small clusters and study-specific clusters. After integrating the gene symbols and filtering these genes, mostly 25000-30000 genes were left for clustering.

Thank you for your feedback. In response to your comment, we have reviewed the positioning of DCs and gene names. With respect to gene names/symbols, we have

performed an exhaustive verification to ensure both consistency and accuracy across our datasets. As detailed in the "Data Download and Pre-Processing" section of our methods, we have standardized gene IDs to the hg38 genome version from Ensembl database prior to the integration of the datasets, crucial for the harmonization of data originating from diverse sources. To achieve this, we employed the AnnotationDbi tool in conjunction with the EnsDb.Hsapiens.v86 package. This resource includes an extensive repertoire of gene and transcript information, consisting of 63,970 genes and 216,741 transcripts. We applied the same conversion process uniformly to the datasets of our validation cohort as well. Post-integration, our datasets encompassed a total of 45,456 genes (Figure 1A for reviewers). For the specific task of clustering DC subsets, we utilized a select group of 3000 highly variable genes (Figure 1B for reviewers), which are listed in Table 1 (appended to this letter), available for reviewers' examination. The majority of the genes used for clustering and UMAP representation are protein-coding genes.

A

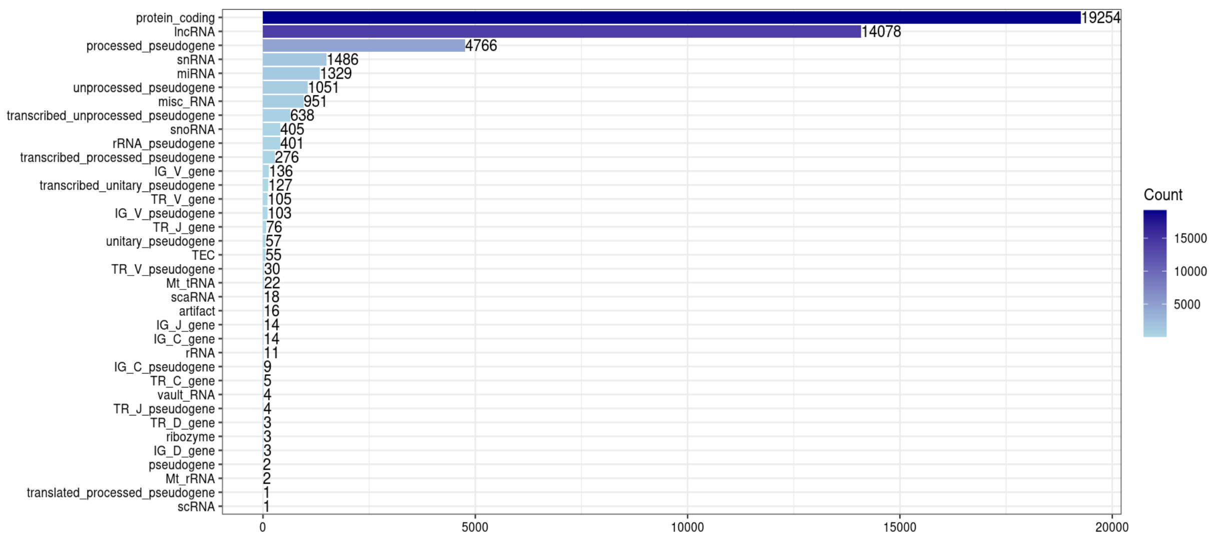

B

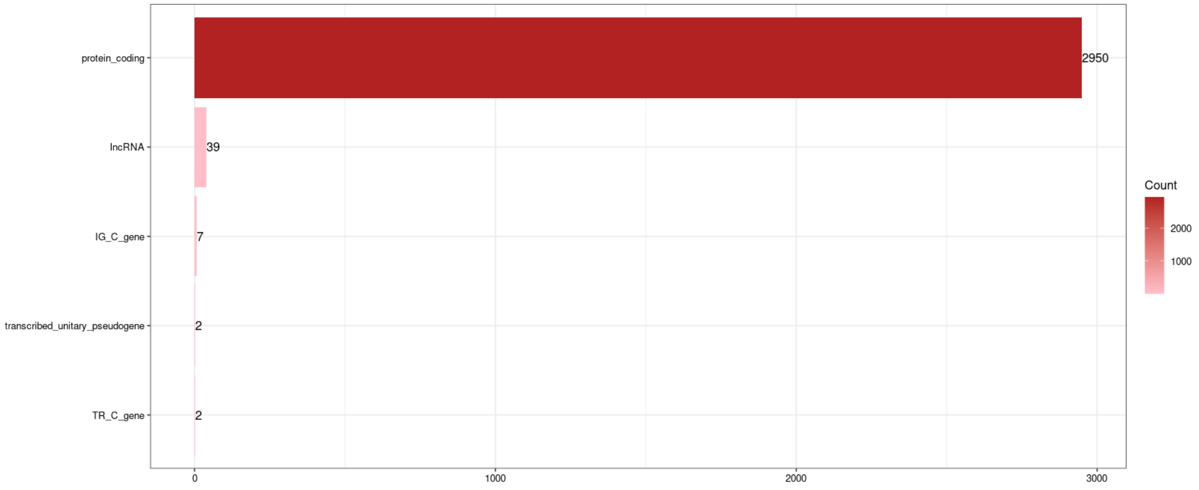

Figure 1 for reviewers: Bar chart illustrating the distribution of various gene types in the analyzed dataset. A) Total genes after integration; B) Highly variable genes used for Dendritic cells clustering. Each bar represents a distinct gene category, with the height corresponding to the count of genes within that category. The categories are listed on the Y-axis, and the counts are shown on the X-axis. The color intensity of each bar correlates with the gene count, as indicated by the 'Count' gradient legend on the right.

Regarding the proximity of DC3 and DC4 to DC2 subclusters, we believe that this observation is consistent with recent advances in dendritic cell biology [1,2,3,4,5]. Several studies, including those by Villani et al. (2017), Satoh, T. et al. (2021) and Cabeza-Cabrerizo et al. (2021), have identified DC3 as a subpopulation of cDC2, based on shared phenotypic and functional characteristics. These findings are supported by the continuum model proposed by Dutertre et al. (2019), which includes classical monocytes and cDC2s, encompassing the DC2 and DC3 subpopulations: *"Classical monocytes (cMos) and CD1c+ cDC2s, which comprise subpopulations DC2 and DC3, are phenotypically related and form a continuum* [3]*.* Ontogenetically, the DC3 and DC4 subsets are suggested to derive from monocytes, as they share key monocyte-associated genes (*CD14* and *FCGR3A*/CD16, respectively) [4]. Moreover, Villani et al. (2017) [4] outlined a dual progenitor model to cDC2 subclusters that can be derived by monocytes and circulating cDC

progenitors. Villani et al. (2017) have highlighted the potential for DC1, DC2, and DC3 subpopulations to arise from a circulating cDC progenitor, providing a broader understanding of DC3's lineage, which serves as an alternative pathway to the well-recognized monocyte differentiation route.

The convergence of DC3 and DC4 with cDC2 subclusters is not solely phenotypical but also reflected in the shared ontogenetic pathways, probably explaining their proximity in UMAP visualizations. This proximity was also observed in the UMAP from our independent cohort used for validation (Supplementary Figure 9), and in the UMAP reported by Cabeza-Cabrerizo et al. (2021), corroborating the patterns found in our main Figure 2a.

To further verify these similarities and relationships among DC clusters, we conducted a correlation analysis among the subpopulations and also performed a non-supervised hierarchical clustering analysis with bootstrap, now considering the same 3000 highly variable genes used for the UMAP construction of DCs. Both the correlation and dendrogram analyses reinforce the proximity of DC3 and DC4 to DC2 clusters (Figure 2 for Reviewers).

A

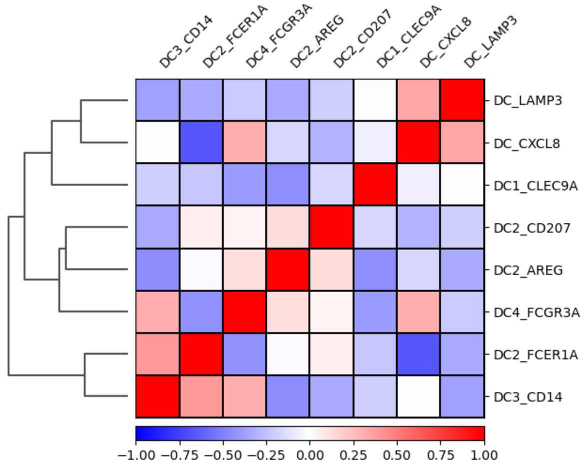

B

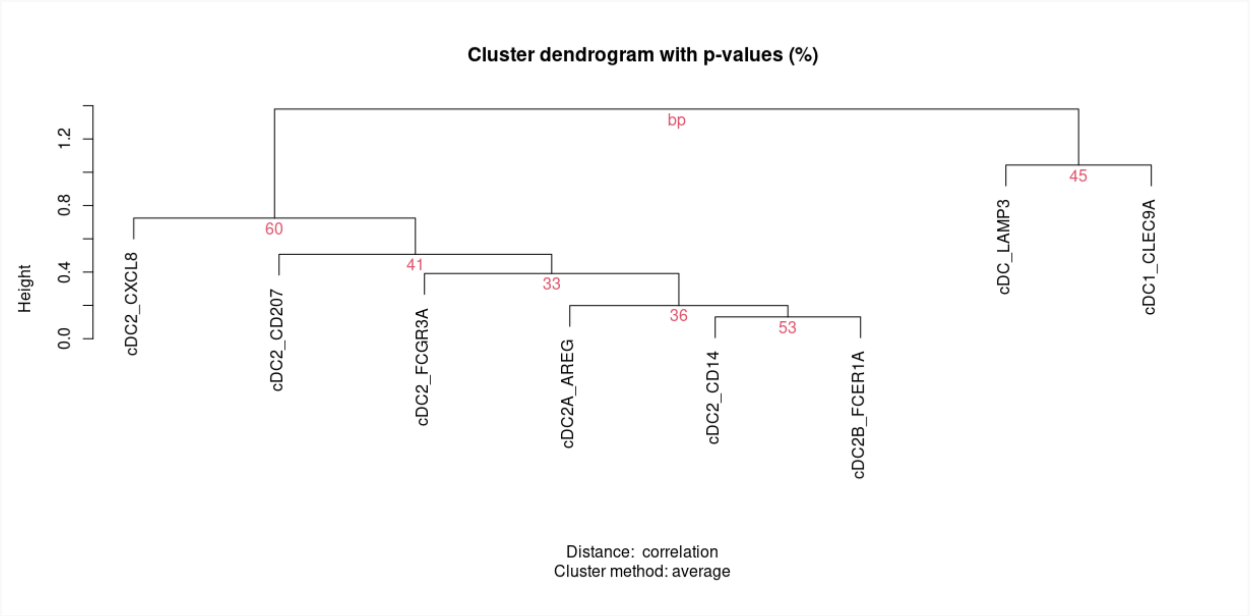

Figure 2 for reviewers: Analysis of Dendritic Cell Subcluster Gene Expression Correlations and Hierarchical Clustering Validation. A) Heatmap displaying the correlation matrix between different DC subclusters and selected marker genes. The intensity of the color reflects the correlation coefficient, which was obtained using the scVI latent space generated after the integration, ranging from -1.00 (strong negative correlation, blue) to +1.00 (strong positive correlation, red). B) Cluster dendrogram with bootstrap p-values expressed as percentages. The dendrogram represents the hierarchical clustering of DC subclusters based on the correlation of gene expression profiles. The height of the branch points reflects the distance or dissimilarity between clusters, with lower heights indicating higher similarity. Bootstrap p-values are indicated next to the branches, quantifying the statistical support for each cluster. The closer the p-value is to 100%, the stronger the support for the cluster.

To further clarify on DCs classification and nomenclature, we have included a second UMAP in Figure 2.

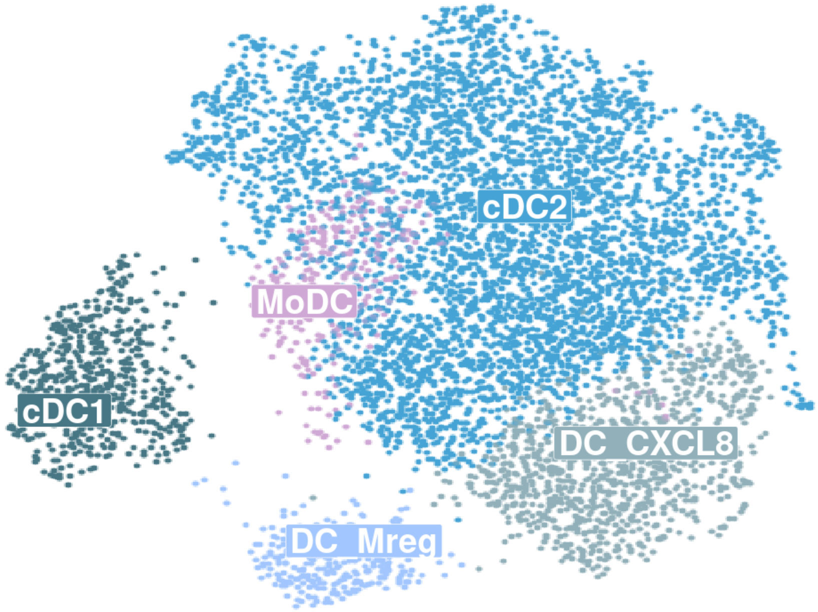

Figure 2a: UMAP color-coded by the broad classification of dendritic cells.

3. I didn't find a supplementary table that list all the top signature genes (normally top 20-30 genes those are significantly enriched) for each cluster, which should be listed as a supplementary table.

A new supplementary Table 5 has now been added to the manuscript, providing details of the top 30 significantly enriched genes for each cluster.

References

1. Satoh, T. *et al.* Human DC3 Antigen Presenting Dendritic Cells From Induced Pluripotent Stem Cells. *Front Cell Dev Biol* **9**, 667304 (2021).
2. Cabeza-Cabrerizo, M., Cardoso, A., Minutti, C. M., Pereira da Costa, M. & Reis e Sousa, C. Dendritic Cells Revisited. *Annu. Rev. Immunol.* **39**, 131–166 (2021).
3. Dutertre, C.-A. *et al.* Single-Cell Analysis of Human Mononuclear Phagocytes Reveals Subset-Defining Markers and Identifies Circulating Inflammatory Dendritic Cells. *Immunity* **51**, 573–589.e8 (2019).
4. Villani, A.-C. *et al.* Single-cell RNA-seq reveals new types of human blood dendritic cells, monocytes, and progenitors. *Science* **356**, (2017).
5. Kvedaraite, E. & Ginhoux, F. Human dendritic cells in cancer. *Sci Immunol* **7**, eabm9409 (2022).

REVIEWERS' COMMENTS

Reviewer #4 (Remarks to the Author):

I have no further comment
